# Quantitative analysis of cryptic splicing associated with TDP-43 depletion

**DOI:** 10.1186/s12920-017-0274-1

**Published:** 2017-05-26

**Authors:** Jack Humphrey, Warren Emmett, Pietro Fratta, Adrian M. Isaacs, Vincent Plagnol

**Affiliations:** 1 University College London Genetics Institute, Gower Street, London, UK; 2 Department of Neurodegenerative Disease, UCL Institute of Neurology, Queen Square, London, UK; 3 Department of Molecular Neuroscience, UCL Institute of Neurology, Queen Square, London, UK; 4 The Francis Crick Institute, Midland Road, London, UK; 5 Department of Motor Neuroscience and Movement Disorders, UCL Institute of Neurology, Queen Square, London, UK

**Keywords:** RNA-seq, Cryptic exons, Splicing, TDP-43

## Abstract

**Background:**

Reliable exon recognition is key to the splicing of pre-mRNAs into mature mRNAs. TDP-43 is an RNA-binding protein whose nuclear loss and cytoplasmic aggregation are a hallmark pathology in amyotrophic lateral sclerosis and frontotemporal dementia (ALS/FTD). TDP-43 depletion causes the aberrant inclusion of cryptic exons into a range of transcripts, but their extent, relevance to disease pathogenesis and whether they are caused by other RNA-binding proteins implicated in ALS/FTD are unknown.

**Methods:**

We developed an analysis pipeline to discover and quantify cryptic exon inclusion and applied it to publicly available human and murine RNA-sequencing data.

**Results:**

We detected widespread cryptic splicing in TDP-43 depletion datasets but almost none in another ALS/FTD-linked protein FUS. Sequence motif and iCLIP analysis of cryptic exons demonstrated that they are bound by TDP-43. Unlike the cryptic exons seen in hnRNP C depletion, those repressed by TDP-43 cannot be linked to transposable elements. Cryptic exons are poorly conserved and inclusion overwhelmingly leads to nonsense-mediated decay of the host transcript, with reduced transcript levels observed in differential expression analysis. RNA-protein interaction data on 73 different RNA-binding proteins showed that, in addition to TDP-43, 7 specifically bind TDP-43 linked cryptic exons. This suggests that TDP-43 competes with other splicing factors for binding to cryptic exons and can repress cryptic exon inclusion.

**Conclusions:**

Our quantitative analysis pipeline confirms the presence of cryptic exons during the depletion of TDP-43 but not FUS providing new insight into to RNA-processing dysfunction as a cause or consequence in ALS/FTD.

**Electronic supplementary material:**

The online version of this article (doi:10.1186/s12920-017-0274-1) contains supplementary material, which is available to authorized users.

## Background

Splicing depends on the reliable recognition and subsequent removal of non-coding intronic sequence by the multi-protein spliceosome complex. Boundaries that demarcate exons from introns are first recognised by the binding of the U1 small nuclear RNA (snRNA) to the 5′ splice site at the beginning of the intron and the U2 snRNA binding to the polypyrimidine tract and 3′ splice site at the end of the intron. The two snRNAs then interact with each other and the intronic sequence is removed via a series of transesterification reactions [1]. During splicing, regulatory sequences in the nascent RNAs recruit RNA-binding proteins (RBPs) to shape mRNAs, selecting or omitting particular exons by acting to enhance or repress splicing.

Due to the long length and reduced evolutionary conservation of intronic sequences, pairs of 3′ and 5′ splice sites can emerge randomly to create potentially new exons. These cryptic exons (also known as pseudoexons) arise due to mutations that create new splice sites or remove the existing binding sites for splicing repressors. These type of mutations have also been implicated in a number of genetic diseases [2–5]. Inclusion of a cryptic exon, untested by evolution, can destabilise the transcript or radically alter the eventual protein structure. The former can occur by the nonsense-mediated decay (NMD) pathway, which occurs when a transcript has a premature termination codon introduced either directly by the cryptic exon or by a subsequent shift of reading frame [6, 7].

Cryptic exons can also emerge from transposable elements. One such example are Alu elements, the predominant transposable element in primates which are often found within introns in the antisense direction [8]. The consensus Alu sequence consists of two arms joined by an adenine-rich linker ending with a poly-adenine tail. When transcribed in the antisense direction these uridine-rich sequences can act as cryptic polypyrimidine tracts and only a few mutations are required to convert them into viable exons in a process termed exonisation [9]. De novo mutations that lead to Alu exonisation have been found in a range of diseases [3, 4, 10, 11] suggesting a need for regulation of potentially damaging Alu exons. Alu exonisation is repressed by the RNA binding protein hnRNP C, which competes with the spliceosome component protein U2AF65, the partner of the U2 snRNA, for binding cryptic 3′ splice sites [12]. Due to the potentially negative effects of incorporation of new exons, aberrant recognition of cryptic exons needs to be repressed.

TDP-43 is an RNA-binding protein encoded by the *TARDBP* gene. Loss of TDP-43 from the nucleus accompanied by TDP-43 positive inclusions in the cytoplasm of cortical and spinal cord neurons is the hallmark pathology of amyotrophic lateral sclerosis (ALS) as well as the majority of cases of frontotemporal dementia (FTD) [13]. In addition, missense mutations in *TARDBP* can cause familial ALS [14]. These findings point to a central role of TDP-43 in the aetiology of ALS and FTD.

Rare ALS-causing mutations have been found in another RNA-binding protein, FUS [15] further raising the possibility that the impairment of RNA processing is a central cause of ALS. TDP-43 was first shown to repress the inclusion of exon 9 in the *CFTR* gene by binding to long UG-rich sequences [16] and subsequently shown to act as both a splicing enhancer and repressor [17–19]. TDP-43 and FUS have both been shown to bind a set of overlapping RNA targets [20]. Transcriptome-wide studies of the effects of TDP-43 depletion, overexpression or mutation have demonstrated widespread changes in gene expression and splicing [21–24]. Of particular interest are long intron containing genes, which are dramatically downregulated during both TDP-43 and FUS depletion [20, 23].

Recently, Ling and colleagues observed the inclusion of cryptic exons when TDP-43 was depleted in HeLa or mouse embryonic stem cells [25]. These cryptic exons were shown to originate from poorly evolutionarily conserved sequence and shared no positions between the two species. These findings raise the possibility that impaired exon recognition contributes to TDP-43’s role in ALS aetiology. We therefore aimed to replicate and expand the findings of Ling and colleagues. Firstly, we undertook a quantitative genome-wide analysis of cryptic exon patterns, defining objective criteria that take advantage of biological replicates when available. Secondly, we applied this computational strategy to seven datasets (four human and three murine models) to systematically quantify cryptic RNA alterations associated with depletion of TDP-43. In addition we also investigated FUS in order to determine whether modulation of cryptic splicing was a common feature of RBPs implicated in ALS. We also analysed hnRNP C, as it had been previously shown to repress cryptic exons. Lastly, we used independent protein-RNA interaction datasets, conservation data, repeat element annotation and splice site scoring to investigate the potential mechanisms linking TDP-43 depletion with the cryptic exon phenomenon.

## Methods

### Data preparation

Table 1 lists all the public data used in this study. For all RNA-seq data we first performed adapter and quality trimming (Phred score > 20) with Trim Galore (0.4.1) on the FASTQ files before aligning to either the human (hg38) or mouse (mm10) reference genome with STAR (2.4.2a) [26]. The resulting BAM file was sorted and PCR duplicate marked with NovoSort (1.03.09). Processed iCLIP peaks data was downloaded from the iCOUNT server (http://icount.biolab.si/). Both the human and mouse TDP-43 iCLIP data have been previously published [27, 28].

**Table 1:** List of accessions. For the ENCODE RNA-seq libraries, the control and target depletion samples are listed under separate accessions

Assay	Accession Code	Downloaded from	Target	Cell-Tissue	Pubmed ID
RNA-seq	PRJNA282887	ncbi.nlm.nih.gov/sra	TDP-43	Mouse ES	26250685
RNA-seq	PRJNA282692	ncbi.nlm.nih.gov/sra	TDP-43	Human Hela	26250685
RNA seq	PRJNA127211	ncbi.nlm.nih.gov/sra	TDP-43	Mouse ES	20660762
RNA-seq	PRJNA141971	ncbi.nlm.nih.gov/sra	TDP-43	Mouse adult brain	21358643
RNA-seq	ENCSR129RWD	encodeproject.com	control	K562 mRNA	NA
RNA-seq	ENCSR134JRE	encodeproject.com	TDP-43	K562 mRNA	NA
RNA-seq	ENCSR372DZW	encodeproject.com	control	K562 total RNA	NA
RNA seq	ENCSR455TNF	encodeproject.com	TDP-43	K562 total RNA	NA
RNA-seq	PRJNA174534	ncbi.nlm.nih.gov/sra	FUS	Mouse adult brain	23023293
RNA-seq	ENCSR084SCN	encodeproject.com	control	K562 mRNA	NA
RNA-seq	ENCSR32500M	encodeproject.com	FUS	K562 mRNA	NA
RNA-seq	PRJEB3048	ncbi.nlm.nih.gov/sra	hnRNP C	HeLa	23374342
iCLIP	20100222_LUjt3	icount.biolab.si	TDP-43	Mouse embryonic brain 1	22934129
iCLIP	20091102_LUjt5	icount.biolab.si	TDP-43	Mouse embryonic brain 2	22934129
iCLIP	20100222_LUjt3	icount.biolab.si	TDP-43	Human neural stem cells	21358640
iCLIP	20101125_LUjt8	icount.biolab.si	TDP-43	Human SH-SY5Y 1	21358640
iCLIP	20091102_LUjt5	icount.biolab.si	TDP-43	Human SH-SY5Y 2	21358640
eCLIP	Multiple	encodeproject.com	Multiple	HepG2/K562	NA

Processed eCLIP data (previously described by [29]) was downloaded from the ENCODE project. The narrowPeaks bed format was used with the first nucleotide of the cluster defined as the peak. Peak coordinates from iCLIP and eCLIP were converted to the hg38 and mm10 builds using the LiftOver tool from UCSC.

### Cryptic splicing definition

Splicing aware alignment software such as STAR cut short reads that originate from a spliced transcript and align the pieces separately, marking the distance between them as a splice junction. Splice junctions can be used to reaffirm known splicing patterns or infer novel splicing. We define cryptic splicing as the emergence or relative increase in splice junctions that splice from known splice sites to unannotated positions within introns. This increase correlates with the depletion of a particular RNA binding protein. Different repositories have different levels of proof for annotating exons but we define an annotated exon as one listed in the Ensembl list of transcripts (release 82).

### Cryptic splicing discovery with the CryptEx pipeline

Due to the diversity in the quality of published RNA-seq data, the cryptic splicing discovery pipeline was designed to be used on any RNA-seq library, whether single or paired end, stranded or unstranded, total RNA or polyA-selected RNA. However, when high quality RNA-seq data is available (paired end reads >100bp sequenced with >30M reads per sample), we instead suggest using one of the several recently released tools that can identify and classify unannotated splice junctions such as JunctionSeq [30], Leafcutter [31], MAJIQ [32] or SGSeq [33].

The inherent flexibility of our pipeline results in a large number of false positive hits which have to be aggressively filtered downstream. The initial cryptic exon discovery pipeline was written in Bash, using SAMTools (version 1.2) [34] and BEDTools (version 2.25.0) [35]. The statistical testing for differential cryptic exon usage was carried out using the well-known DEXSeq framework [36]. All code for downstream processing and filtering of cryptic hits was written in the R language (version 3.1.1) using the Biostrings, data.table, DEXSeq, dplyr, GenomicRanges, ggplot2, gridExtra, optparse, plyr, stringr, and tidyr packages. All the code for reproducing this paper is available in a GitHub repository [37].

In order to discover all possible splice junctions that travel into the intron we first extracted all spliced reads from each aligned bam file using SAMTools, filtering out any secondary alignments. To extract only the spliced reads that overlap an annotated exon we then performed an intersection in BEDTools with a flattened list of exons, created using the dexseq prepare annotation.py Python script included with the DEXSeq package [36]. An inverse intersection was then performed with the same exon list to retain only the spliced reads which do not bridge two annotated exons. The intronic mapping sections of each read were split off from the rest and retained. In each dataset all intronic mapping spliced reads from each sample were grouped together irrespective of condition. Split reads that were within 500bp of each other were merged into larger intervals, hereby referred to as tags. This ideally captures both the upstream and downstream splice junction to a central cryptic cassette exon. To keep only the tags that are splicing within the gene body another intersection was performed with a list of introns. This was generated from the same flattened exon file by an R script written by Devon Ryan [38]. The tags were then incorporated into the flattened list of exons. The reads that overlap annotated exons and tags were counted using HTSeq [39] on the default settings, ignoring PCR duplicate reads. The read counts were used to calculate differential usage of each exon with DEXSeq.

All the cryptic tags with an adjusted *P*-value (false discovery rate) < 5% and a | log_2_(fold change)| > 0.6 were extracted from the DEXSeq results table (see Additional file 1: Figure S1). The splice junctions from the alignment of each sample were used to work out the coordinates of the canonical junction that spans the intron within which the cryptic tag is or isn’t spliced in control samples. Using splice junctions from the depletion condition samples, the upstream and downstream junctions that connect the adjacent annotated exons to the cryptic tag were re-discovered and quantified. Any cryptic example that did not have at least one upstream or downstream junction per sample or had fewer than ten canonical splice junctions was removed. These junctions were used to calculate per-condition mean Percent Spliced In (PSI) values which are a ratio of cryptic splicing over the sum of cryptic and canonical splicing [40]. As a number of cryptic splicing events are present at a low level in control samples, ∆PSI values were created for both upstream and downstream splicing for each tag. This is the difference in PSI between the depletion samples and the control samples. Any cryptic tag that had either an upstream or downstream ∆PSI < 5% was removed.

### iCLIP/eCLIP enrichment

The coordinates of each cryptic tag were flanked by 100 base pairs on either side to capture binding around the putative splice sites. In order to compare the overlap between cryptic exons and RNA-protein binding peaks, two sets of null exons were created for comparison, which maintain the same length as their corresponding cryptic exon but sample either the intronic sequence outside of the flanked exon or that of the adjacent introns within the same gene if available. Overlaps between exons and iCLIP and eCLIP peaks were calculated using BedTools.

### Motif enrichment analysis

FASTA sequence was generated for the cryptic exons flanked by 100 nucleotides either side and submitted to the MEME web tool [41] under the default settings. The analysis was repeated using the HOMER algorithm [42] on RNA mode. Motifs were created using WebLogo [43]. Frequencies of the 16 possible dinucleotides were compared between flanked cryptic exon sequences with adjacent intron sequences from the same gene.

### Transposable element enrichment

Lists of transposable elements in human and mouse (hg38 and mm10 respectively) were previously generated by the RepeatMasker tool [44] and were downloaded from UCSC. Overlap between different transposable elements and the cryptic exons was calculated in each orientation using BedTools.

### Conservation analysis

PhyloP compares the sequence alignments of multiple species to produce per base conservation scores [45]. Average conservation score per cryptic tag was calculated using bigWigSummary (UCSC) for both human and mouse data. The lists of splice junctions created by STAR when aligning each sample were used to identify the coordinates of the exons adjacent to the cryptic exon. The randomly sampled intronic sequence from the cryptic-containing intron was used as a negative control.

### Differential expression

For each sample the number of reads overlapping each gene were counted using HTSeq using the same Ensembl gene models as before. Differentially expressed genes were identified using DESeq2 [46] at a false discovery rate of 10%.

### Protein prediction analysis

Any cryptic exon which did not fall within the coding sequence of a transcript was omitted. Splice junctions generated by STAR were used to determine the upstream and downstream exons adjacent to each cryptic exon. Only cryptic exons that had both upstream and downstream splice junctions (cassette exons) were kept. The upstream and downstream exons were matched to their corresponding annotated exon in the Ensembl transcript file for each species to assign the correct reading frame for translation. Nucleotide sequences for transcripts either including or excluding the central cryptic exon were created and translated in silico using the R Biostrings package. Premature termination codons (PTCs) were identified from the translated sequence and defined as escaping nonsense mediated decay (NMD) if they occurred within 50 nucleotides of the downstream exon-exon junction. Frameshifts were identified by comparing the sequence of the downstream exon with and without the central cryptic exon. A null distribution of PTC-containing or frame shifted transcripts was created by generating central exons from random nucleotide sequence, keeping the length distribution the same. For each species this was repeated 100 times.

### Splice junction scoring

The strength of 5′ and 3′ splice sites was calculated for the human cryptic exons using maxEnt [47]. Higher scores indicate the increased log odds of a given splice site being a true splice site. The 5′ splice site is defined as the last 3 nucleotides of the upstream exon flanked by 6 intronic nucleotides, of which the first two are invariably GU. The 3′ splice site is defined as the last 20 intronic nucleotides of which the final two are invariably AG, flanked by the first 3 nucleotides of the downstream exon. The splice sites of annotated exons were used as a positive control. Randomly generated sequence with invariant AG or GT was used as a negative control. Paired t-tests were carried out to test the direction of change between the cryptic and annotated splice sites for each class of cryptic exon.

## Results

### Depletion of TDP-43 but not FUS results in cryptic exons

We compared the involvement of two ALS-linked RNA-binding proteins in cryptic splicing: TDP-43 and FUS. We analyzed publicly available TDP-43 depletion RNA-Seq datasets (three human, three murine, datasets 1–6 in Table 2), FUS depletion RNA-Seq datasets (1 human, 1 murine, datasets 7–8 in Table 2) and as a positive control a human hnRNP C depletion dataset for which cryptic exons have previously been reported (dataset 9 in Table 2). While these datasets differ in library preparation method, read depth and length, and protein depletion method (Table 2), the FUS datasets match the TDP-43 datasets in cell type, depletion method sequencing depth and lab of origin (Table 2), as do the TDP-43 and FUS depletion data created by the ENCODE consortium. Using these datasets, we took a genome-wide approach to describe the pattern of unannotated splicing in human and mouse that we call CryptEx. The first step involves identifying novel splice junctions between annotated exons and unannotated intronic regions. These reads were clustered to create putative cryptic exons. Making use of biological replicate samples we compared the abundance of reads covering the cryptic region relative to the rest of the annotated exons of the surrounding gene. Differential usage of each cryptic region was tested between depletion and control samples. Results were omitted if they fell outside of a strict 5% false discovery rate.

**Table 2:** All RNA-sequencing data used in this study

	Species	Cell type	Protein	Depletion	Library type	Read type	Depth	Citation
1	Human	HeLa	TDP-43	siRNA	mRNA	l00bp PE	97-116M	Ling, 2015 [25]
2	Mouse	ES	TDP-43	Knockout	mRNA	l00bp PE	70-75M	Ling, 2015 [25]
3	Human	K562	TDP-43	shRNA	Total RNA	l00bp PE	55-62M	ENCODE
4	Human	K562	TDP-43	shRNA	mRNA	l00bp PE	25-29M	ENCODE
5	Mouse	Adult brain	TDP-43	ASO	mRNA	75bp SE	35-60M	Polymenidou, 2011 [23]
6	Mouse	ES	TDP-43	Knockout	mRNA	40bp SE	2-11M	Chiang, 2010 [21]
7	Human	K562	FUS	shRNA	mRNA	l00bp SE	12-21M	ENCODE
8	Mouse	Adult brain	FUS	ASO	mRNA	72bp SE	20-60M	Lagier-Tourenne, 2012 [20]
9	Human	HeLa	hnRNP C	siRNA	mRNA	72bp SE	26-28M	Zarnack, 2013 [12]

For single end sequencing, depth is measured in millions of mapped reads whereas paired end sequencing depth is measured in millions of mapped fragments

*ES* leukaemia cell line. *siRNA* small interfering RNA, *shRNA* short hairpin RNA, *ASO* antisense oligonucleotide, *PE* paired end sequencing, *SE* single end sequencing

**Fig. 1:**
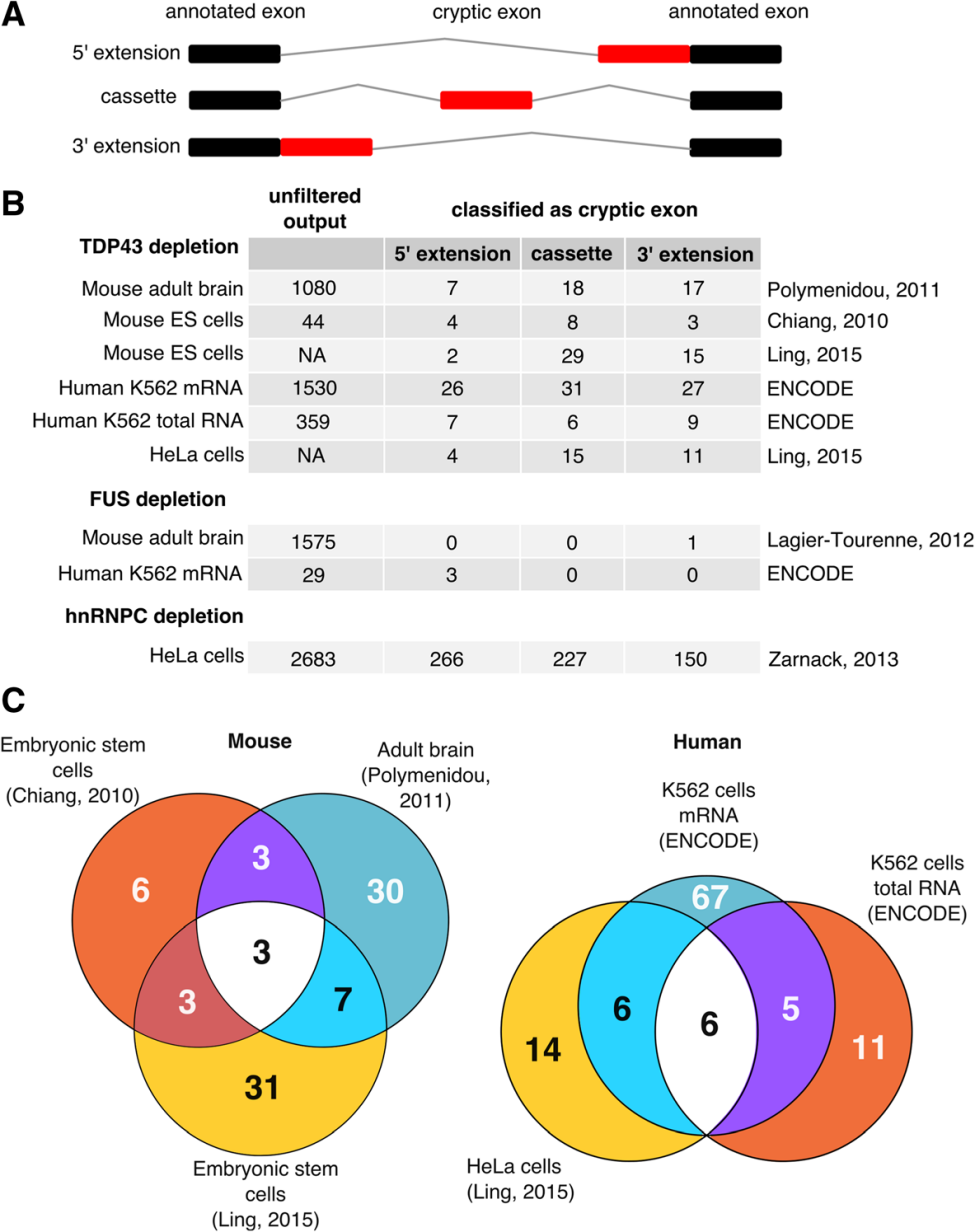
Cryptic splicing discovered by the CryptEx pipeline. **a** Schematic of the three classes of cryptic exon. *Black boxes* represent annotated exons and *red boxes* represent a cryptic exon. *Grey lines* represent the spliced intron. **b** Tally of the three classes of cryptic exon discovered by the CryptEx pipeline across the nine datasets. “Unfiltered output” refers to the number of differentially used cryptic splicing events at a false discovery rate (FDR) < 5% before undergoing cryptic exon classification. Counts from Ling et al’s data are taken from the paper itself. **c** Venn diagrams showing the overlap between the six TDP-43 depletion datasets

In order to further refine our cryptic exon detection algorithm, we then measured the amount of splicing to and from each region and used this information to filter and classify cryptic splicing events into three categories of cryptic exon: (i) cassette-like, where novel 3′ and 5′ splice sites are recognised, which forms a completely new exon; (ii) 5′ extension, where a novel 3′ splice site is recognised and an existing exon is extended upstream of its annotated start and (iii) 3′ extension, where a novel 5′ splice site is recognised and an exon is extended downstream of its annotated end (Fig. 1a). Note that our approach does not consider fully retained introns, but several other methods have been designed for this purpose [48–51].

Figure 1b lists the counts of both pre-classification cryptic regions (“unfiltered output”) and the post-classification cryptic exons. Comparing the two human ENCODE K562 cell line TDP-43 depletion datasets (3-4), the poly-A selected mRNA-Seq dataset yielded far more splicing events than the total RNA dataset, presumably due to polyA selection leading to a higher coverage of mature spliced mRNA species. In total 95 human cryptic exons were discovered and classified, with the majority only detected in the mRNA-seq dataset. 11 cryptic splicing events were shared between datasets 3 and 4 (Fig. 1c). Of the 26 human cryptic exons reported by Ling, 12 were seen in at least one of the two datasets 3 and 4.

Both mouse datasets differ in both cell type (adult striatum in dataset 5 vs embryonic stem (ES) cell in dataset 6) and read depth (35-60M in dataset 5 vs 2-10M in dataset 6). 52 cryptic exons were identified in total, with 46 detected in the adult striatum and 15 in ES cells, with 6 exons observed in both. Of the 46 cryptic splicing events identified in murine samples by Ling et al, 13 were detected in at least one of datasets 5 and 6. Side by side visual inspection (Additional file 2: Figure S2 and Additional file 3: Figure S3) suggests that differences in library preparation and read depth are behind the low concordance rates in both human and mouse, as cryptic exons detected in the higher depth dataset (K562 mRNA and mouse adult brain) can be observed by eye in the lower depth dataset (K562 total RNA and mouse ES cell). These exons currently fail to be detected by the CryptEx algorithm.

No cryptic splicing events were shared between human and mouse as previously reported [25]. Note that to report overlap with Ling and colleagues (datasets 1 and 2), the raw data was unsuitable for our cryptic exon discovery pipeline due to a lack of biological replicate samples. Instead the sequence data was aligned and the splice junctions generated by the aligner were used to classify previously reported cryptic exons.

In contrast, while a large number of novel splicing events were observed in the FUS depletion datasets, our algorithm only classified 3 in mouse and 1 in human as cryptic exons. FUS depletion was not observed to produce any cassette-like cryptic exons in either species. Additional file 1: Figure S1 visualises the full unclassified output of the pipeline and illustrates the diversity of splicing alterations that occur upon RBP depletion. Additional file 2: Figure S2 and Additional file 3: Figure S3 comprise of screenshots of every reported cryptic exon from the IGV browser in mouse and human respectively. Additional file 4: Tables S1 and S2 (ST1/ST2) list the coordinates of each cryptic exon along with the results of each experiment.

To verify that the cryptic exons were not included under normal conditions, we looked for evidence of the 52 total mouse cryptic exons in a set of 9 mouse tissues previously published in [52]. For the 95 total human cryptic exons we looked for evidence of inclusion in 49 normal human tissues collected by the GTEx project [53]. For each cryptic exon we looked for junctions that splice to and from the cryptic exon and divided by the counts of a junction that spans the length of it, to produce a ratio of cryptic exon inclusion. Only 4 mouse and 3 human cryptic exons showed any evidence of being included at all in normal tissues, albeit at low levels and with very high variability (Additional file 5: Tables S5 and S6). This suggests that the cryptic exons we discovered are generally not included in normal tissues and that they can only be seen during the depletion of TDP-43.

### Cryptic exons are enriched in TDP-43 binding motifs and iCLIP peaks

For the remainder of our study, cryptic exons were grouped into unions of all cassette-like exons and extension events discovered in human and mouse, totalling 95 human and 52 murine cryptic exons. We then explored whether TDP-43 binding could explain the observed splicing changes in RNA-Seq data, as observed by Ling and colleagues. We took two complementary and genome-wide approaches: (i) searching for enriched motifs in the RNA sequence including and surrounding the cryptic exons and (ii) correlating the positions of cryptic exons with TDP-43 protein-RNA interaction data.

**Fig. 2:**
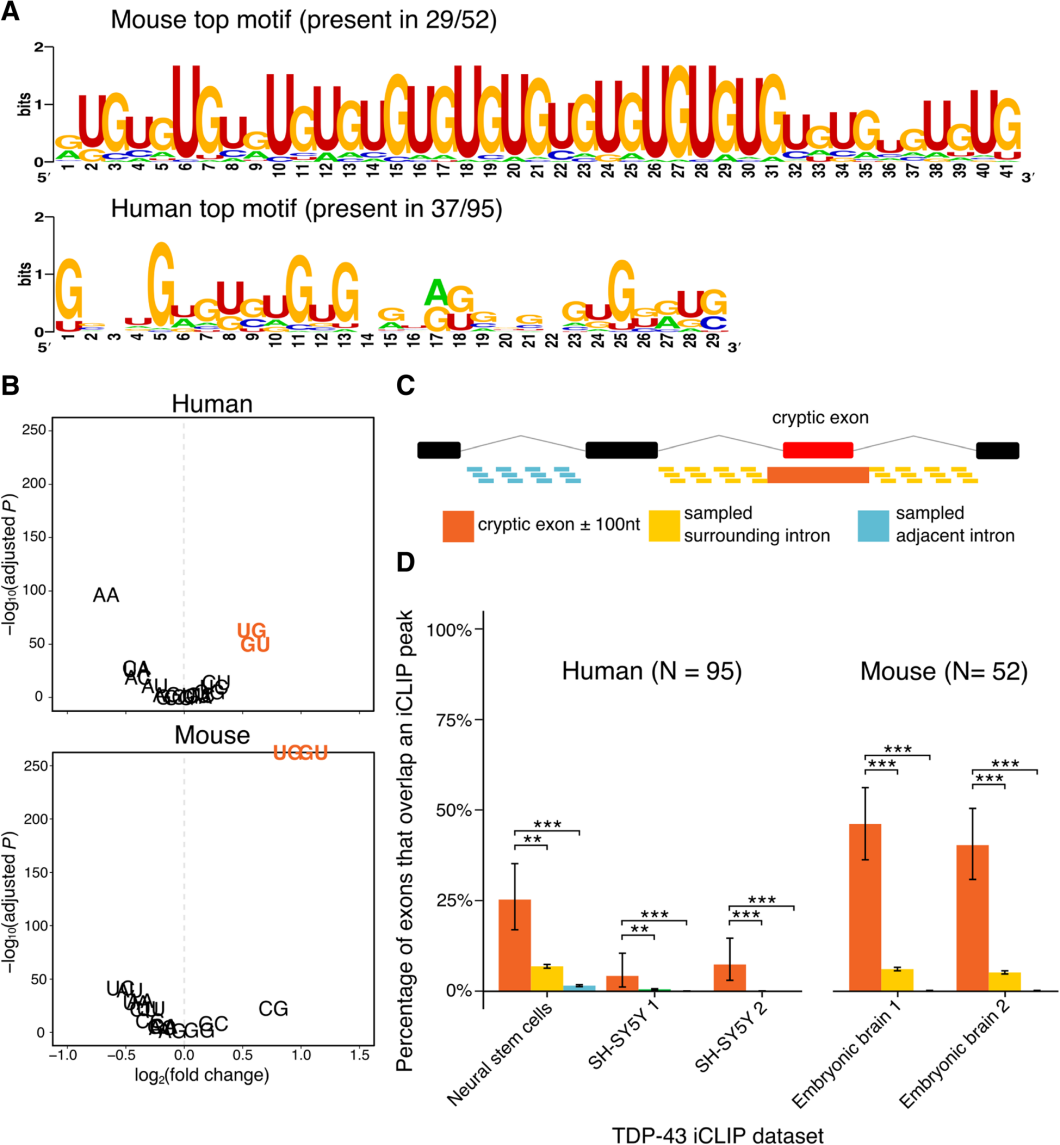
Evidence of TDP-43 binding cryptic exons. **a** Results of MEME motif search. Only the motif with the greatest enrichment compared to background sequence is presented. **b** Dinucleotide enrichment in the flanked cryptic exons compared to adjacent introns. **c** Schematic of iCLIP peak enrichment test. For illustration a cassette-like cryptic exon (*green box*) is shown between two annotated exons (*black boxes*) separated by intronic sequence (*black lines*). The proportion of the group of cryptic exons flanked either side by 100 nucleotides (*orange*) that overlap at least one iCLIP peak is compared to the proportion of overlaps in a group of length matched sequences from either the surrounding intron (*yellow*) or an adjacent intron (*blue*) each randomly sampled 100 times per gene. **d** iCLIP peak overlap enrichment for the 95 human cryptic exons found in either K562 cell TDP-43 depletion dataset and the 52 cryptic exons found in either mouse TDP-43 depletion dataset. Error bars denote 95% confidence intervals of the binomial distribution. ^∗^ *P* < 0.05, ^∗∗^ *P* < 0.001, ^∗∗∗^ *P* < 10^−16^. All *P*-values adjusted for multiple testing by Bonferroni method

TDP-43 can repress or enhance the inclusion of a given exon by either binding within or adjacent to the exonic sequence [27]. Hence for our motif search, we flanked cryptic exon sequences by 100 nucleotides on either side. UG-rich motifs were found to be enriched in both mouse and human cryptic exons using two different algorithms: MEME (Fig. 2a) and HOMER (Additional file 6: Figure S4). Of the 52 mouse cryptic exons 29 had a run of UG up to 40 nucleotides in length. Similarly, human cryptic exons were enriched in a UG motif but not in a continuous manner. By comparing the frequencies of 16 possible dinucleotides between the flanked cryptic exon sequence and the sequence of the adjacent intron either up or downstream of the cryptic-containing intron we were able to resolve the enrichment of UG dinucleotides (Fig. 2b). UG and GU were enriched in flanked cryptic exon sequence in both human (fold change GU = 1.53; UG = 1.48; *P* < 10^−50^; proportion test) and mouse (fold change GU = 2.14; UG = 1.85; *P* < 10^−50^; proportion test).

Individual nucleotide resolution UV crosslinking followed by immunoprecipitation (iCLIP) allows for precise nucleotide-resolution observation of which RNA species interact with a particular RNA-binding protein [12, 54]. We downloaded all publicly available TDP-43 iCLIP data from the iCount repository (http://icount.biolab.si/). We then intersected each set of iCLIP peaks with our cryptic exons, surrounded by 100 base pairs of flanking sequence either side. The proportion of overlapping cryptic exons was compared to the proportion of overlap between iCLIP peaks and two classes of null exon; the first created from the surrounding intronic sequence outside of each flanked cryptic and the second from an adjacent intron (Fig. 2c). If TDP-43 binding was uniform throughout an intron or gene then we would expect to see similar proportions of overlap in each. However, both species show an enrichment in TDP-43 binding peaks specific to the cryptic exons in every iCLIP dataset used, with as much as 25% of human cryptic exons and 50% of mouse cryptic exons overlapping at least one iCLIP peak each (Fig. 2d; both species *P* < 10^−16^; proportion test).

### Cryptic exons are marginally enriched in transposable elements

Motivated by previous findings of cryptic exons associated with hnRNP C depletion and caused by exonisation of antisense Alu elements [12, 55], we investigated whether TDP-43 induced cryptic exons preferentially overlap specific families of transposable elements and/or class of repetitive sequences. Transposable/repeat element annotations were obtained using the RepeatMasker software and these features were split by family and orientation. Although Alu elements are a subfamily within the primate SINE element family, we included them separately given the prior hnRNP C result. As transposable elements are abundant in the genome, the number of overlapping exons was compared as before to the overlap in the whole intron and adjacent introns.

**Fig. 3:**
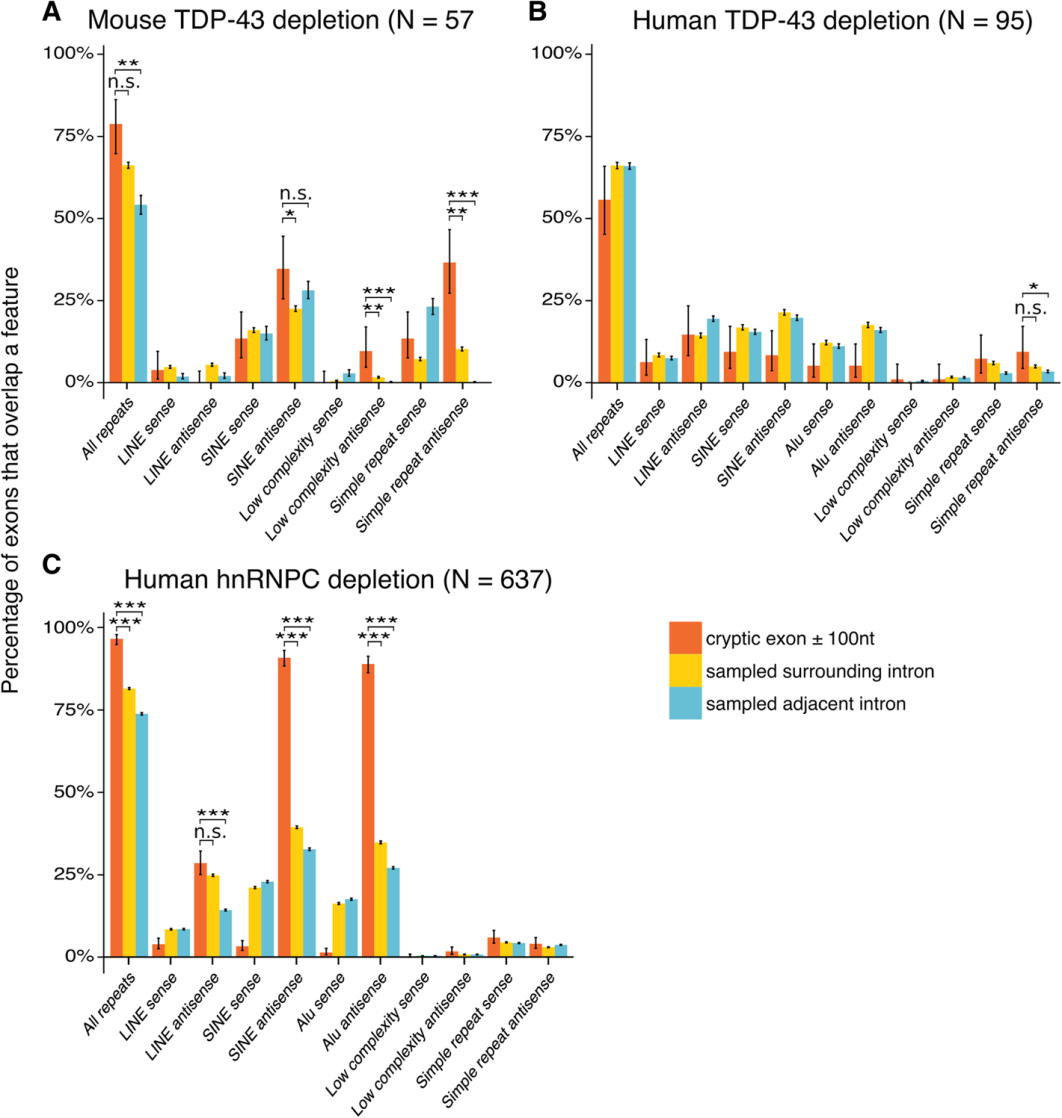
Cryptic exons in transposable elements Overlap between different families of repetitive element with lists of exons, separated by orientation. **a** Mouse TDP-43 depletion. **b** Human TDP-43 depletion. **c** Human hnRNP C depletion. The proportion of the cryptic exons that contain a particular element are shown in *orange*. Length - matched random samples from the surrounding intron (*yellow*) and adjacent introns (*blue*) are used as controls. LINE: Long Interspersed Nuclear Element; SINE: Short Interspersed Nuclear Element. ^∗^ : *P* < 0.05, ^∗∗^ : *P* < 0.001, ^∗∗∗^ : *P* < 10^−16^ . All *P*-values corrected for multiple testing with Bonferroni method

The mouse cryptic exons show a modest enrichment in antisense SINE elements in and around the cryptic exons when compared to the rest of the surrounding intron. However the elements are also common in adjacent introns and so are not enriched for this comparison (Fig. 3a). The antisense SINE elements are mainly from the B2 family (Additional file 7: Table S3) which in the antisense direction is rich in stretches of UG dinucleotides (Additional file 7: Figure S5). Mouse cryptic exons are also enriched in repeat elements annotated as “low complexity” or “simple repeat”, which are also mostly UG dinucleotide repeats, labelled as such or as AC repeats antisense to the direction of transcription. The human cryptic exons only show a small enrichment in”simple repeat” elements (Fig. 3b), most of which contain sense UG or antisense AC repeats (Additional file 7: Table S4). This contrasts with hnRNP C depletion, which shows a striking enrichment of antisense SINE elements of which all are of the Alu type (*P* < 10^−16^; proportion test), a result consistent with previous analyses of dataset 9 [12]. Altogether, these results are in line with findings from the motif analysis, that cryptic exons are enriched in TDP-43 binding motifs but not due to the large - scale enrichment in transposable elements.

### Cryptic exons are poorly conserved and their inclusion acts to destabilise their host transcript

**Fig. 4:**
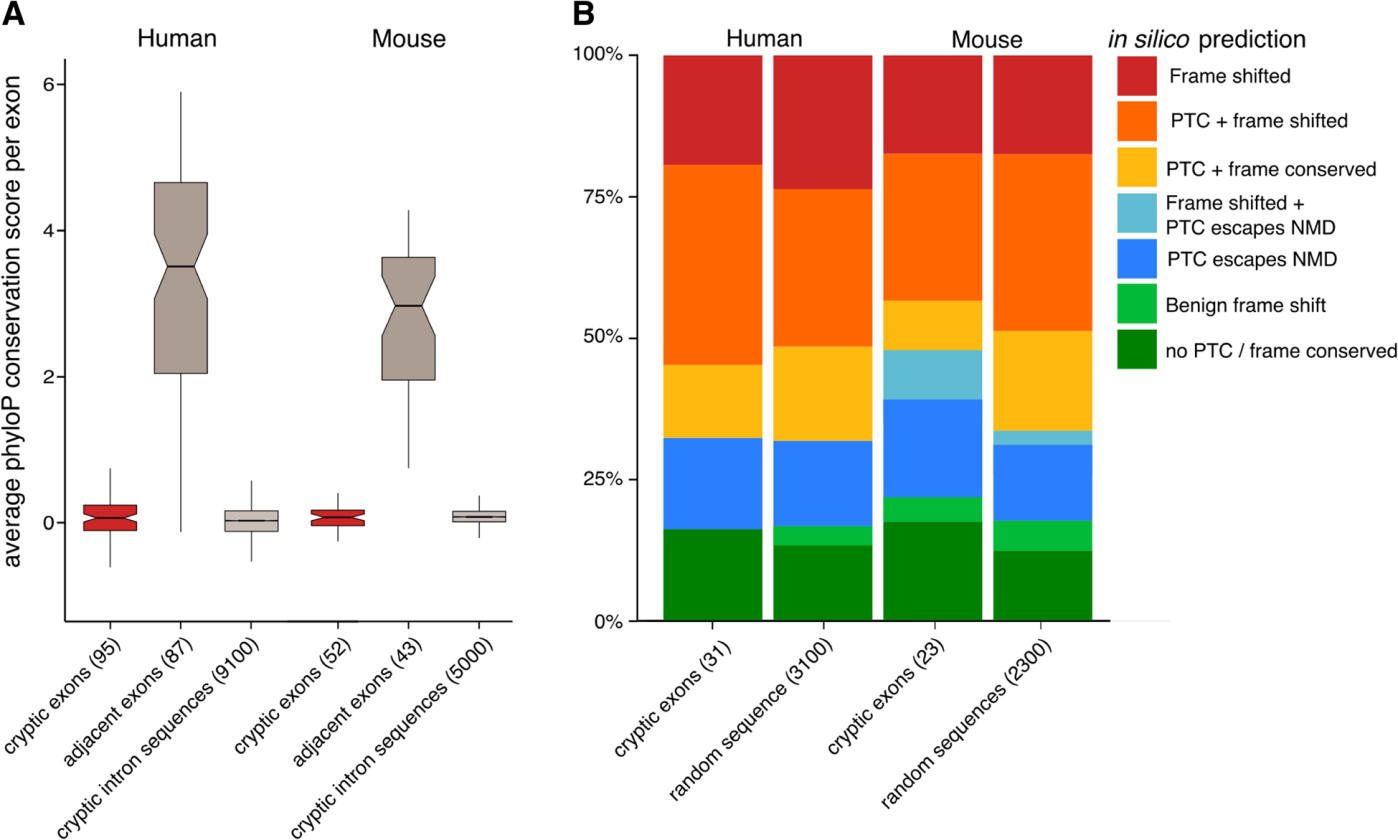
Conservation and premature termination codon analysis. **a** Per exon average PhyloP conservation scores in cryptic exons, adjacent exons within the same set of genes (when available) and randomly sampled sequences from the cryptic containing intron. Box plots show first quartile, median and third quartile with the notches representing the 95% confidence interval of the median. Whiskers represent the minimum and maximum values that fall within 1.5 times the interquartile range. **b** The functional impact of cryptic exon inclusion on the host transcripts in human and mouse. Colours indicate the category of prediction and box size indicates the proportion of the total group of exons in each category. Categories from top to bottom: Frame shifted (*red*); Frame shifted and at least one premature termination codon (PTC) included (*orange*); PTC included only (*yellow*); Frame shifted but the resulting PTC falls within 50bp of the exon-exon junction, creating a truncated protein of novel sequence (*light blue*); PTC falls within 50bp of the exon-exon junction, creating a truncated protein (*dark blue*); Frame shift without any PTCs to create a protein of novel sequence (*light green*); Cryptic exon inclusion does not frame shift nor introduces a PTC, behaving as a benign cassette exon (*dark green*). For each species there is a corresponding set of predictions calculated from random sequences with matching lengths

We then quantified the extent of evolutionary conservation of cryptic exons using the multiple species alignment conservation scores generated by PhyloP. We calculated mean conservation scores per exon for the cryptic exons and compared them to scores from both the annotated exons and randomly sampled intronic sequences from the same genes. We found no difference between cryptic exons and matched intronic sequences (Fig. 4a), and a much lower conservation level than adjacent annotated exons.

We also investigated the consequences of inclusion of cryptic exons on translation of the transcript. The inclusion of a premature termination codon (PTC) into a transcript could lead to either mRNA degradation by the nonsense-mediated decay (NMD) pathway. Alternatively, if the PTC falls within 50 nucleotides upstream of an exon-exon junction then NMD can be evaded, leading to the production of a truncated protein [7]. These two outcomes are compounded by the possibility of a cryptic exon shifting the reading frame of the downstream exon, leading either to more PTCs or the creation of a benign transcript of a different function. This gives cryptic exon inclusion six functional outcomes that can destabilise either the RNA or the downstream protein. We tested for all these outcomes by translating the predicted transcripts *in silico*. In this analysis we only considered the”cassette-like” cryptic exons that had both upstream and downstream splice junctions. Of the 31 human cryptic exons tested, 21 (67%) are predicted to destabilise their host transcript through the NMD pathway (Fig. 4b). Five cryptic exons are predicted to impart new functionality whereas five others are predicted to contain premature stop codons but evade NMD, creating truncated proteins upon translation. However, comparing these results to randomly generated sequence of the same length demonstrates that this distribution of outcomes can occur purely by chance. The 23 mouse cryptic exons tested behave similarly, with four cryptic exons creating functional proteins, one creating a new downstream protein, six leading to protein truncation and 12 being degraded by NMD (Fig. 4b). The random sequences were similarly distributed. Together this suggests that the cryptic exons are not enriched in particular sequence features and upon inclusion behave like randomly chosen sequences. This disruption of multiple mRNA species may have an effect at the protein level and explain the toxicity seen in response to TDP-43 depletion.

### Cryptic exon containing genes are downregulated

**Fig. 5:**
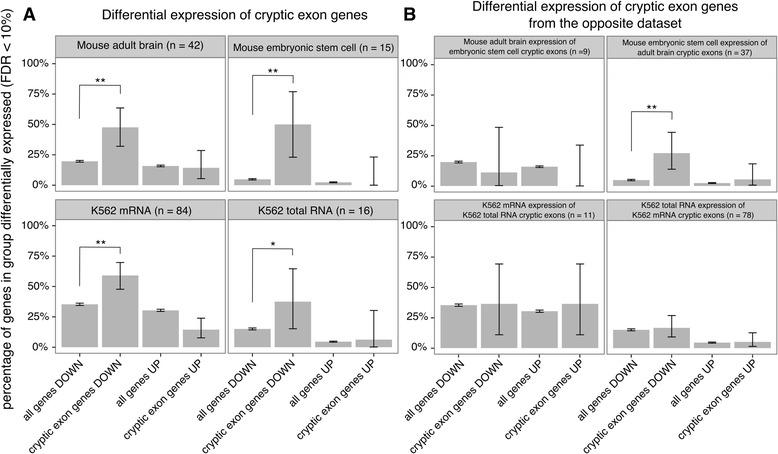
Differential expression of cryptic exon genes. **a** Significantly differentially expressed genes between TDP-43 depletion and control samples (false discovery rate = 10%) in datasets 3-6. Comparisons were made between all genes tested with an expression level at or greater than the lowest expressed cryptic exon (“all genes”) and the genes where cryptic exons were discovered (“cryptic exon genes”) with each group divided by direction of change. **b** As above but for cryptic exon genes from the other dataset of the same species. Error bars show 95% confidence intervals for each proportion. ^∗^ : *P* < 0.05, ^∗∗^ : *P* < 0.001. All *P*-values adjusted by Bonferroni correction

We then investigated, in datasets 3-6, whether genes containing cryptic exons showed a specific pattern of altered expression. We calculated the proportion of the cryptic exon containing genes in each dataset that were differentially expressed at a FDR of 10%. We compared this with the proportion of differential expression of all genes with an expression level at or greater than the lowest expressed cryptic exon found in that dataset. Figure 5a shows the number of differentially expressed genes in each dataset as a proportion of the total, separated by direction. In all four TDP-43 depletion datasets, the cryptic exon containing genes as a group are more likely to be significantly downregulated compared to the genome-wide proportion (*P* < 0.001; hypergeometric test).

Furthermore, we performed the same analysis for each dataset with the cryptic exon containing genes that were only found in the other dataset of the same species (Fig. 5b). Surprisingly, in the mouse ES cell dataset 6 there was an enrichment of downregulated genes that contain cryptic exons only detectable in the mouse adult brain dataset 5 (*P* < 0.001; hypergeometric test). Visual inspection of these 10 introns in the mouse ES cell data (Additional file 2: Figure S2) suggests that seven of them may harbour cryptic exons in the ES cell data that are currently undetectable by the CryptEx algorithm.

### Human cryptic exons are driven by the recognition of strong splice sites that are normally repressed

**Fig. 6:**
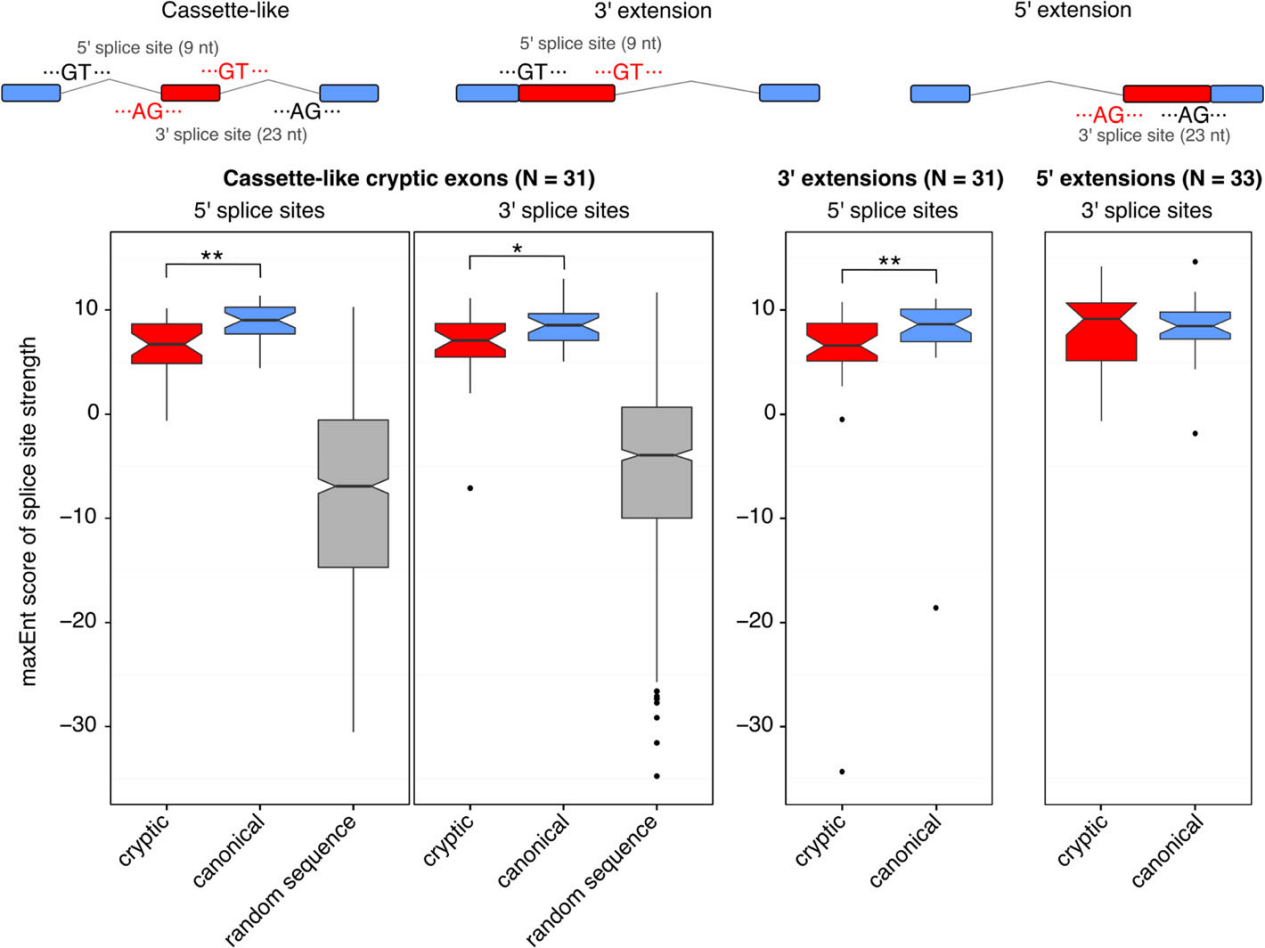
Scoring cryptic splice sites against canonical splice sites. Cassette-like cryptic exons have 5′ and 3′ splice sites that are recognised by the spliceosome under TDP-43 depletion. 5′ and 3′ splice site scores are plotted separately. Cryptic splice sites are shown in *red*, canonical splice sites are shown in *blue*. Random sequences with AG or GT consensus are plotted in grey. Cryptic extensions are the result of a cryptic splice site competing with the canonical splice site. 5′ extensions result from cryptic 3′ splice sites whereas 3′ extensions result from cryptic 5′ splice sites. *Box* plots show first quartile, median and third quartile with the notches representing the 95% confidence interval of the median. Whiskers represent the minimum and maximum values that fall within 1.5 times the interquartile range. Outliers are plotted as *black dots*. Cryptic splice sites are compared to canonical splice sites with paired t-tests. ^∗^ : *P* < 0.05, ^∗∗^ : *P* < 0.001

Whereas cassette-like cryptic exons appear as separate exons distinct from their surrounding exons, extension events must rely on a switch from a canonical splice site to a newly accessible splice site. We hypothesised that these extension events result from competition between two splice sites upon TDP-43 depletion. This would require the sequence of and around the cryptic splice site to be similarly recognisable to the spliceosome. Using the MaxEnt statistical model [47] to score splice sites by comparing their DNA sequences with constitutive observed canonical sequences, we scored the 5′ and 3′ splice sites of our cryptic exons and compared them with the scores of the surrounding canonical splice sites. The model compares splice sites from annotated exons with so-called decoy splice sites that retain the consensus AG/GT at the 3′ or 5′ splice site respectively. Therefore we also scored randomly generated sequences, which retained the consensus AG/GT positions. Figure 6 shows the scores for both the 3′ and 5′ splice sites for each class of human cryptic exons. Although the canonical splice sites were on average stronger than their corresponding cryptic splice site (*P* < 0.05; paired t-test), the majority had scores far greater than those from random sequence, suggesting that they are able function as real, albeit weaker, splice sites when TDP-43 is depleted.

### Cryptic exons are bound by other RNA - binding proteins

**Fig. 7:**
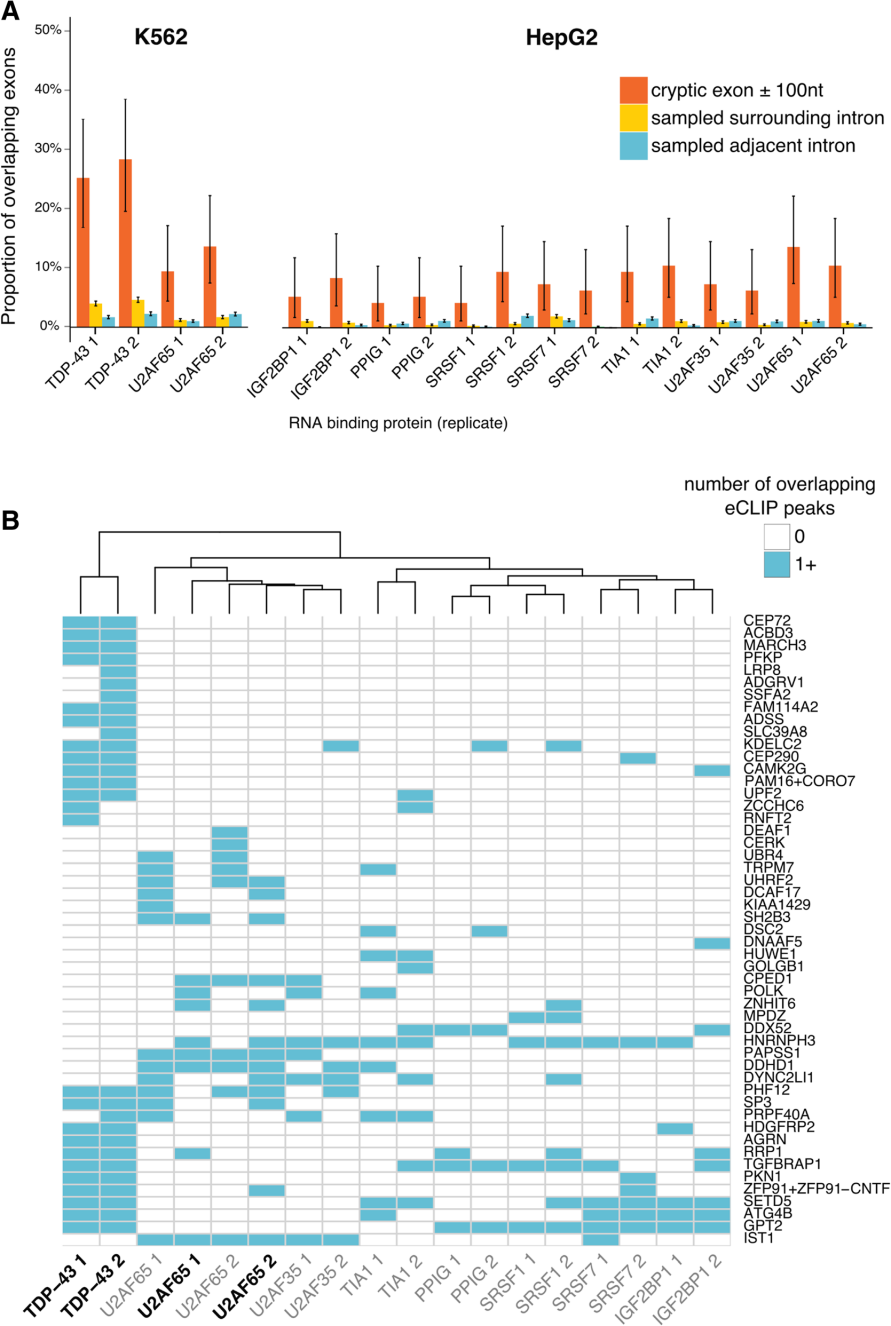
Mining of ENCODE eCLIP data in K562 and HepG2 cells. **a** RNA-binding proteins (RBPs) with significant (*P* < 0.05) proportion of overlapping cryptic exons (*red*) compared to sampled intronic sequence from the same (*green*) and adjacent introns (*blue*). **b** Comparison of eCLIP datasets from different RNA binding proteins (columns) showing the overlap with the cryptic exons (*rows*). RBPs in *bold* typeface are from K562 cells whereas those in regular typeface are from HepG2 cells

Proteomic studies have demonstrated that TDP-43 interacts with a number of RNA-binding proteins (RBPs), including multiple members of the heterologously expressed ribonucleoprotein (hnRNP) family and other splicing factors [56–58]. The splicing of specific annotated exons has been shown to depend on the interaction of TDP-43 with multiple splicing factors [59]. We hypothesised that some cryptic exons may be included indirectly through a loss of TDP-43’s interactions with different RBPs. Van Nostrand and colleagues have performed eCLIP, a higher throughput modification of the iCLIP protocol, on 73 different RBPs including TDP-43 and FUS [29]. The experiments were carried out in two human cell lines (K562 and HepG2) with 29 of the RBPs being tested in both cell lines. We performed the same overlap analysis between our human cryptic exons and each set of eCLIP peaks, using the same two sets of control sequences as before. Each eCLIP experiment was performed in duplicate. This gives each RBP four possible enrichment results using a proportion test. For each RBP, the highest *P*-value from the four tests was reported and corrected for multiple testing using a strict Bonferroni approach. Only proteins with a resulting *P* < 0.05 are reported. Unsurprisingly TDP-43 had the highest number of overlapping exons (*P* < 10^−22^; proportion test), followed by U2AF65, TIA1, SRSF7, U2AF35, PPIG, SRSF1 and IGF2BP1. Figure 7a shows the overlap between the different RBPs and the human cryptic exons. Figure 7b shows the overlap between each gene (columns) against each RBP (rows). Hierarchical clustering was performed on the RBPs. The three largest clusters consist of TDP-43 alone, the U2 snRNP binding proteins U2AF35 and U2AF65, and a third cluster containing the other proteins.

## Discussion

We have designed an analytical strategy to identify cryptic splicing that takes advantage of biological replicates in RNA sequencing data. We have applied this tool to a set of human and murine TDP-43 depletion datasets, as well as datasets that deplete hnRNP C or FUS. Our results are consistent with the previous findings that depletion of TDP-43 or hnRNP C leads to the inclusion of novel cryptic exons in both human and mouse, albeit with hnRNP C depletion leading to a far greater number of events than TDP-43. Although FUS undoubtedly plays an important role in splicing and mRNA stability and shares a number of targets with TDP-43 [20], the low number of cryptic exons observed due to FUS depletion suggests that it does not play a major role in cryptic splicing and is a key point of differentiation with TDP-43. This is despite the FUS and TDP-43 data analysed being produced under the same conditions for both mouse and human.

Further examination of TDP-43 linked exons suggests they tend to possess the necessary UG-rich sequence elements to be bound by TDP-43 and using iCLIP data we observed that a subset of the cryptic exons are bound by TDP-43 in vivo. We went on to investigate the origins of these TDP-43 bound cryptic exons, as has been done for the targets of hnRNP C. We observed that unlike hnRNP C linked cryptic exons, which invariably originate from antisense Alu elements, TDP-43 linked cryptic exons do not originate from any single family of transposable element. Furthermore their sequences show very low species conservation, akin to random intronic sequence, but remarkably they contain splice sites very close in strength to those of their adjacent annotated exons. Our differential expression analysis suggests that the bulk of cryptic exon containing genes are significantly downregulated upon TDP-43 depletion. We hypothesize that while some cassette-like cryptic exons will create functional or truncated proteins, most will lead to premature stop codons and nonsense-mediated decay of the inclusion transcript. The large number of exon extensions discovered by CryptEx may similarly lead to frameshifting and nonsense-mediated decay upon inclusion but may alternatively be sequestered in the nucleus and degraded by the mRNA surveillance pathway, as has been observed for retained introns [60]. A number of cassette-like cryptic exons could be not be spliced *in silico* to any annotated coding exons, suggesting a role for cryptic exon inclusion in to upstream open reading frames and 3′ untranslated regions, both of which have been observed to provide a site for modulation of gene expression by introducing premature termination codons [61, 62].

Consistent with this possibility, a recent proteomic study of TDP-43 depletion in human SH-SY5Y cells [63] that protein levels were changed for 3 of the 95 human cryptic exon containing genes. Two, *HUWE1* and *GOLGB1* had protein levels that were 8 and 31% of the control cells respectively whereas the third, *HNRNPH3* was found to be 7-fold increased under TDP-43 depletion. Interestingly, the cryptic exon discovered in *HNRNPH3* falls upstream of the start codon whereas those found in *HUWE1* and *GOLGB1* are predicted to trigger NMD by inclusion into the coding sequence (see Additional file 4: Table S1). Another gene with cryptic splicing seen in both K562 datasets and in the initial Ling HeLa data, *AGRN*, was recently shown to be decreased at the protein level in the cerebrospinal fluid of ALS patients compared to healthy controls and other neurological diseases [64]. Correct splicing of *AGRN* has shown to be crucial for the formation of the neuromuscular junction [65].

Our current understanding of TDP-43’s role in cryptic splicing is that of a safeguard against the inclusion of potentially damaging intronic sequence into transcripts. However, the relationship between the UG-rich sequences and the strong 5′ and 3′ splice sites and their changes over evolutionary time are unknown as we observed no conserved cryptic exons between human and mouse.

Using publicly available ENCODE eCLIP data, we identified a number of RNA binding proteins that also bind subsets of human cryptic exons under normal conditions, that is, in the presence of TDP-43. It is unsurprising that the splicing factors U2AF35 and U2AF65 are enriched as they preferably bind pyrimidine-rich 3′ splice site sequences, which all cryptic exons appear to possess. That only 10–15% of cryptic exons show U2AF35/65 binding may be due to competition from TDP-43 in a manner similar to that seen between hnRNP C and U2AF65. TIA-1 is an exciting finding due to its role in the formation of stress granules, key regulators of RNA stability [66]. In addition, IGF2BP1 has been reported as binding to TDP-43 in HEK293T and HeLa cell extracts [57, 58], whereas SRSF7 was reported as binding to TDP-43 in mouse N2A cells [56]. None of the observed proteins have been reported to change their protein level in response to TDP-43 depletion [63].

As the majority of cryptic exons are predicted to lead to nonsense-mediated decay of the inclusion transcript it seems peculiar that we can observe these transcripts at all. We hypothesise that cryptic splicing may be much more widespread than can be observed by RNA sequencing due to the highly efficient nature of nonsense- mediated decay. Over half of all cryptic exon genes are significantly downregulated in each dataset (Fig. 2b), suggesting that cryptic exon inclusion may be a key mechanism in the widespread changes in RNA expression that occur upon TDP-43 depletion. An alternative hypothesis is there is an interaction between TDP-43 and hnRNPC with the NMD machinery, which reduces the efficiency of degradation that is not shared by FUS. Experiments depleting both hnRNP C and the NMD component UPF1 have increased the number of hnRNP C-associated cryptic exons even further [67], suggesting that these experiment should be repeated for TDP-43 and FUS to better understand the relationship between cryptic splicing and mRNA degradation. Differences between the number of cryptic exons between TDP-43 and hnRNP C depletion may also relate to this, as well as differences in strength of depletion. Both TDP-43 and FUS proteins are known to bind their own mRNAs [68, 69] and so may compensate protein levels in response to shRNA knockdown.

Two genes, *ATG4B* and *GPSM2*, have previously been demonstrated to have cryptic exon inclusion RNA transcripts in ALS patient brain samples, suggesting a role for cryptic splicing in disease [25]. Our analysis also identified a cryptic exon in *ATG4B* in human cells, but not *GPSM2*; however we did not analyse human brain data. By expanding the list of cryptic exons, it will be interesting to explore whether these are also dysregulated in ALS patient brains. However, such analysis may prove challenging owing to the likely small concentrations of RNA originating from diseased cells in brain homogenate and the likelihood of degradation by NMD. Alternate strategies may involve mass spectrometry screens for the subset of cryptic exon containing genes that escape the NMD process and are translated into functional or truncated proteins. Such proteins may represent useful biomarkers for TDP-43 mislocalization and therefore ALS pathology.

A recent paper has used a complementary bioinformatic method to increase the number of cryptic exons seen in the Ling data [70]. This study extends the cryptic splicing phenomenon to RBM17, another RNA-binding protein. This finding makes the very low number of cryptic exons observed in the FUS depletion datasets even more surprising,

## Conclusions

We confirm the presence of cryptic exons after TDP-43 depletion and show they have a negative impact on the genes they reside in, leading to decreased expression levels. Further work is warranted to determine the relevance of cryptic exons to ALS and FTD pathogenesis.

## Additional files

Additional file 1: Figure S1. Pre-classification output of CryptEx pipeline demonstrates a variety of novel splicing events in TDP-43, FUS and hnRNP C depletion data. Every novel splicing event plotted by mean depth of reads covering the novel event against log_2_(fold change) between depletion and control samples. The cryptic exon classifier throws out any splicing event where the canonical intron in which the cryptic splicing event appears is represented by less than five spliced reads (purple) or where the |log_2_(fold change)| < 0.6 (light blue). Splicing events are classified as cryptic exons if the spliced reads agree with the rest of the reads and have at least 1 spliced read per sample (red). Those that fail this step are coloured orange. (ZIP 1386 kb)

Additional file 2: Figure S2. Each cryptic exon discovered in either of the two mouse datasets. For each dataset, the biological replicates were combined to create merged BAM files for each condition. The BAM files were loaded into IGV for visualisation. Read coverage and splice junctions are shown. The Ensembl transcripts for the mm10 build are provided, as are the coordinates of the cryptic exons discovered by CryptEx. (PDF 2436 kb)

Additional file 3: Figure S3. Each cryptic exon discovered in either of the two human K562 datasets. As above, with the hg38 human genome build. (PDF 5917 kb)

Additional file 4: Tables S1 and S2. Quantification of all results for each cryptic exon discovered by CryptEx in mouse (ST1) and human (ST2). Change in percent spliced in (∆PSI) scores were calculated by taking the difference of ratios of novel splice junctions over the sum of novel and annotated (intron-spanning) splice junctions in control and depletion samples for the 5′ and 3′ splice junctions. (ZIP 8 kb)

Additional file 5: Tables S5 and S6. Inclusion of cryptic exons in normal mouse (ST5) and human (ST6) tissues. RNA-seq data from multiple mouse tissues from [52] and multiple human tissues from the GTEx project [53] was downloaded from the Sequence Read Archive (accession PRJNA177791) and dbGap (phs000424.v6.p1) respectively. All samples were aligned with STAR using two-pass mapping. The resulting junction coordinates were grouped into overlapping clusters for each tissue using Leafcutter [31]. A custom R script then queried the resulting junction tables for evidence of junctions belonging to the previously discovered mouse and human cryptic exons, as well as the canonical junctions in which the cryptic exons splice to and from. The counts of upstream and downstream junctions in each sample were divided by the counts of the canonical junction to produce an inclusion ratio. For each cryptic exon that was detected, the mean inclusion ratio for each tissue is presented with the standard deviation. For the 52 mouse cryptic exons, only 4 showed any evidence of being included in normal tissues. *Reep3* E001i1 and *Adipor2* E010i2 are included in a few tissues at very low levels. *Elmod1* E003i2 is seen in brain tissue at an inclusion rate above 10%, whereas *Thoc7* E007i1 was included in every tissue bar heart. However, the inclusion rates were highly variable. Among the 95 human cryptic exons, only three were detected in 49 different human tissues from the GTEx project. *MPDZ* E015i1, *UHRF2* E017i2 and *DEAF1* E005i2 were all detected in at least 1 tissue. Only UHRF2 E017i2 was regularly seen above 10% inclusion, albeit with very high variance between samples. (PDF 66 kb)

Additional file 6: Figure S4. Motif finding with HOMER The top ten motifs reported by the algorithm when comparing flanked cryptic exons with adjacent intronic sequence. The red asterisk indicates that the motif is potentially a false positive result. (TIF 1799 kb)

Additional file 7: Tables S3 and S4. All repeat elements enriched in mouse (ST3) and human (ST4) cryptic exons. For each class of repeat element that was enriched in a set of cryptic exons, the exact overlapping repeat element was compiled into a table. The strand column refers to the orientation of the gene, not the repeat element. For the simple repeats, all annotations are made in the positive direction. Therefore (AC)n containing repeats in the antisense direction are in fact (GT)n repeats for genes on the negative strand. **Figure S5.** The RNA sequence of the B2 SINE in the antisense orientation. The DNA sequence of the B2 SINE was downloaded from lncrnadb [71] and converted to the reverse complement to get its antisense orientation. Ts were changed to Us to reflect the RNA sequence. Stretches of UG and GU are highlighted in red. (PDF 123 kb)
