# Supplementary figures and images for: Quantitative analysis of cryptic splicing associated with TDP-43 depletion
Source: BMC Med Genomics. 2017 May 26;10:38. doi: 10.1186/s12920-017-0274-1 (PMC5446763; doi:10.1186/s12920-017-0274-1)

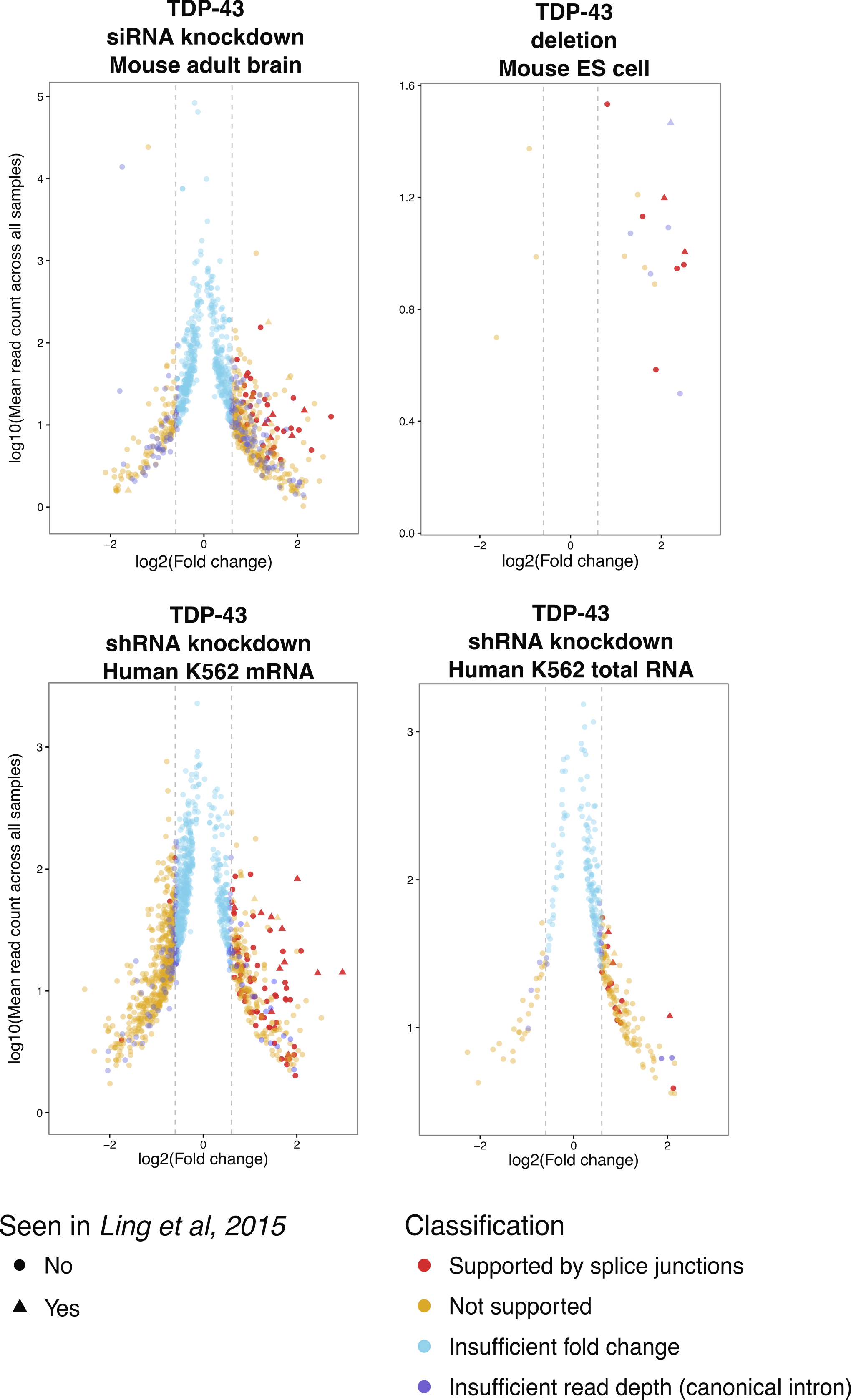

Supplement: Supplementary file 1 — Pre-classification output of CryptEx pipeline demonstrates a variety of novel splicing events in TDP-43, FUS and hnRNP C depletion data. Every novel splicing event plotted by mean depth of reads covering the novel event against log2(fold change) between depletion and control samples. The cryptic exon classifier throws out any splicing event where the canonical intron in which the cryptic splicing event appears is represented by less than five spliced reads (purple) or where the |log2(fold change)| < 0.6 (light blue). Splicing events are classified as cryptic exons if the spliced reads agree with the rest of the reads and have at least 1 spliced read per sample (red). Those that fail this step are coloured orange. (ZIP 1386 kb) [file 12920_2017_274_MOESM1_ESM.zip › Figure_S1_volcano_partR2_1.tif]

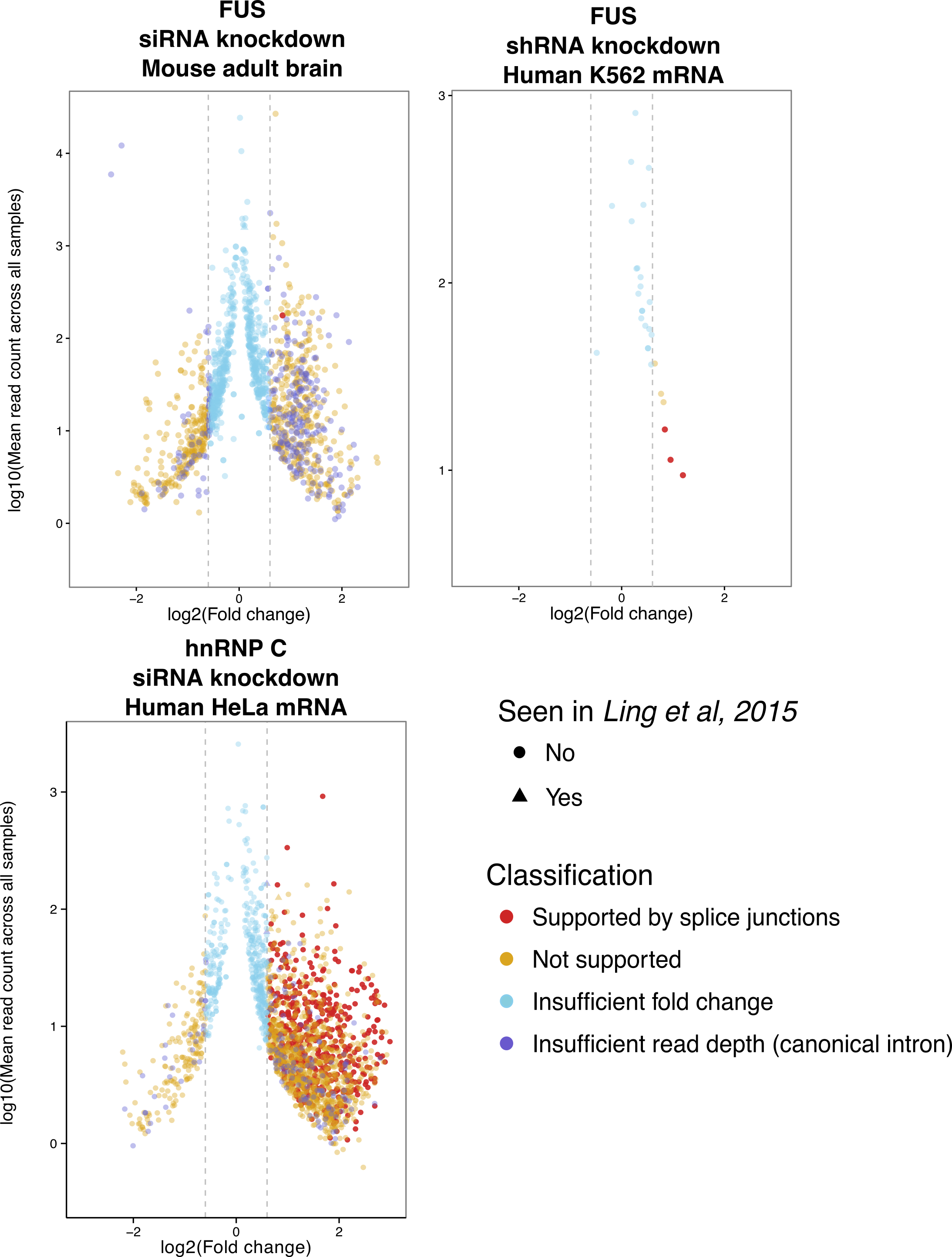

Supplement: Supplementary file 1 — Pre-classification output of CryptEx pipeline demonstrates a variety of novel splicing events in TDP-43, FUS and hnRNP C depletion data. Every novel splicing event plotted by mean depth of reads covering the novel event against log2(fold change) between depletion and control samples. The cryptic exon classifier throws out any splicing event where the canonical intron in which the cryptic splicing event appears is represented by less than five spliced reads (purple) or where the |log2(fold change)| < 0.6 (light blue). Splicing events are classified as cryptic exons if the spliced reads agree with the rest of the reads and have at least 1 spliced read per sample (red). Those that fail this step are coloured orange. (ZIP 1386 kb) [file 12920_2017_274_MOESM1_ESM.zip › Figure_S1_volcano_partR2_2.tif]

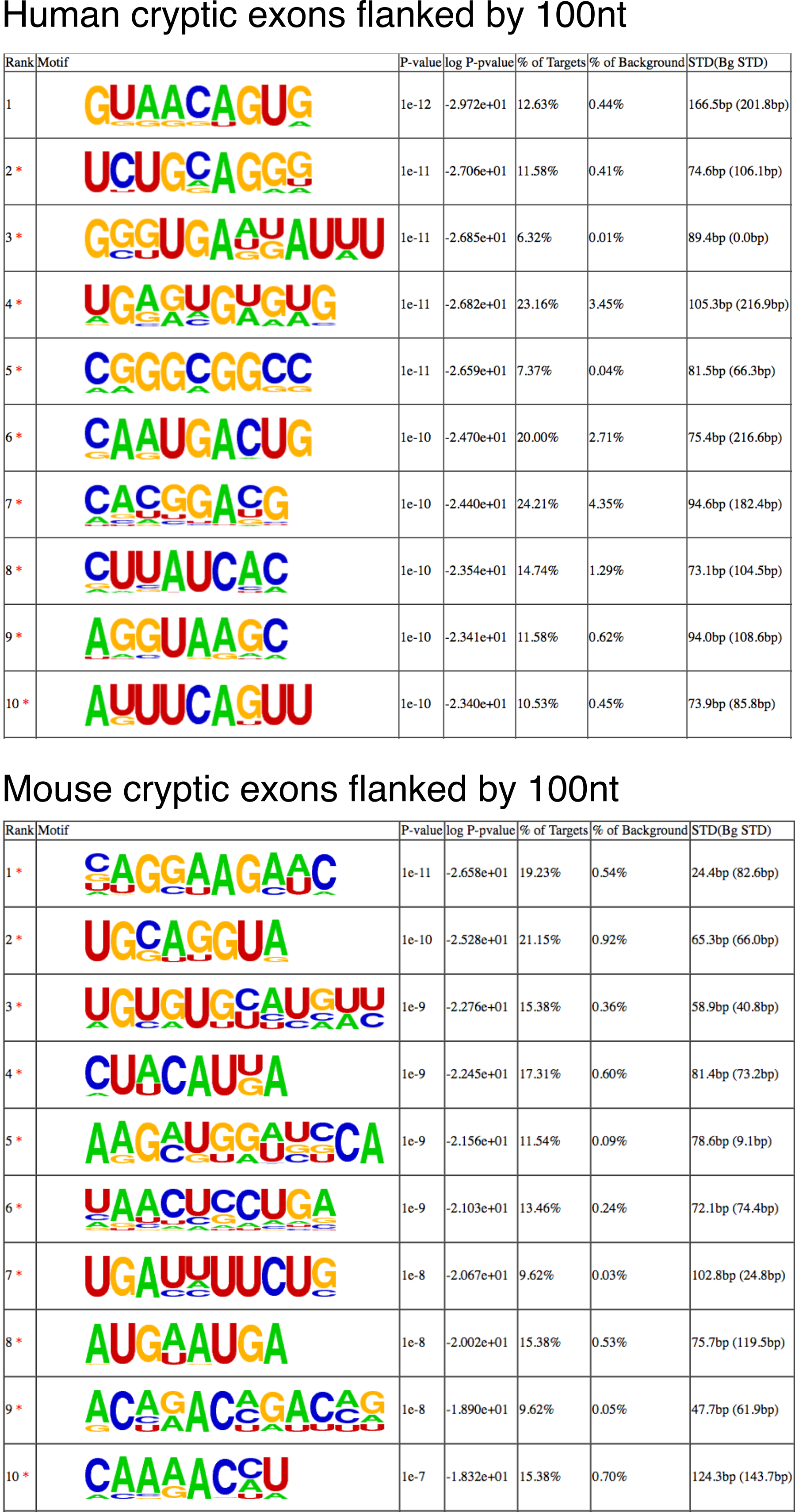

Supplement: Supplementary file 6 — Motif finding with HOMER The top ten motifs reported by the algorithm when comparing flanked cryptic exons with adjacent intronic sequence. The red asterisk indicates that the motif is potentially a false positive result. (TIF 1799 kb) [file 12920_2017_274_MOESM6_ESM.tif]
