# Supplementary material for: Quantitative analysis of cryptic splicing associated with TDP-43 depletion
Source: BMC Med Genomics. 2017 May 26;10:38. doi: 10.1186/s12920-017-0274-1 (PMC5446763; doi:10.1186/s12920-017-0274-1)

Figure S2

Mouse Cryptic Exons from *Quantitative analysis of cryptic splicing associated with TDP-43 depletion*

Jack Humphrey, Warren Emmett, Pietro Fratta, Adrian M. Isaacs & Vincent Plagnol

September 16, 2016

Each cryptic exon discovered in either of the two mouse datasets was visualised in both datasets using the IGV browser. For each dataset, the biological replicates were combined to create merged files for each condition. Read coverage and splice junctions are shown. The Ensembl transcripts for the mm10 build are provided, as are the coordinates of the cryptic exons discovered by CryptEx.

1 Abr E026i1

Mouse Adult Brain

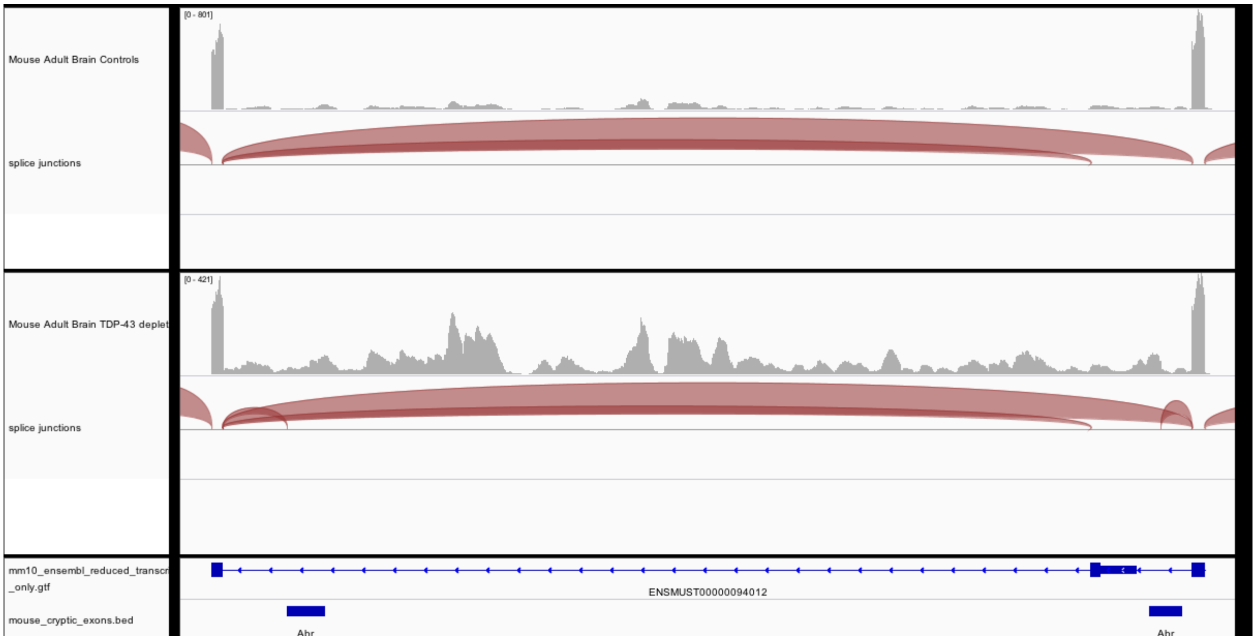

Mouse Embryonic Stem Cells

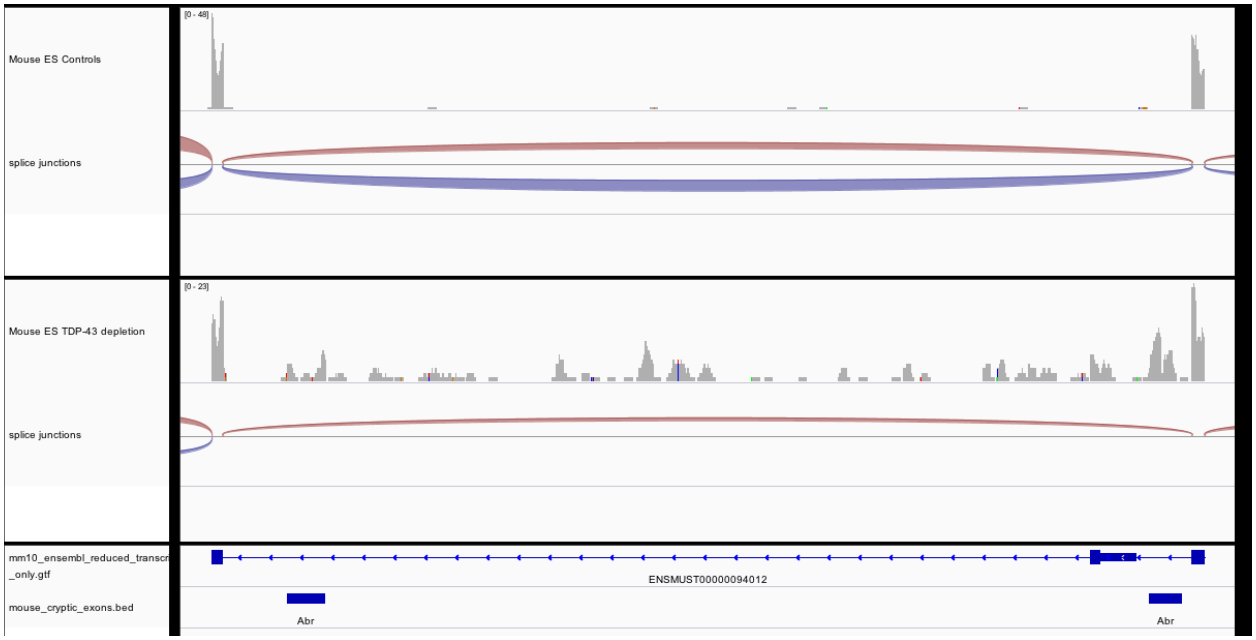

2 Pnpla6 E016i1

Mouse Adult Brain

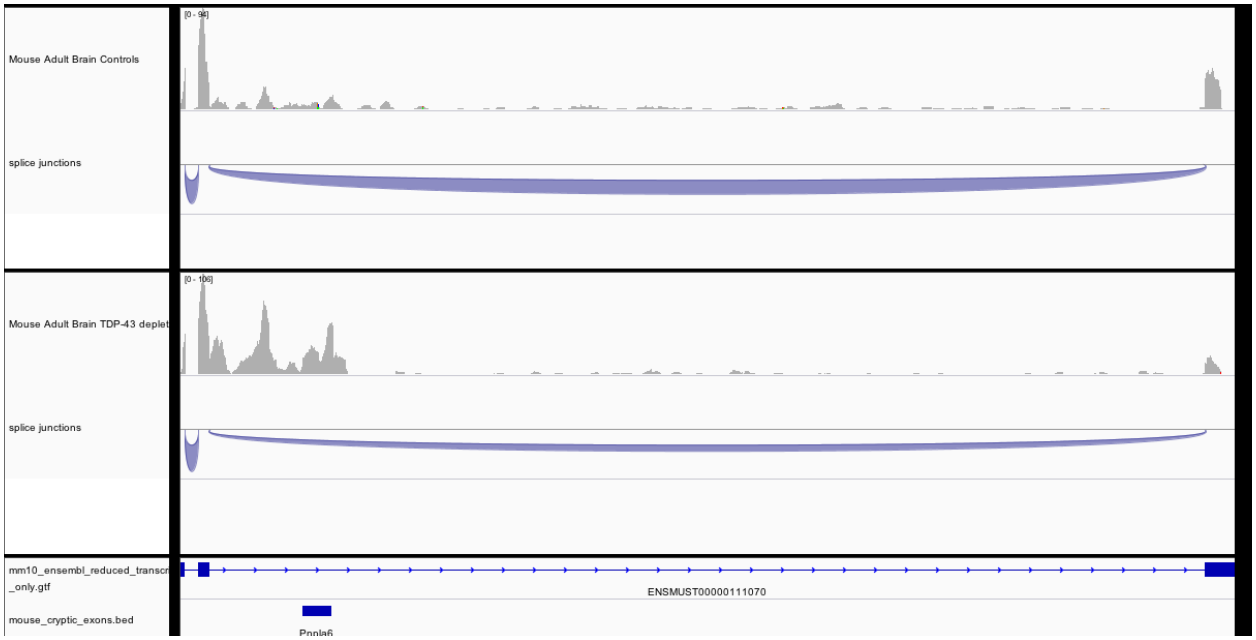

Mouse Embryonic Stem Cells

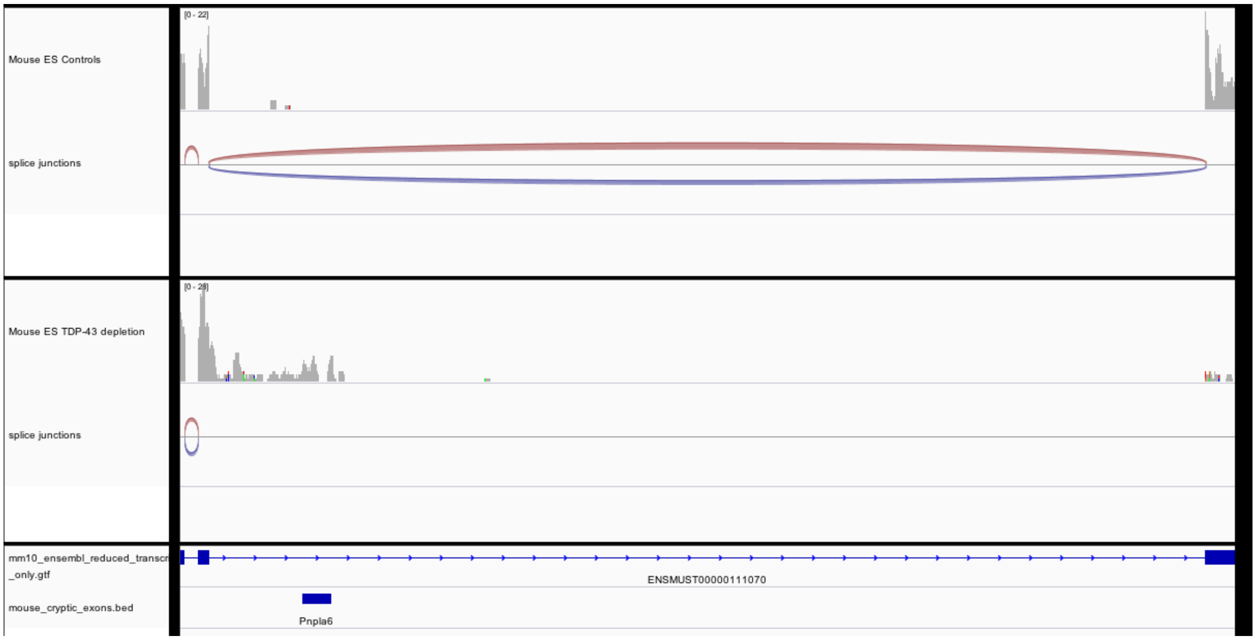

3 Lrrc49 E017i3

Mouse Adult Brain

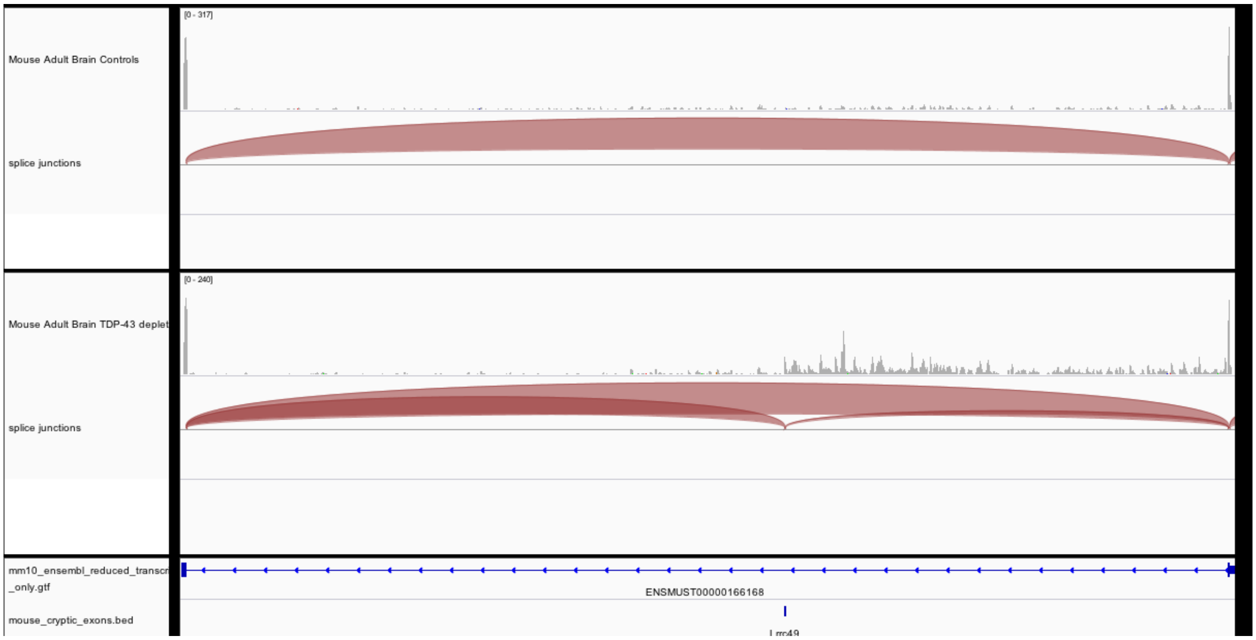

Mouse Embryonic Stem Cells

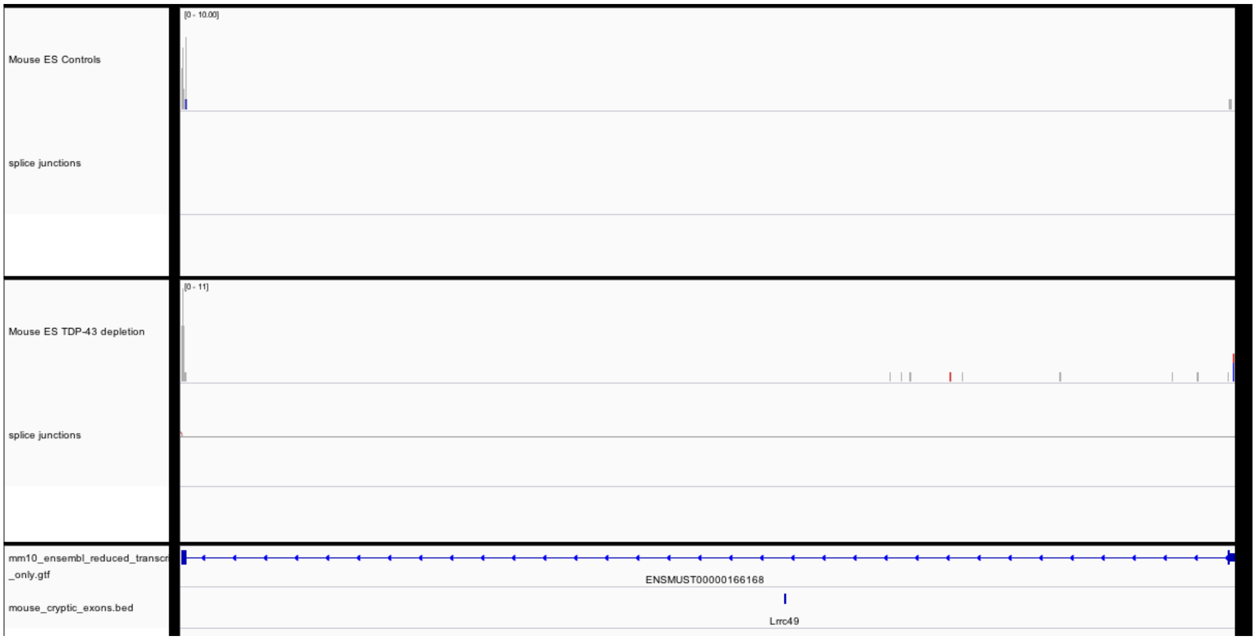

4 Ccdc149 E009i3

Mouse Adult Brain

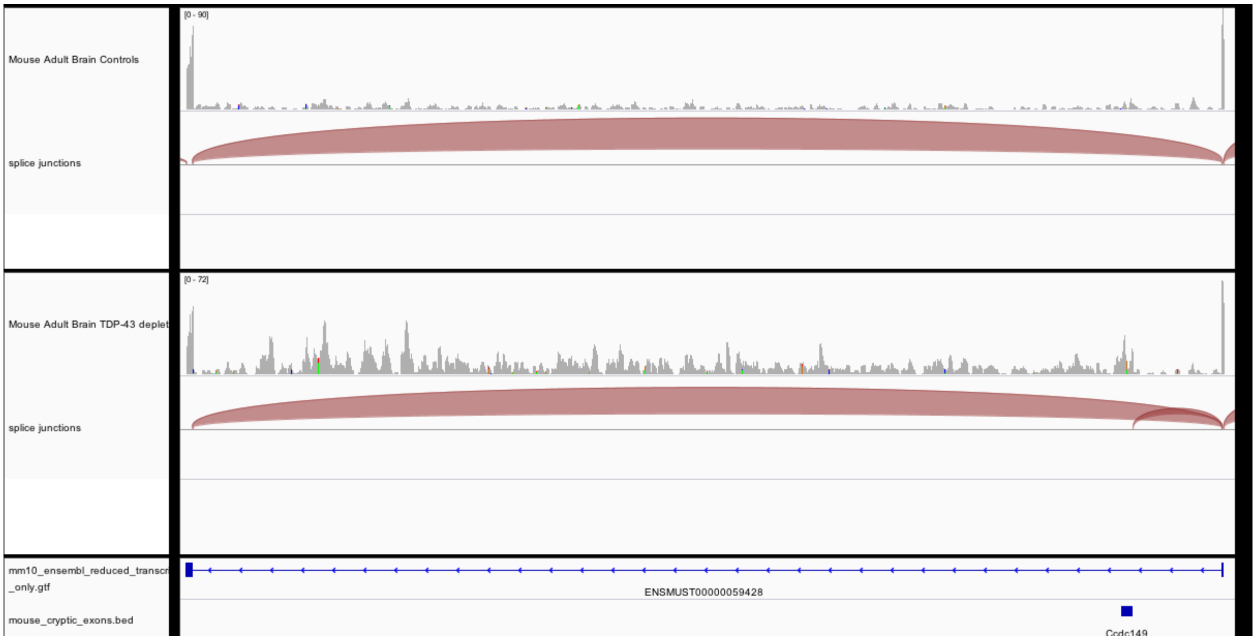

Mouse Embryonic Stem Cells

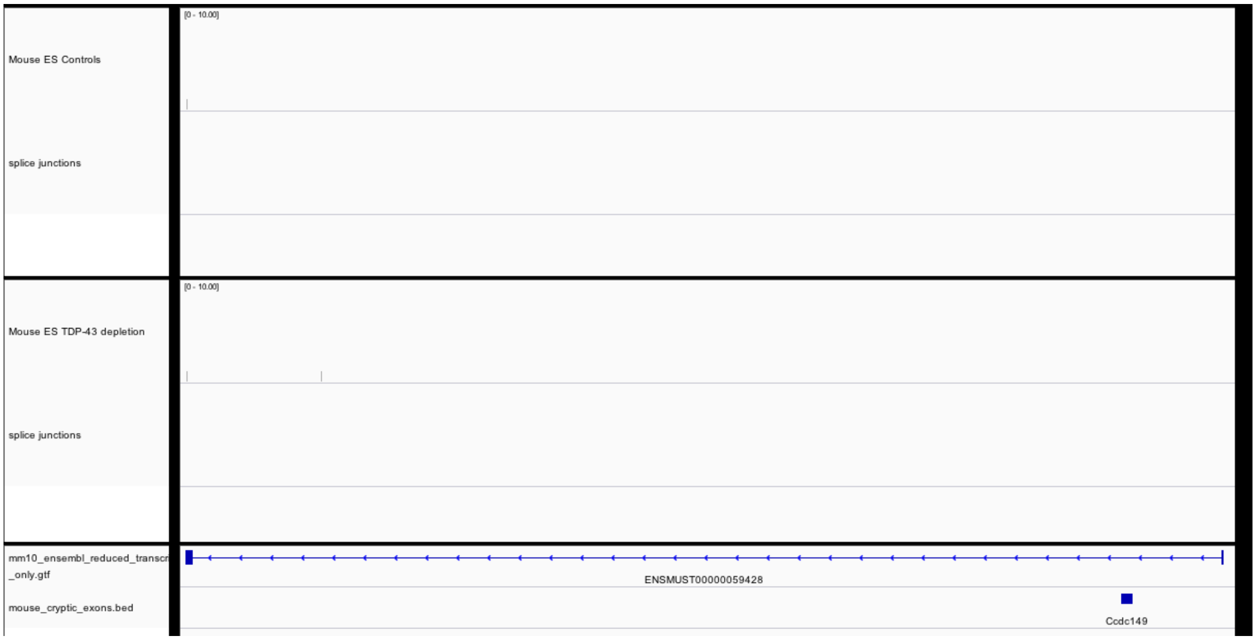

5 Ak5 E014i6

Mouse Adult Brain

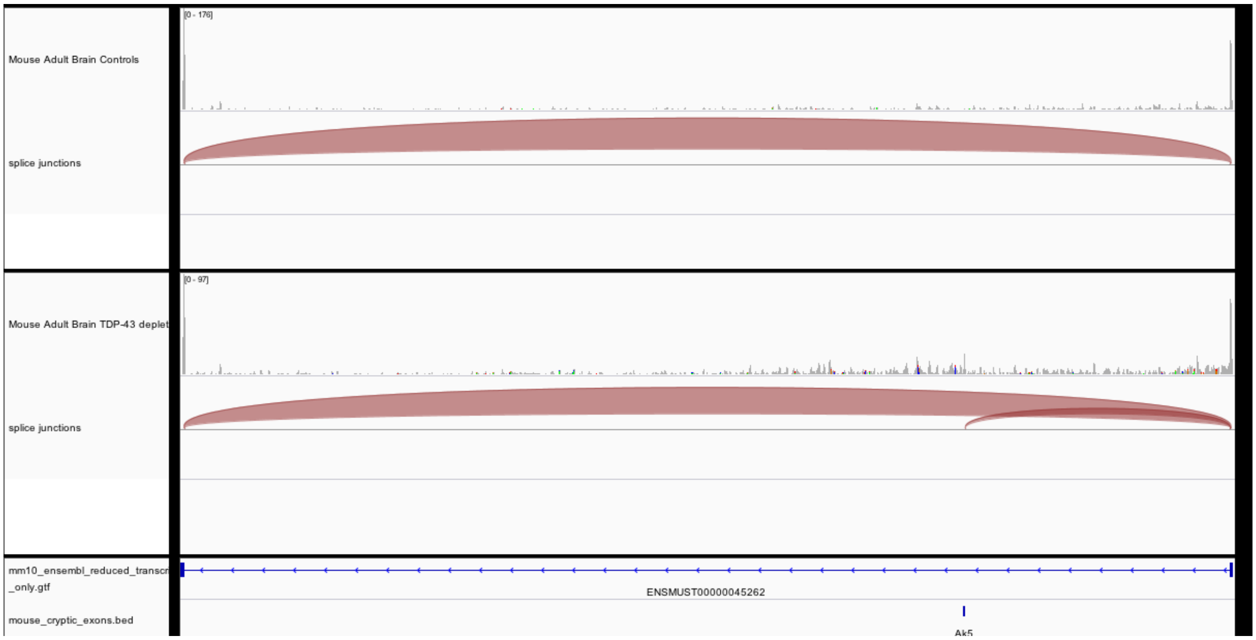

Mouse Embryonic Stem Cells

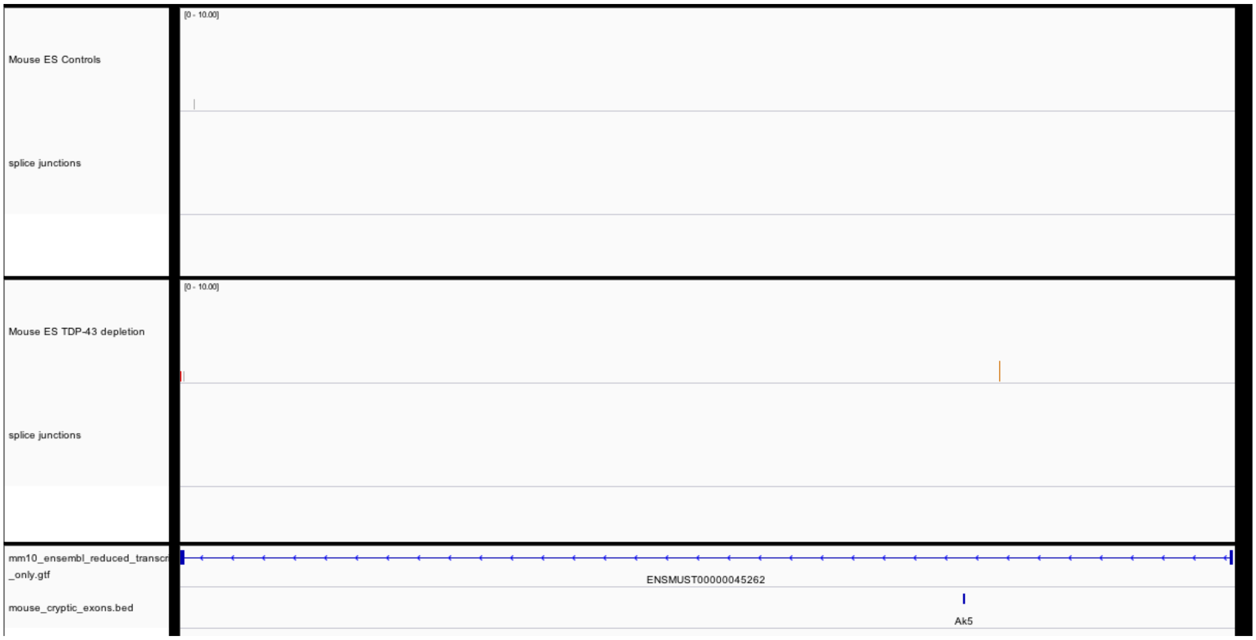

6 Kctd10 E017i2

Mouse Adult Brain

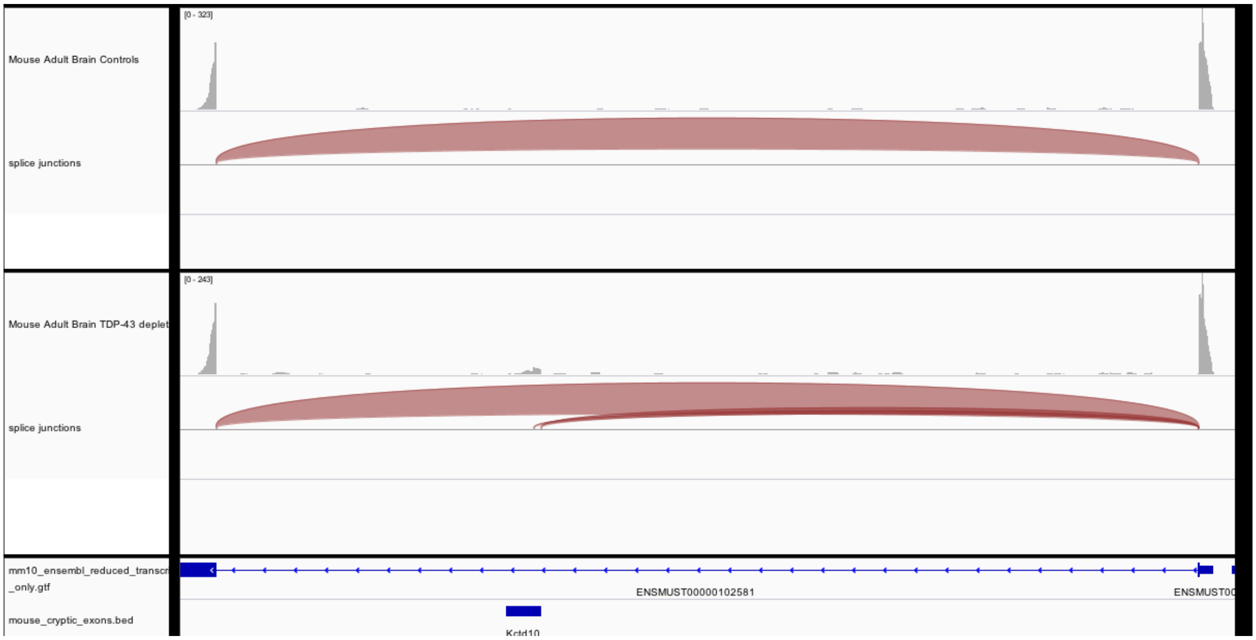

Mouse Embryonic Stem Cells

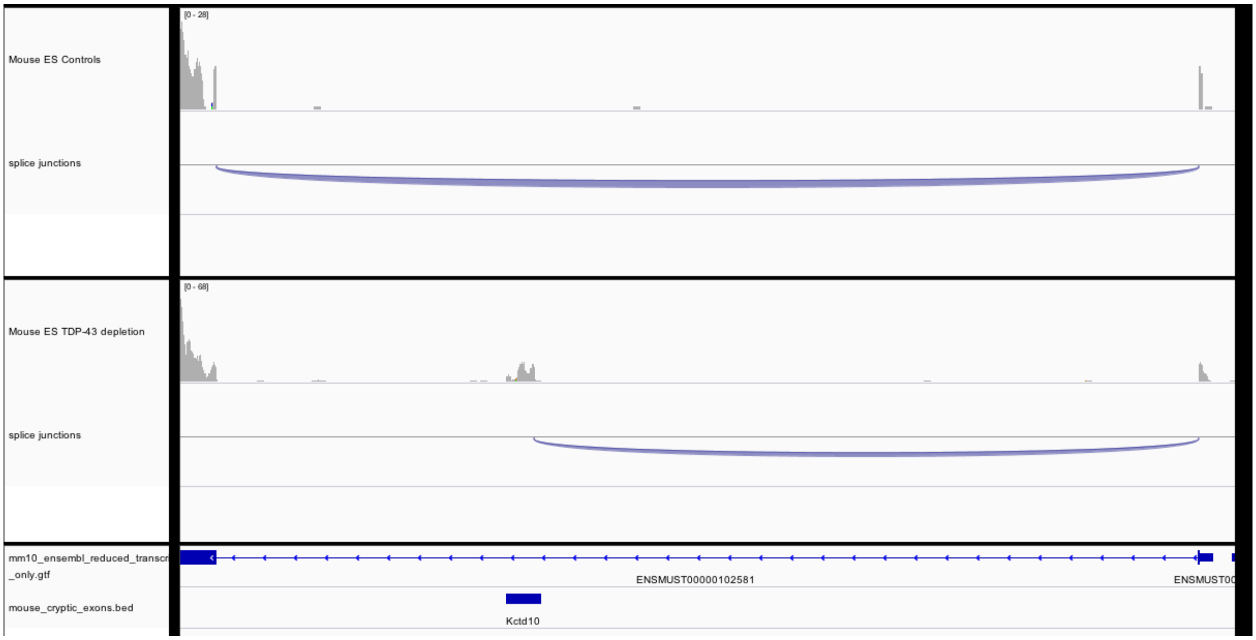

7 Tmem14a E005i1

Mouse Adult Brain

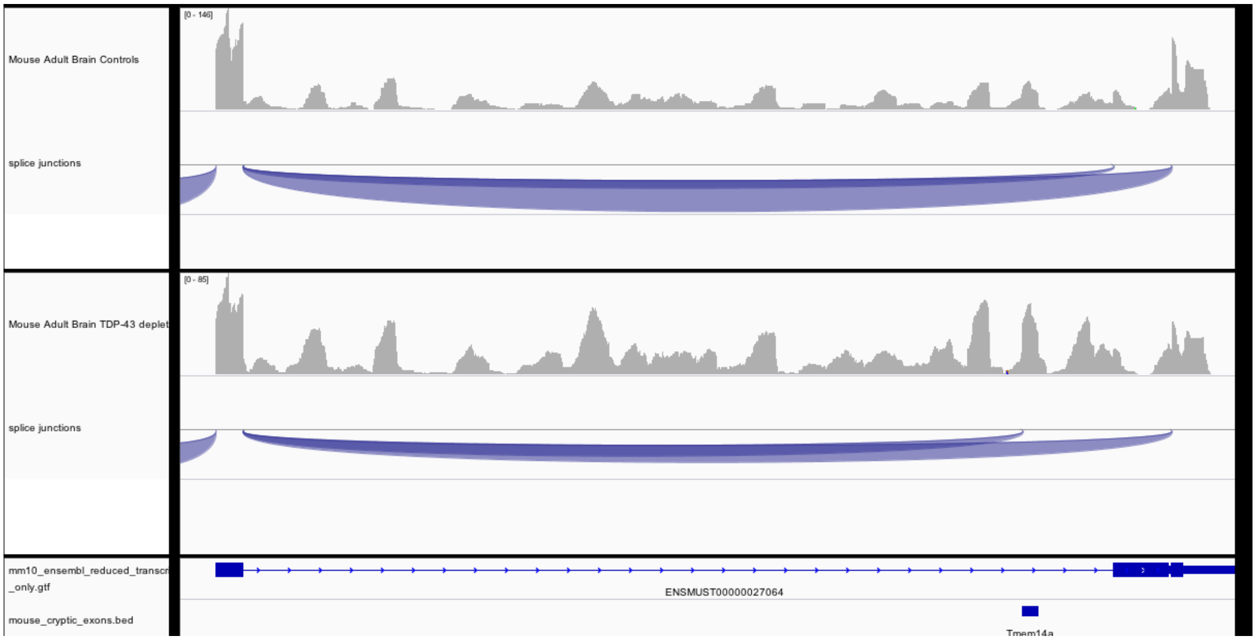

Mouse Embryonic Stem Cells

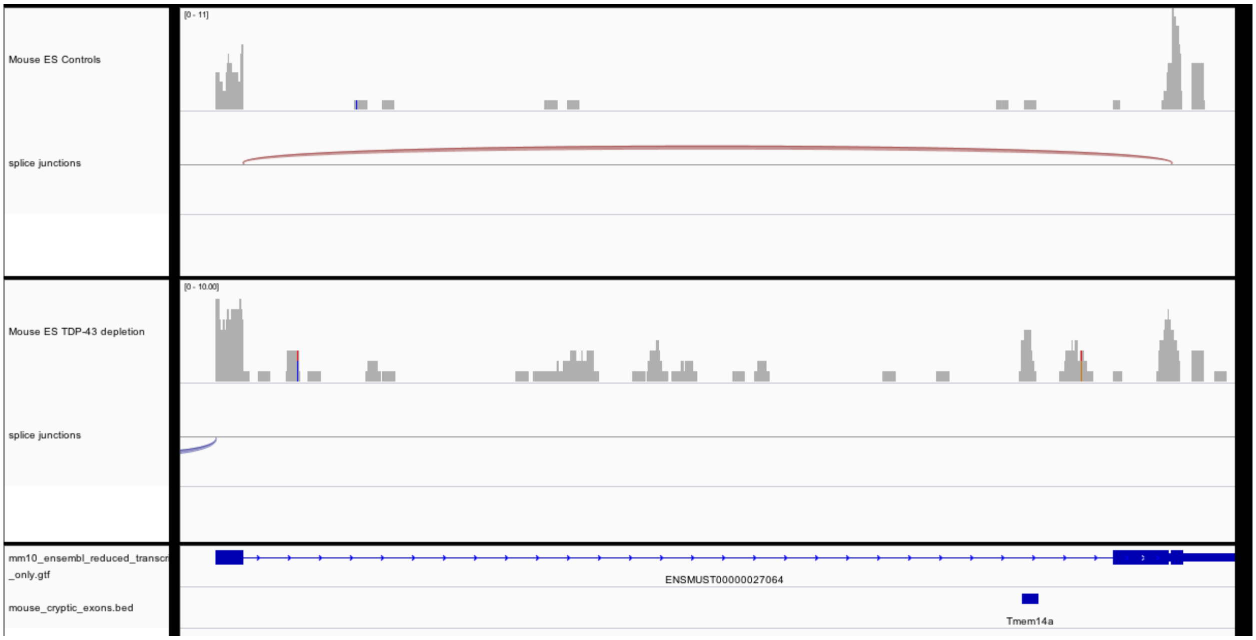

8 Apod E006i1

Mouse Adult Brain

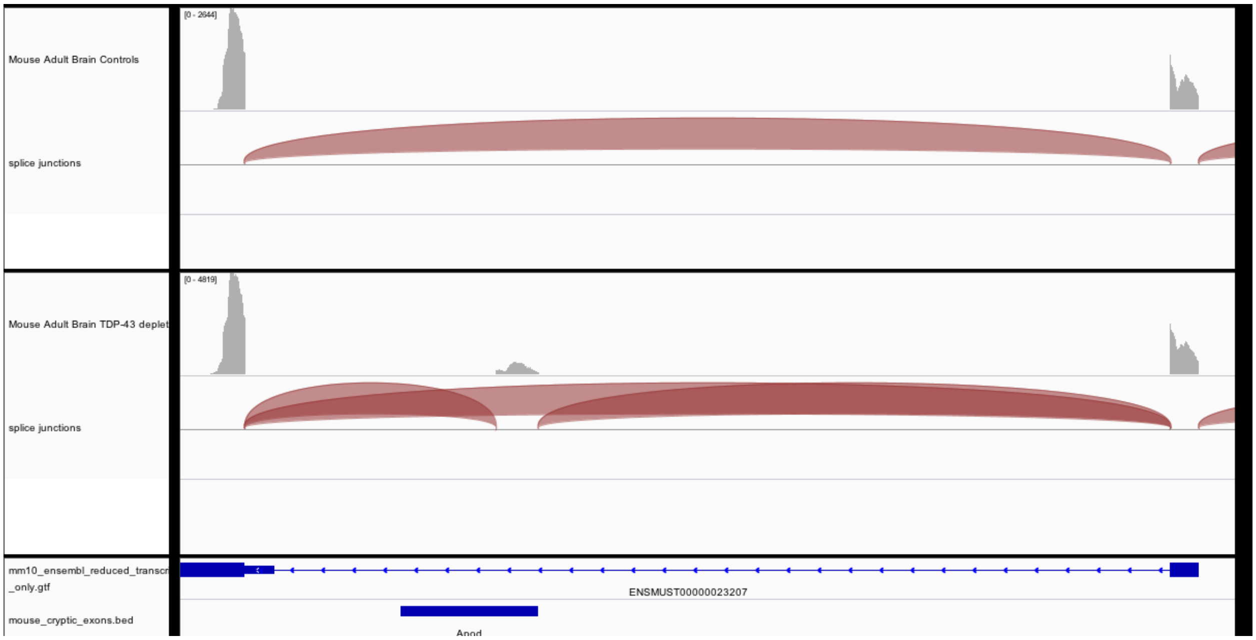

Mouse Embryonic Stem Cells

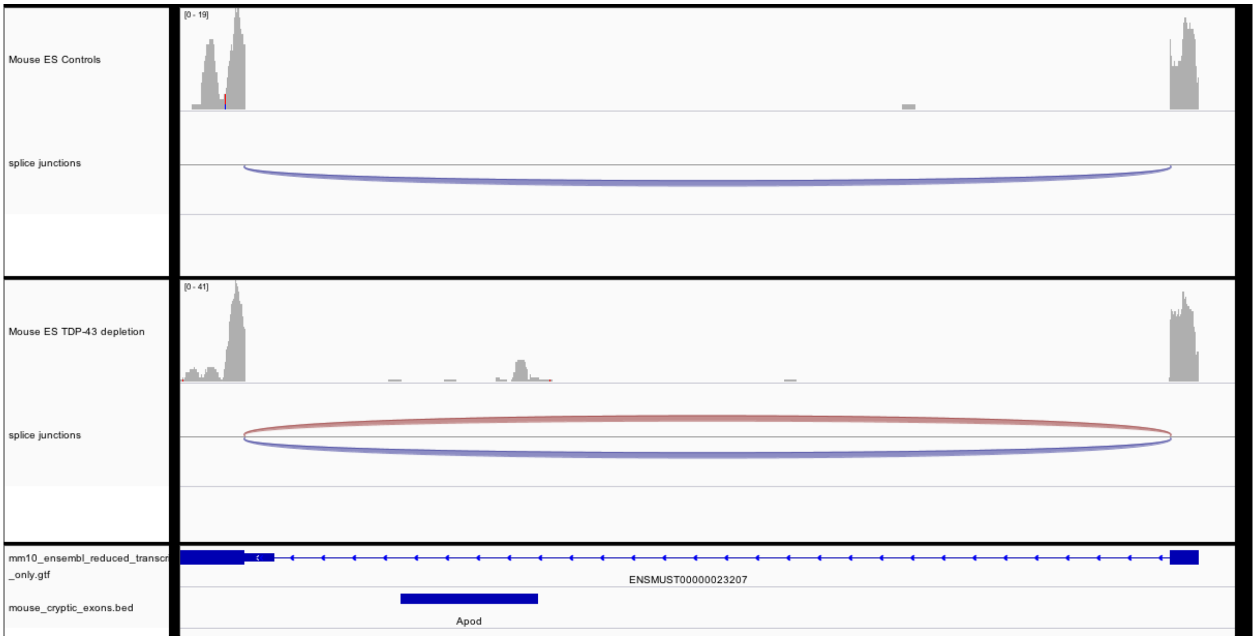

9 Erbb2ip E026i1

Mouse Adult Brain

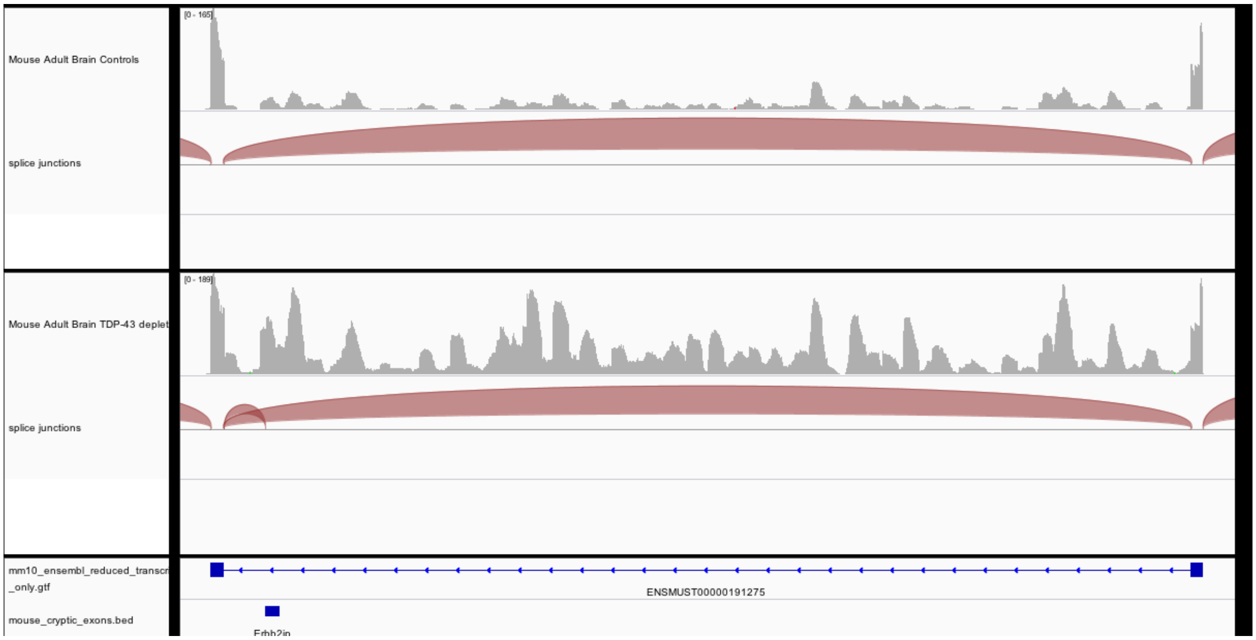

Mouse Embryonic Stem Cells

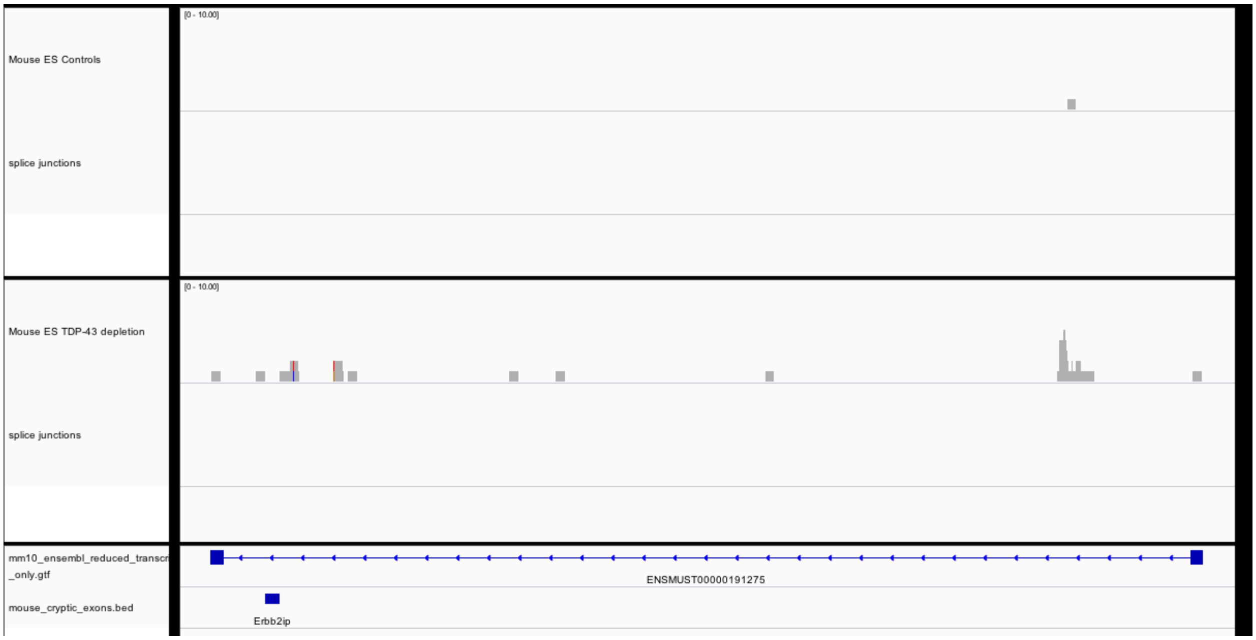

10 Trim8 E001i2

Mouse Adult Brain

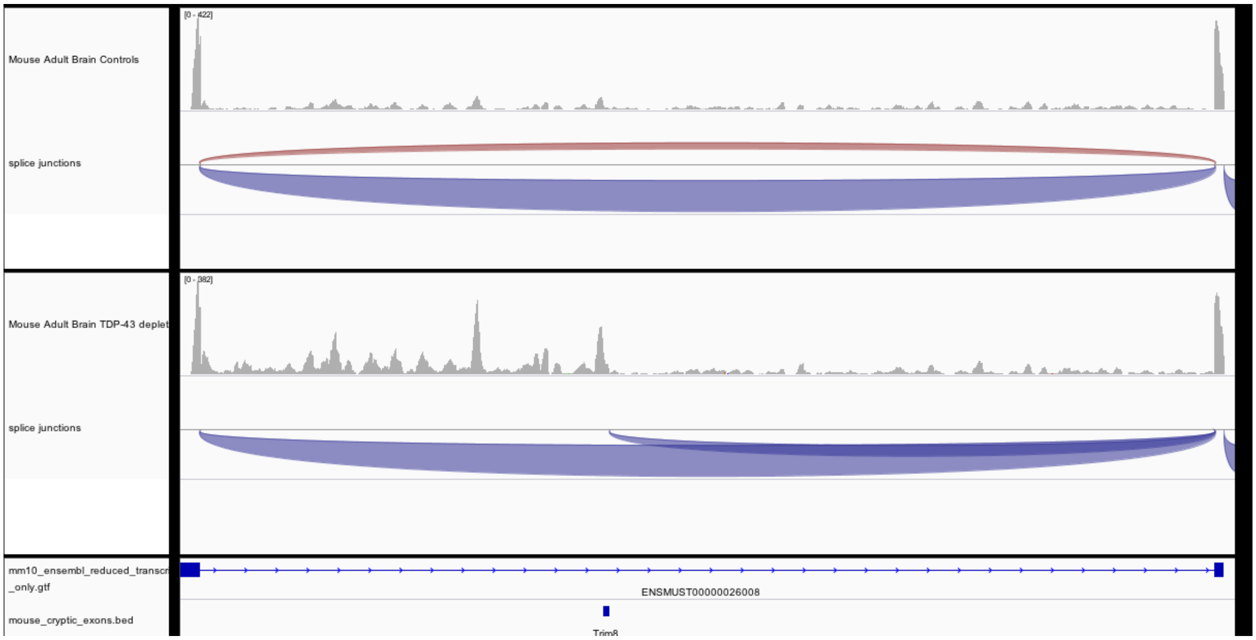

Mouse Embryonic Stem Cells

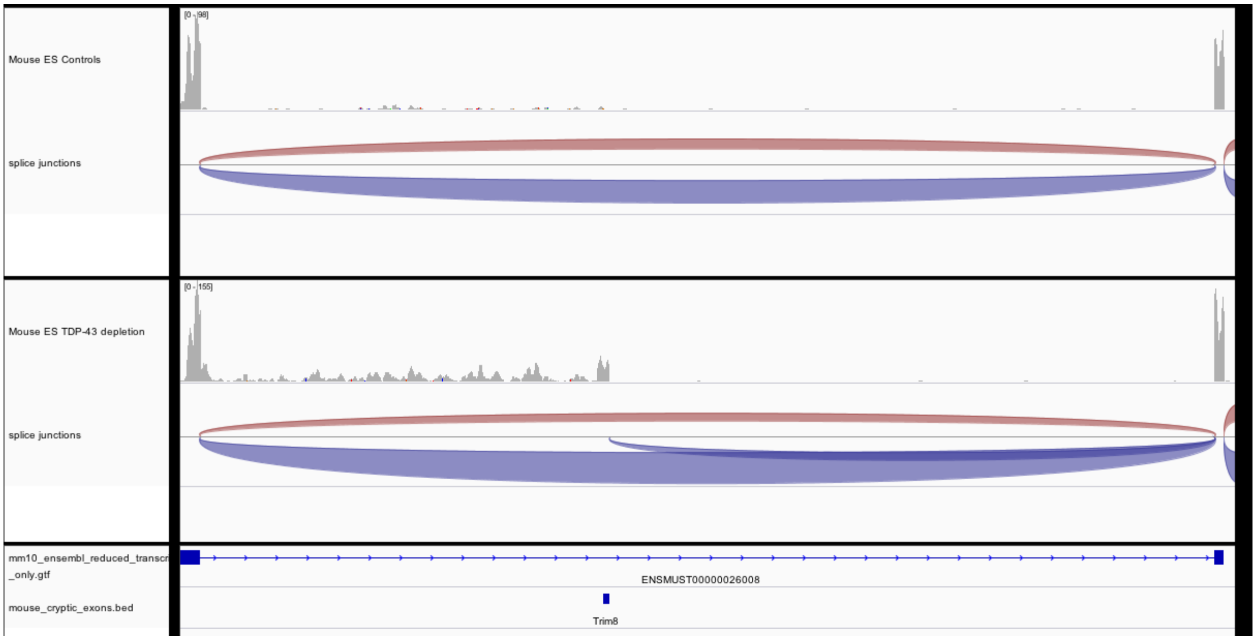

11 Usp15 E015i3

Mouse Adult Brain

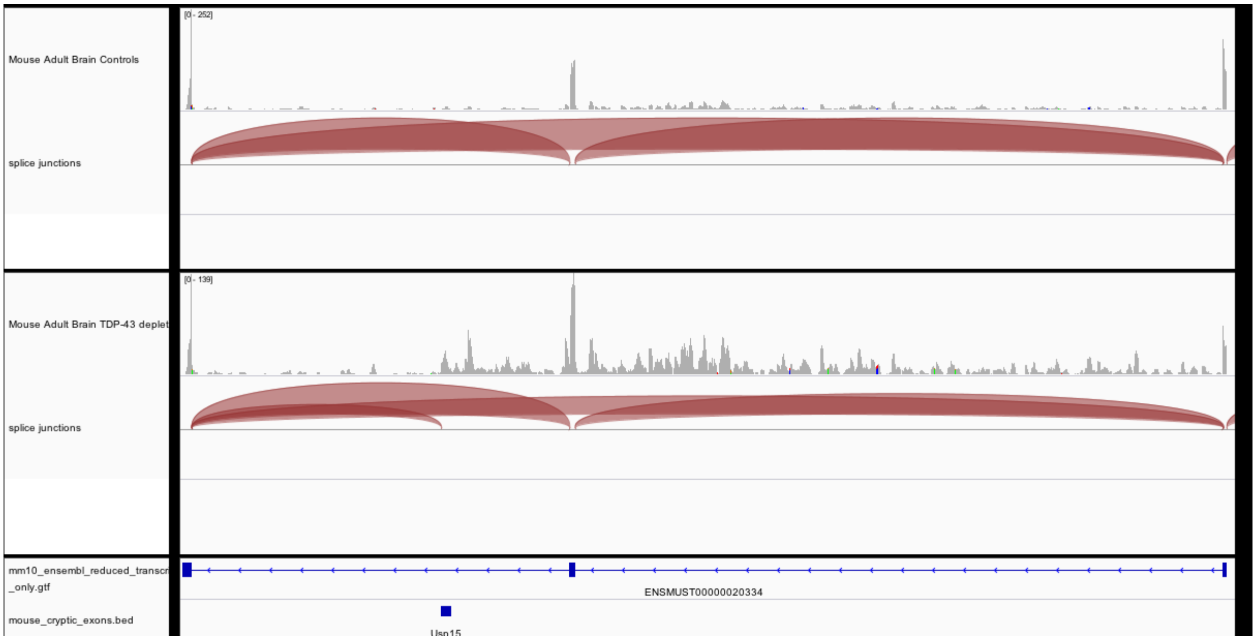

Mouse Embryonic Stem Cells

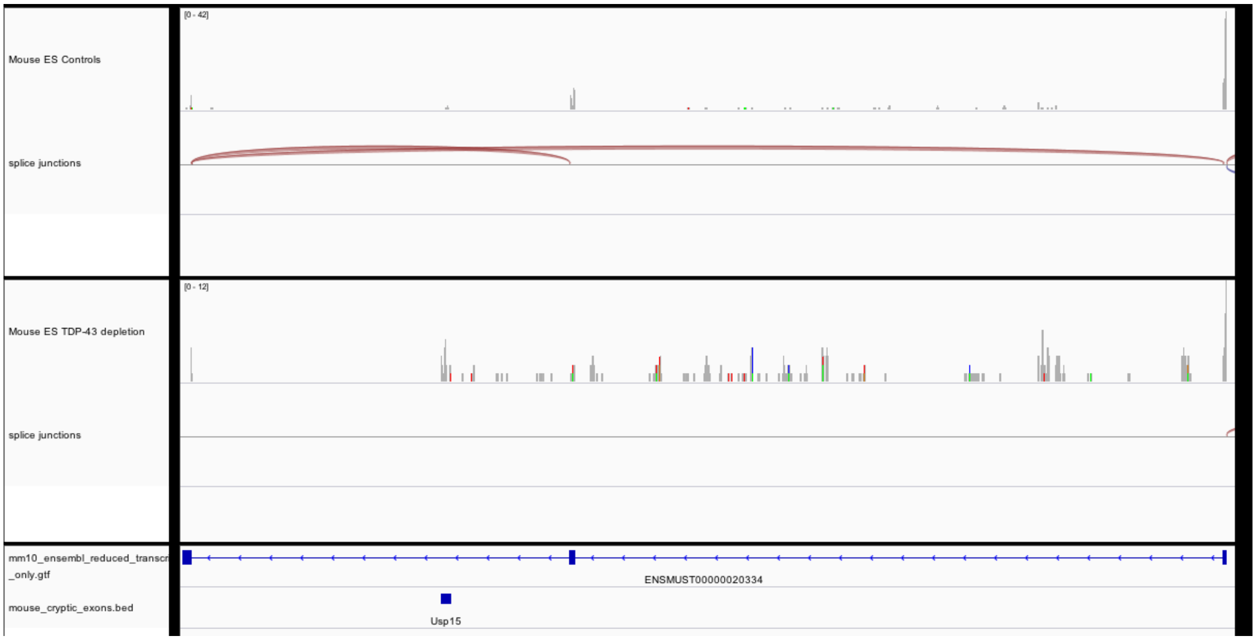

12 Fam135a E008i1

Mouse Adult Brain

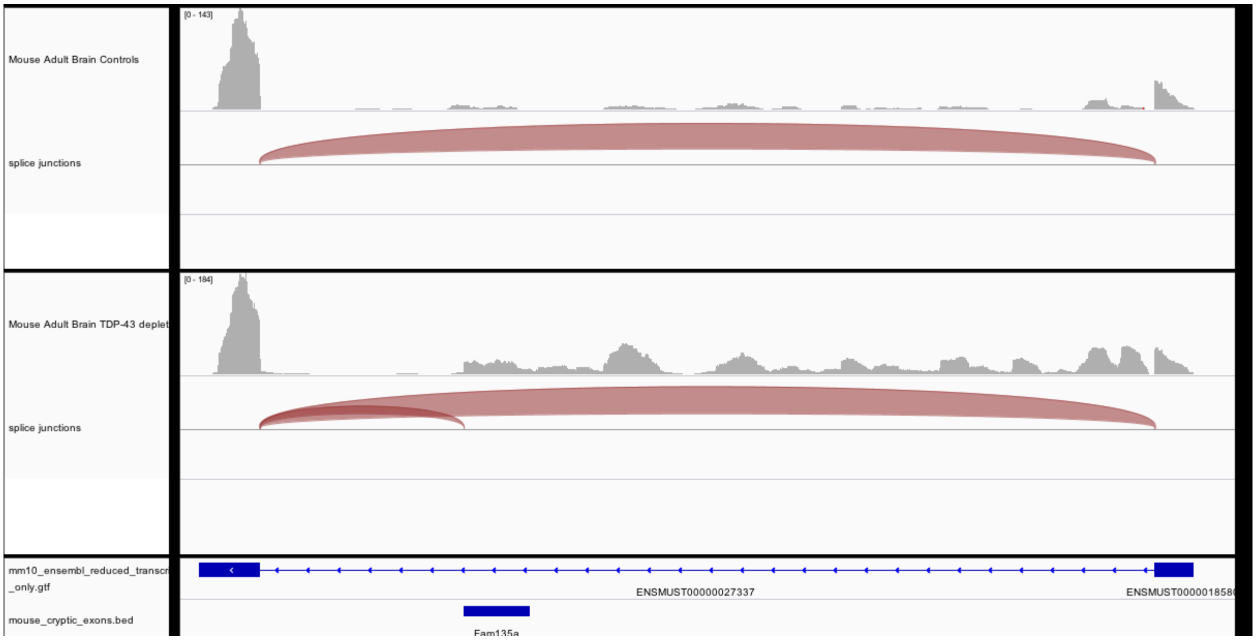

Mouse Embryonic Stem Cells

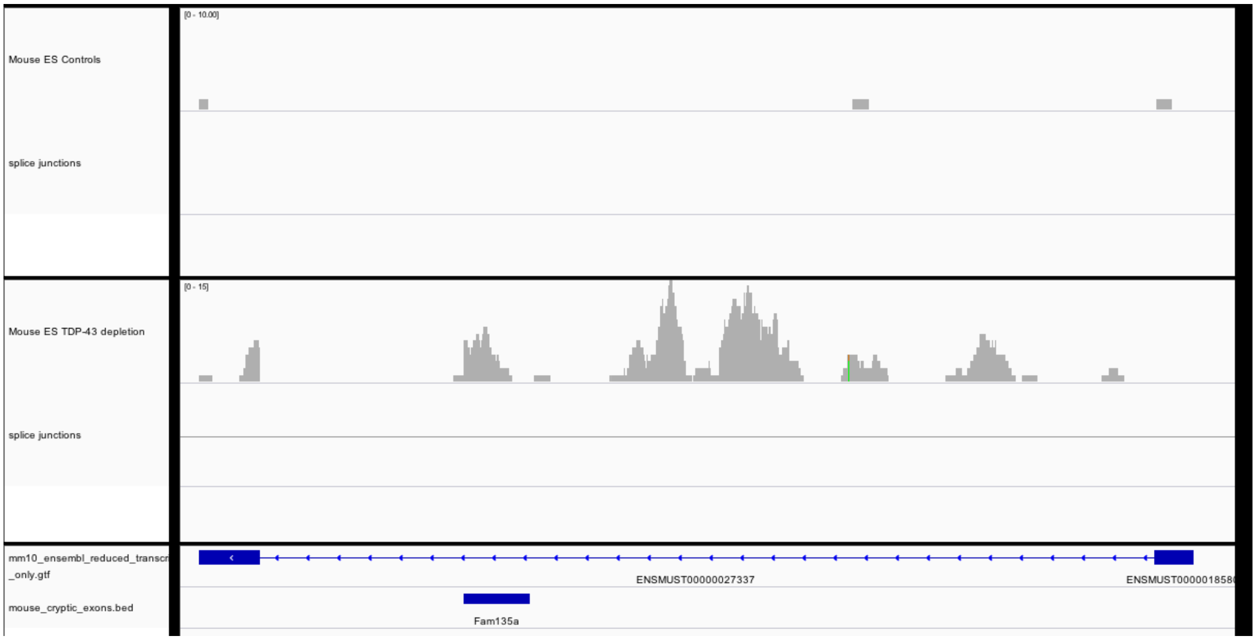

13 Rasal1 E007i1

Mouse Adult Brain

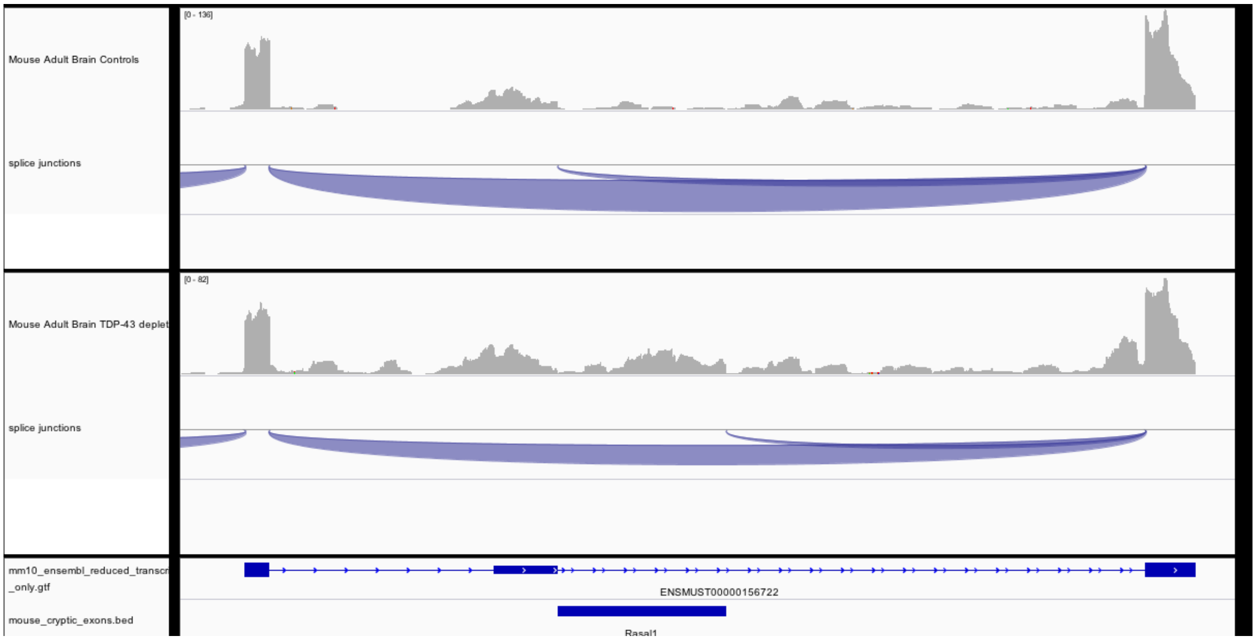

Mouse Embryonic Stem Cells

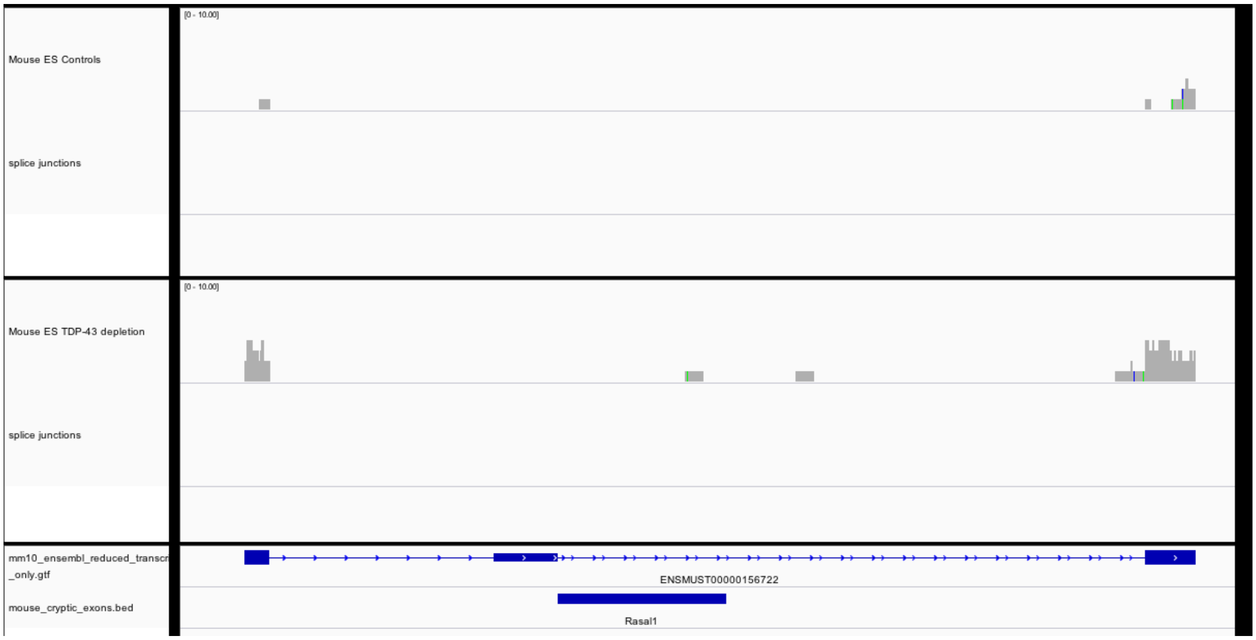

14 Brms1l E008i1

Mouse Adult Brain

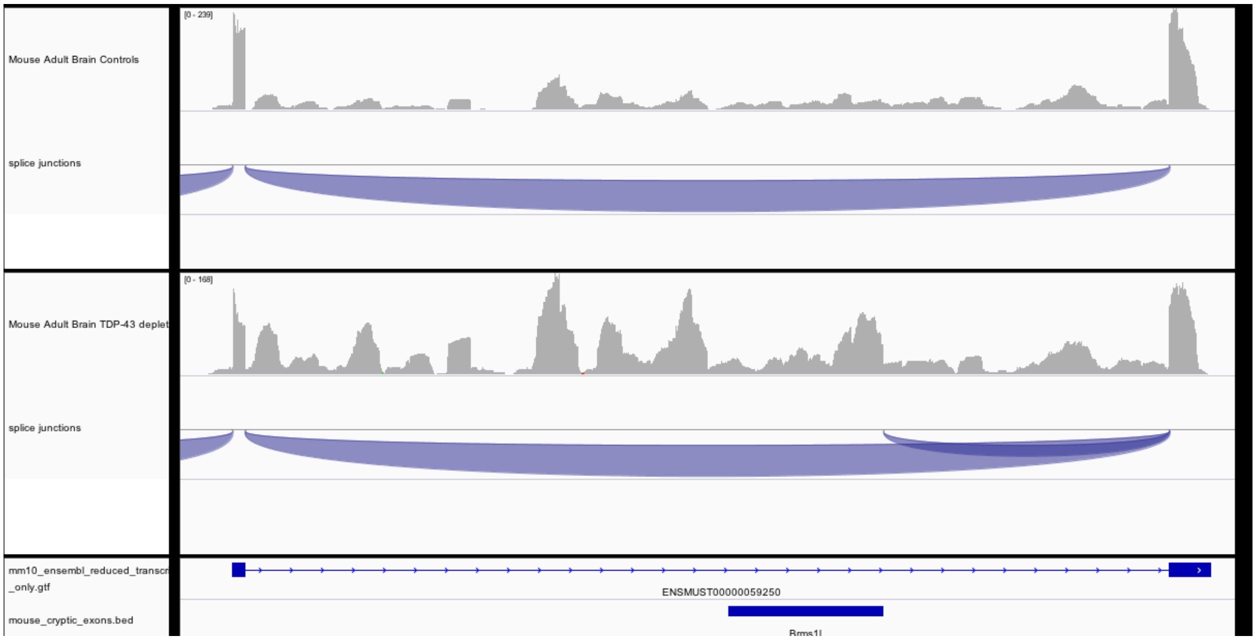

Mouse Embryonic Stem Cells

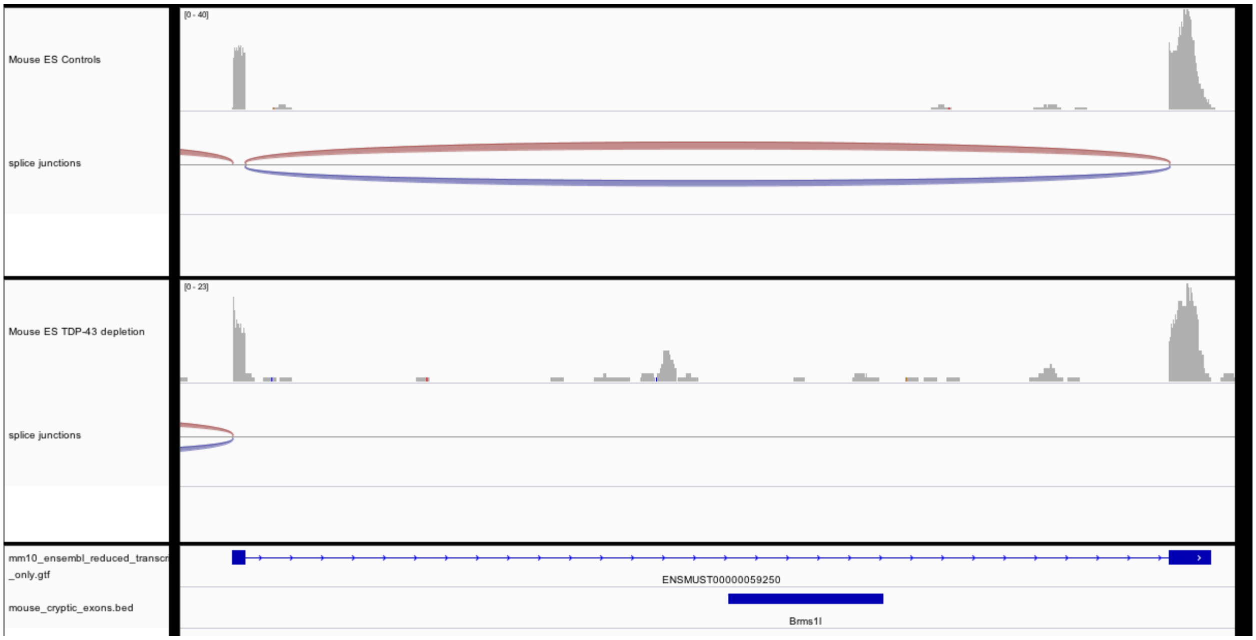

15 Cacna1b E050i1

Mouse Adult Brain

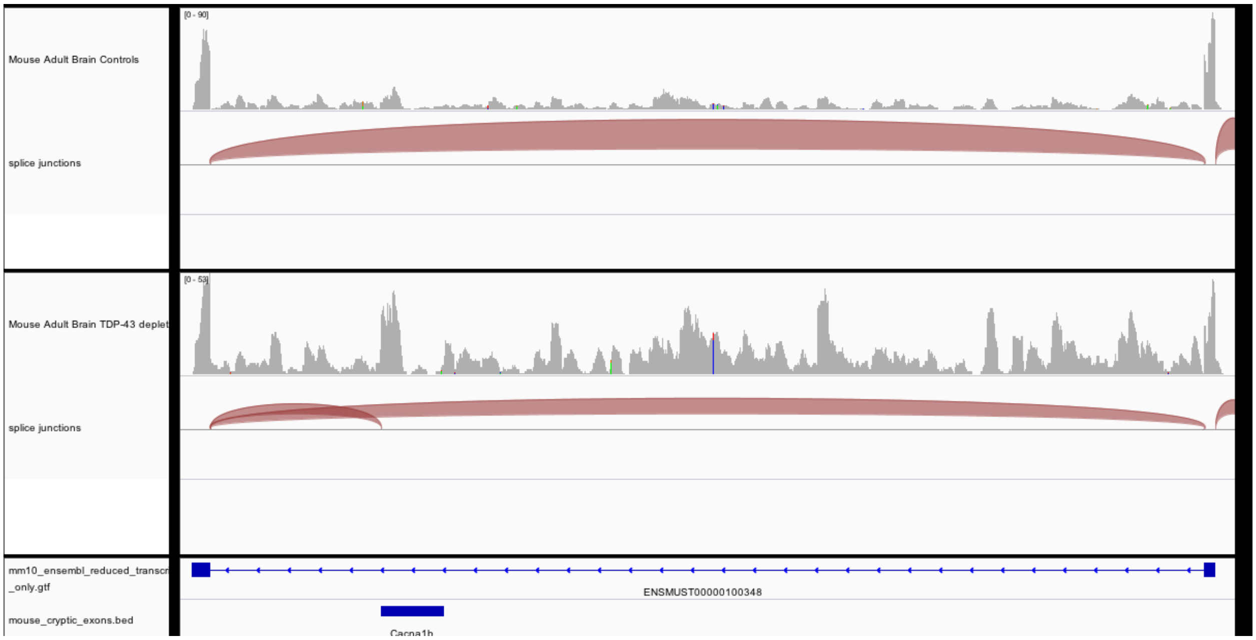

Mouse Embryonic Stem Cells

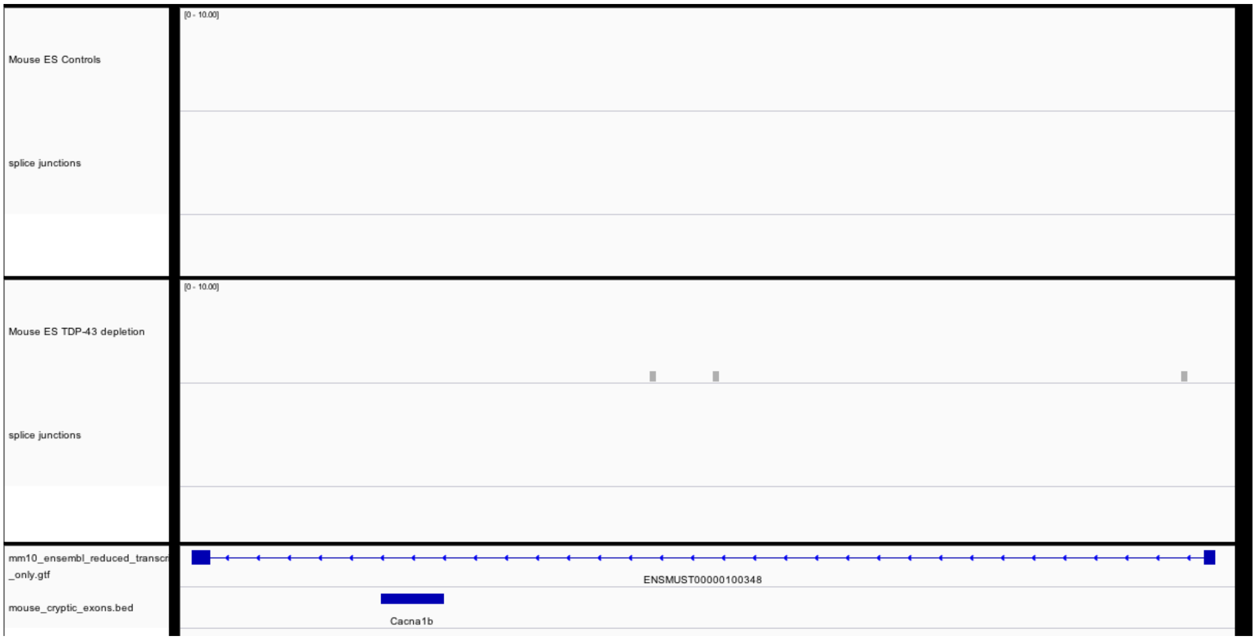

16 Nt5c3b E010i1

Mouse Adult Brain

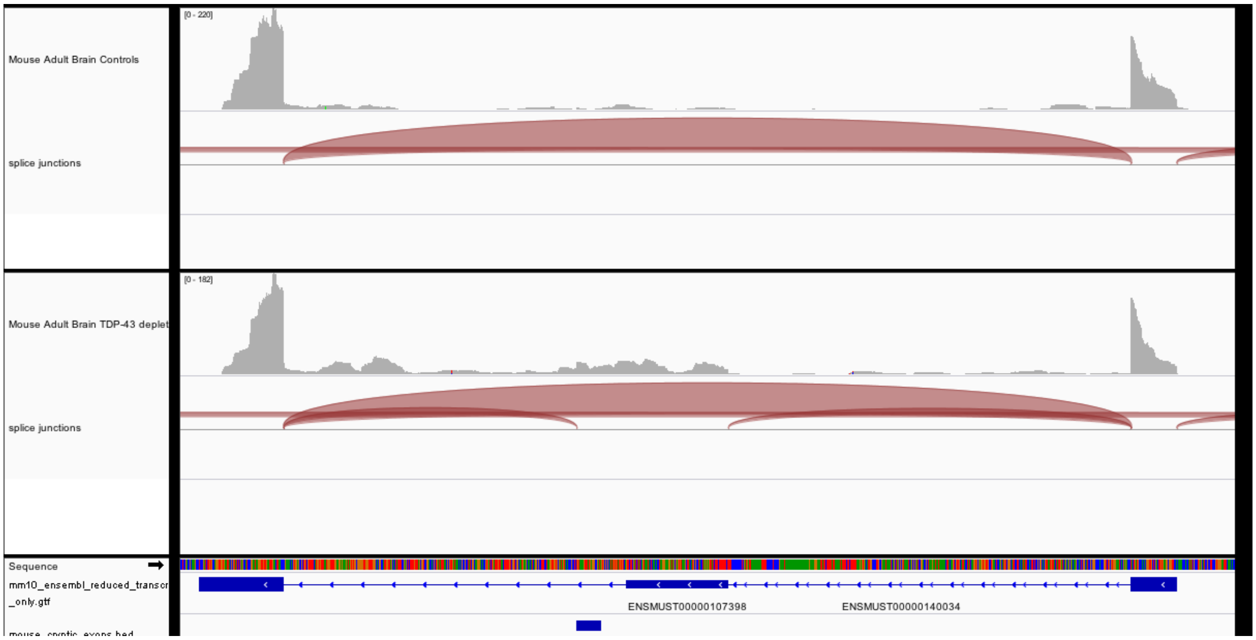

Mouse Embryonic Stem Cells

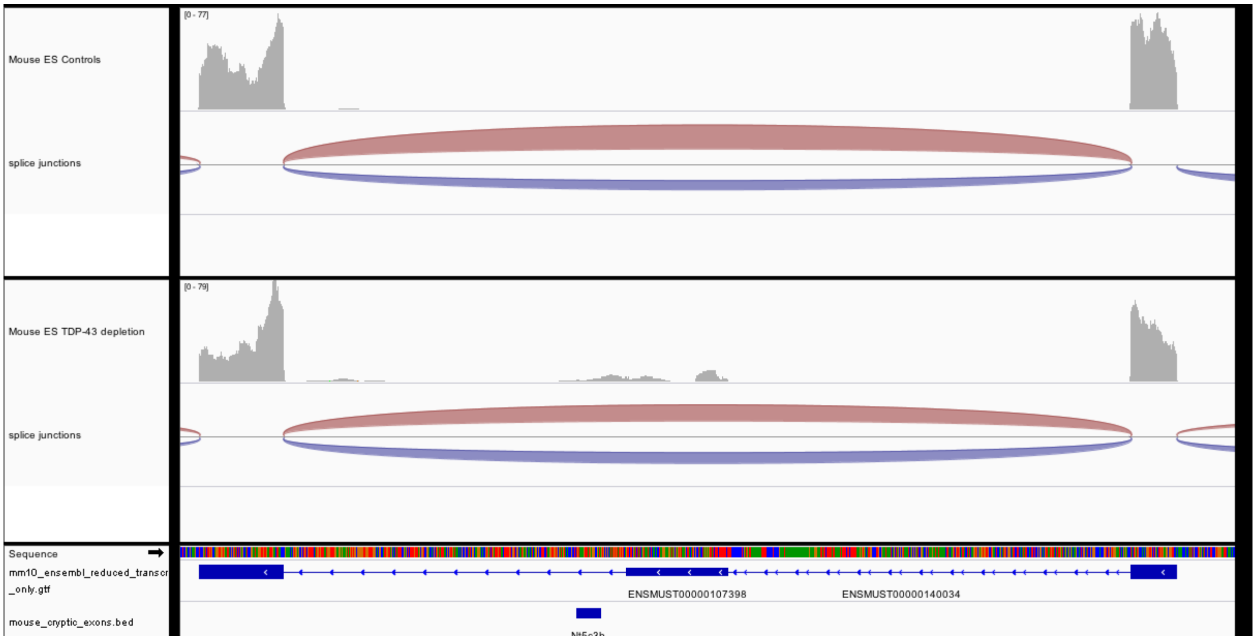

17 Spcs2 E004i1

Mouse Adult Brain

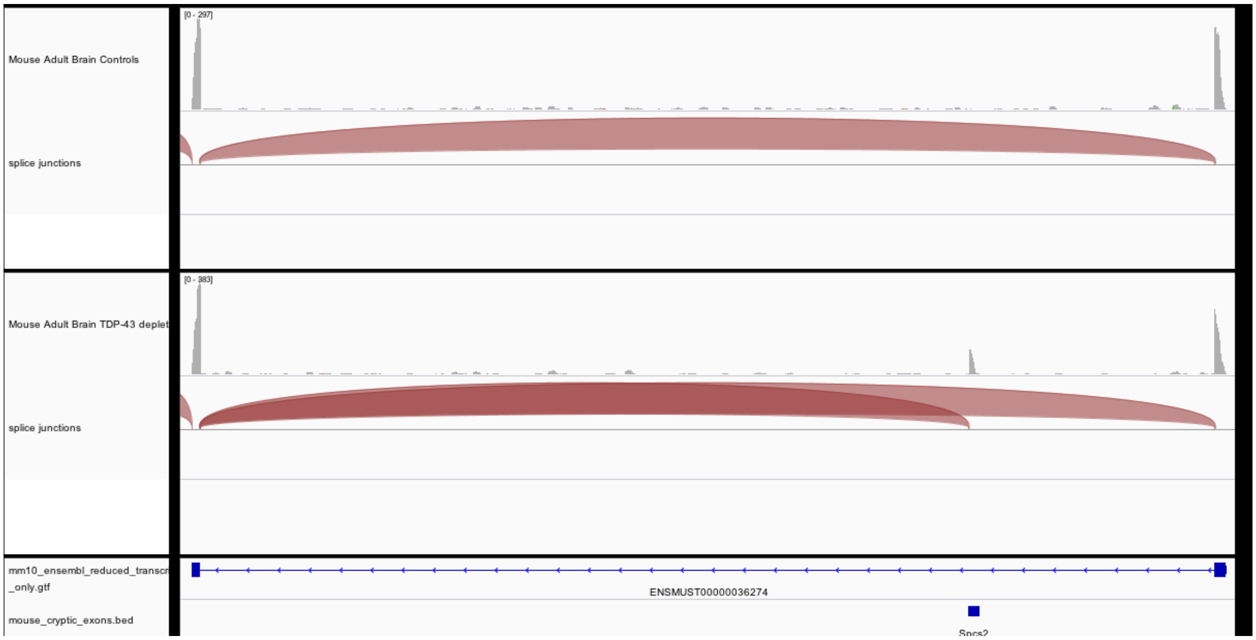

Mouse Embryonic Stem Cells

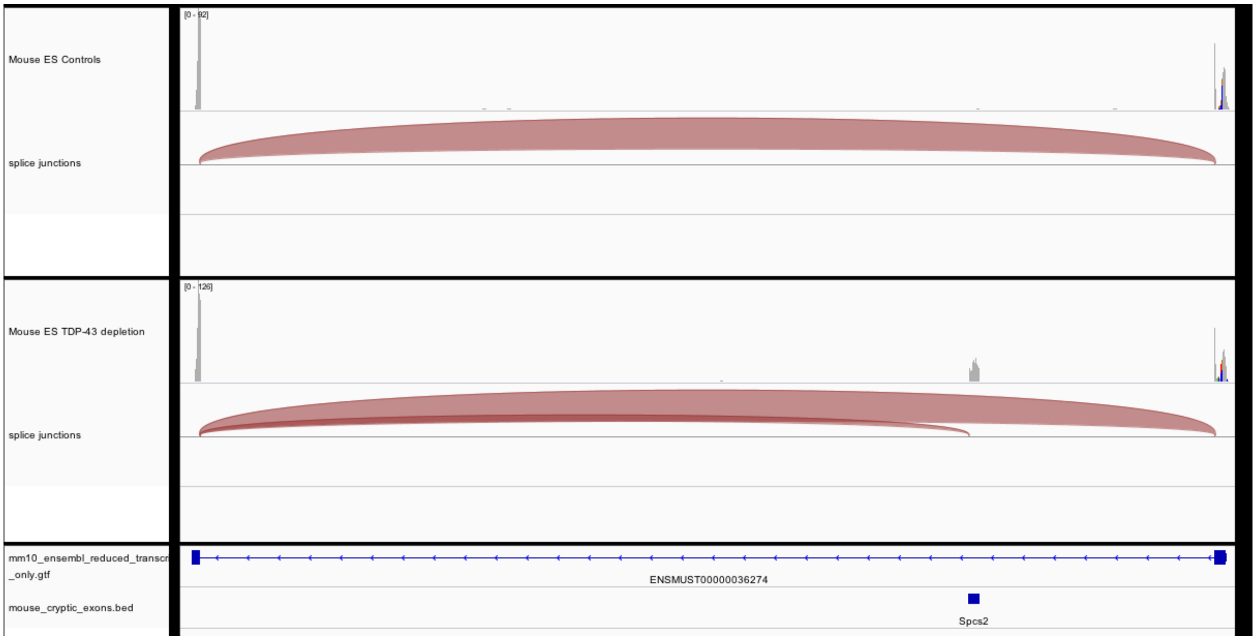

18 Ccdc88a E004i8

Mouse Adult Brain

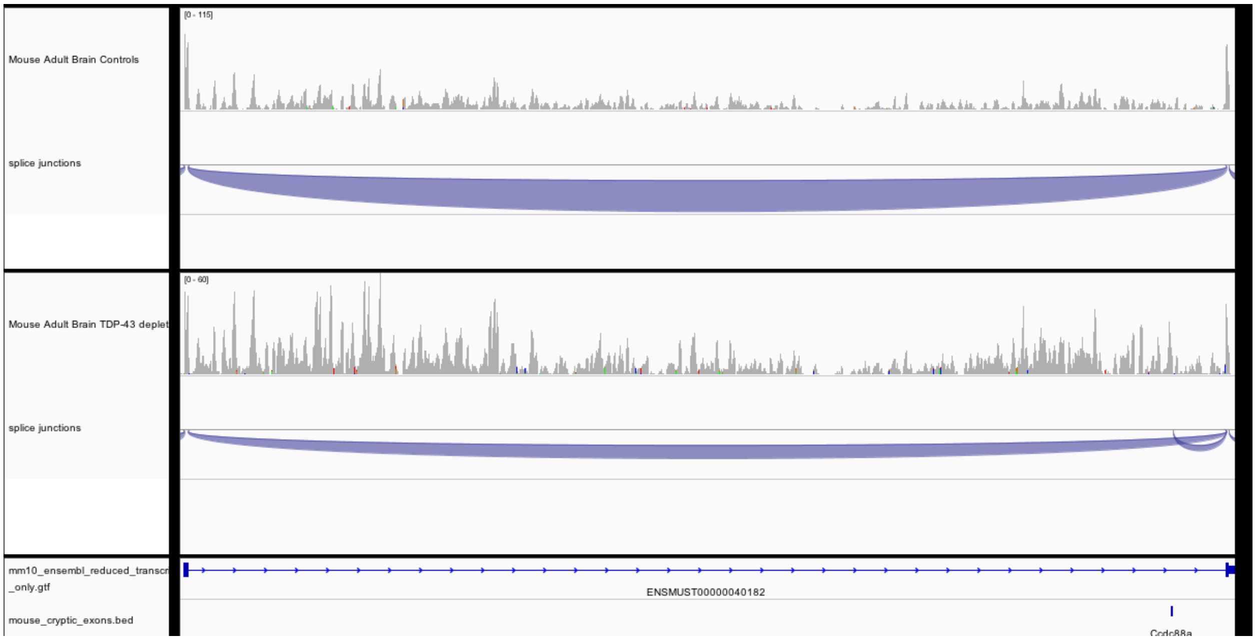

Mouse Embryonic Stem Cells

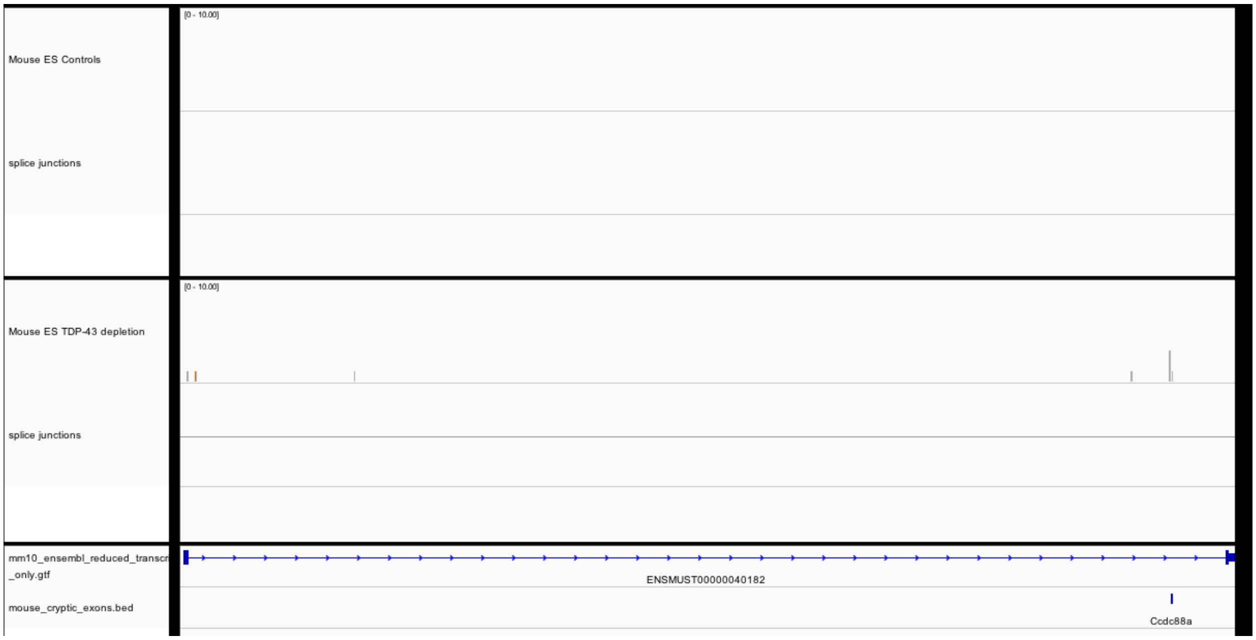

19 Adap1 E014i3

Mouse Adult Brain

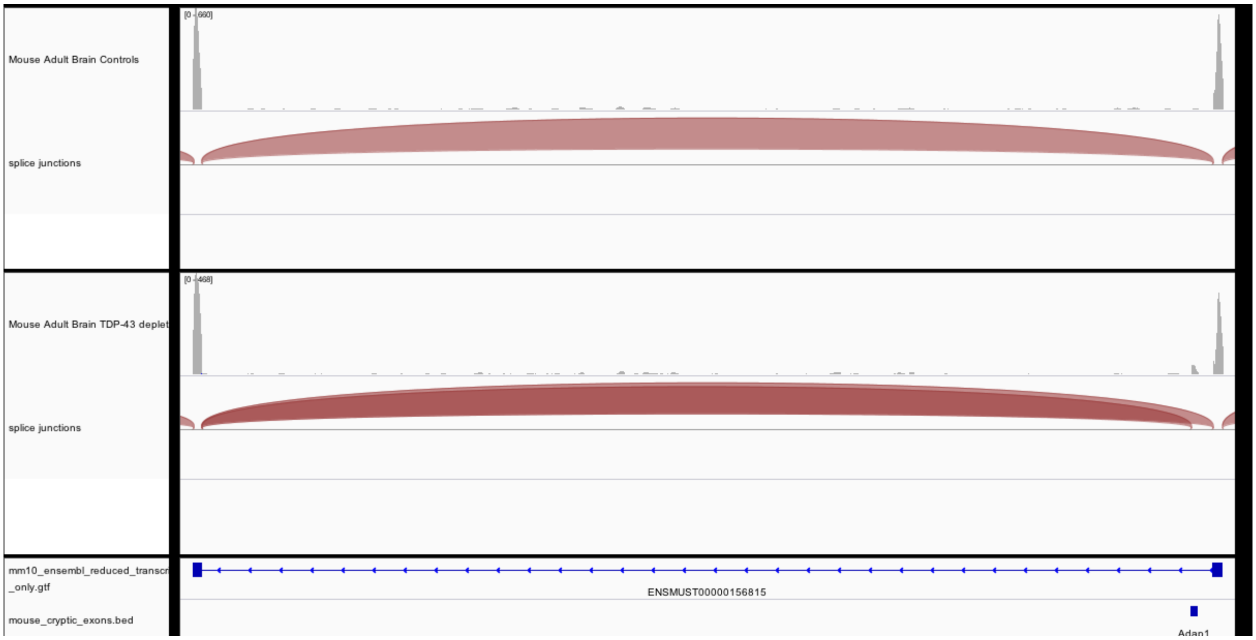

Mouse Embryonic Stem Cells

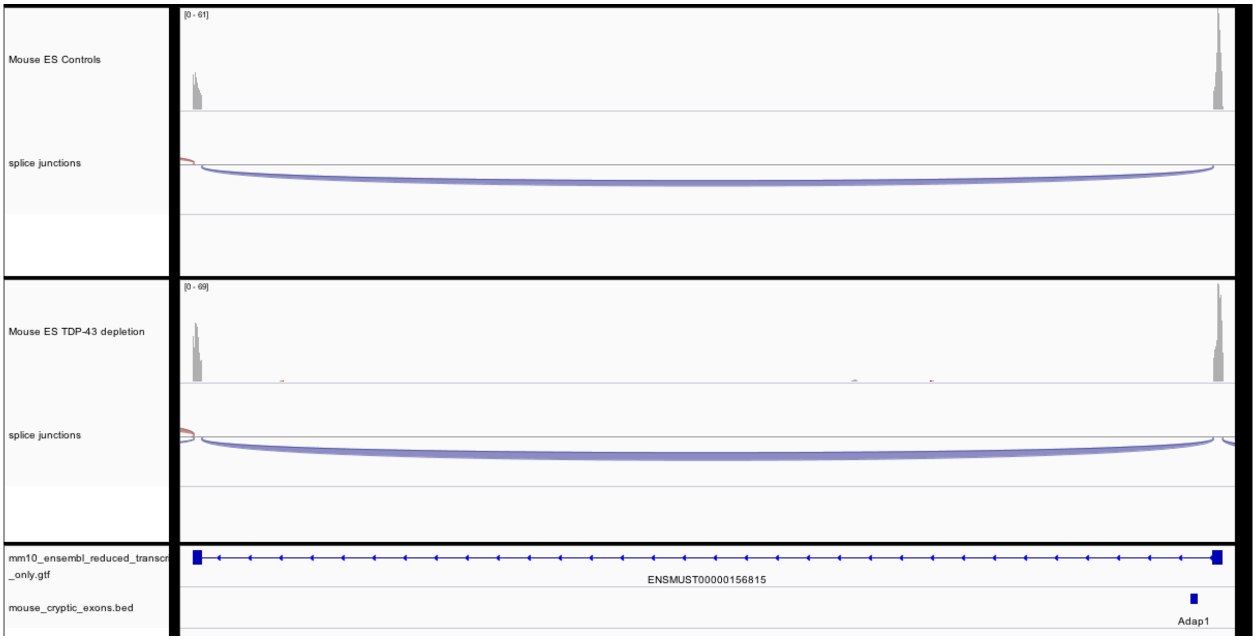

20 Ap3b2 E031i2

Mouse Adult Brain

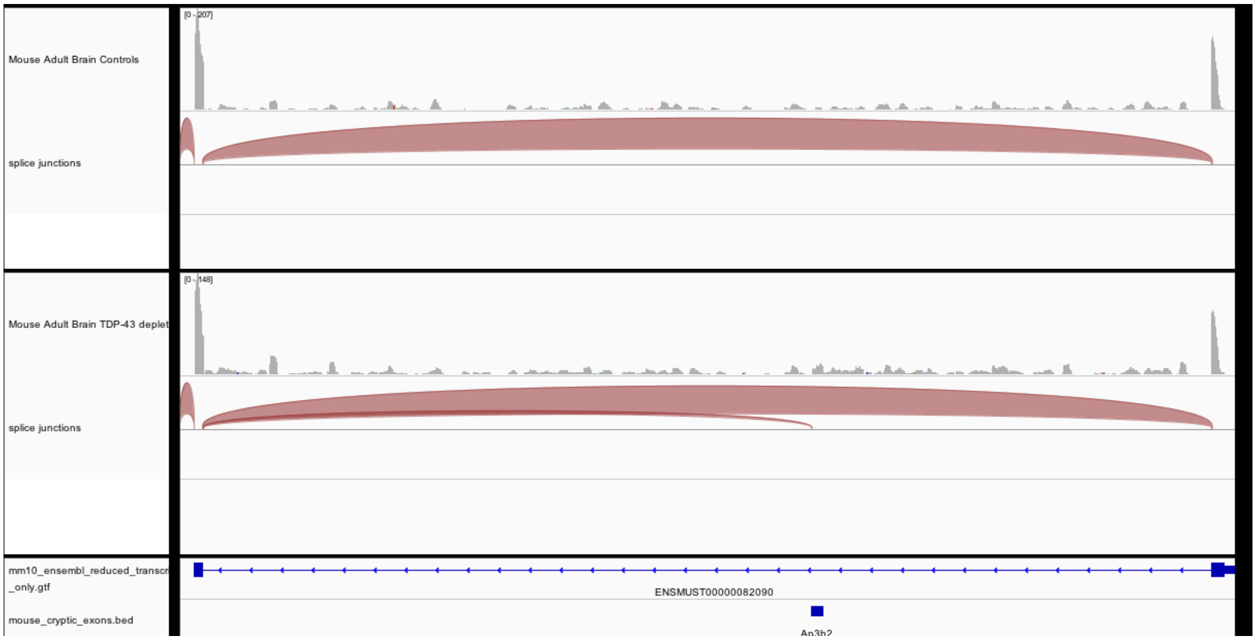

Mouse Embryonic Stem Cells

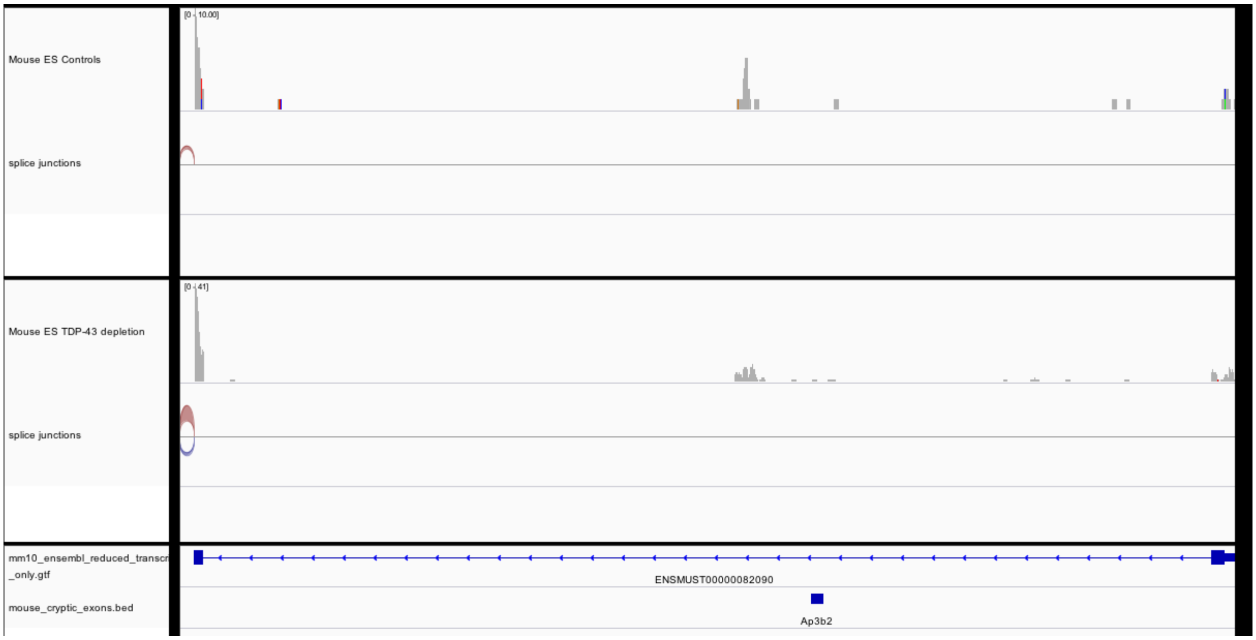

21 Fam73a E015i2

Mouse Adult Brain

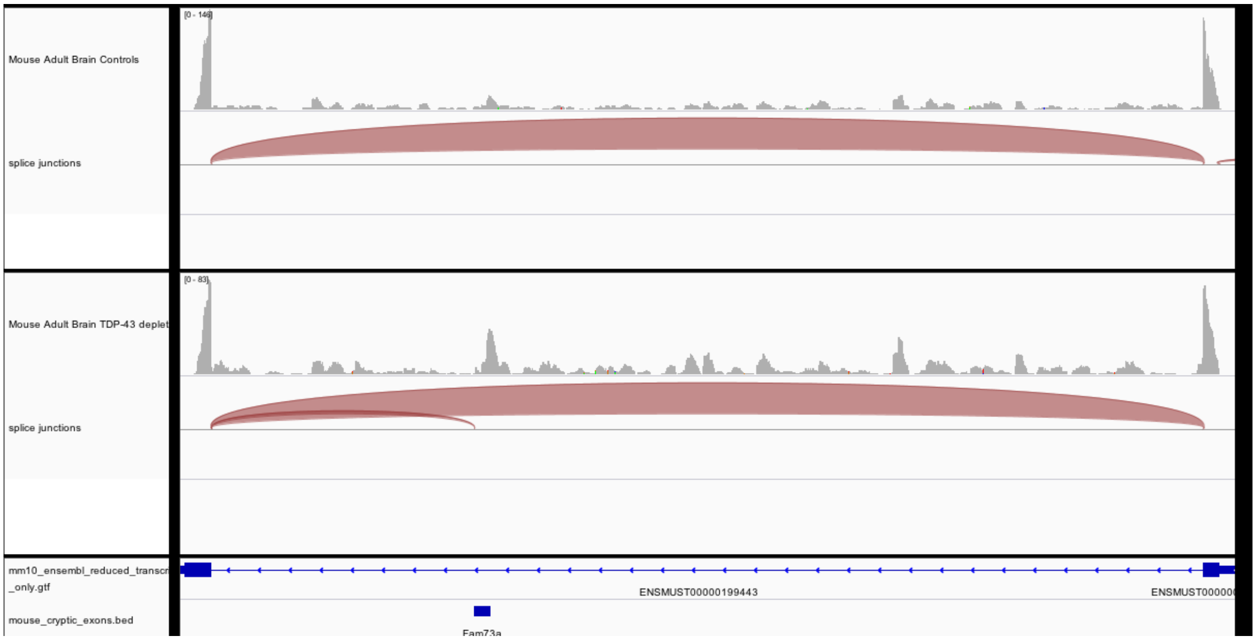

Mouse Embryonic Stem Cells

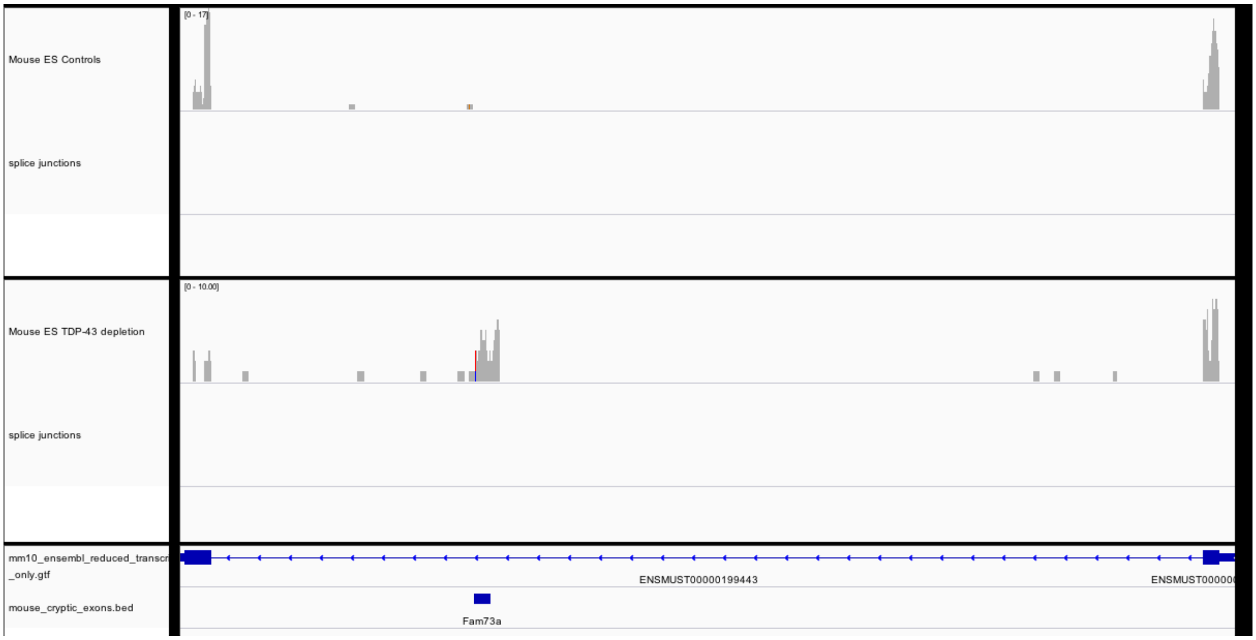

22 Ptprn2 E019i11

Mouse Adult Brain

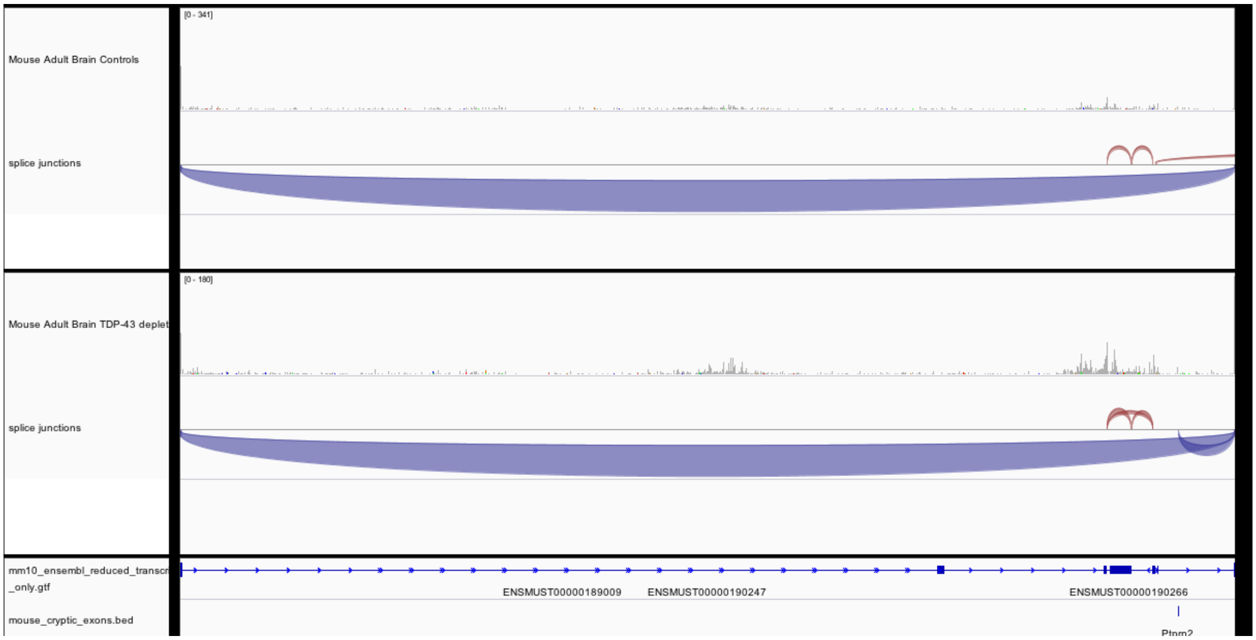

Mouse Embryonic Stem Cells

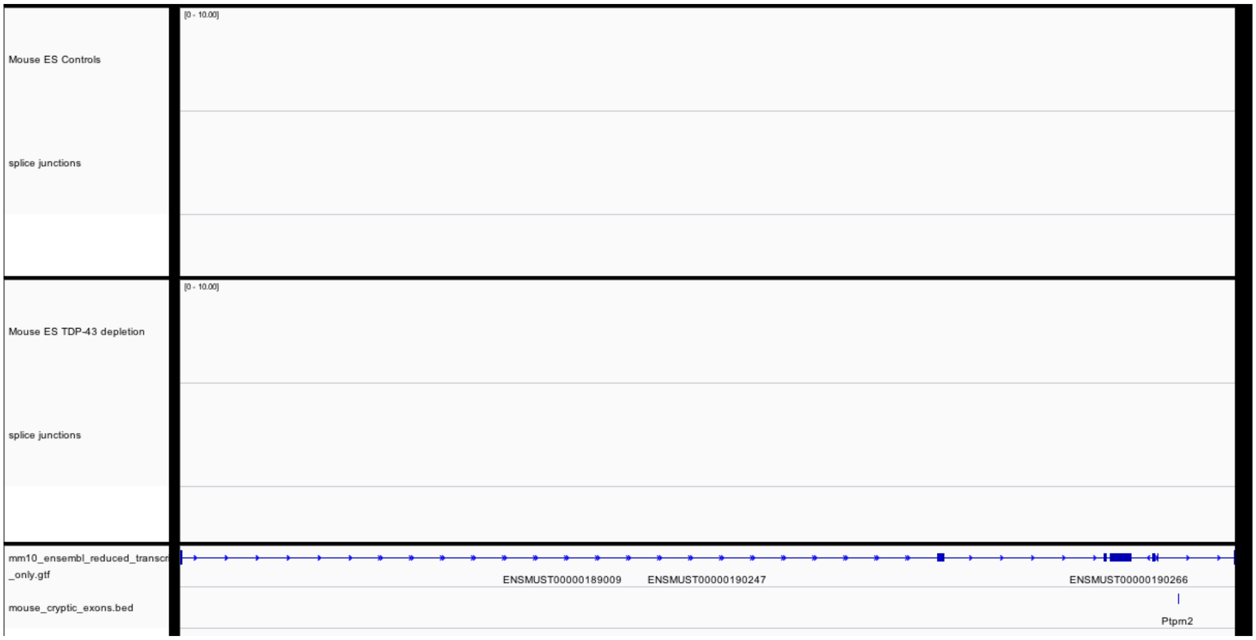

23 Dlg2 E004i17

Mouse Adult Brain

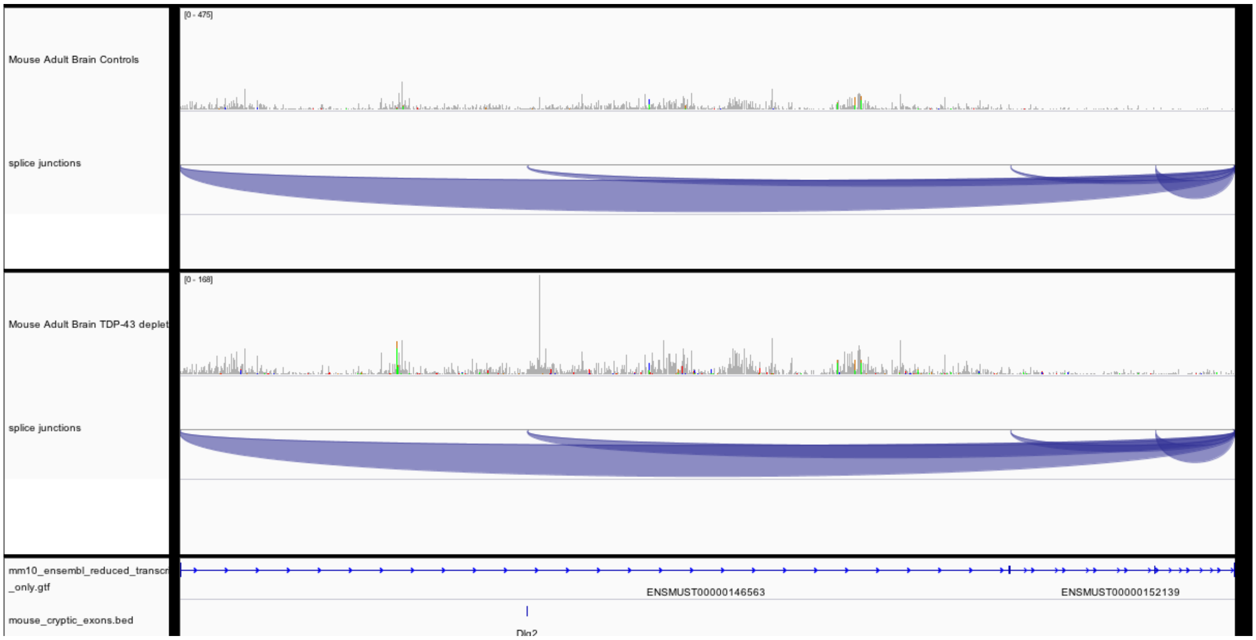

Mouse Embryonic Stem Cells

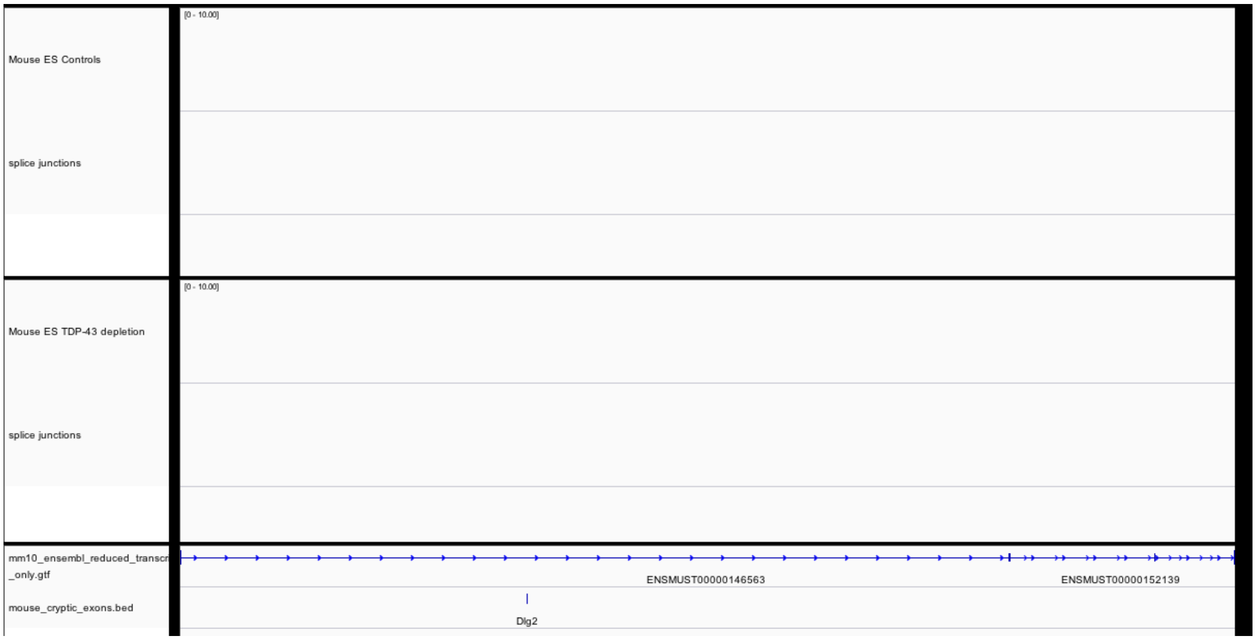

24 Cd200 E003i1

Mouse Adult Brain

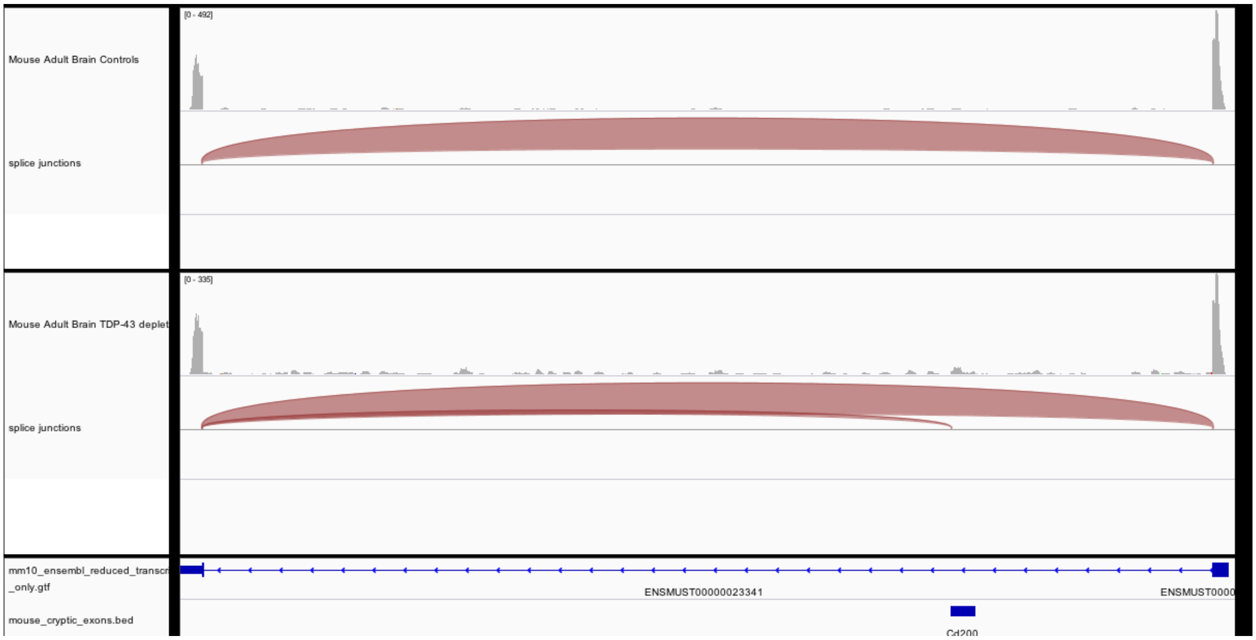

Mouse Embryonic Stem Cells

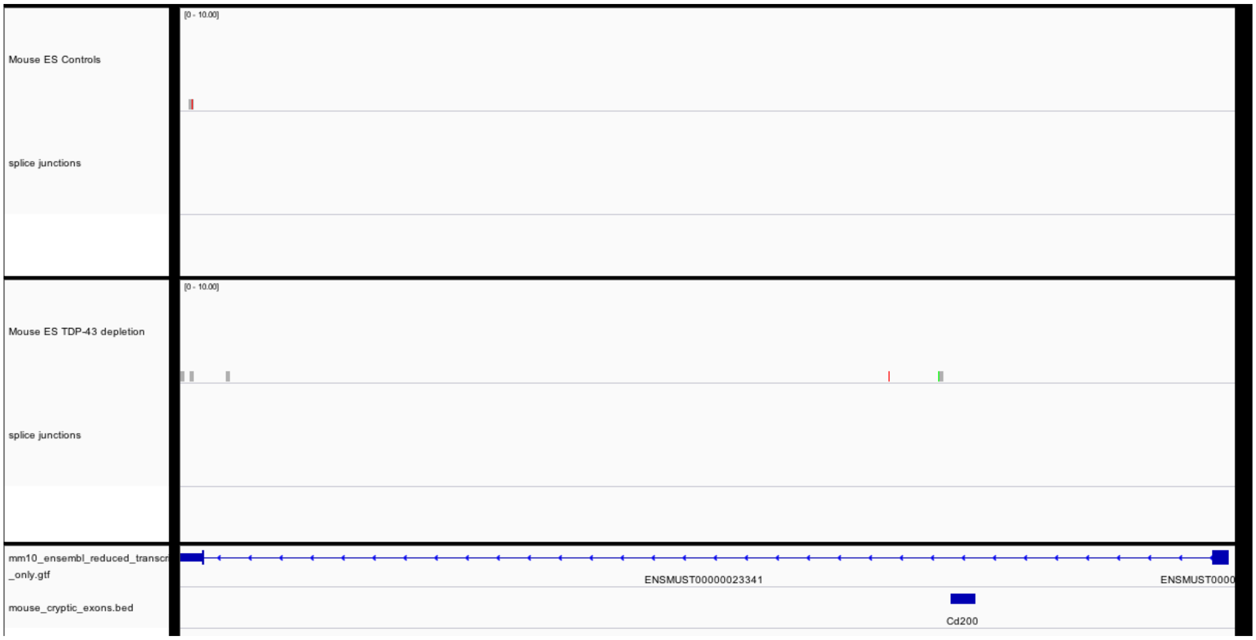

25 Dock4 E049i1

Mouse Adult Brain

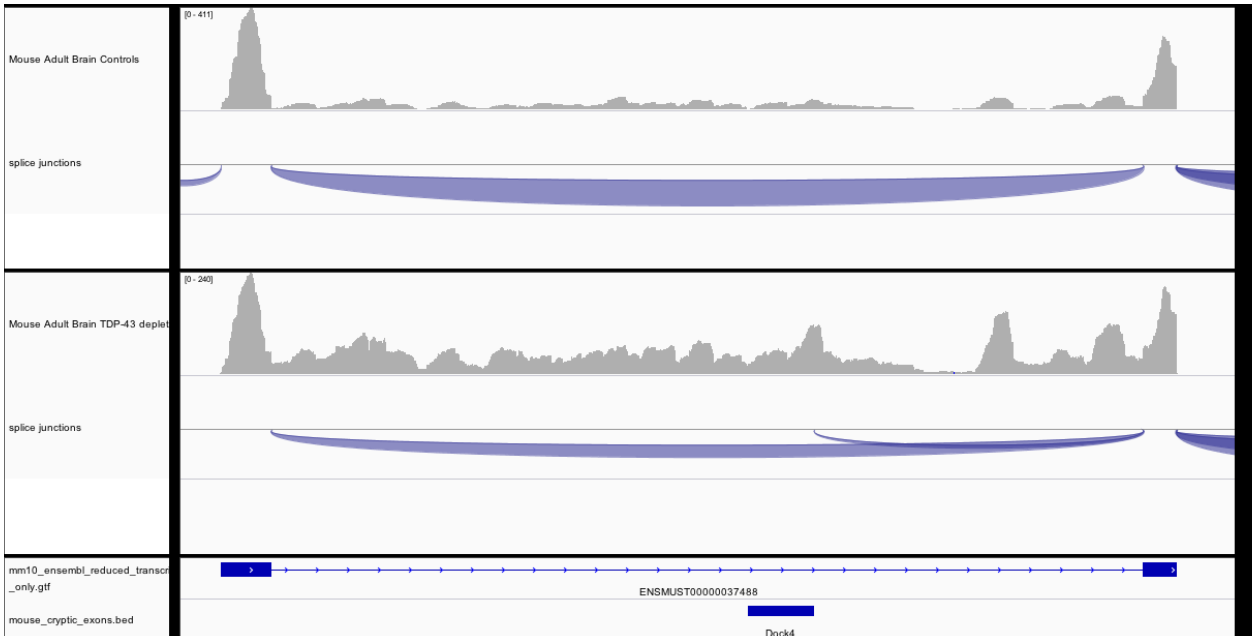

Mouse Embryonic Stem Cells

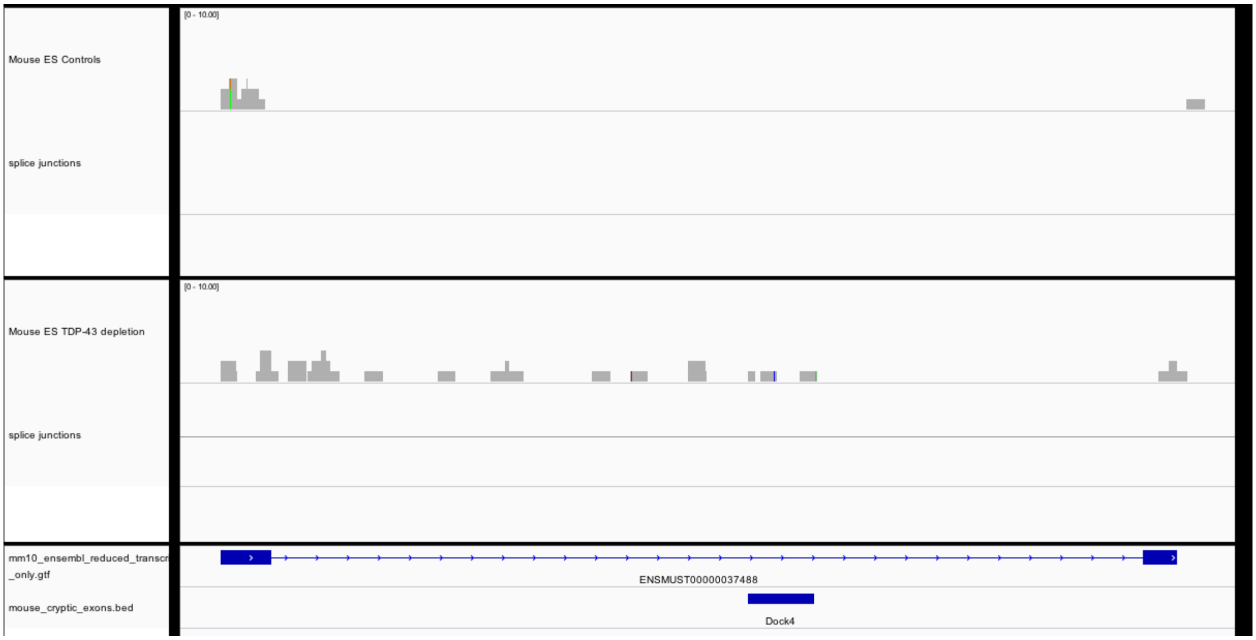

26 C4b E032i1

Mouse Adult Brain

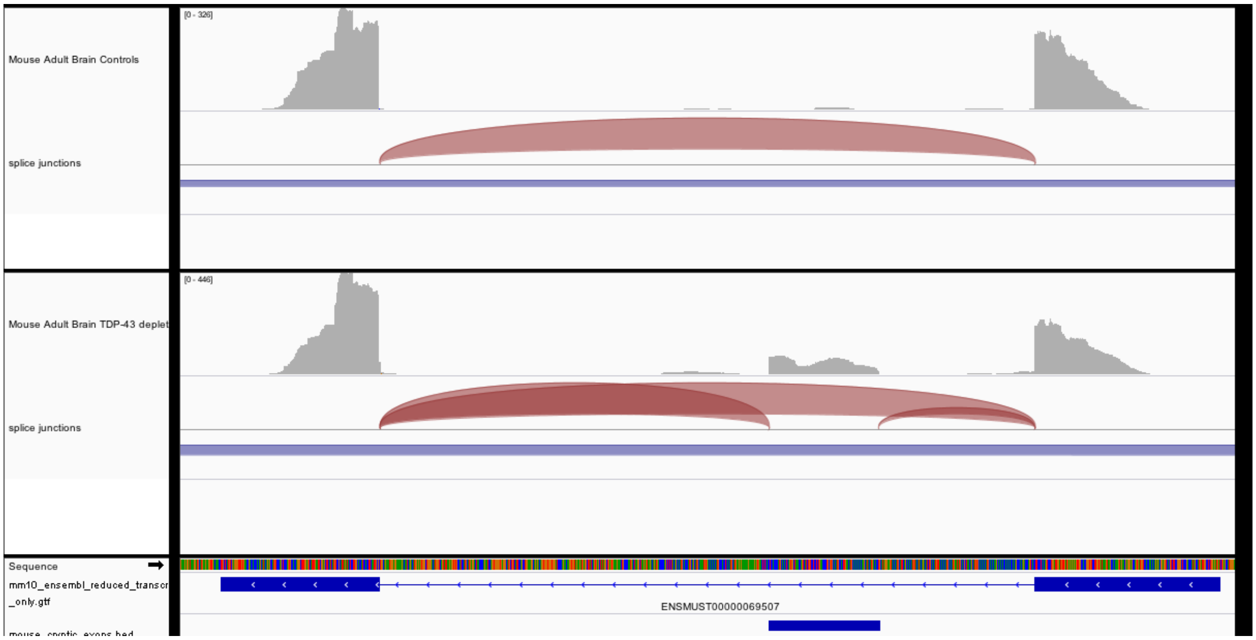

Mouse Embryonic Stem Cells

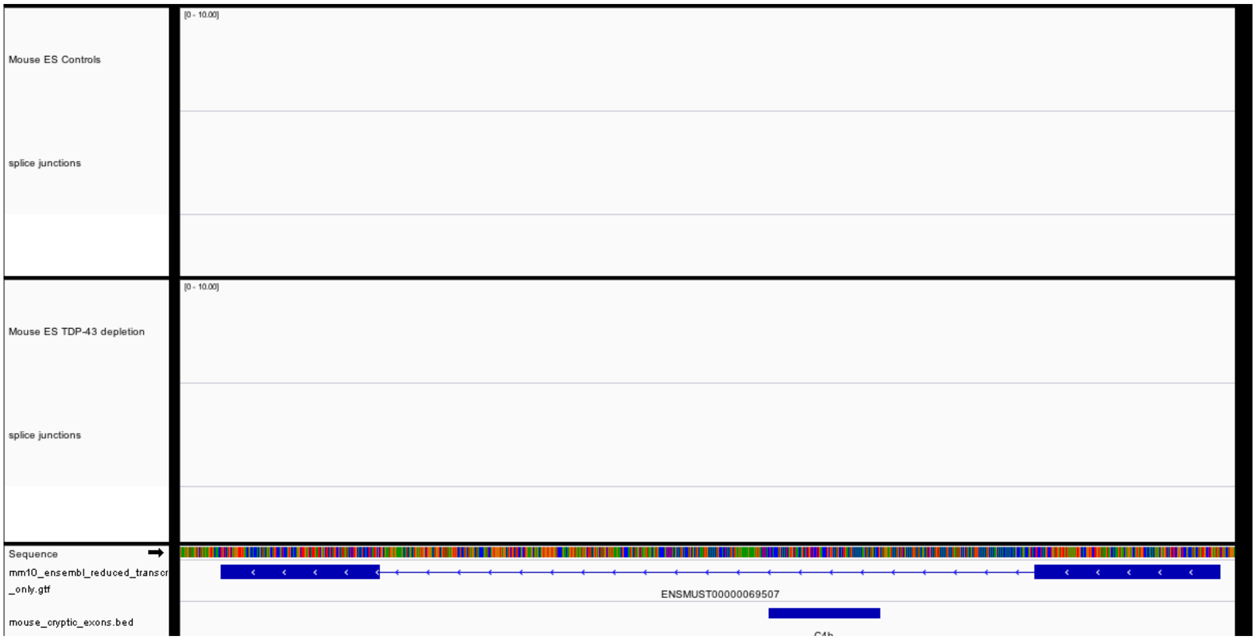

27 Camk1g E017i2

Mouse Adult Brain

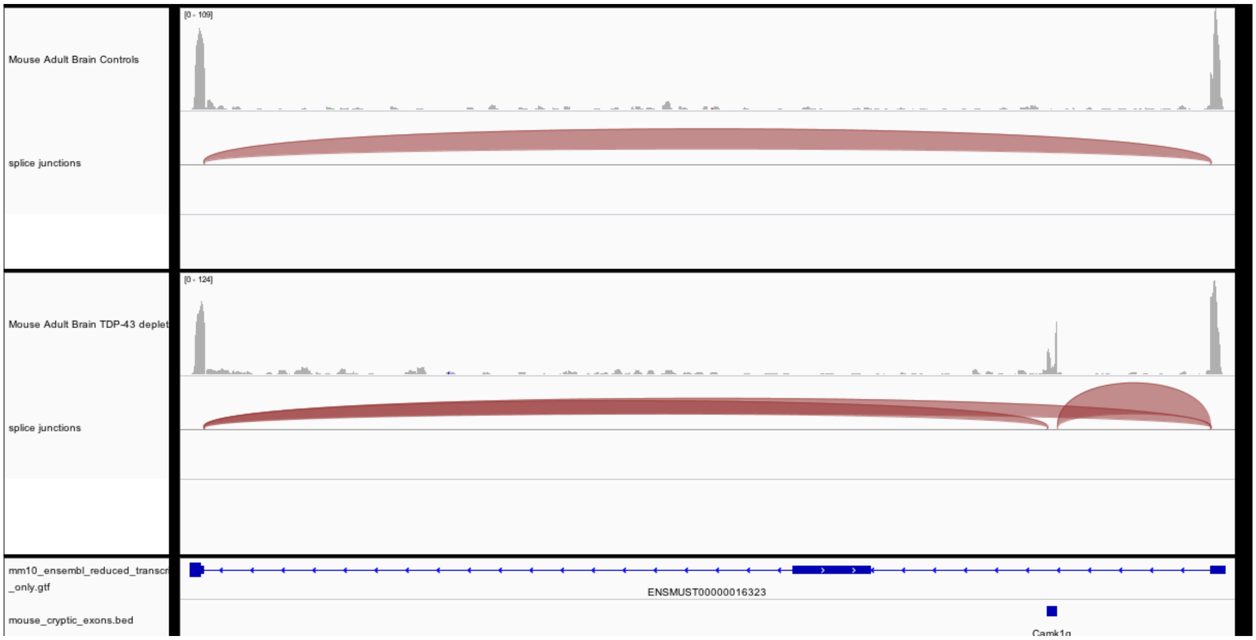

Mouse Embryonic Stem Cells

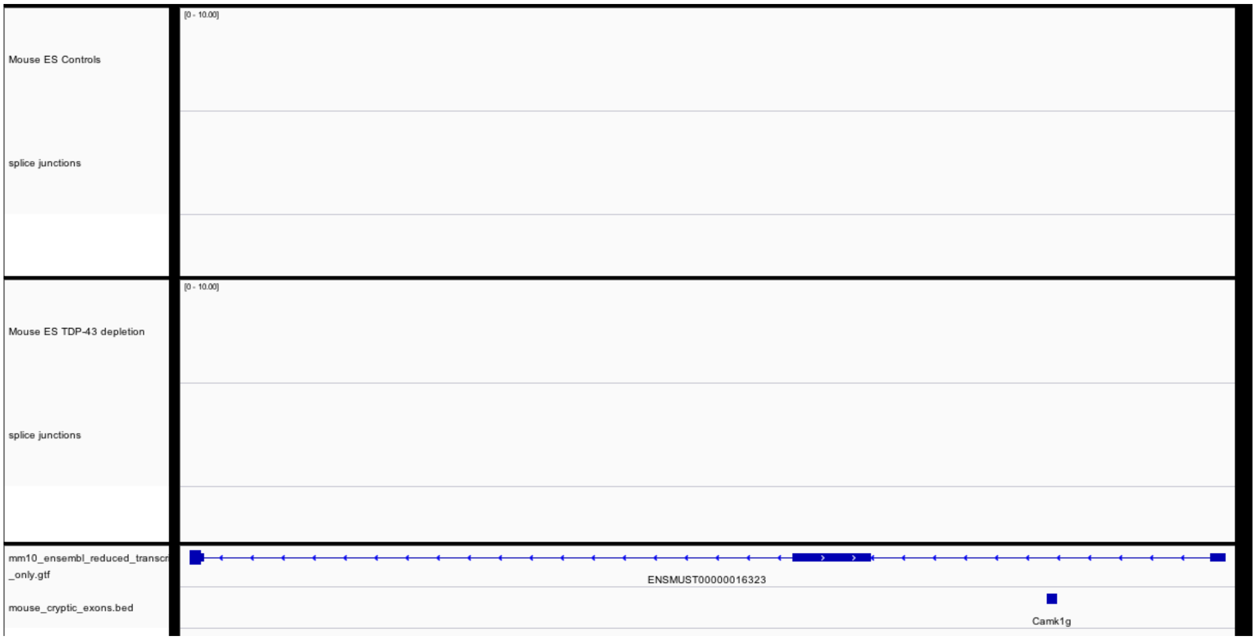

28 Reep3 E001i1

Mouse Adult Brain

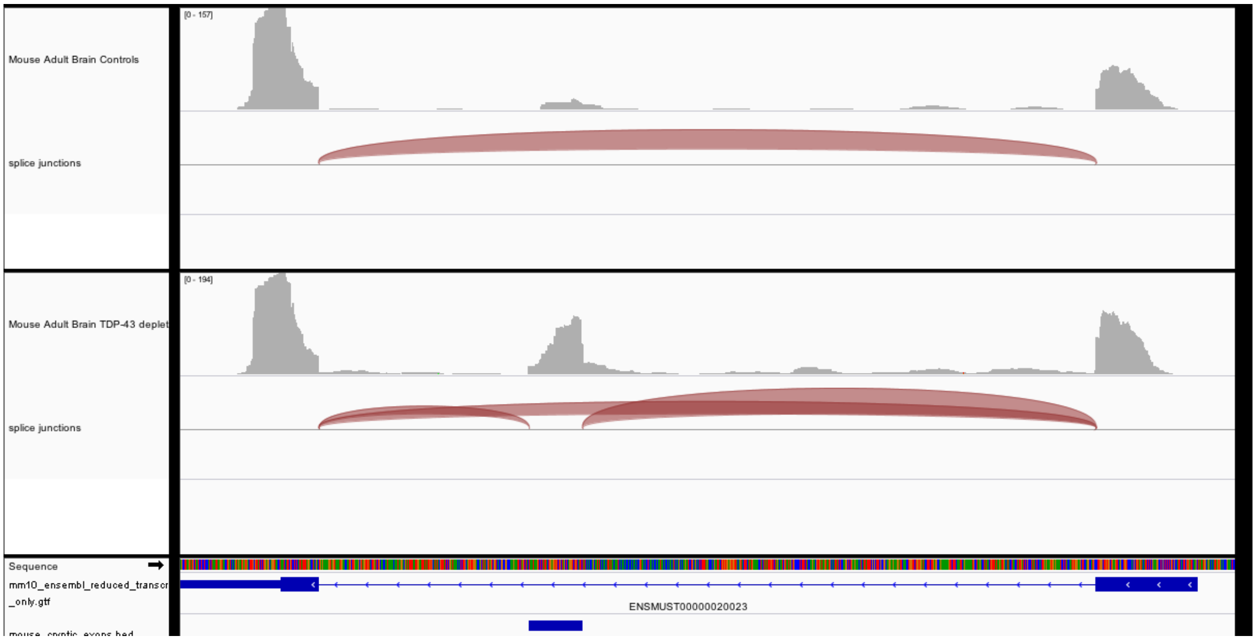

Mouse Embryonic Stem Cells

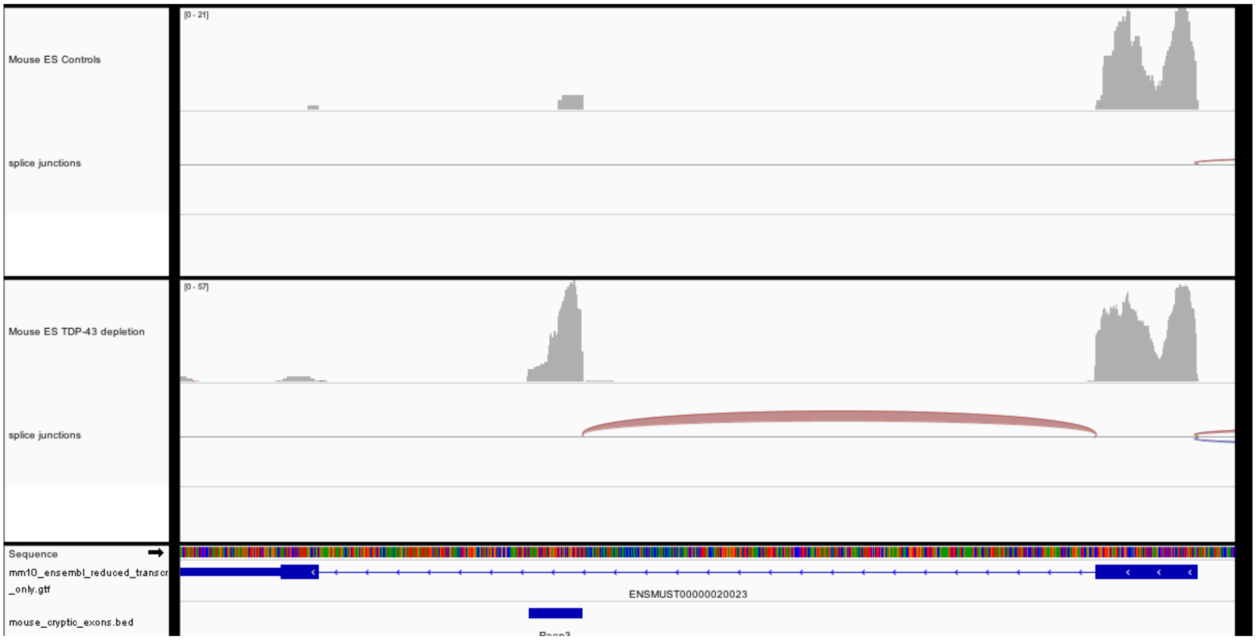

29 Drosha E009i1

Mouse Adult Brain

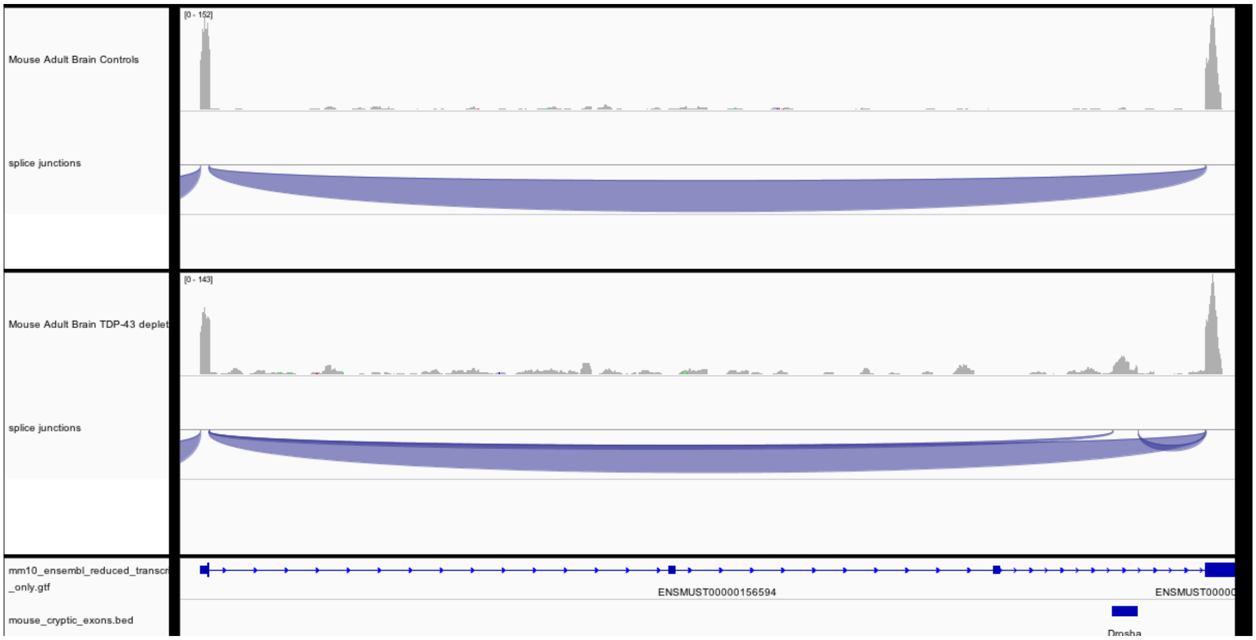

Mouse Embryonic Stem Cells

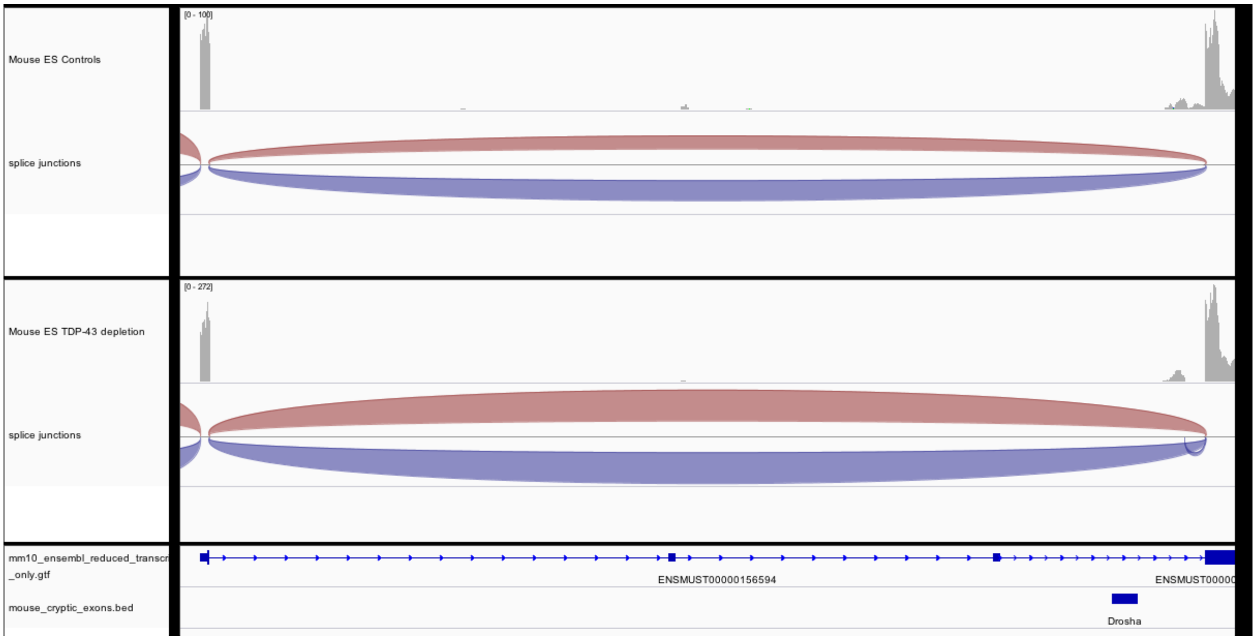

30 Synj2bp+Gm20498 E036i2

Mouse Adult Brain

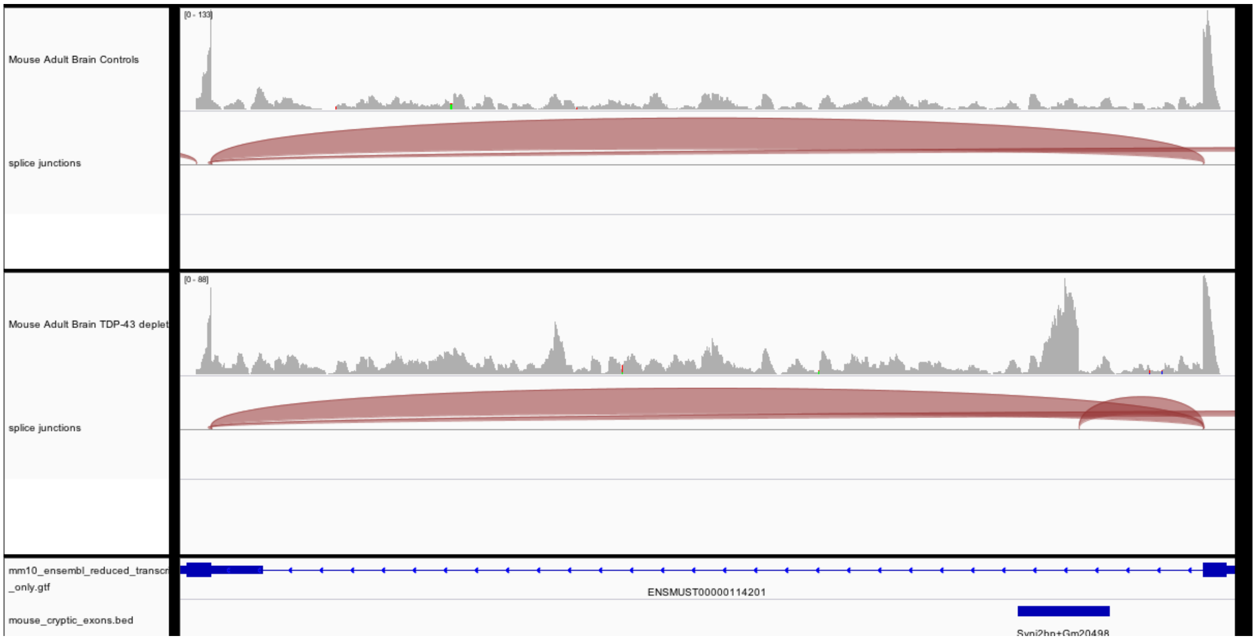

Mouse Embryonic Stem Cells

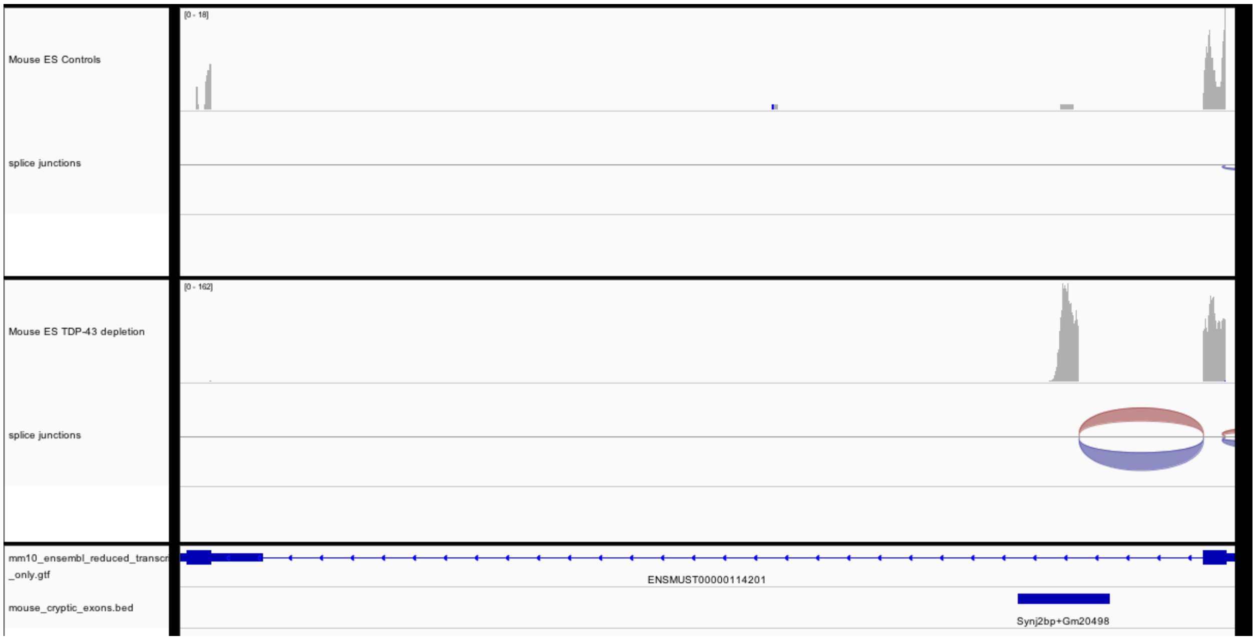

31 Adnp2 E002i1

Mouse Adult Brain

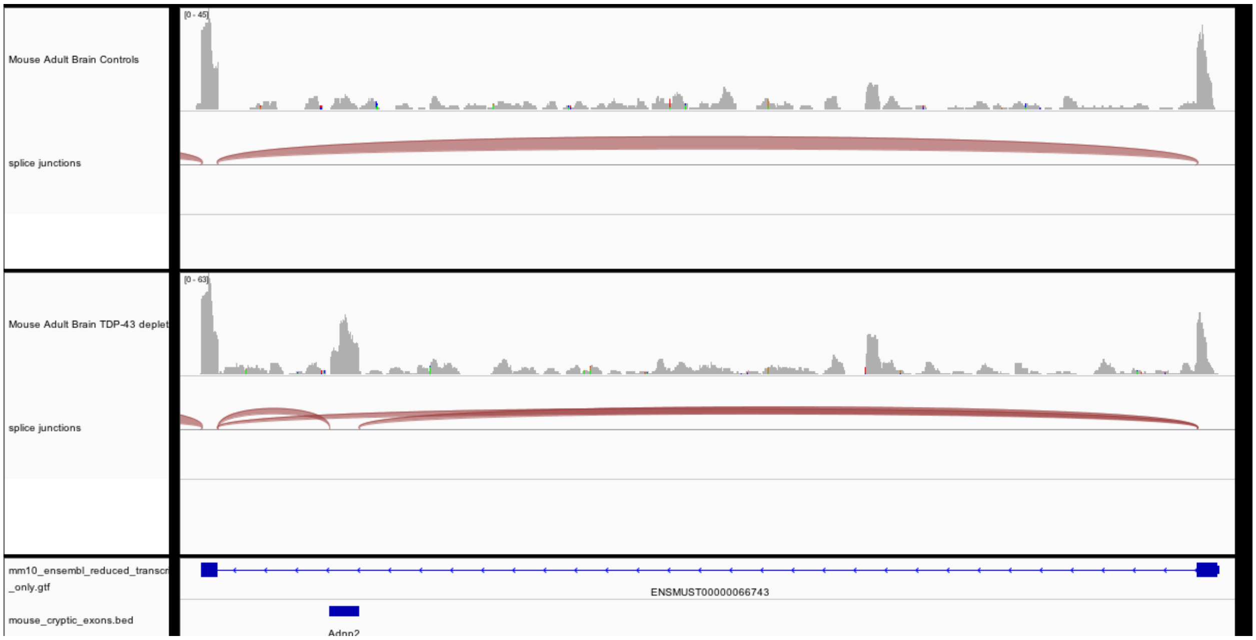

Mouse Embryonic Stem Cells

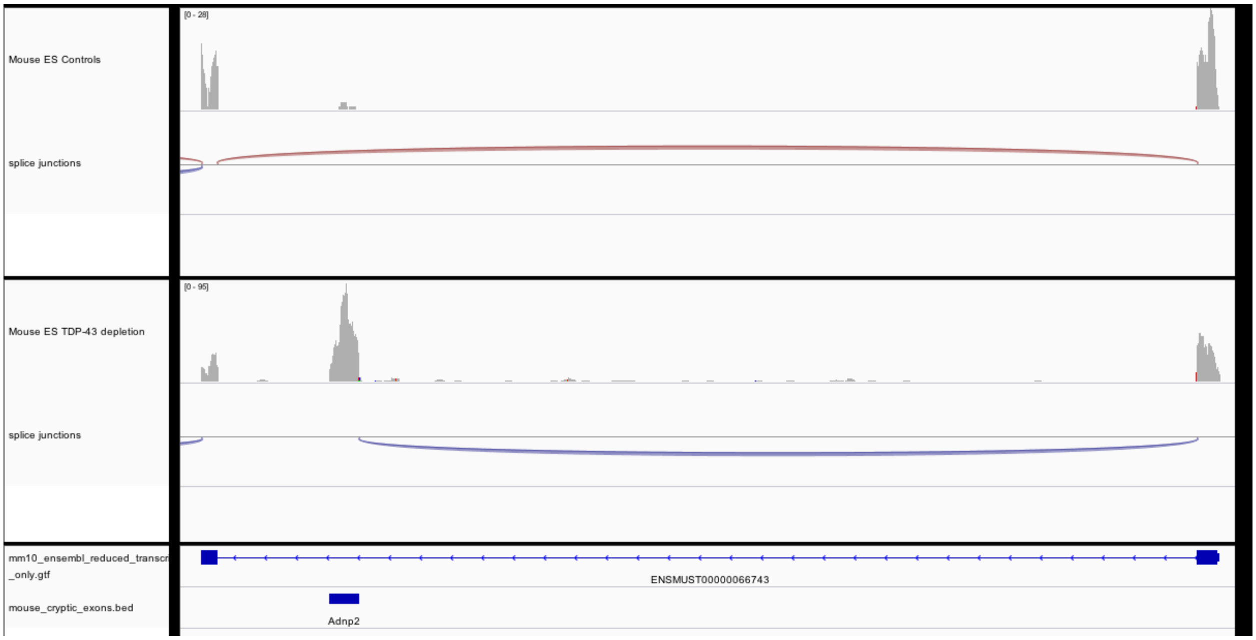

32 Adipor2 E010i2

Mouse Adult Brain

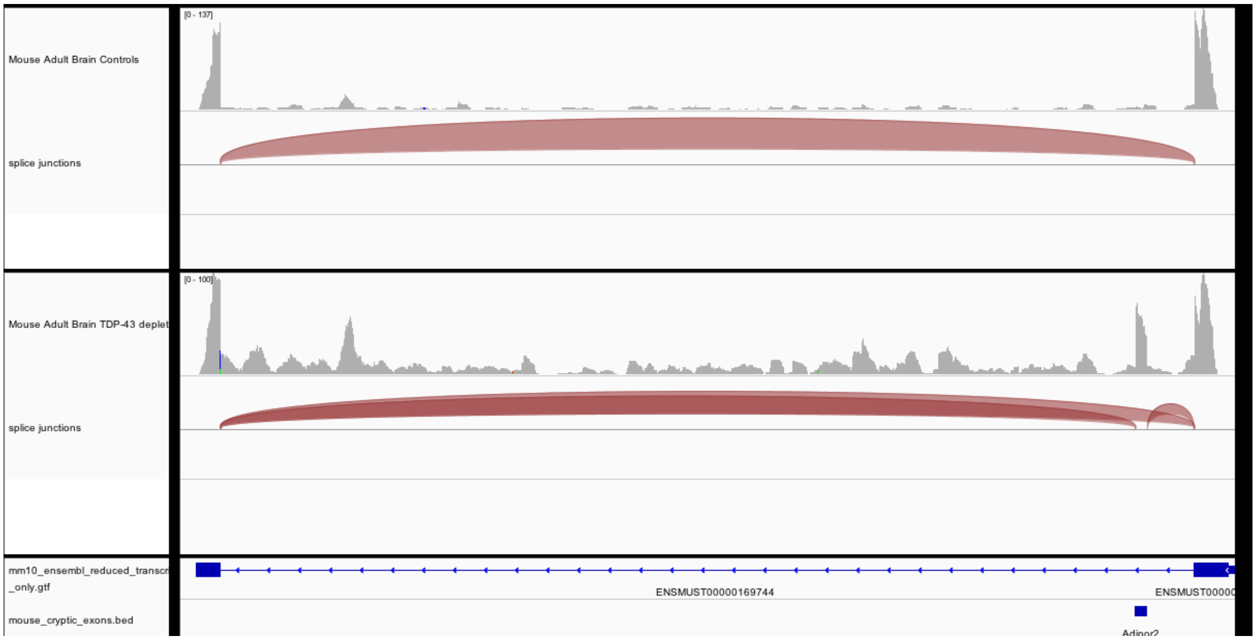

Mouse Embryonic Stem Cells

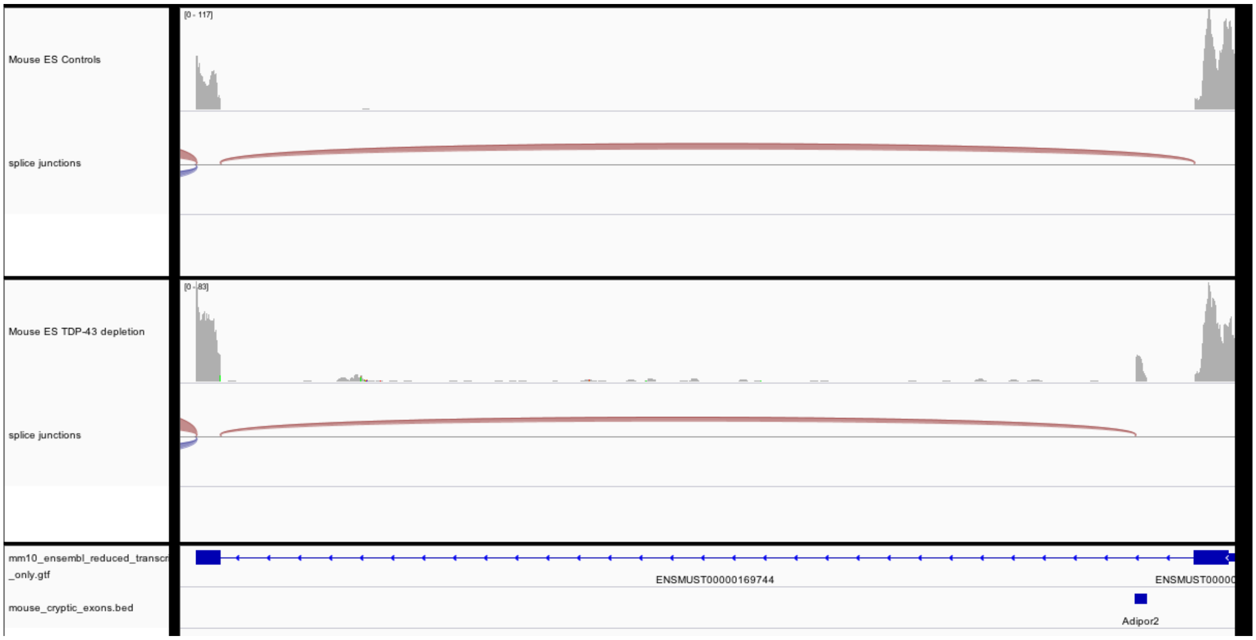

33 Cdh22 E013i2

Mouse Adult Brain

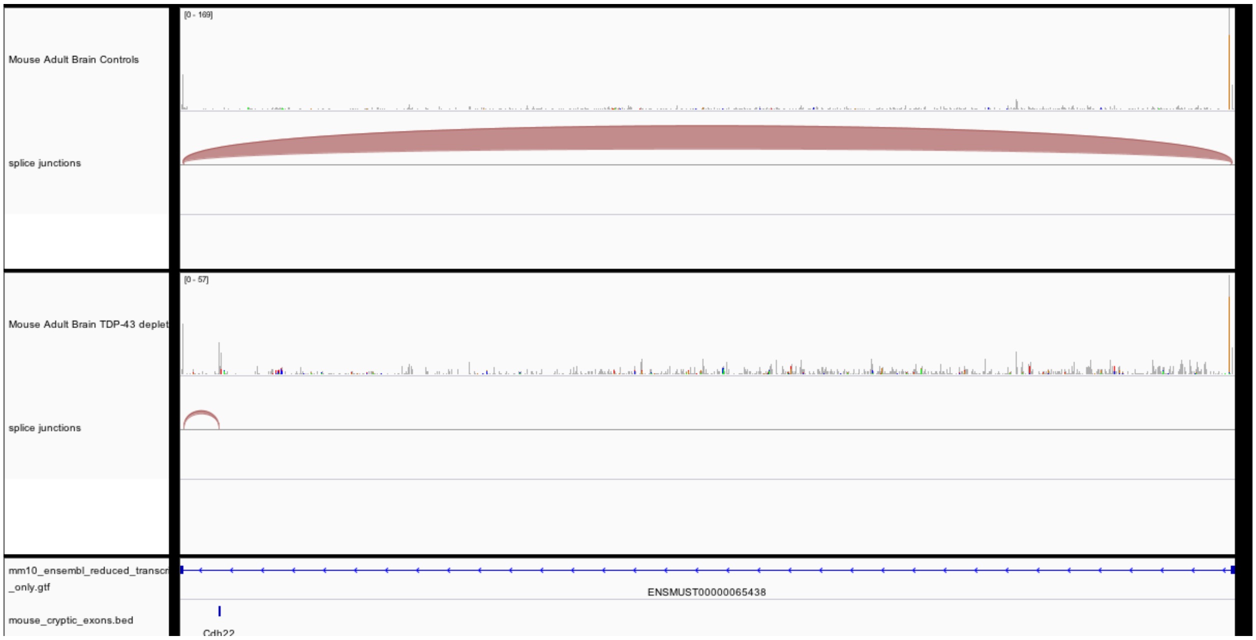

Mouse Embryonic Stem Cells

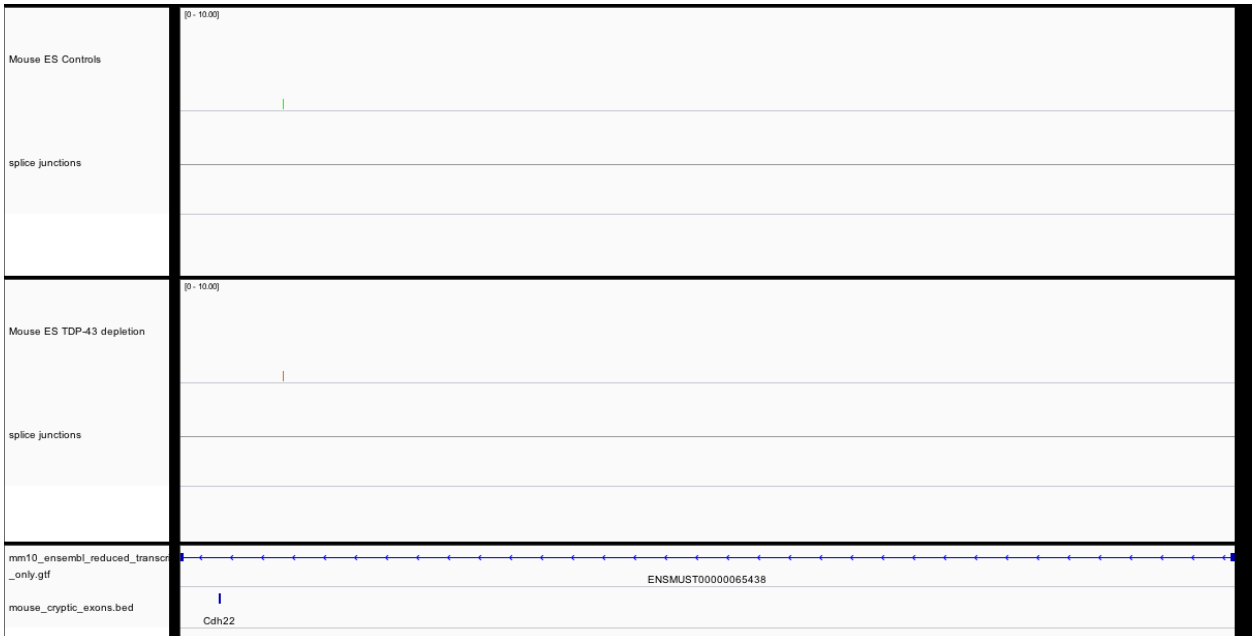

34 Ermn E001i1

Mouse Adult Brain

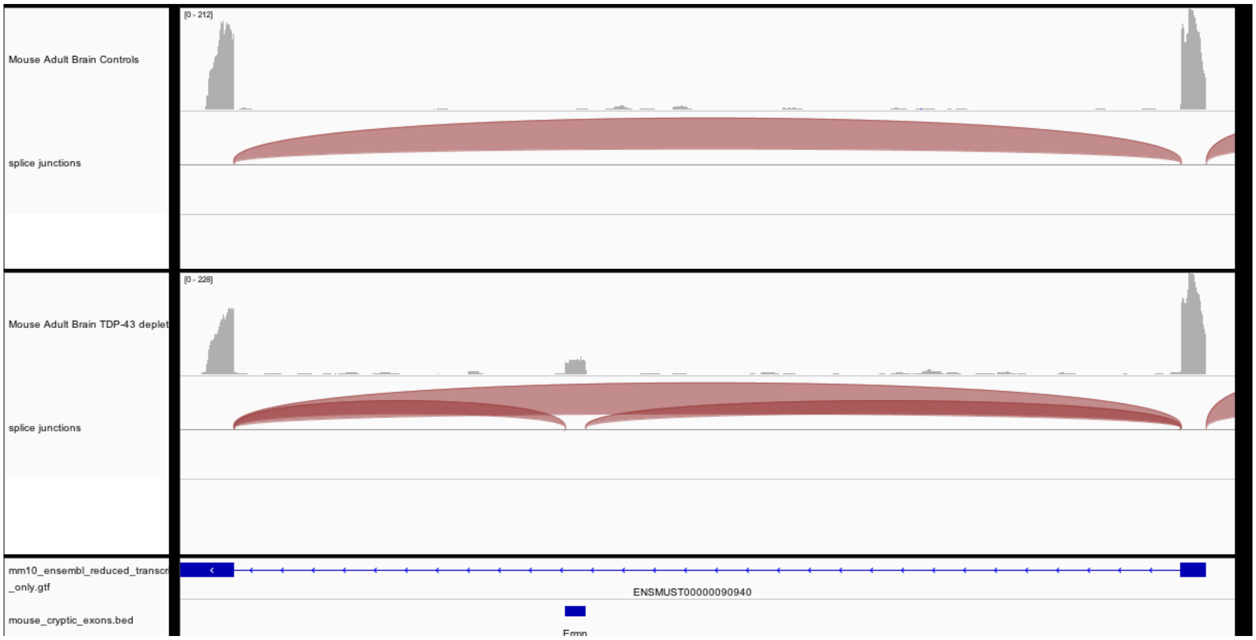

Mouse Embryonic Stem Cells

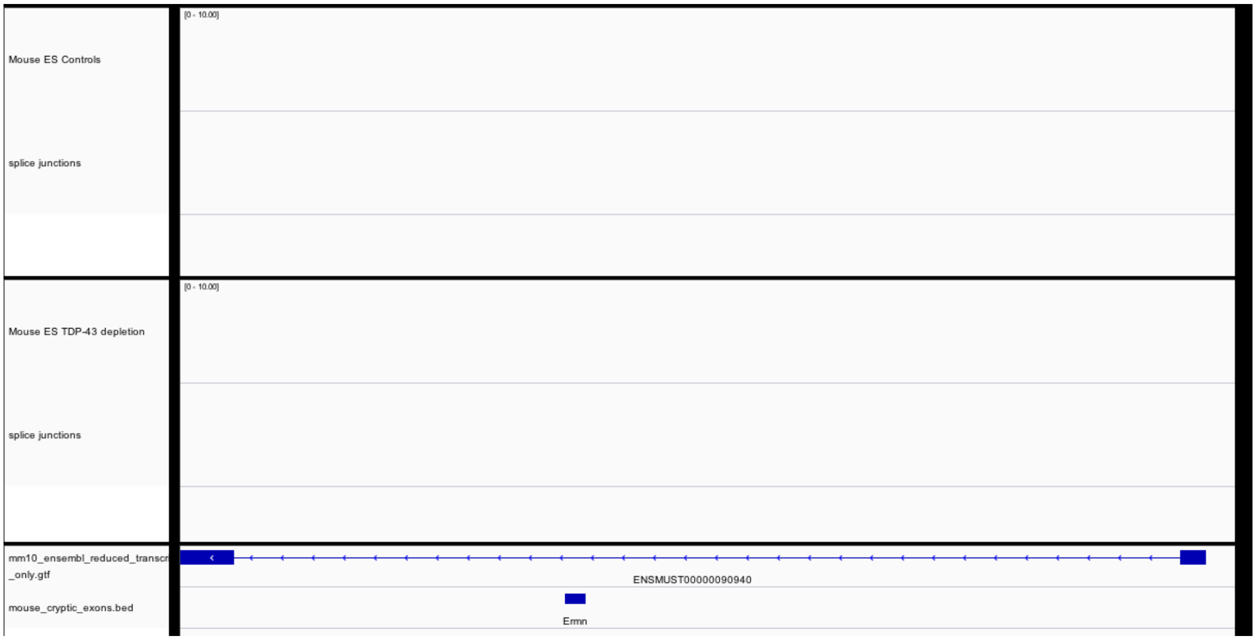

35 Ift81 E003i1

Mouse Adult Brain

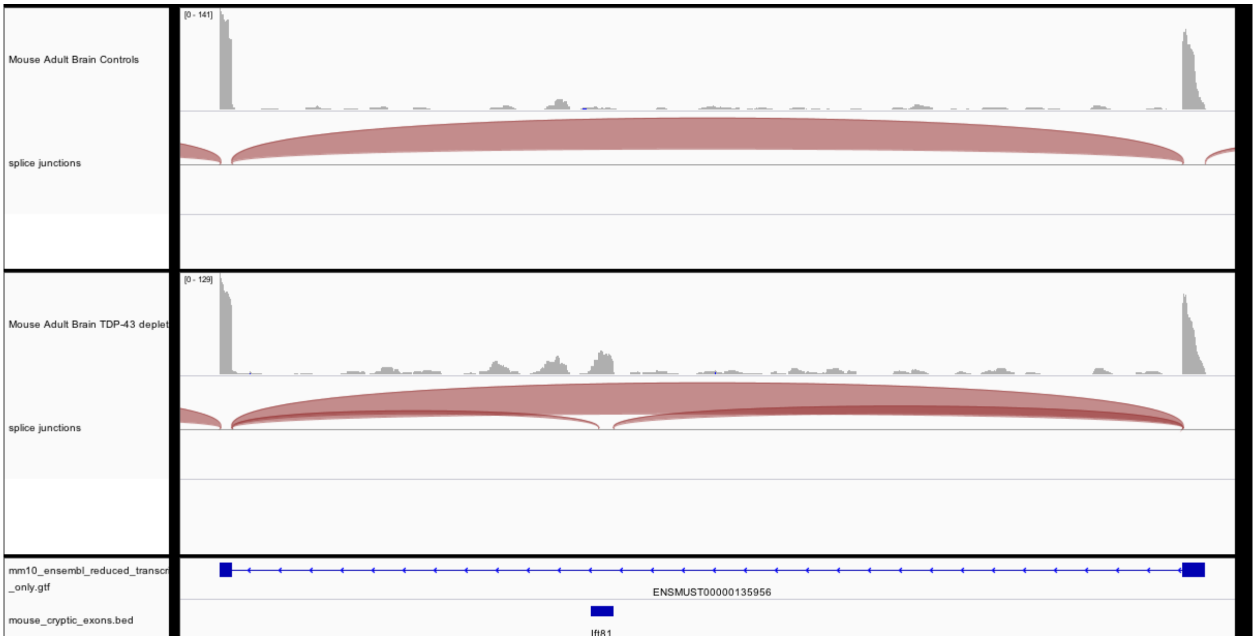

Mouse Embryonic Stem Cells

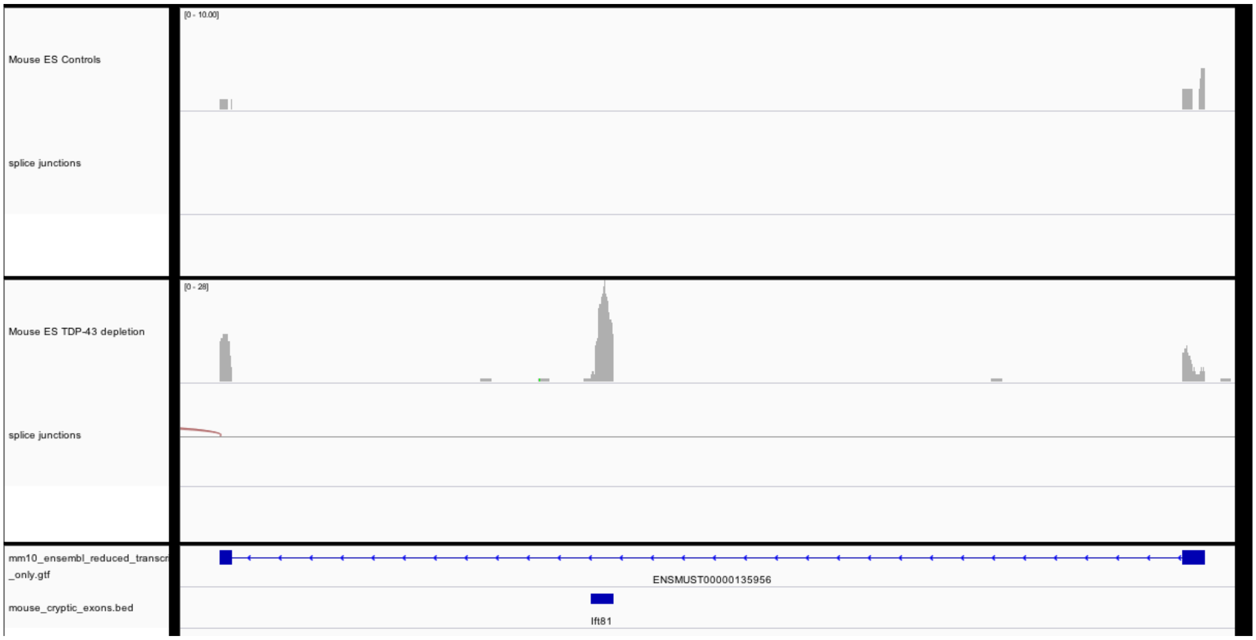

36 Vps13d E043i1

Mouse Adult Brain

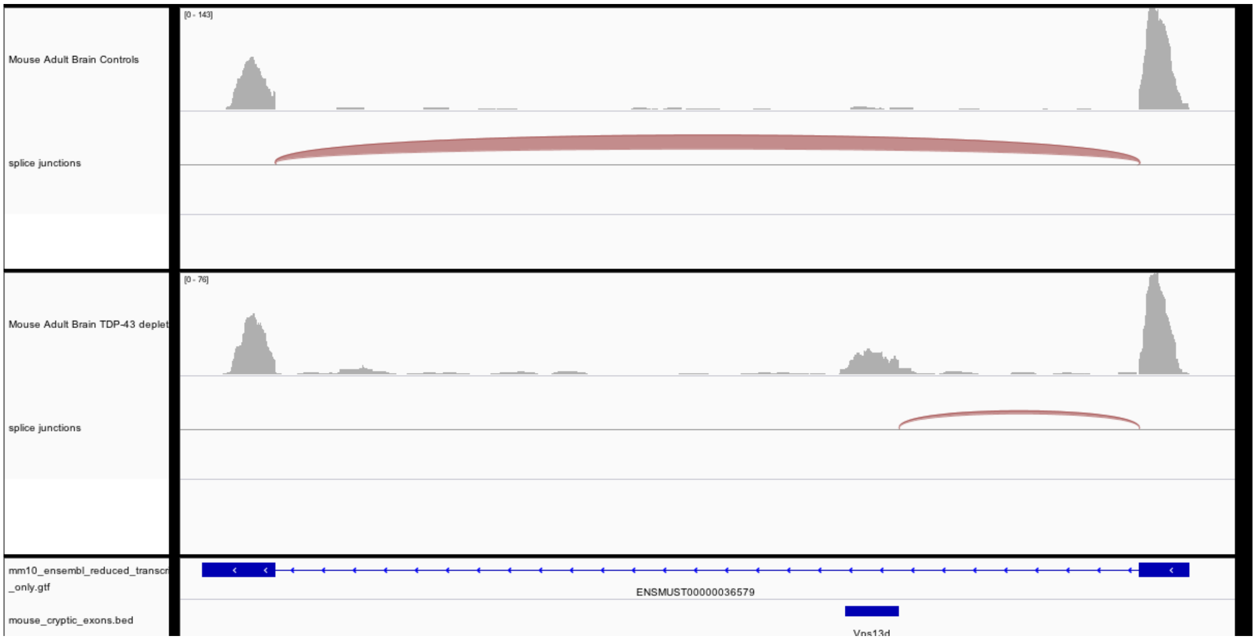

Mouse Embryonic Stem Cells

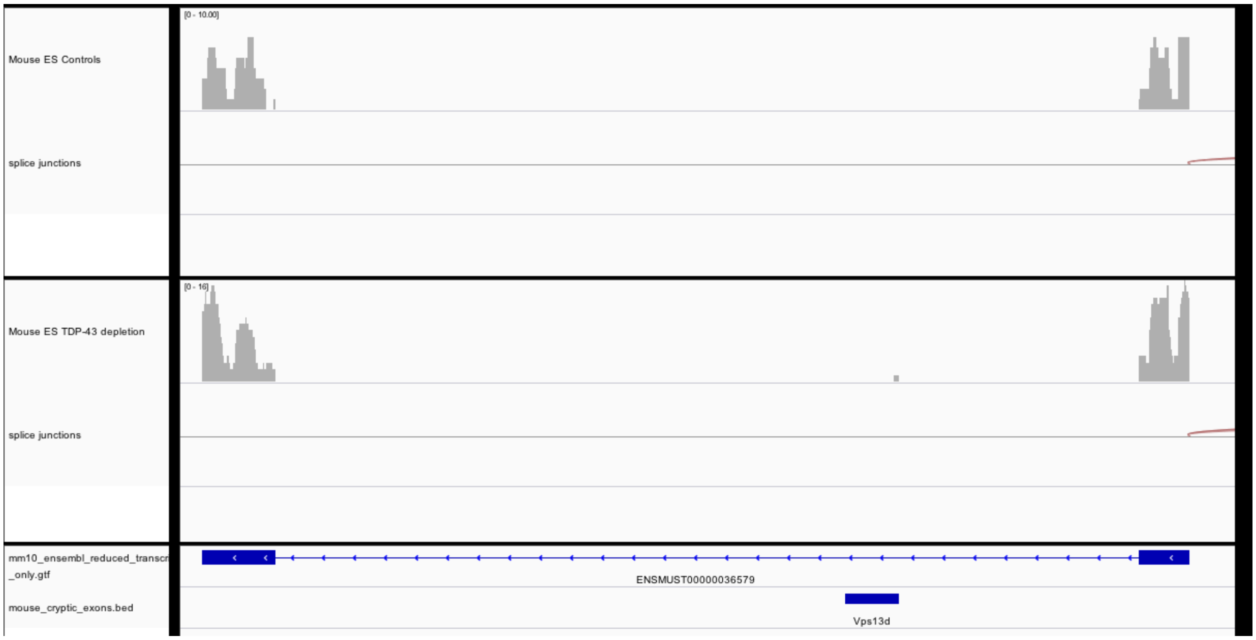

37 Ube2d1 E010i1

Mouse Adult Brain

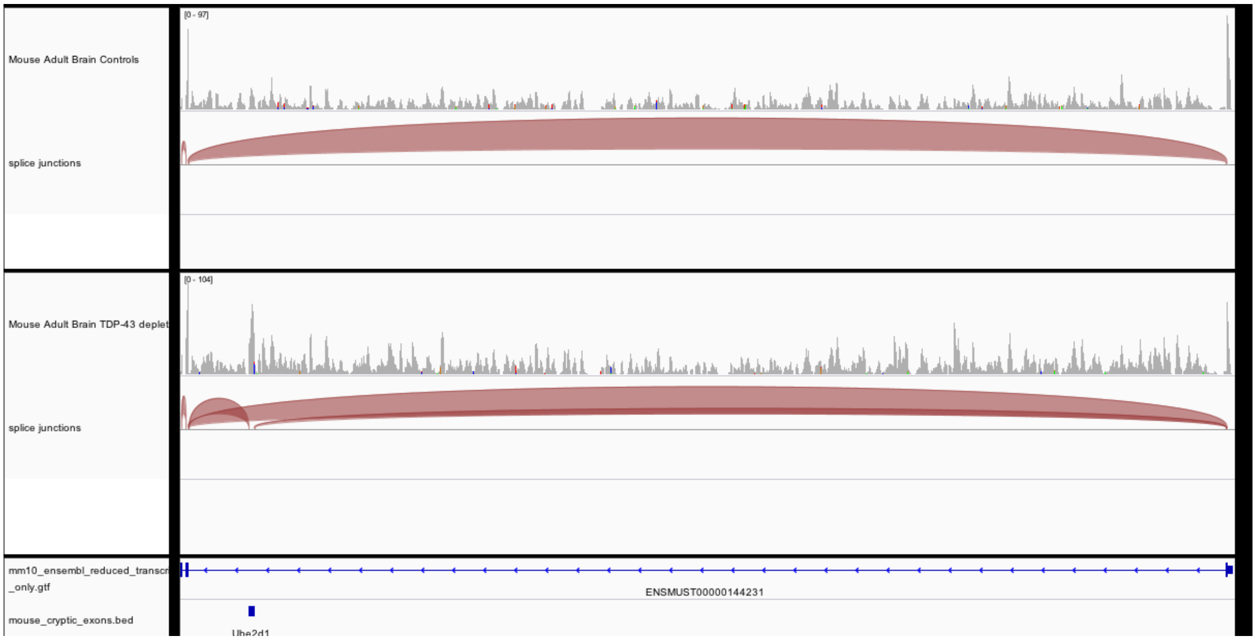

Mouse Embryonic Stem Cells

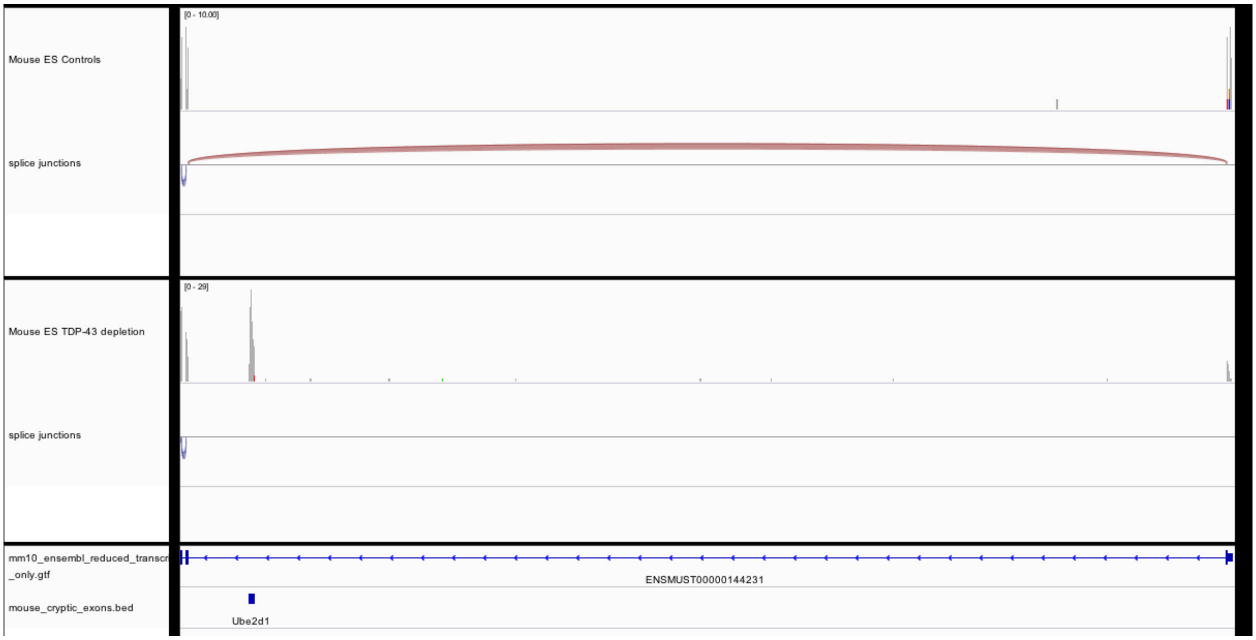

38 Hgsnat E001i1

Mouse Adult Brain

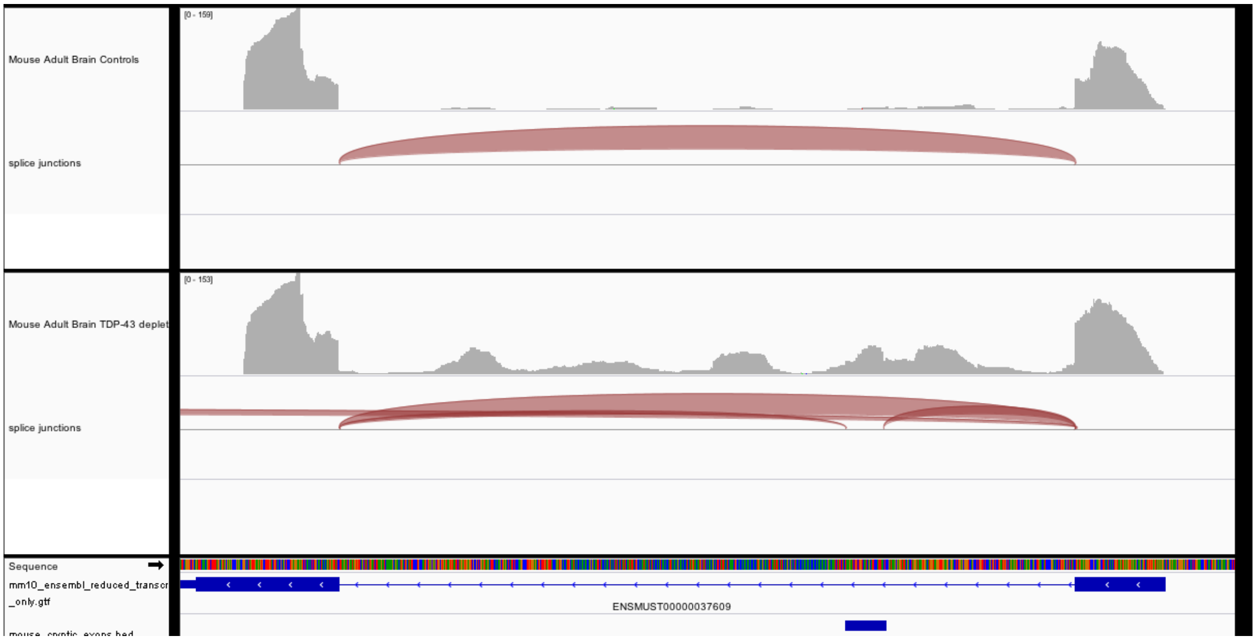

Mouse Embryonic Stem Cells

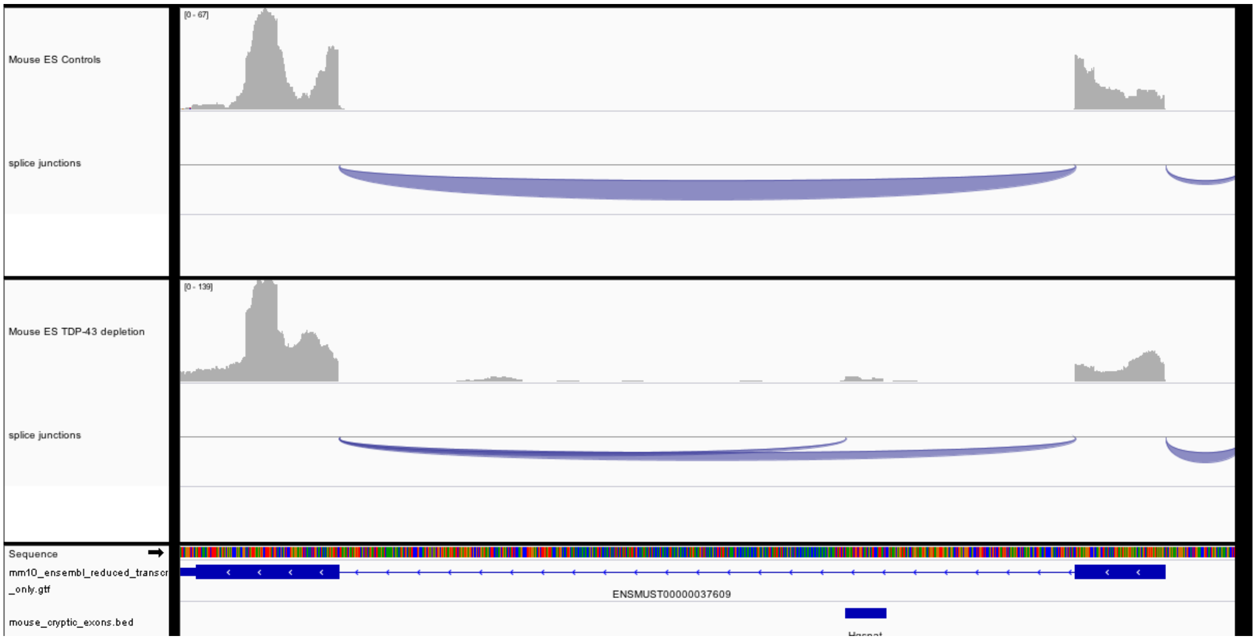

39 Elmod1 E003i2

Mouse Adult Brain

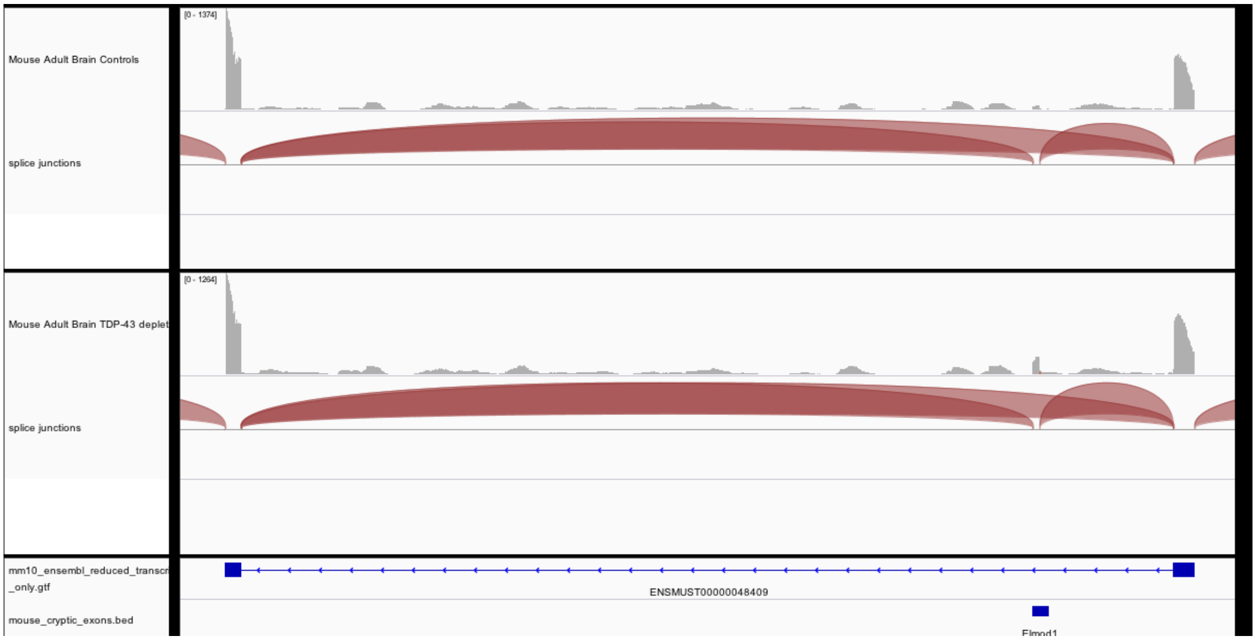

Mouse Embryonic Stem Cells

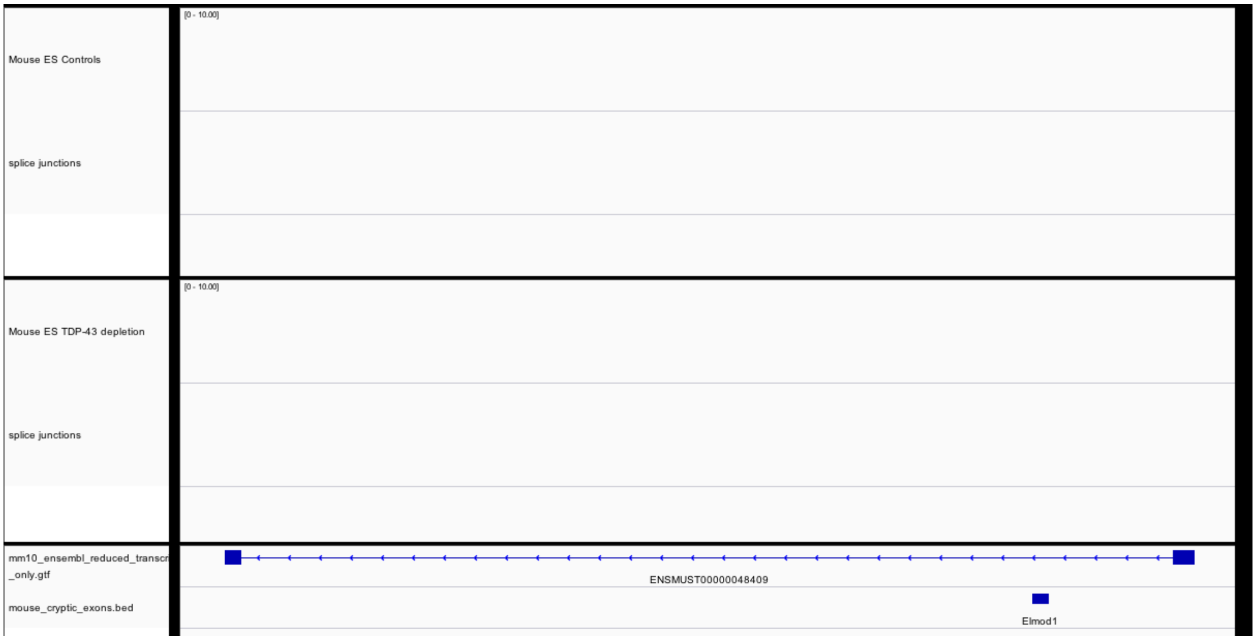

40 Mkx E007i7

Mouse Adult Brain

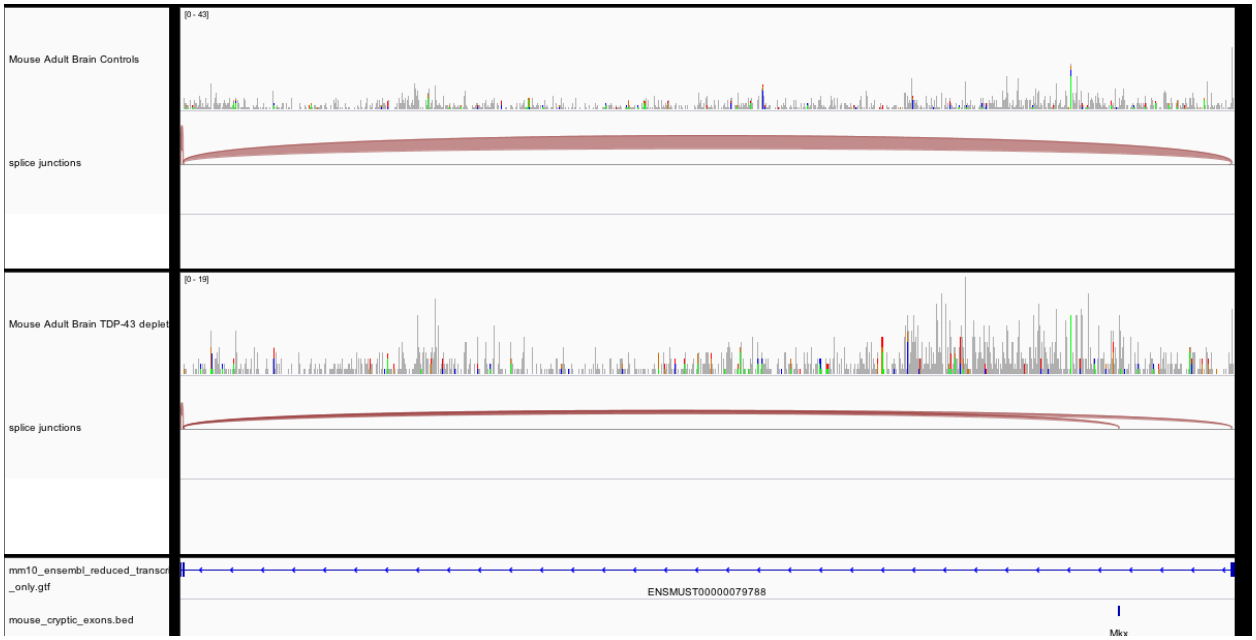

Mouse Embryonic Stem Cells

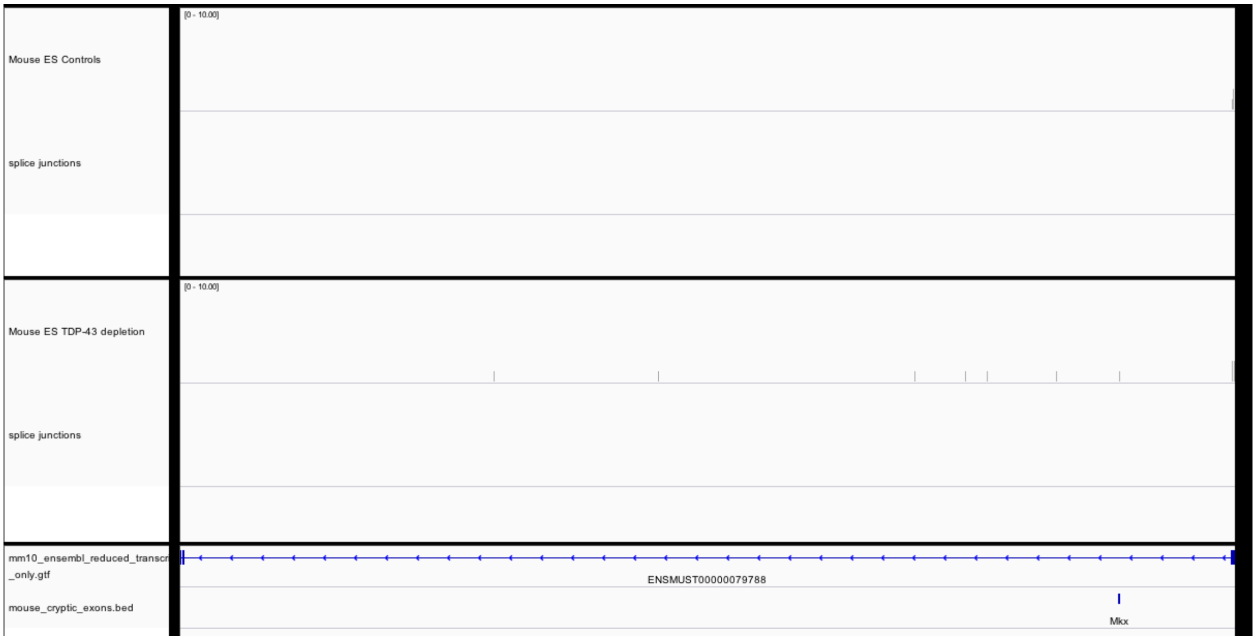

41 Hace1 E013i1

Mouse Adult Brain

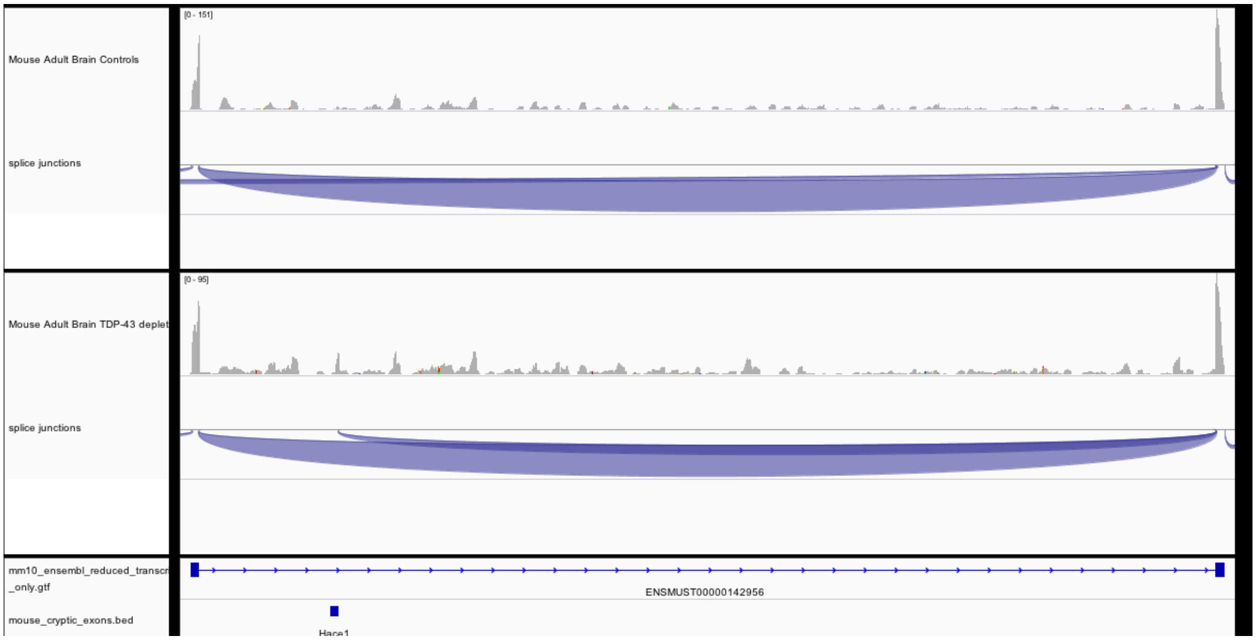

Mouse Embryonic Stem Cells

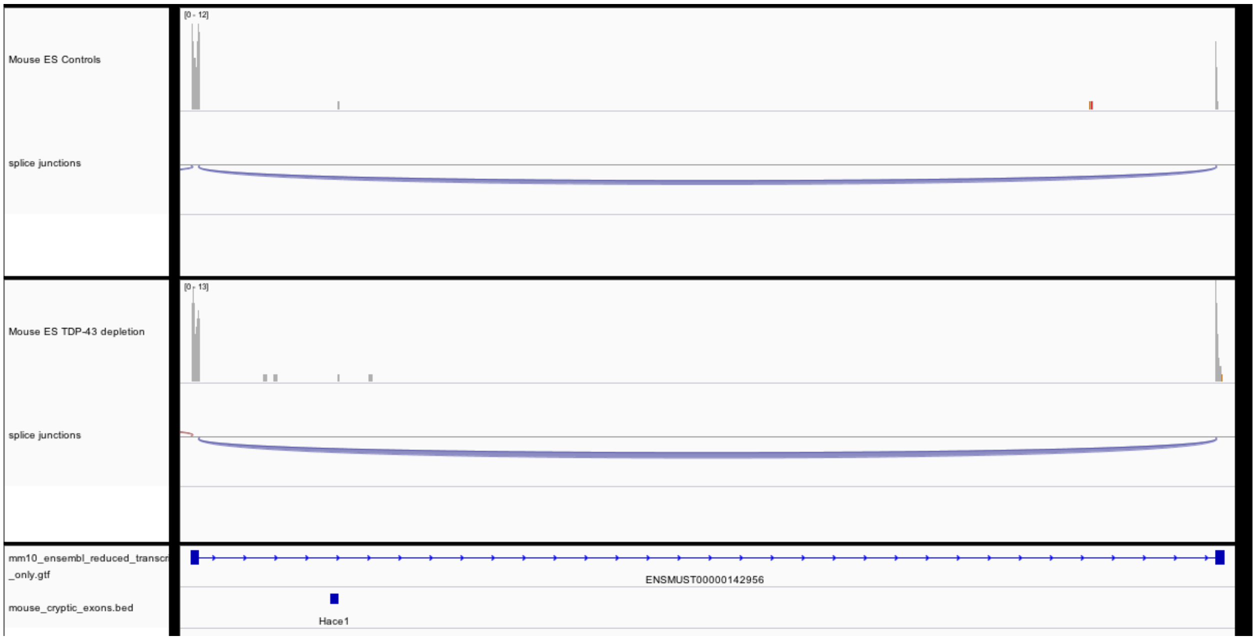

42 Fxyd2 E003i1

Mouse Adult Brain

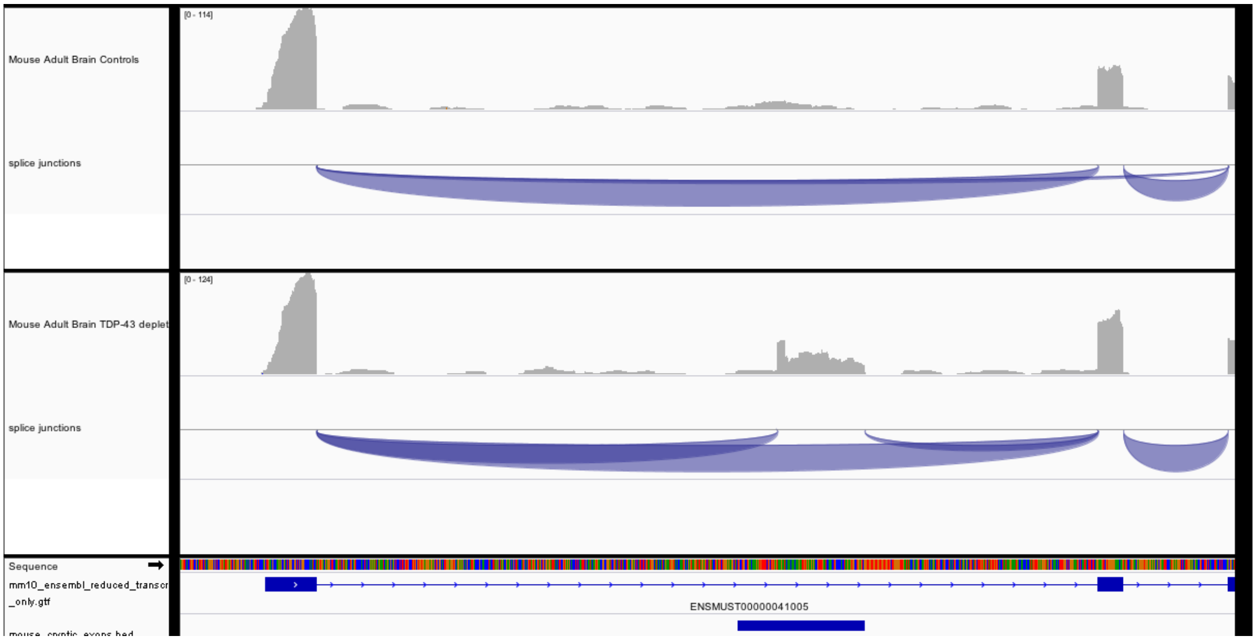

Mouse Embryonic Stem Cells

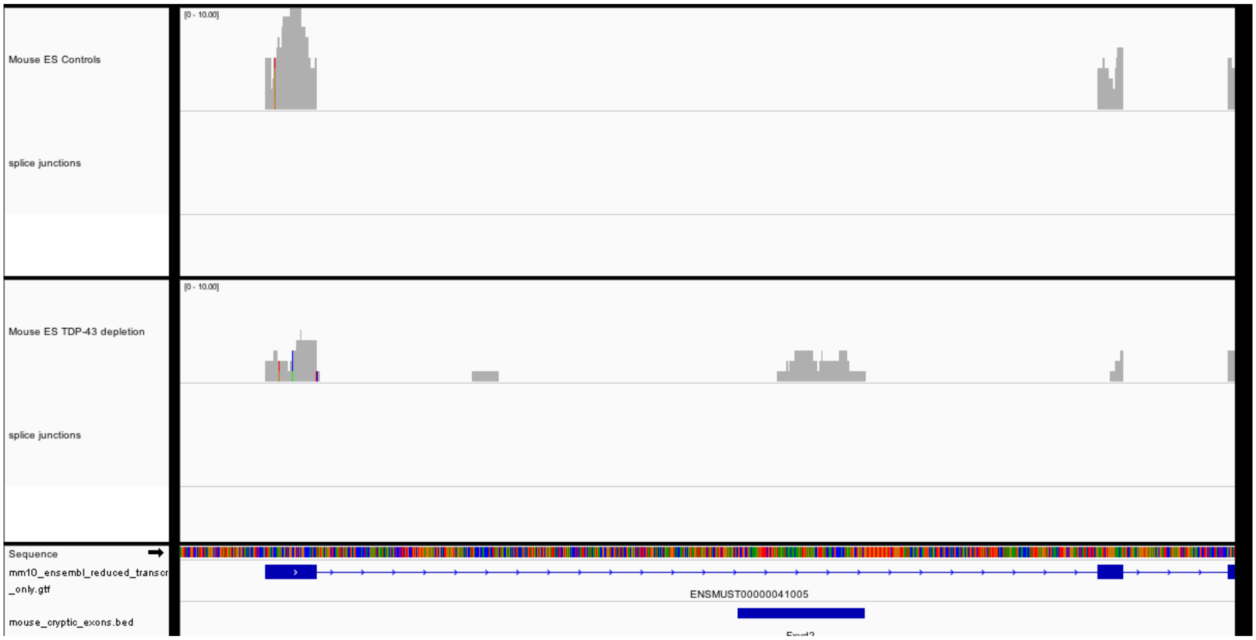

43 Thoc7 E007i1

Mouse Adult Brain

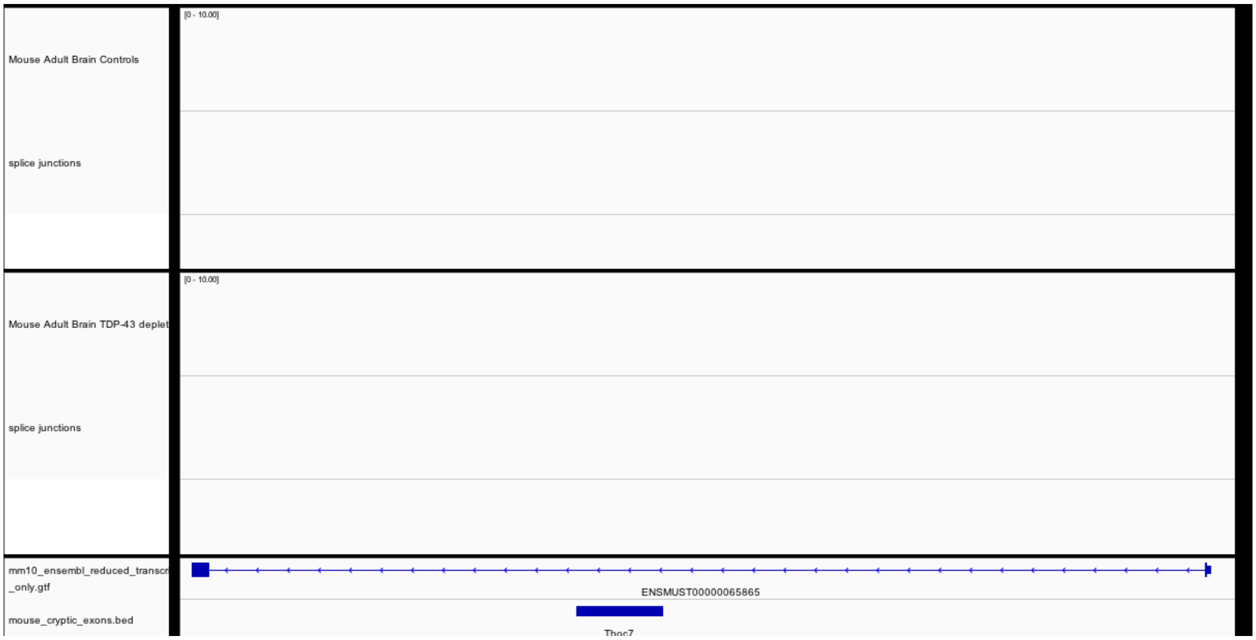

Mouse Embryonic Stem Cells

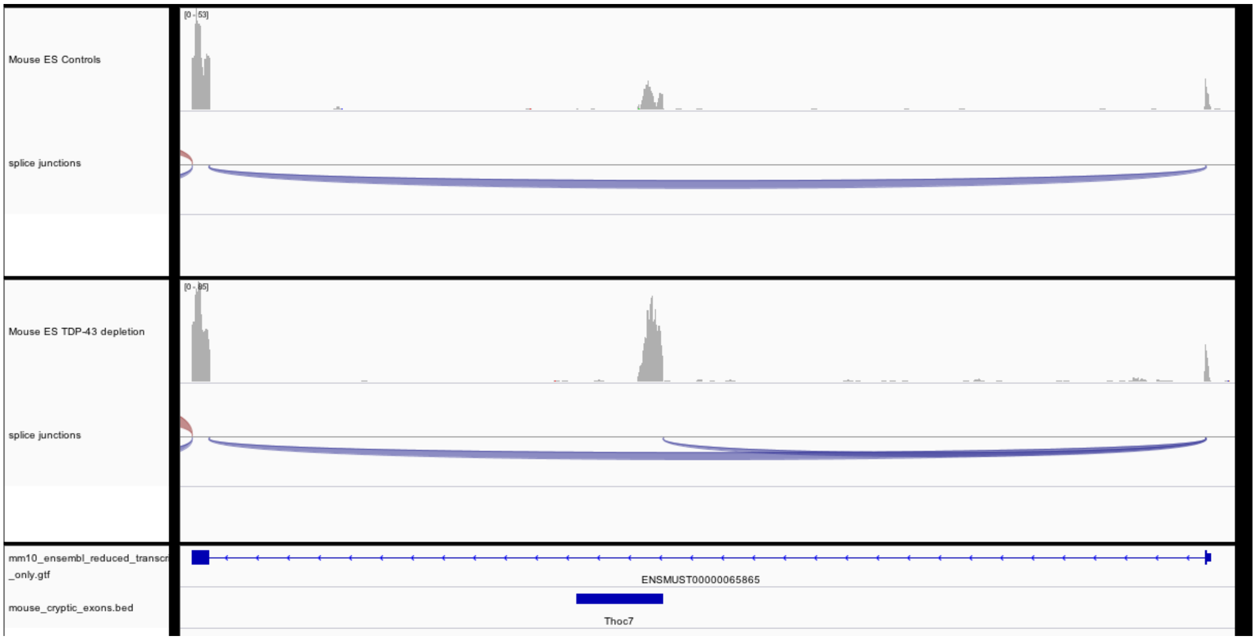

44 Nme6 E020i1

Mouse Adult Brain

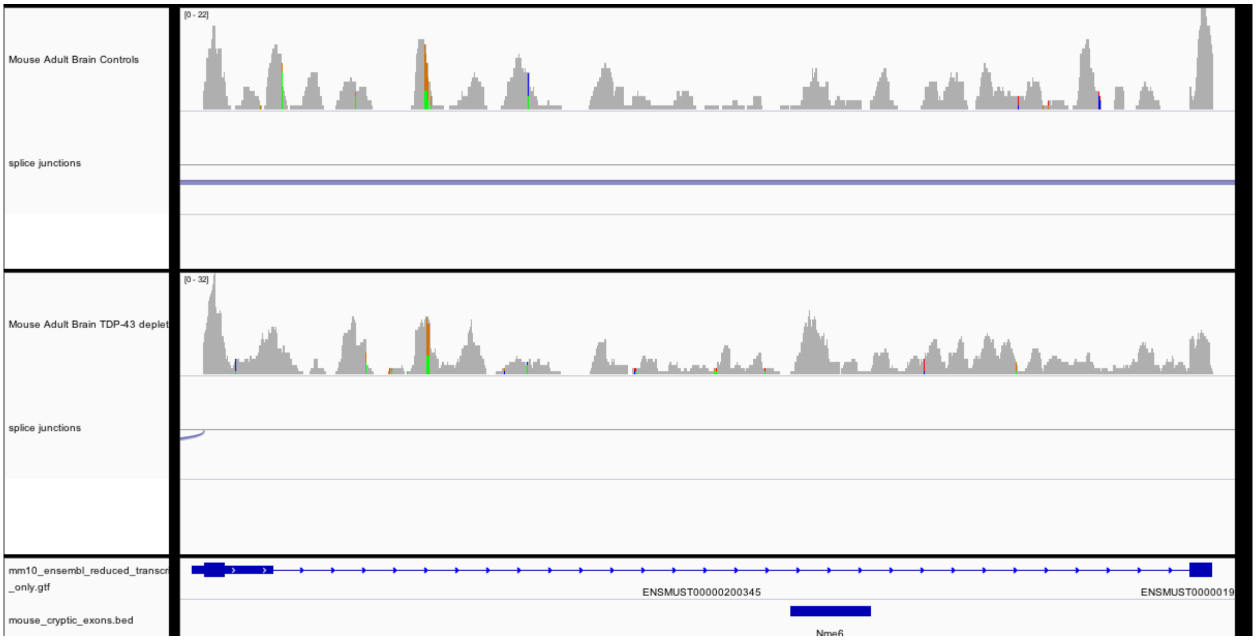

Mouse Embryonic Stem Cells

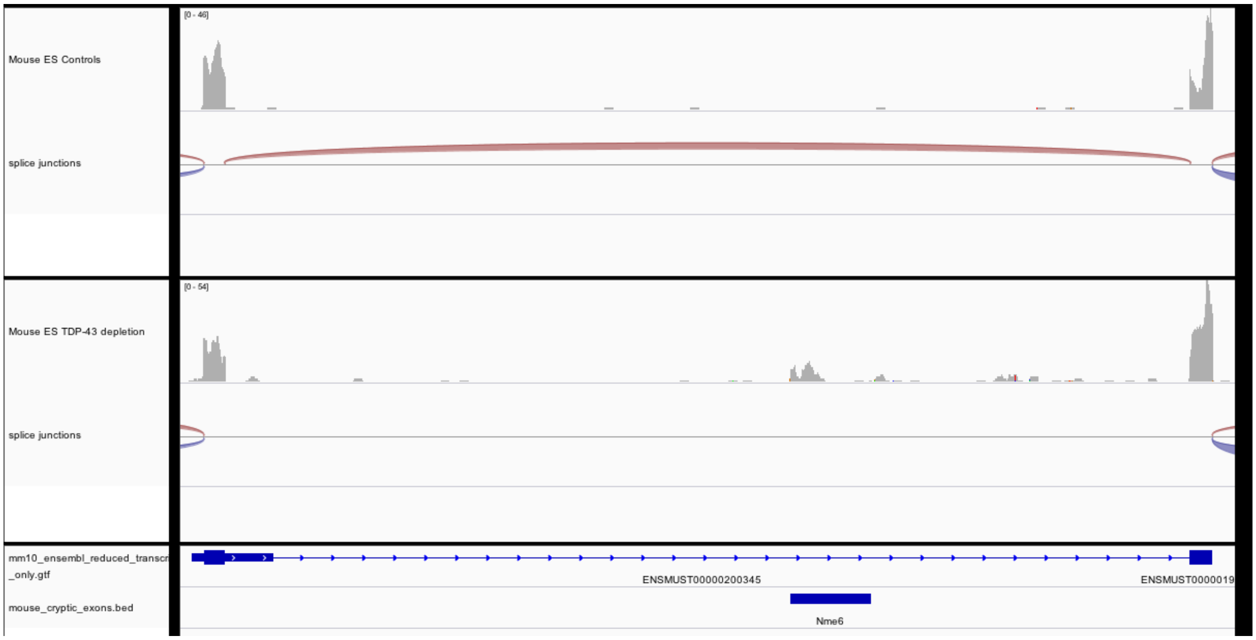

45 Wtip E001i1

Mouse Adult Brain

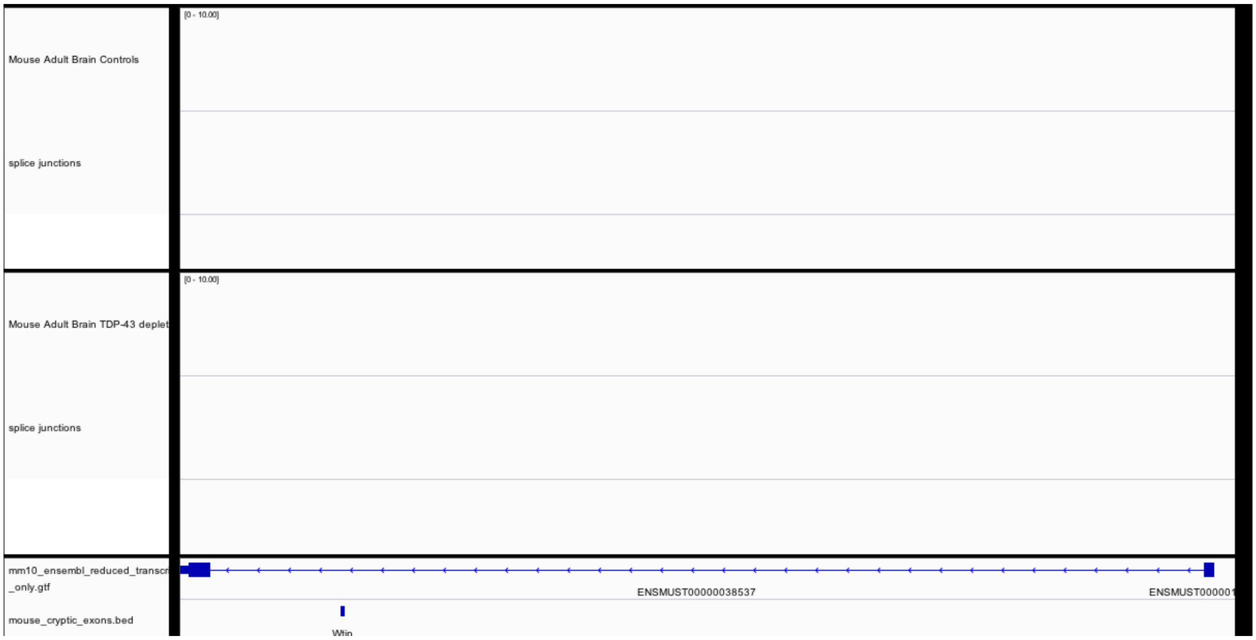

Mouse Embryonic Stem Cells

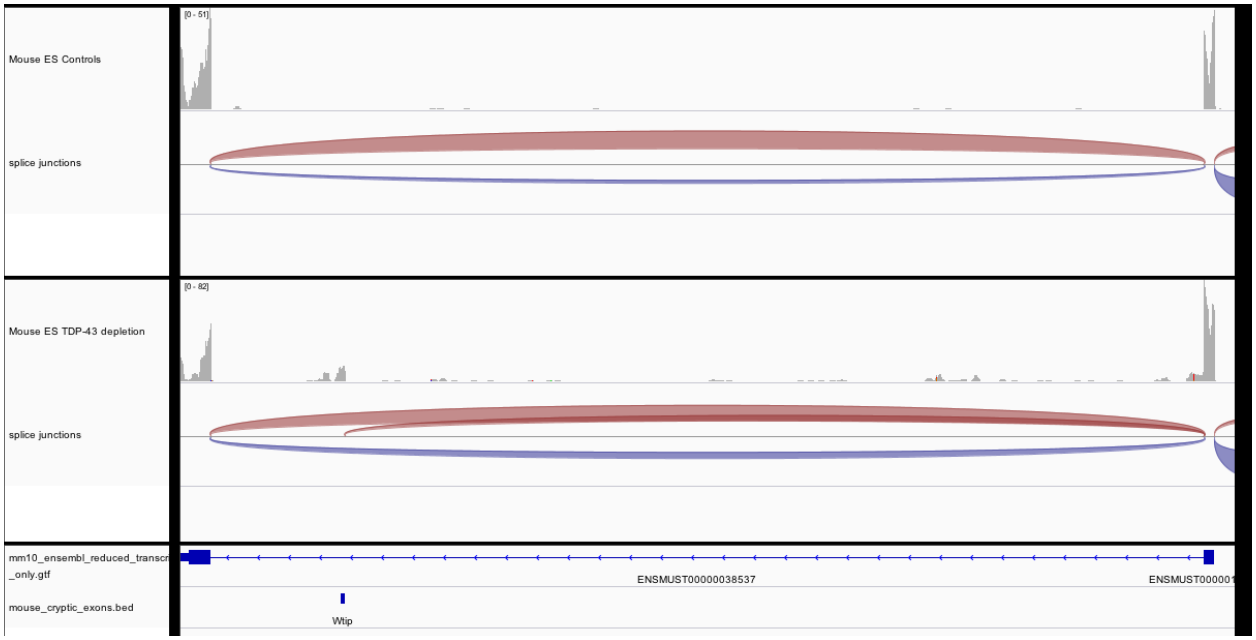

46 Gsta4 E005i2

Mouse Adult Brain

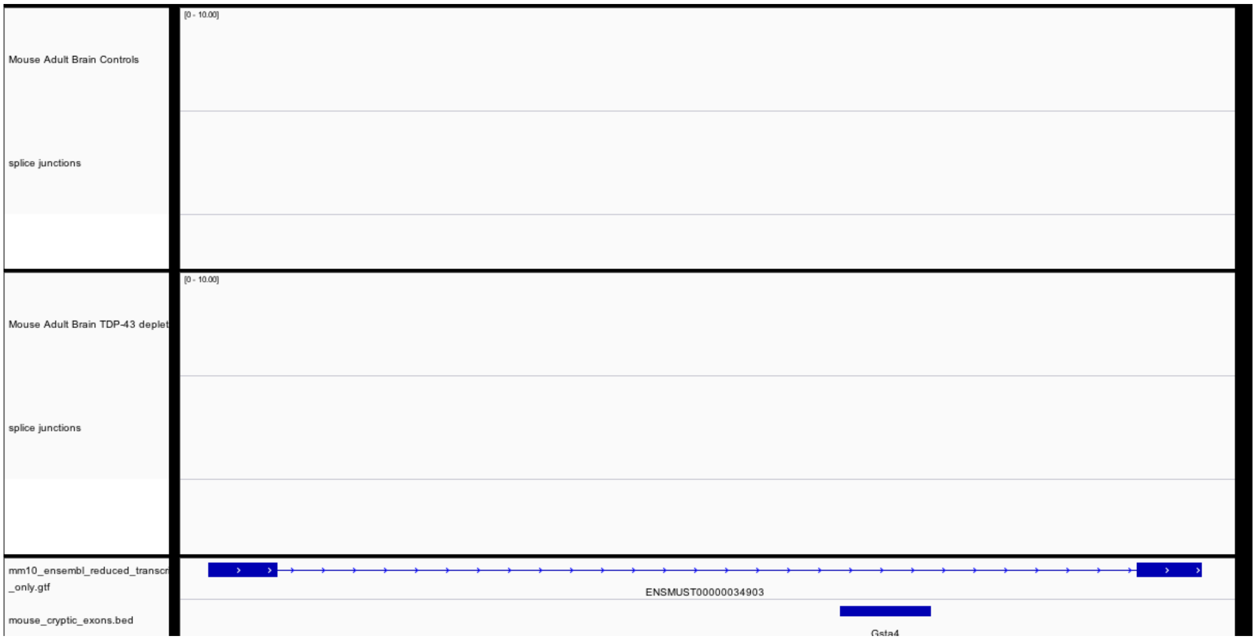

Mouse Embryonic Stem Cells

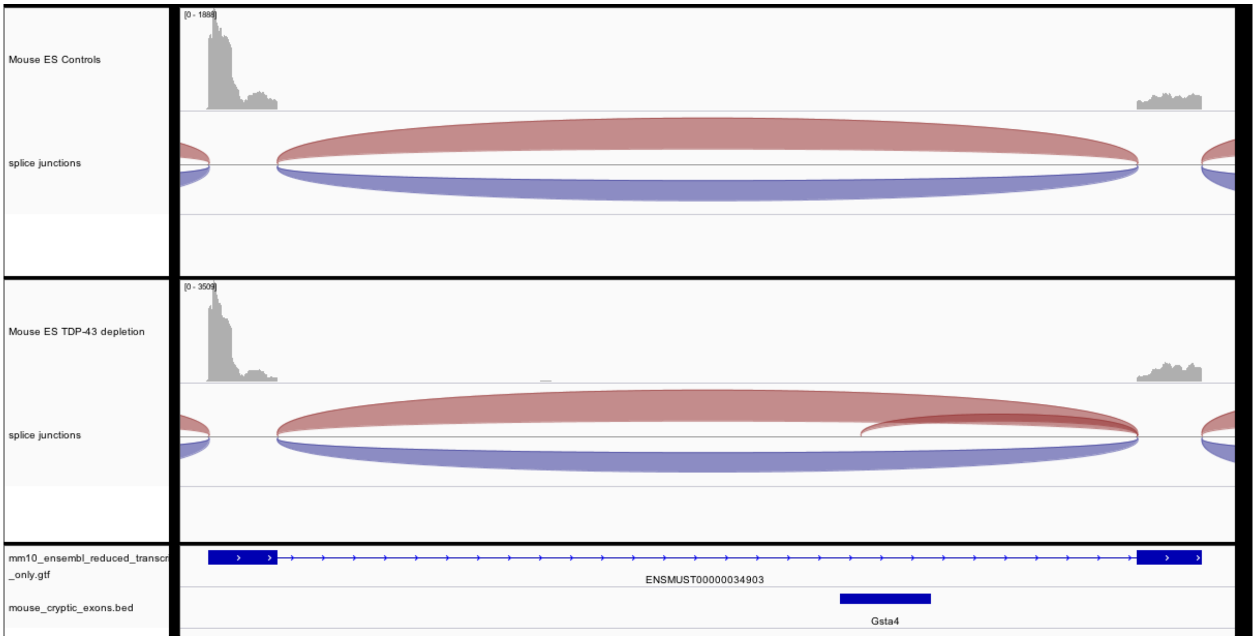

47 Slc7a6 E001i1

Mouse Adult Brain

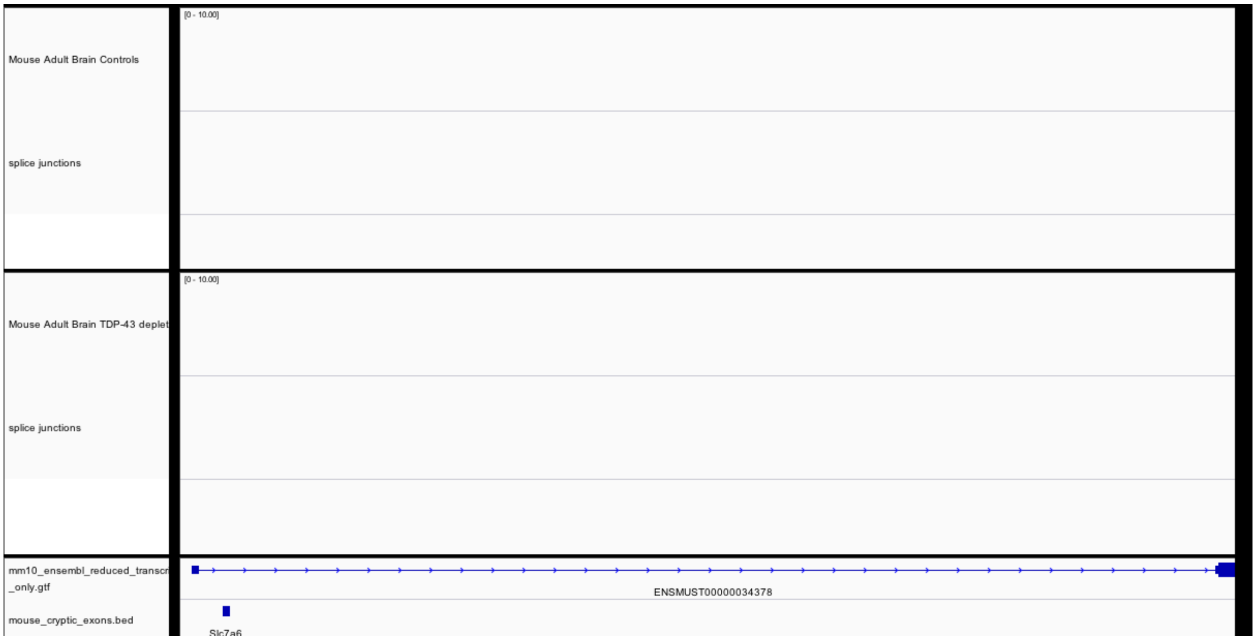

Mouse Embryonic Stem Cells

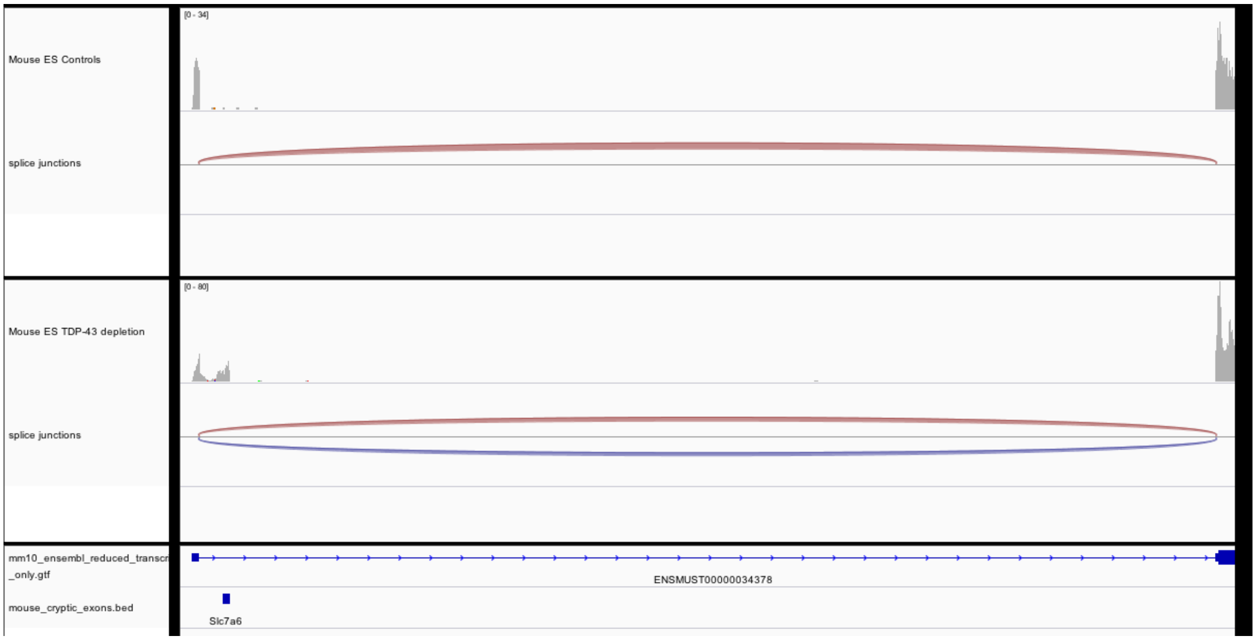

48 Flnb E040i1

Mouse Adult Brain

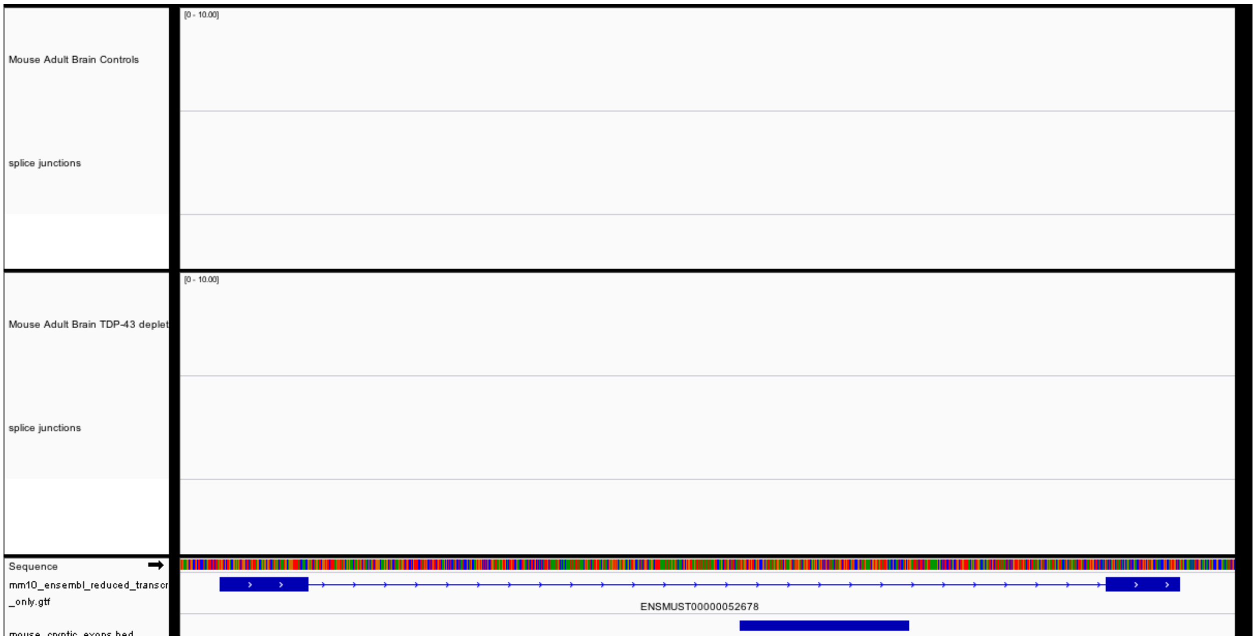

Mouse Embryonic Stem Cells

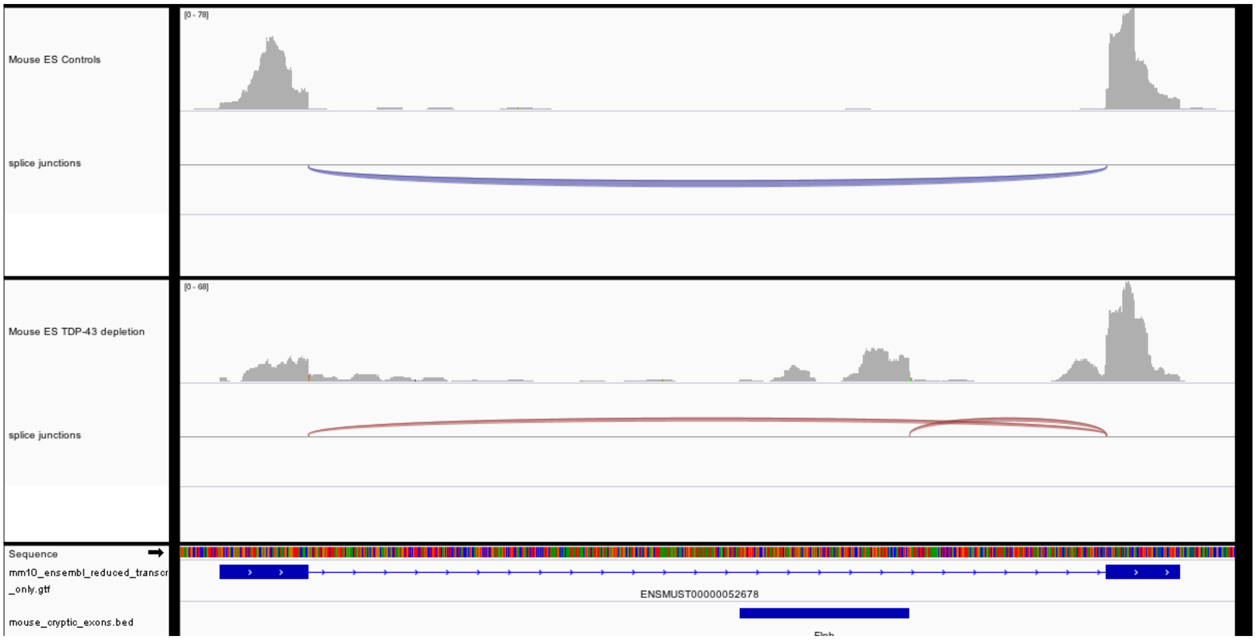

49 B4galnt3 E002i1

Mouse Adult Brain

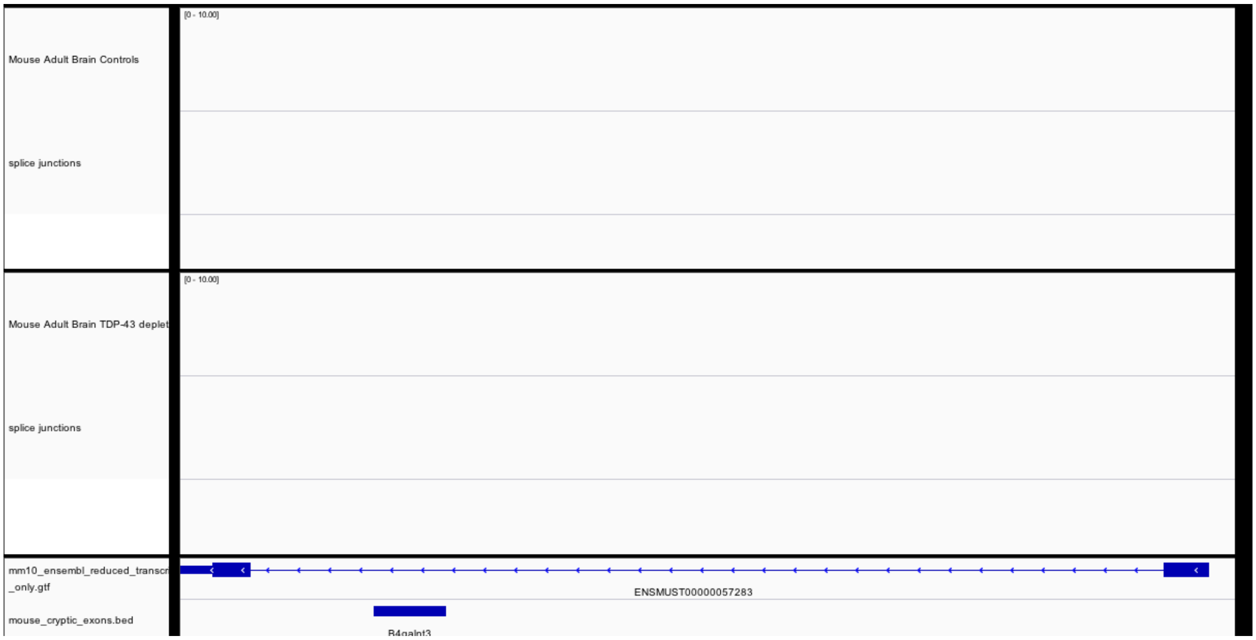

Mouse Embryonic Stem Cells

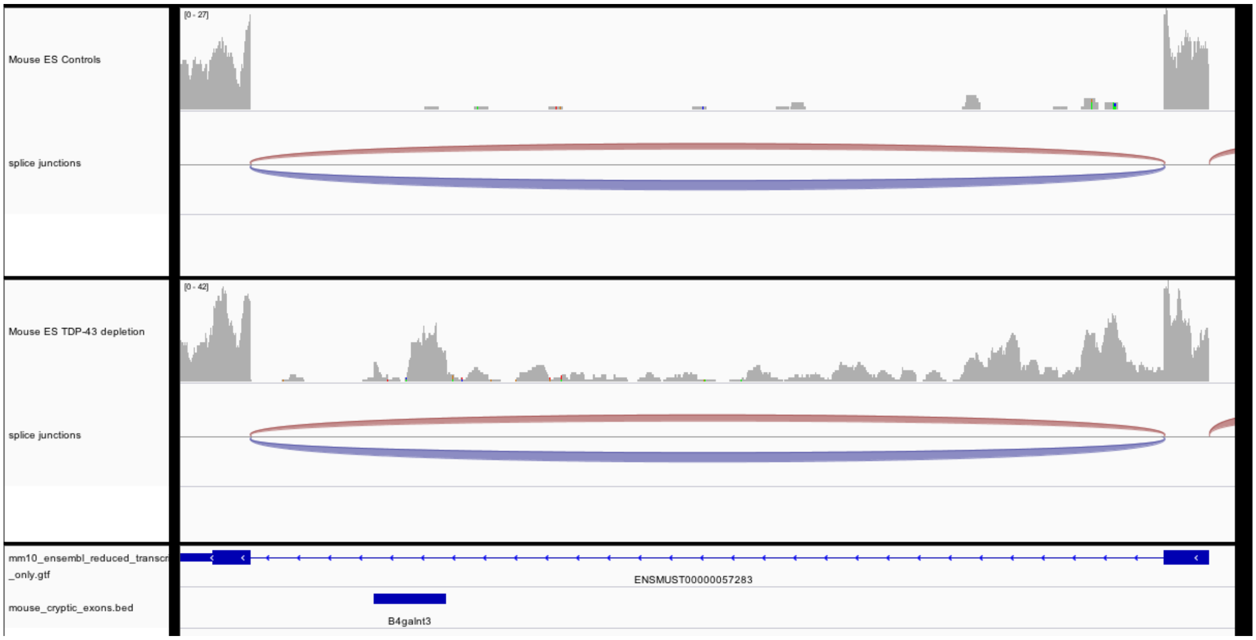

50 Psma2 E005i1

Mouse Adult Brain

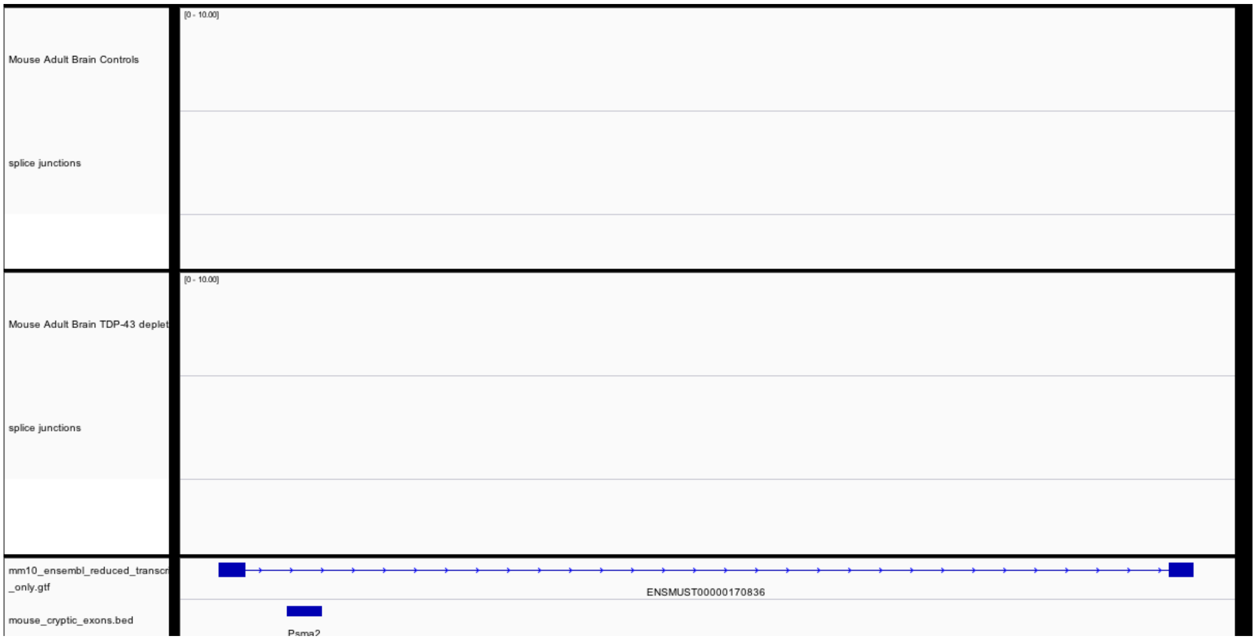

Mouse Embryonic Stem Cells

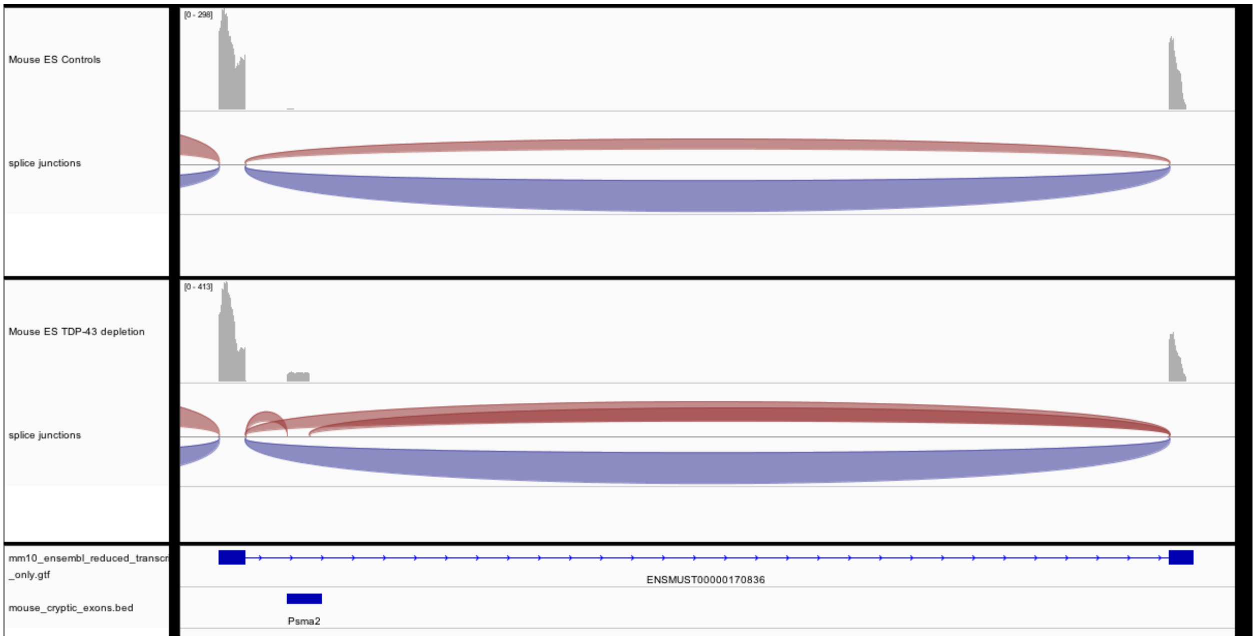

51 Abr E024i1

Mouse Adult Brain

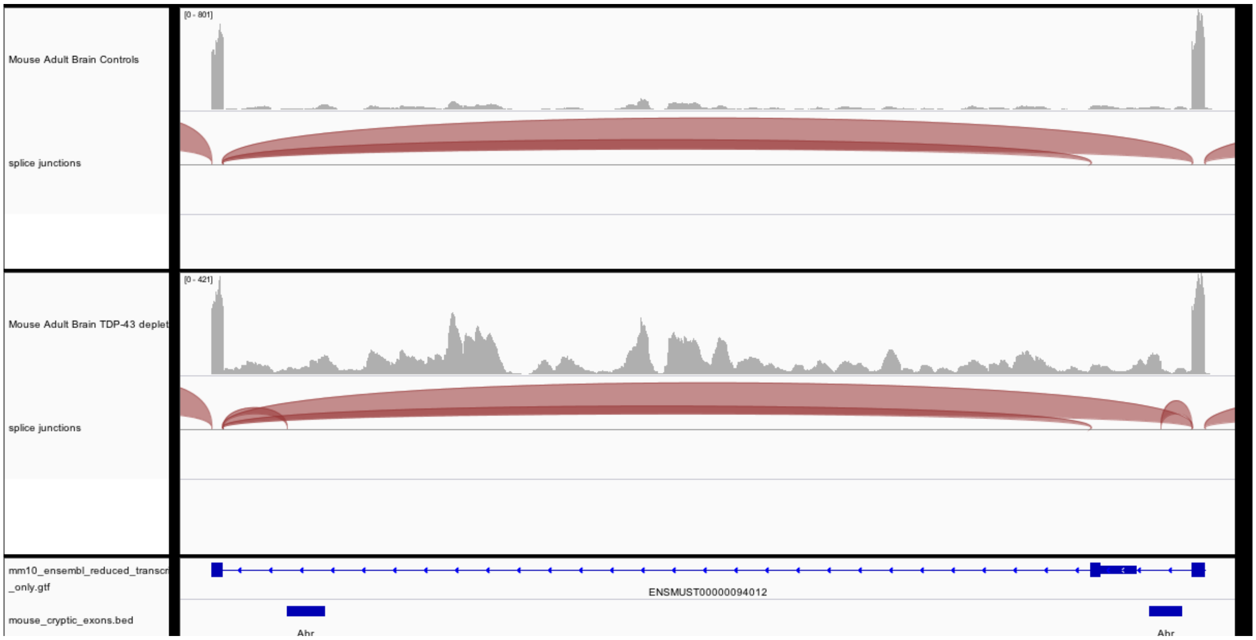

Mouse Embryonic Stem Cells

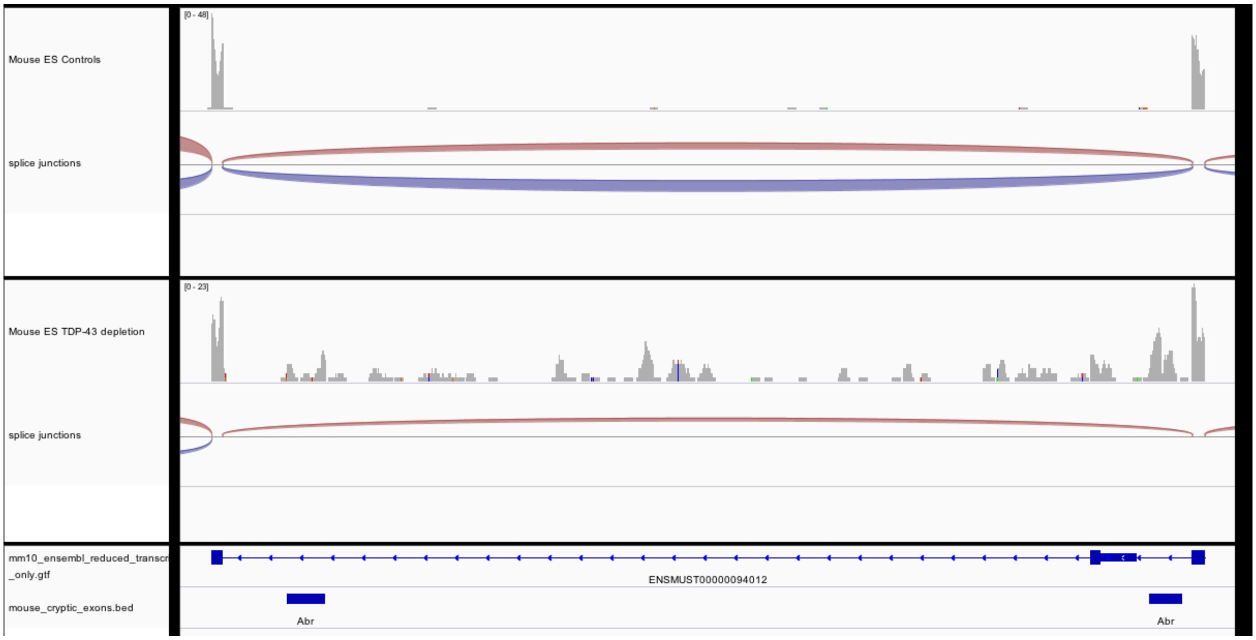

52 Wasf3 E009i1

Mouse Adult Brain

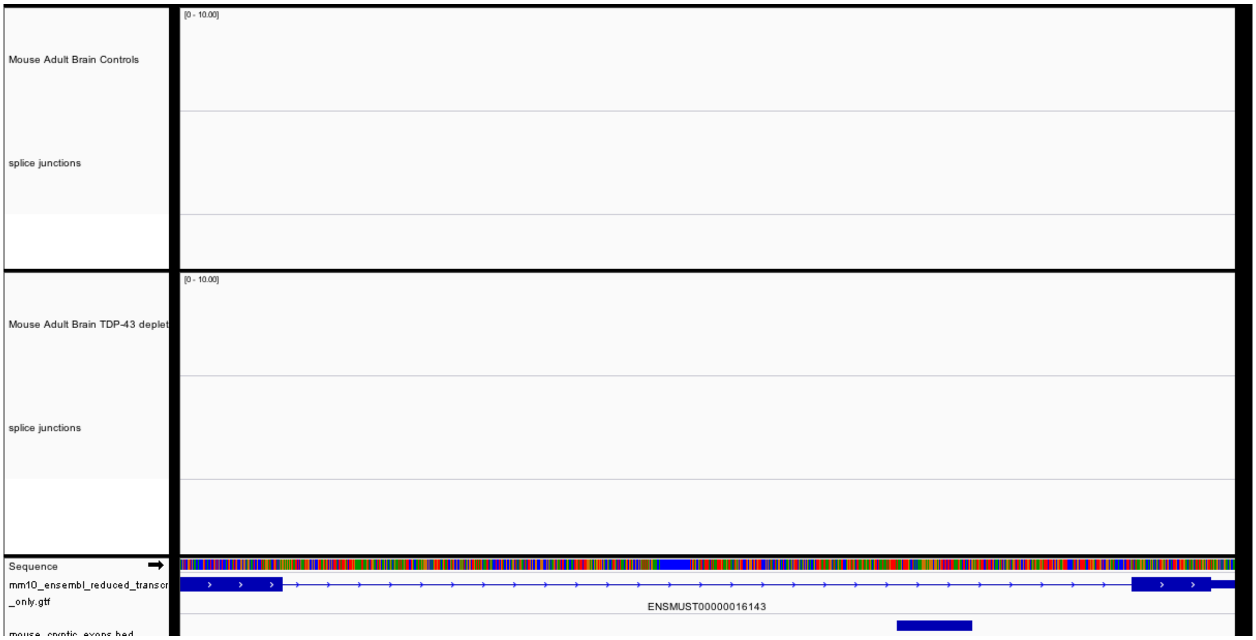

Mouse Embryonic Stem Cells

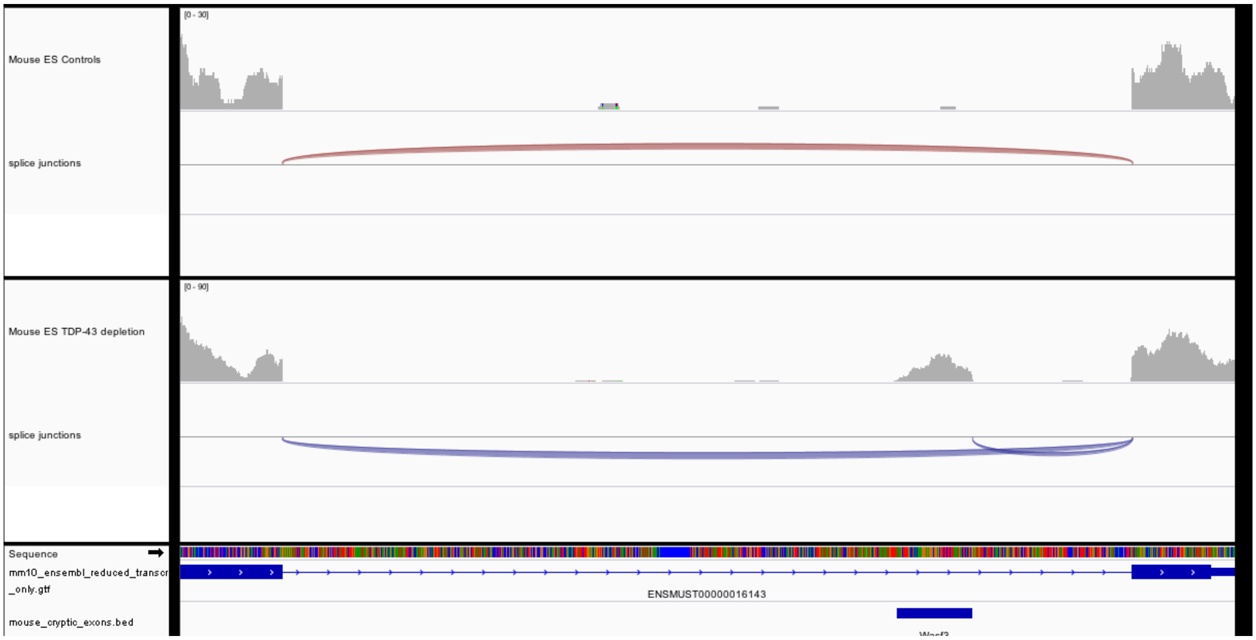

Supplement: Supplementary file 2 — Each cryptic exon discovered in either of the two mouse datasets. For each dataset, the biological replicates were combined to create merged BAM files for each condition. The BAM files were loaded into IGV for visualisation. Read coverage and splice junctions are shown. The Ensembl transcripts for the mm10 build are provided, as are the coordinates of the cryptic exons discovered by CryptEx. (PDF 2436 kb) [file 12920_2017_274_MOESM2_ESM.pdf]
