# Supplementary material for: Quantitative analysis of cryptic splicing associated with TDP-43 depletion
Source: BMC Med Genomics. 2017 May 26;10:38. doi: 10.1186/s12920-017-0274-1 (PMC5446763; doi:10.1186/s12920-017-0274-1)

Figure S3

Human Cryptic Exons from *Quantitative analysis of cryptic splicing associated with TDP-43 depletion*

Jack Humphrey, Warren Emmett, Pietro Fratta, Adrian M. Isaacs & Vincent Plagnol

September 16, 2016

Each cryptic exon discovered in either of the two human K562 datasets was visualised in both datasets using the IGV browser. For each dataset, the biological replicates were combined to create merged files for each condition. Read coverage and splice junctions are shown. The Ensembl transcripts for the hg38 build are provided, as are the coordinates of the cryptic exons discovered by CryptEx.

1 PHF12 E028i1

Human K562 mRNA

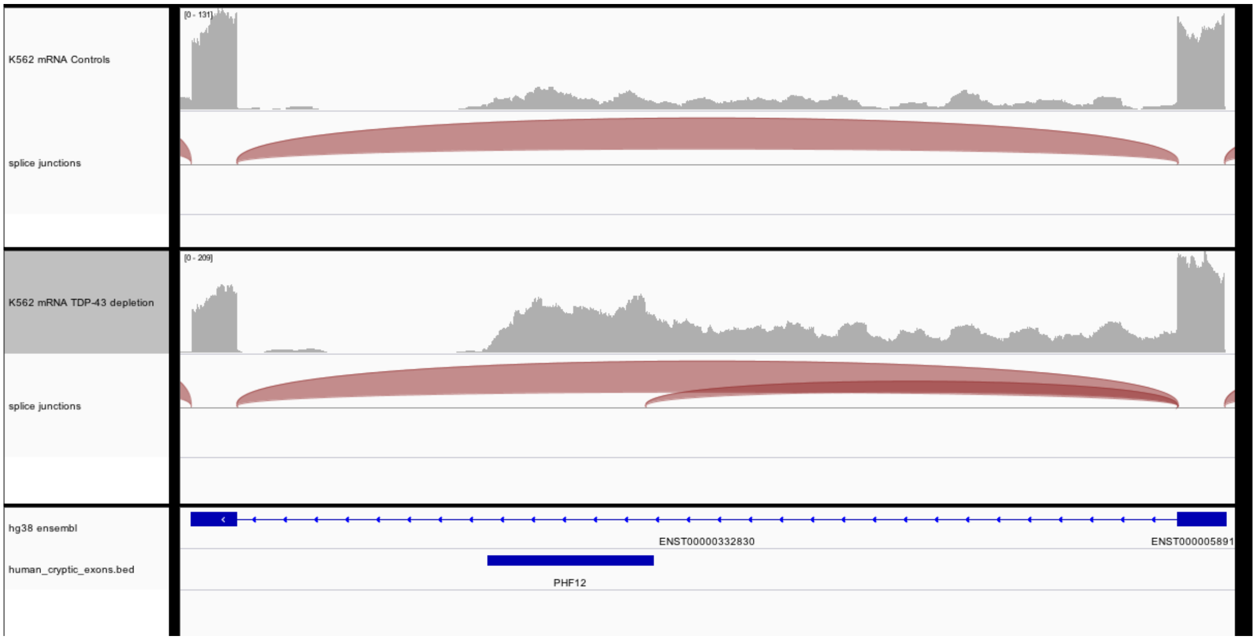

Human K562 total RNA

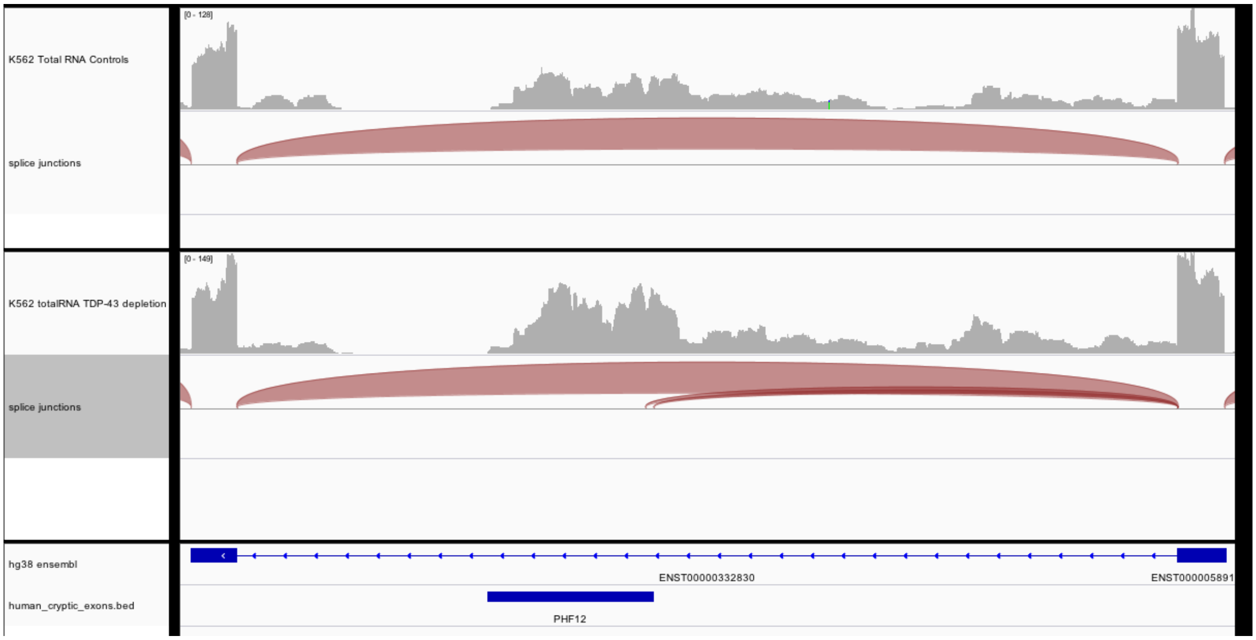

2 ZFPM2 E016i1

Human K562 mRNA

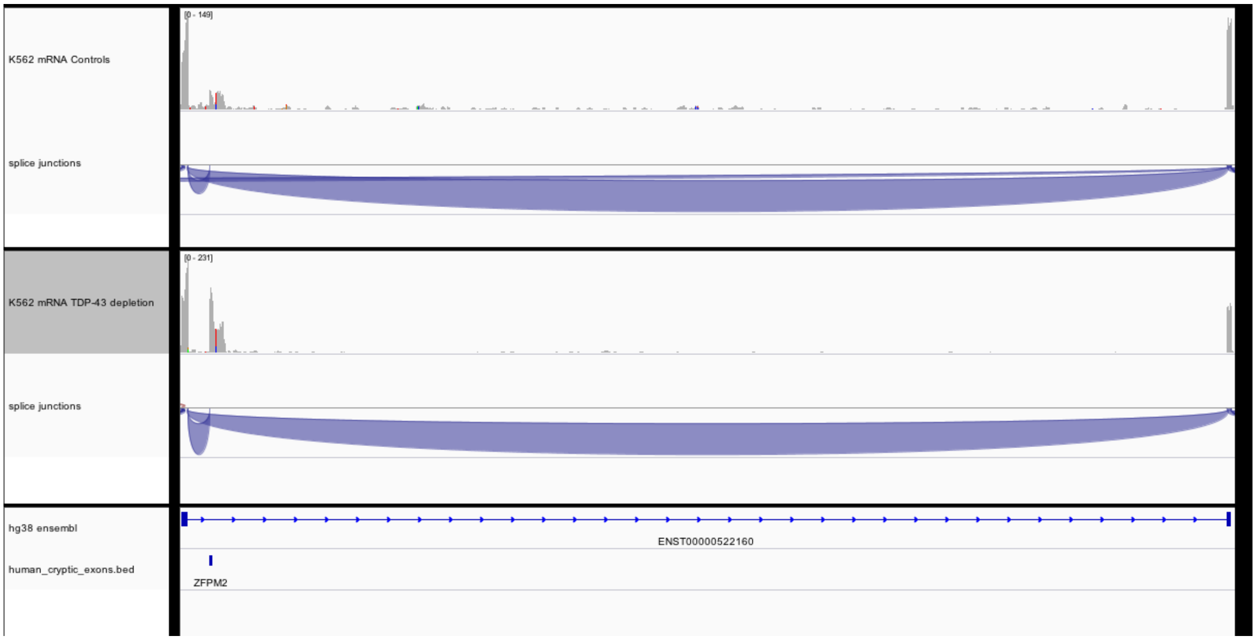

Human K562 total RNA

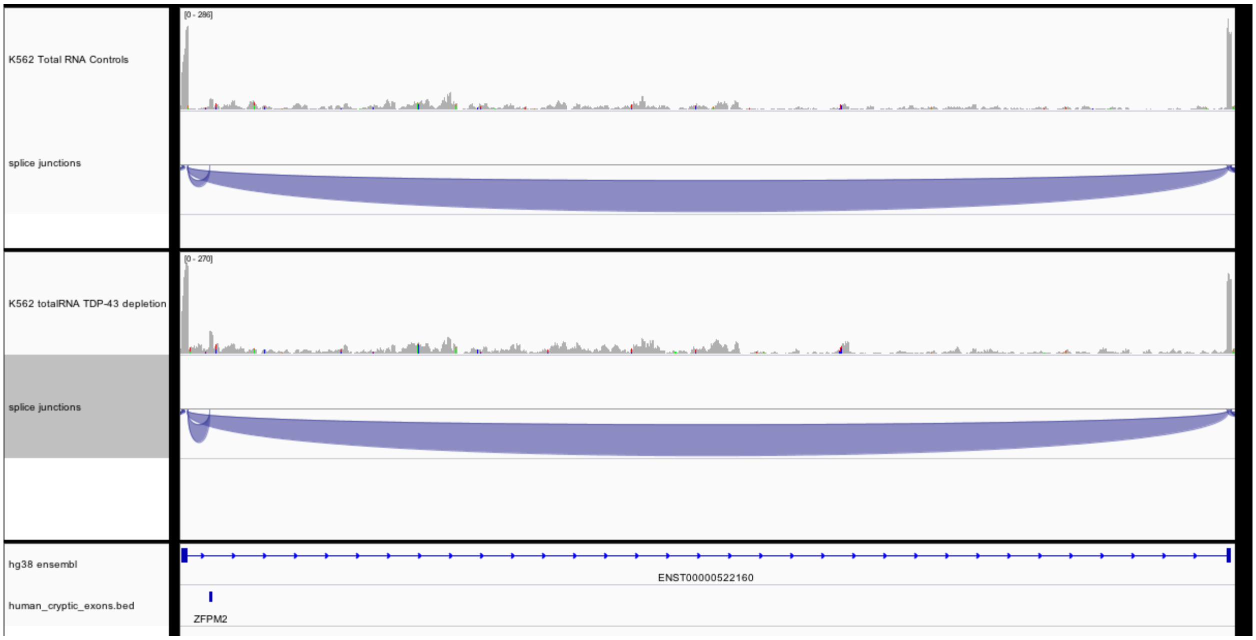

3 ACBD3 E004i1

Human K562 mRNA

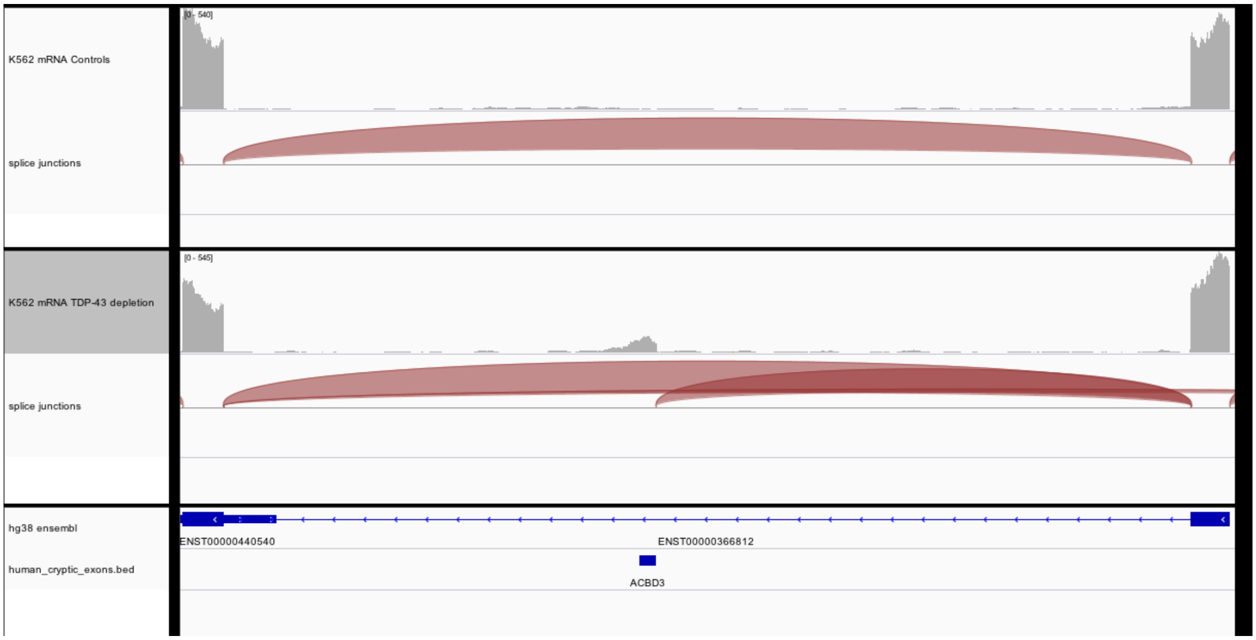

Human K562 total RNA

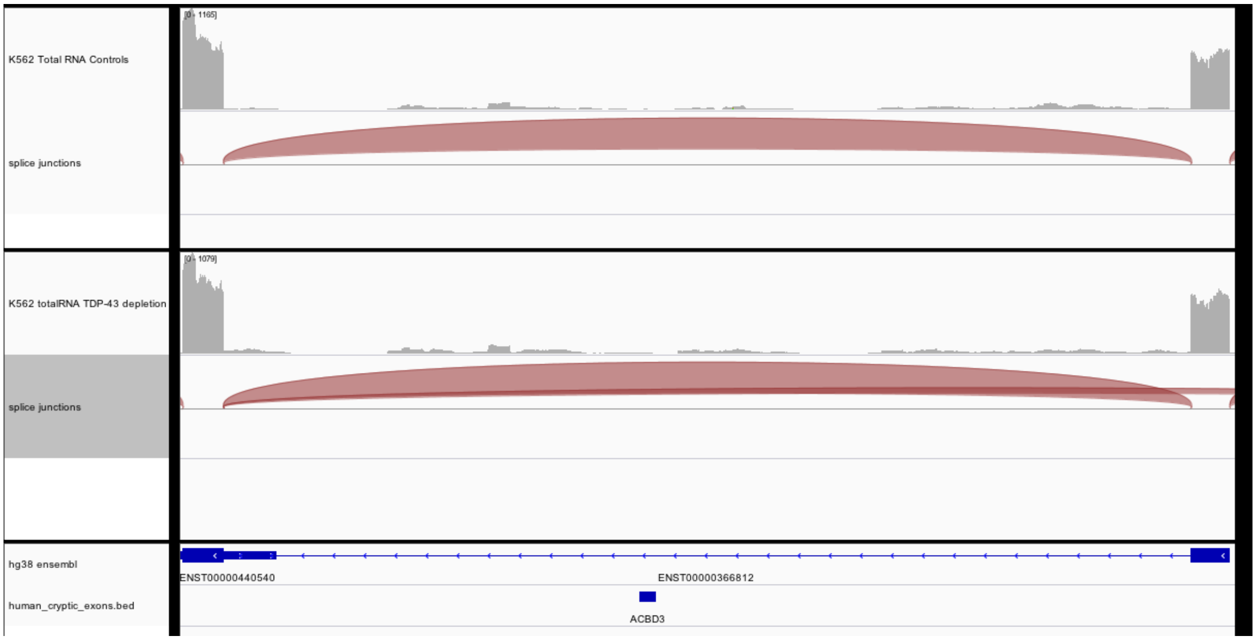

4 RPS6KA3 E031i2

Human K562 mRNA

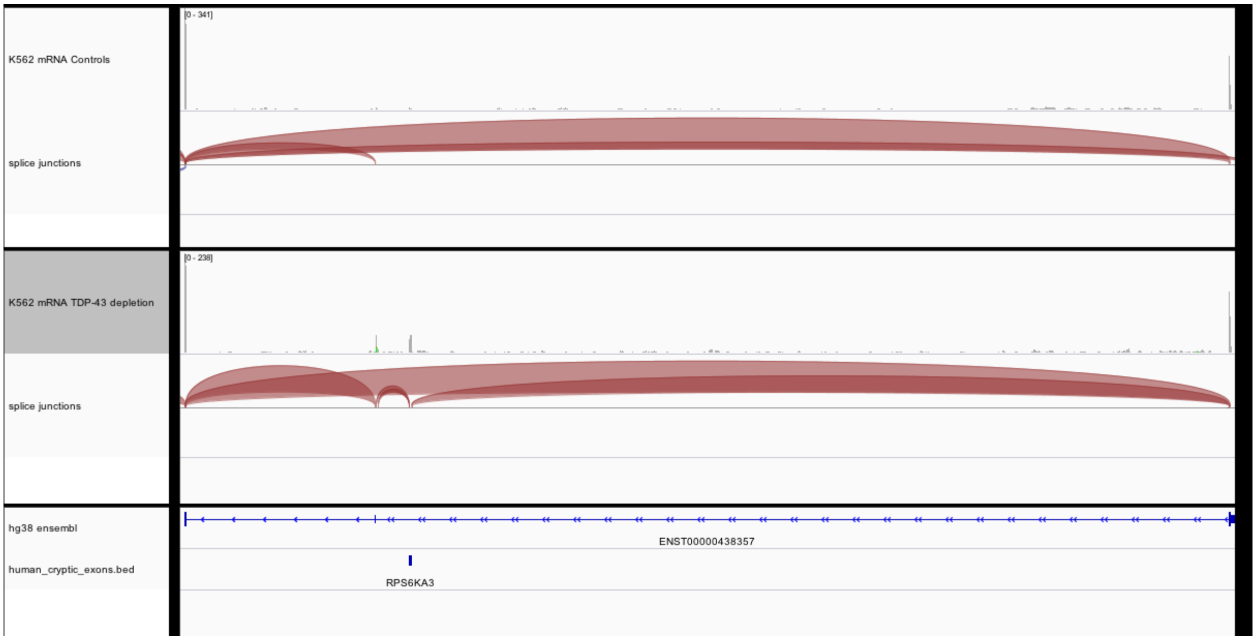

Human K562 total RNA

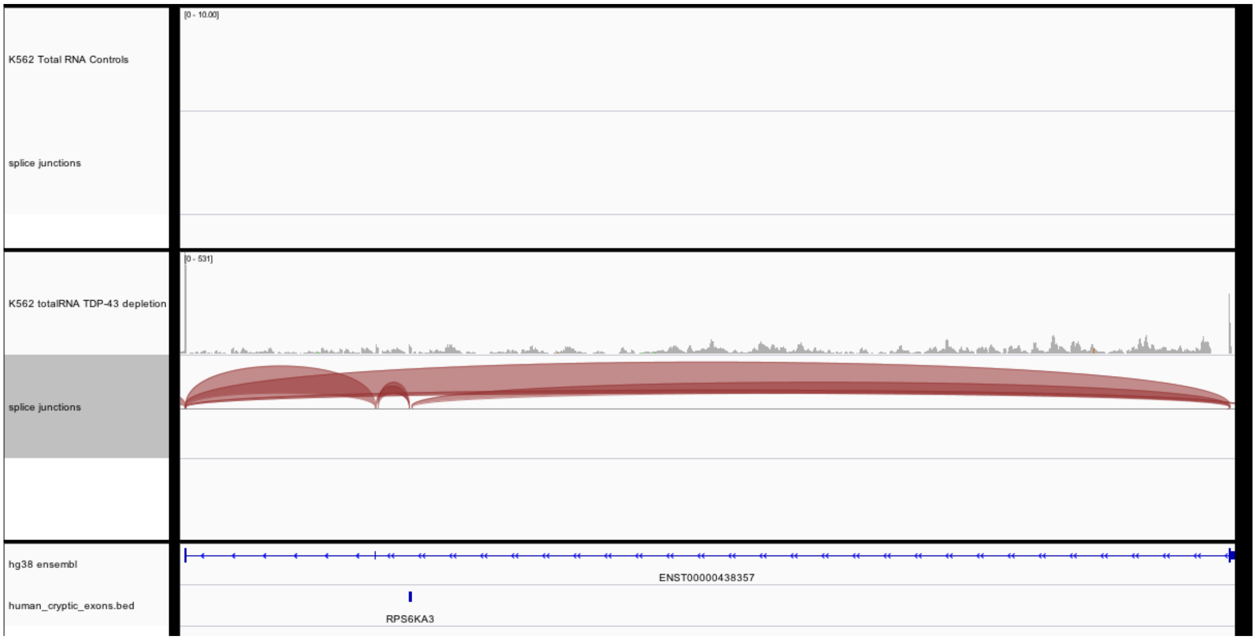

5 CPED1 E034i7

Human K562 mRNA

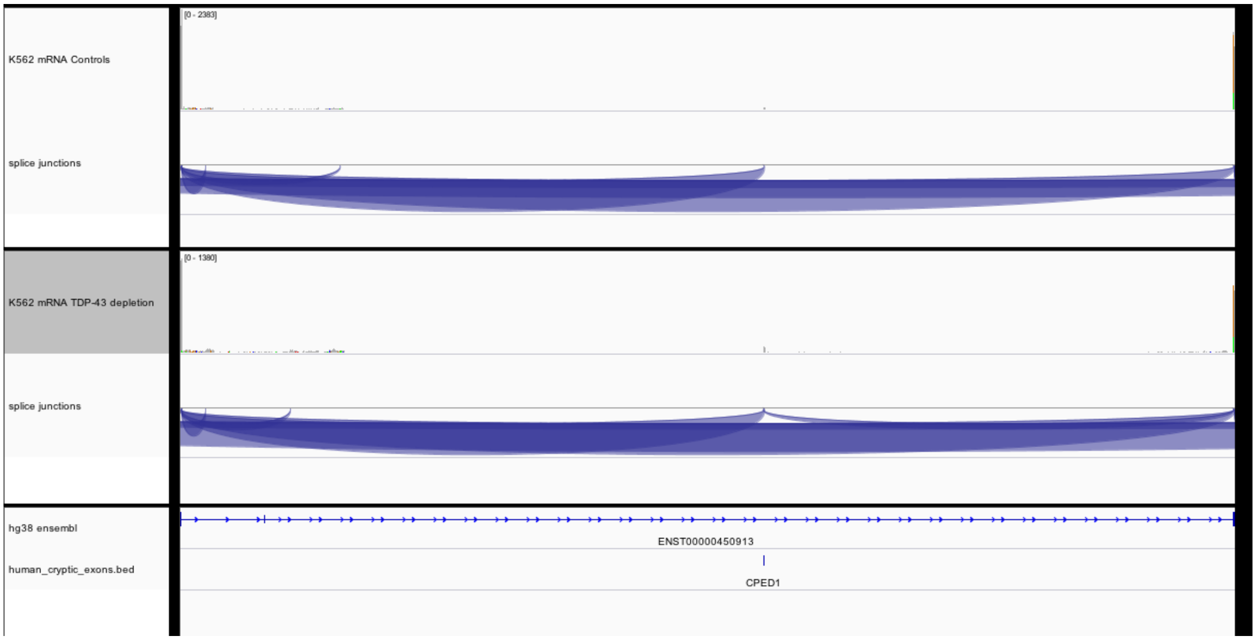

Human K562 total RNA

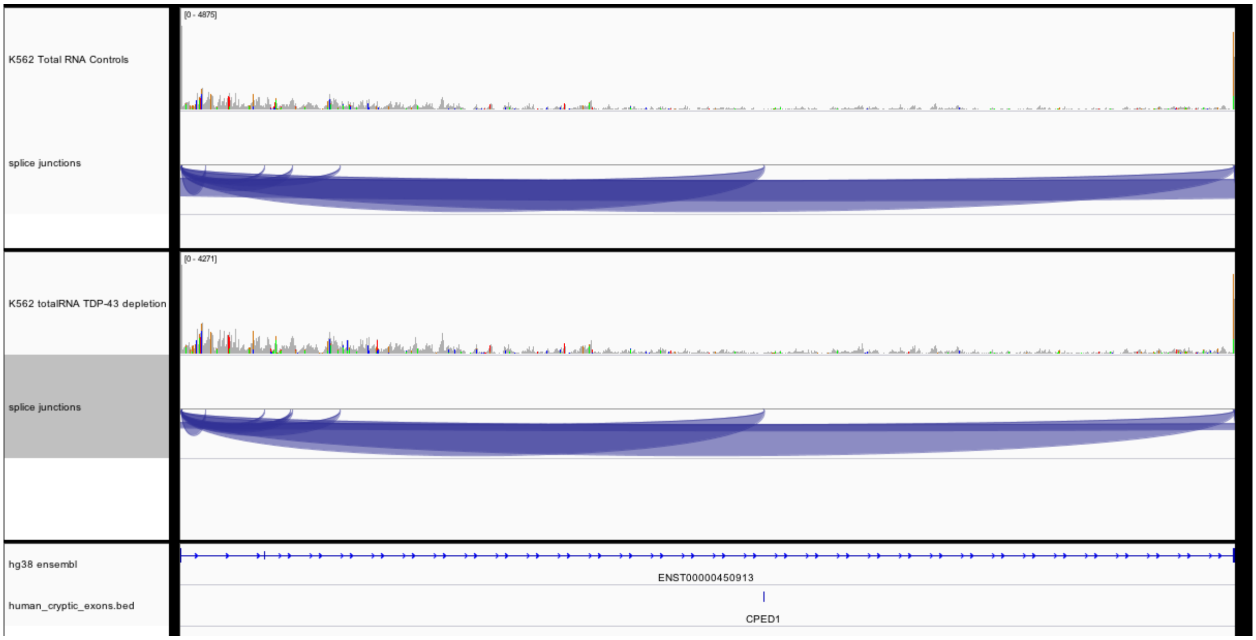

6 HUWE1 E033i1

Human K562 mRNA

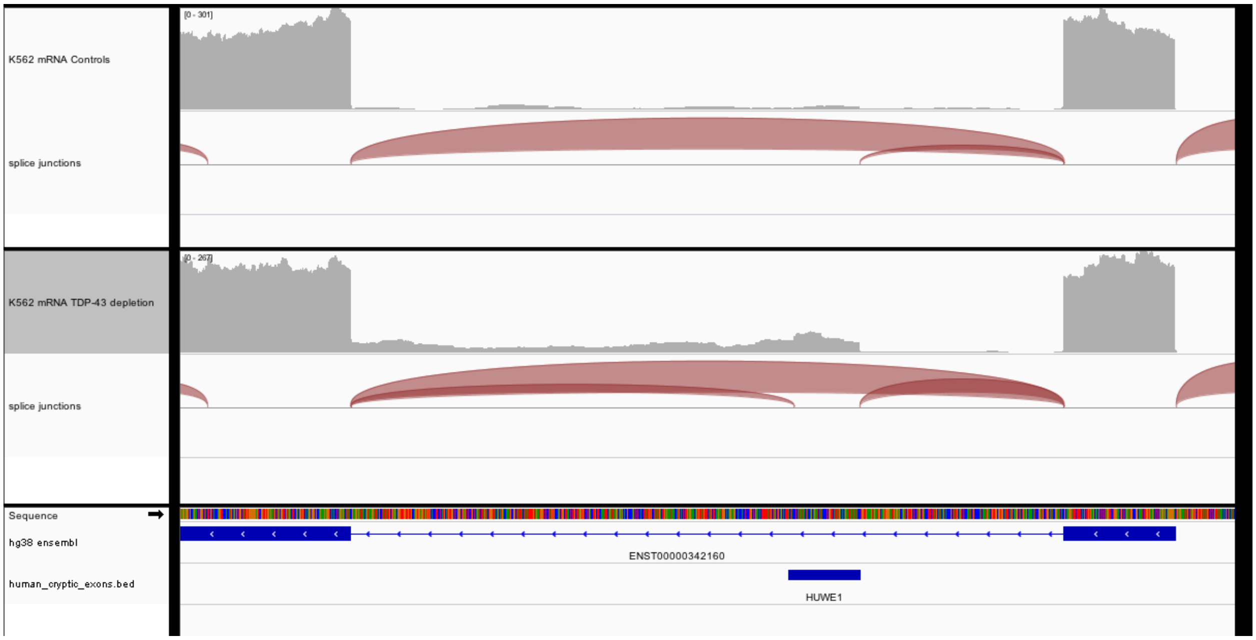

Human K562 total RNA

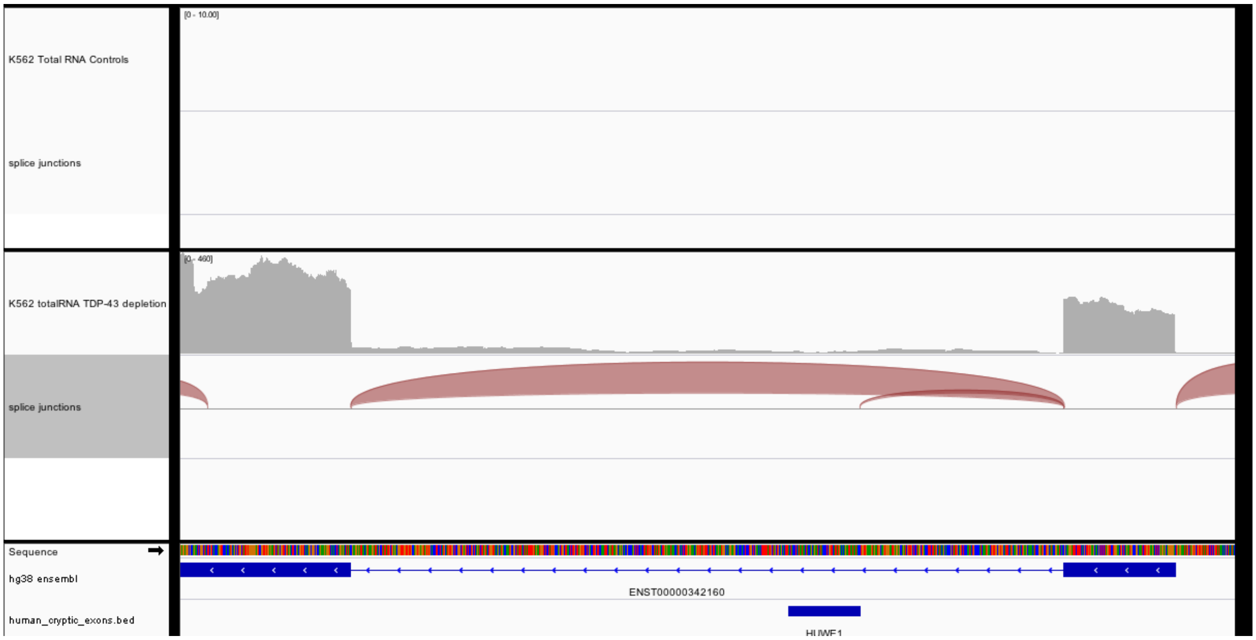

7 DYNC2LI1 E006i1

Human K562 mRNA

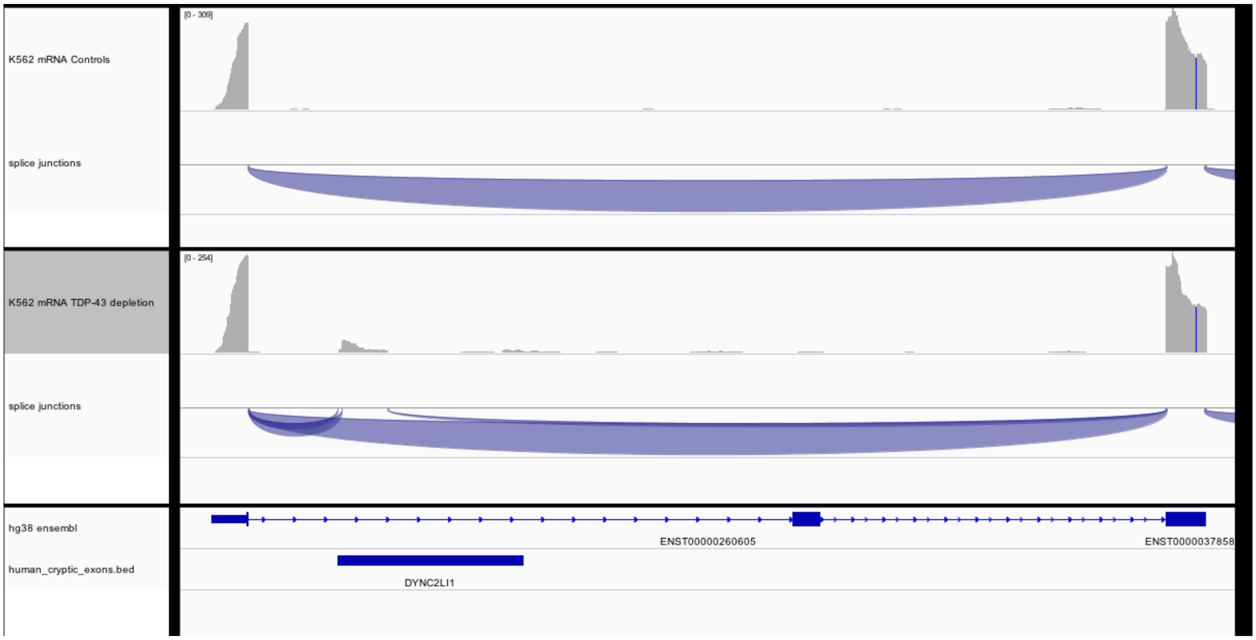

Human K562 total RNA

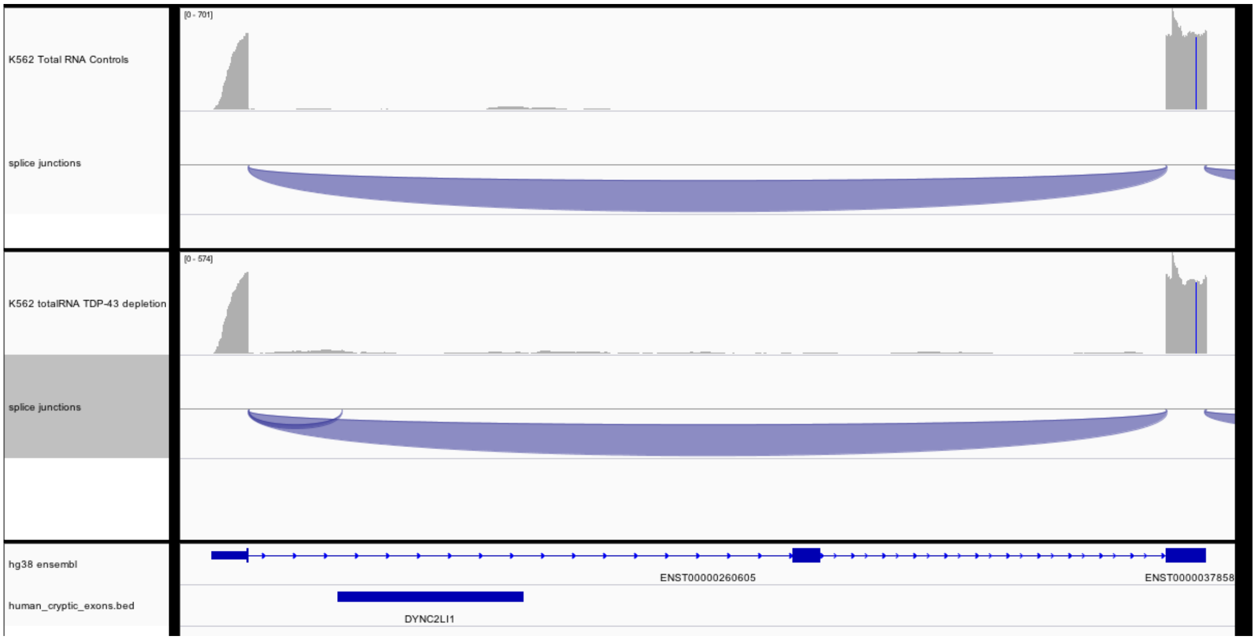

8 ZMYND8 E019i2

Human K562 mRNA

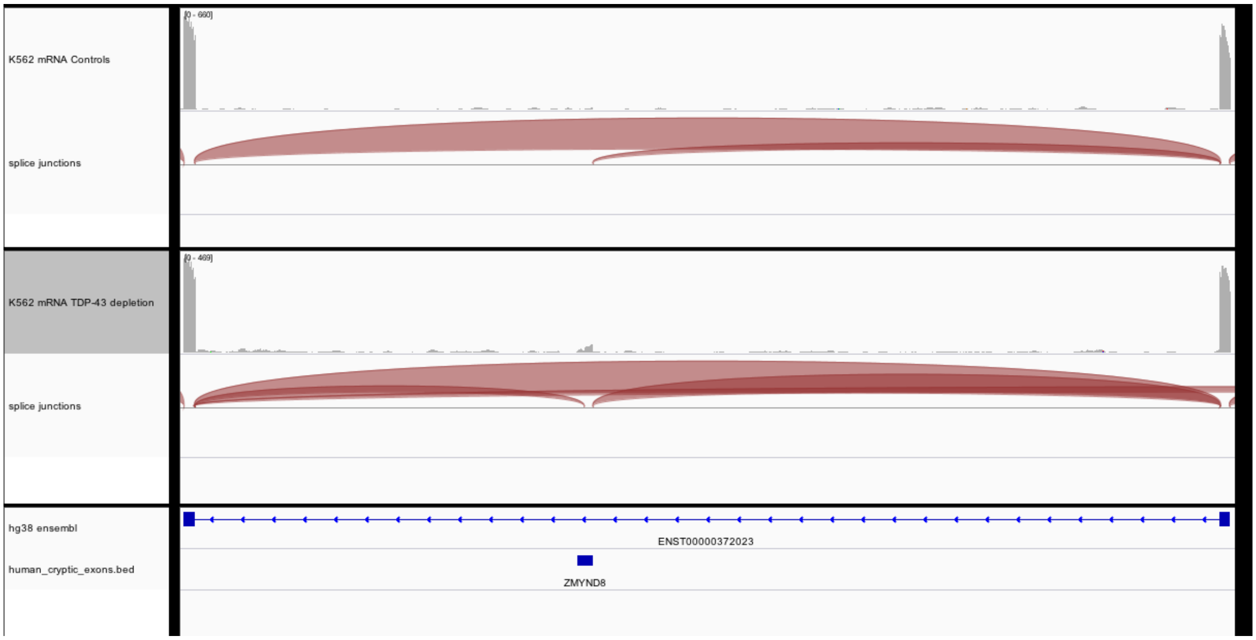

Human K562 total RNA

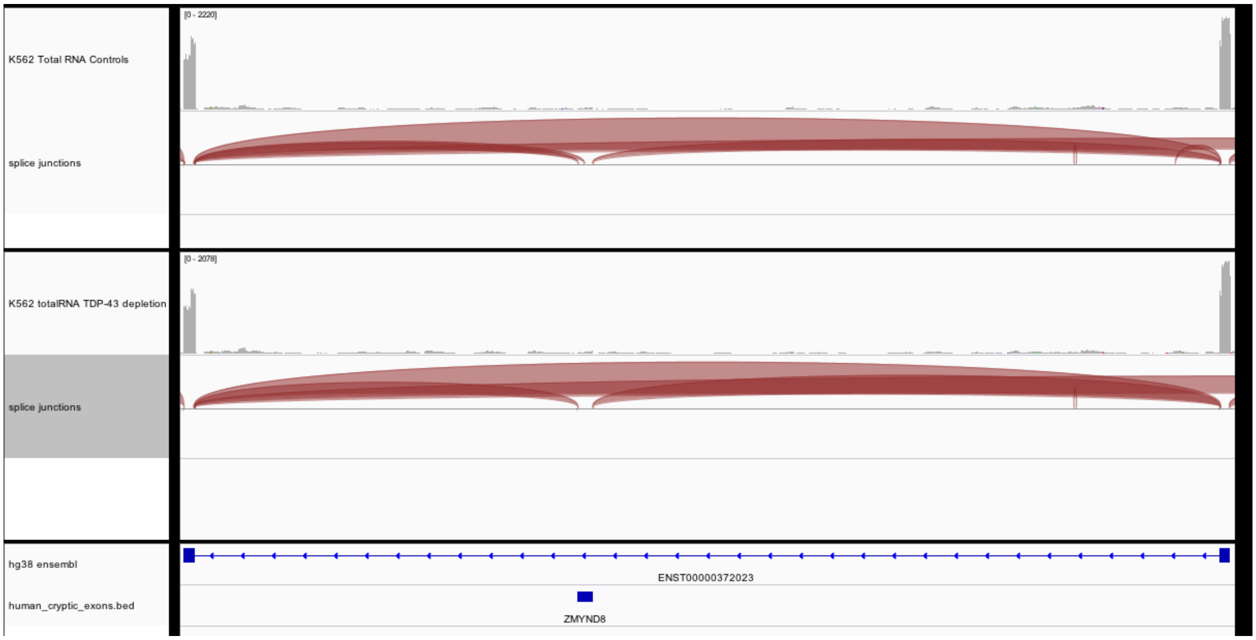

9 HEPH E030i1

Human K562 mRNA

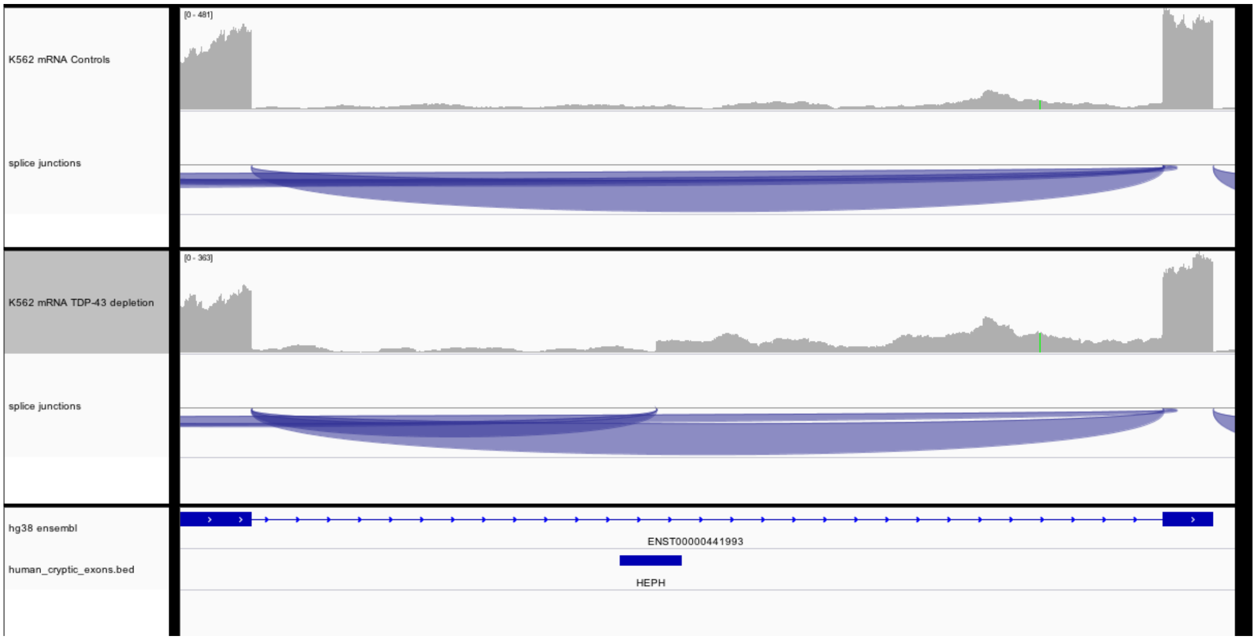

Human K562 total RNA

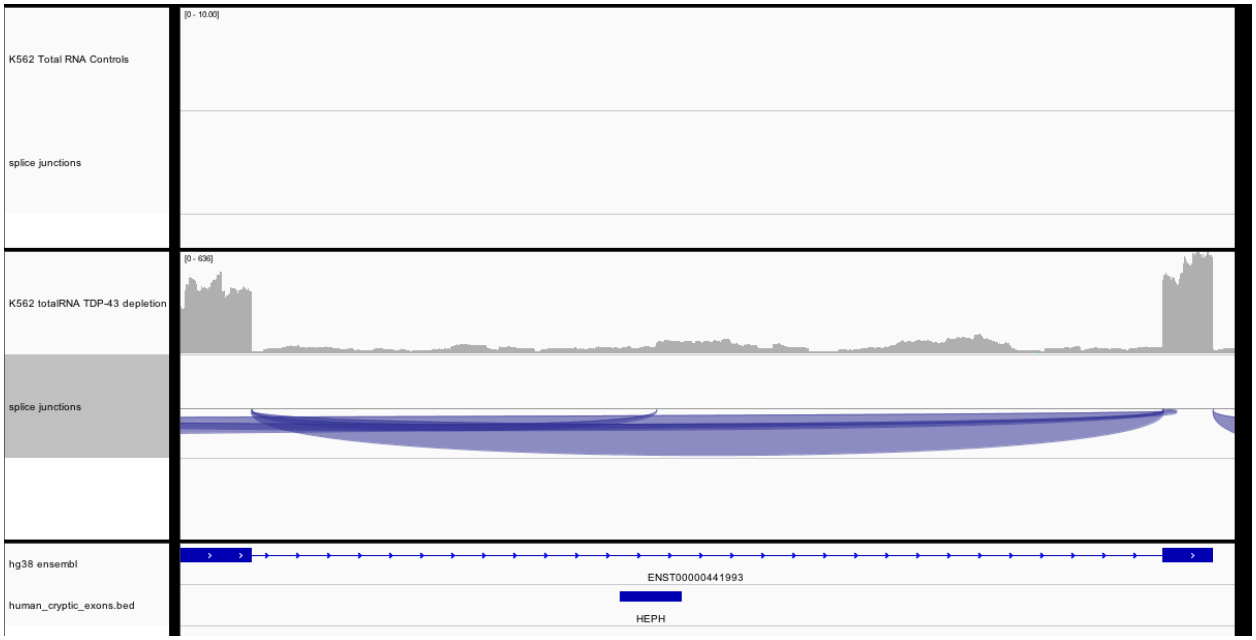

10 BTNL9 E031i1

Human K562 mRNA

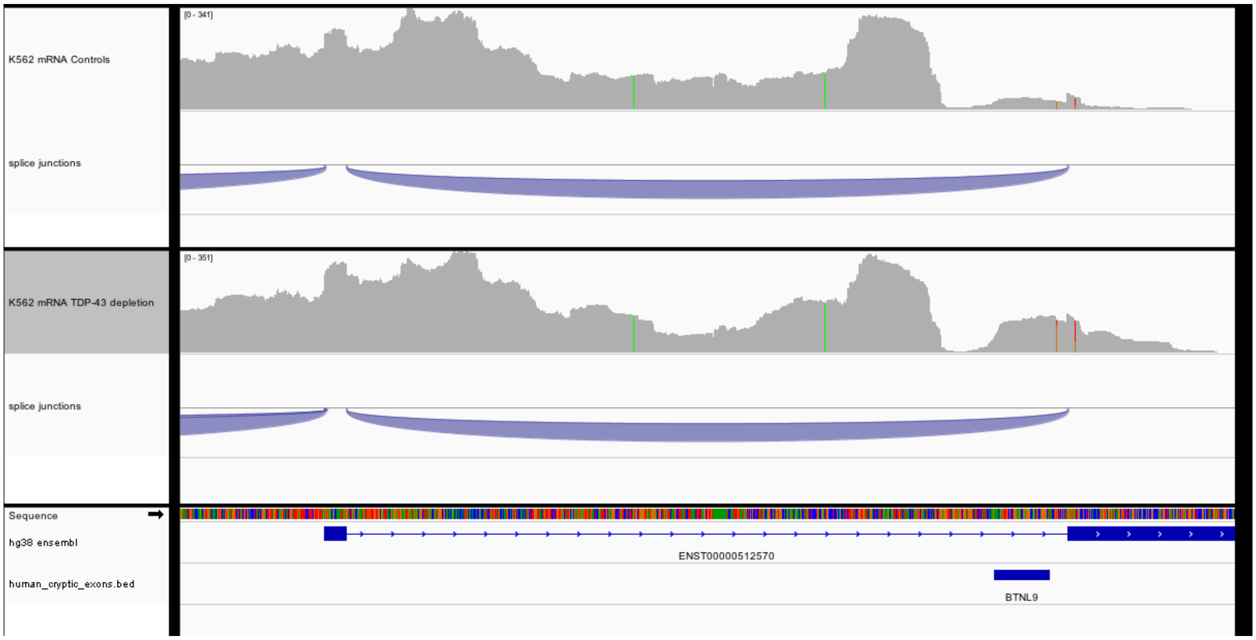

Human K562 total RNA

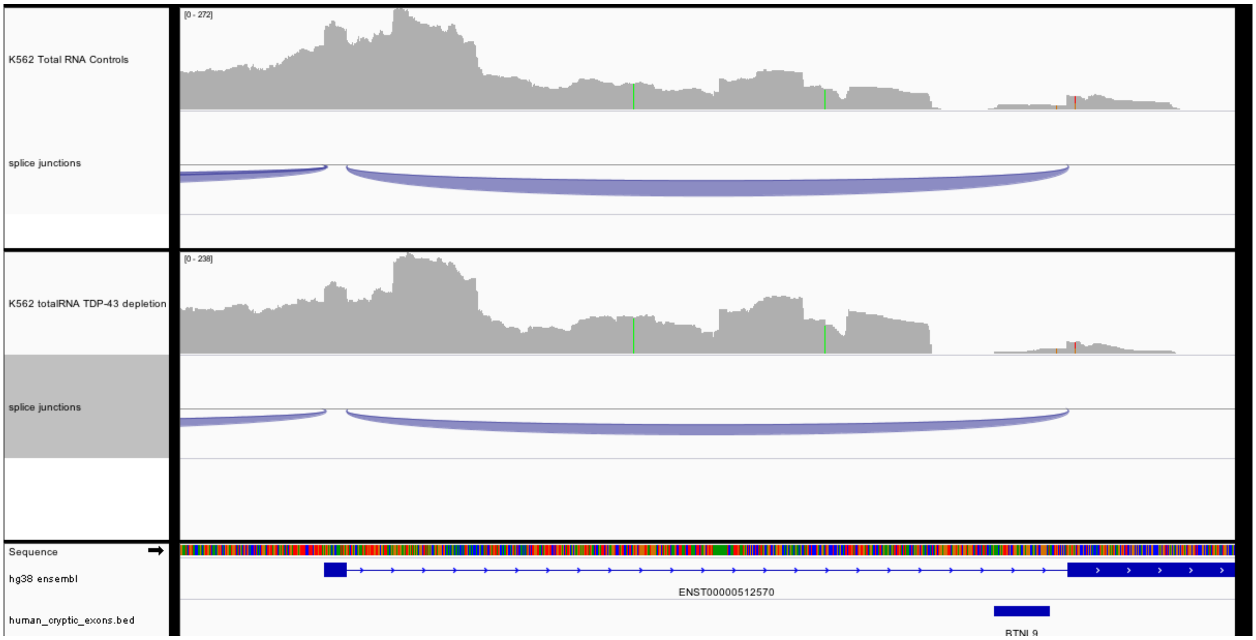

11 PAPSS1 E008i1

Human K562 mRNA

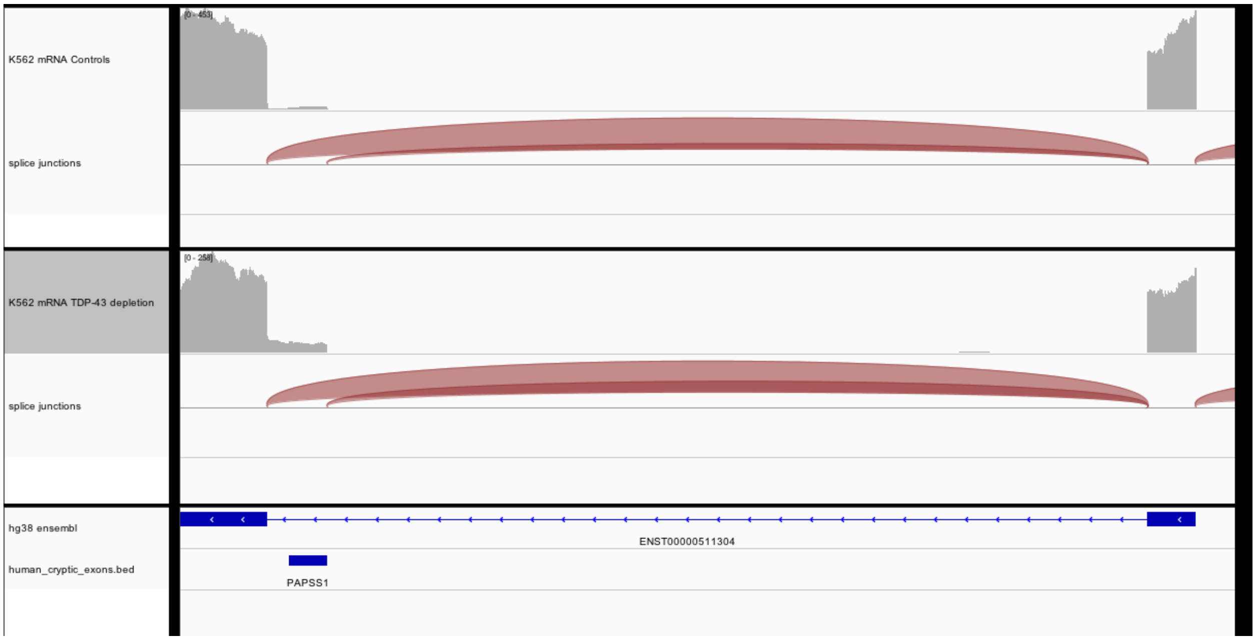

Human K562 total RNA

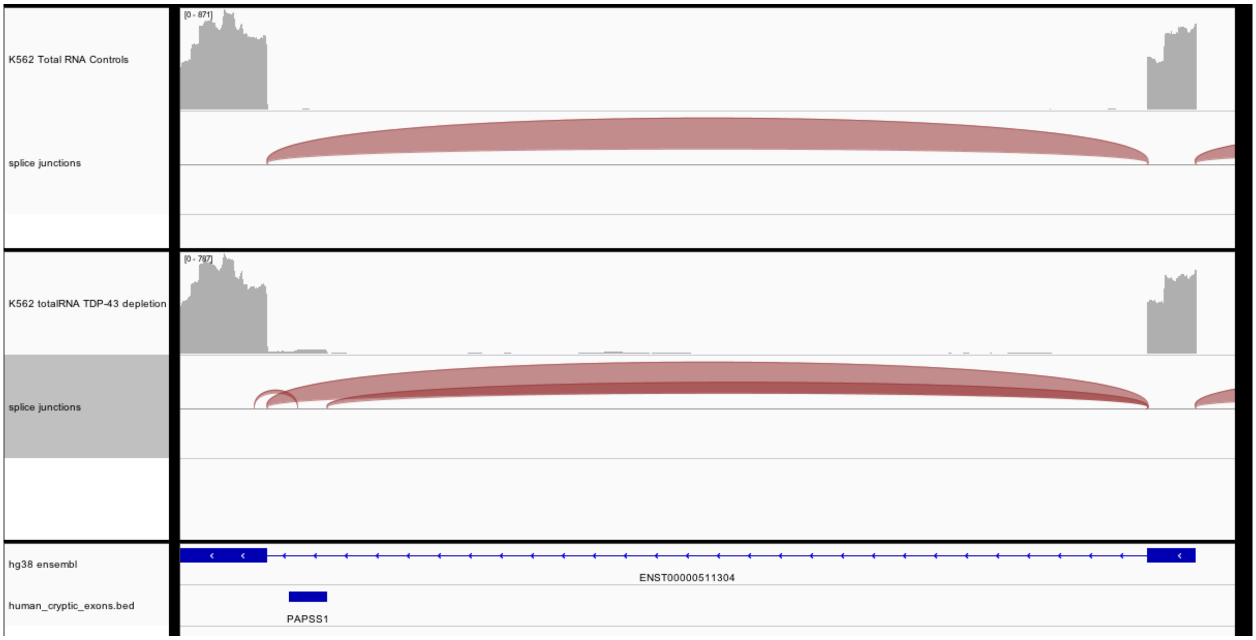

12 CLASP1 E049i1

Human K562 mRNA

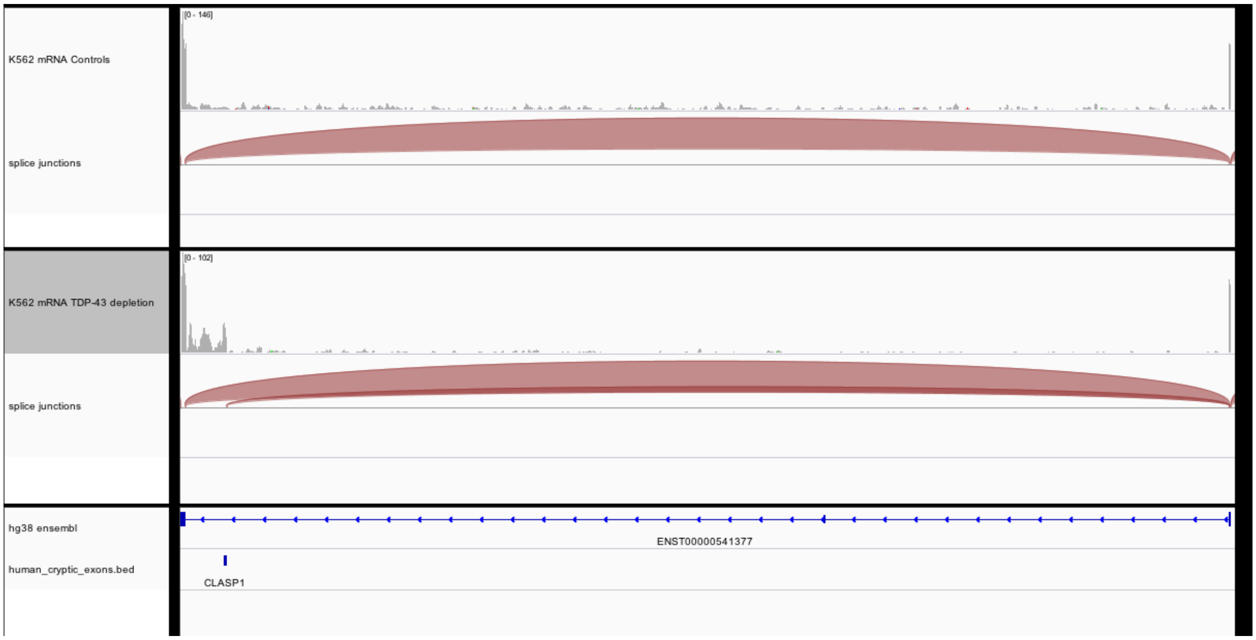

Human K562 total RNA

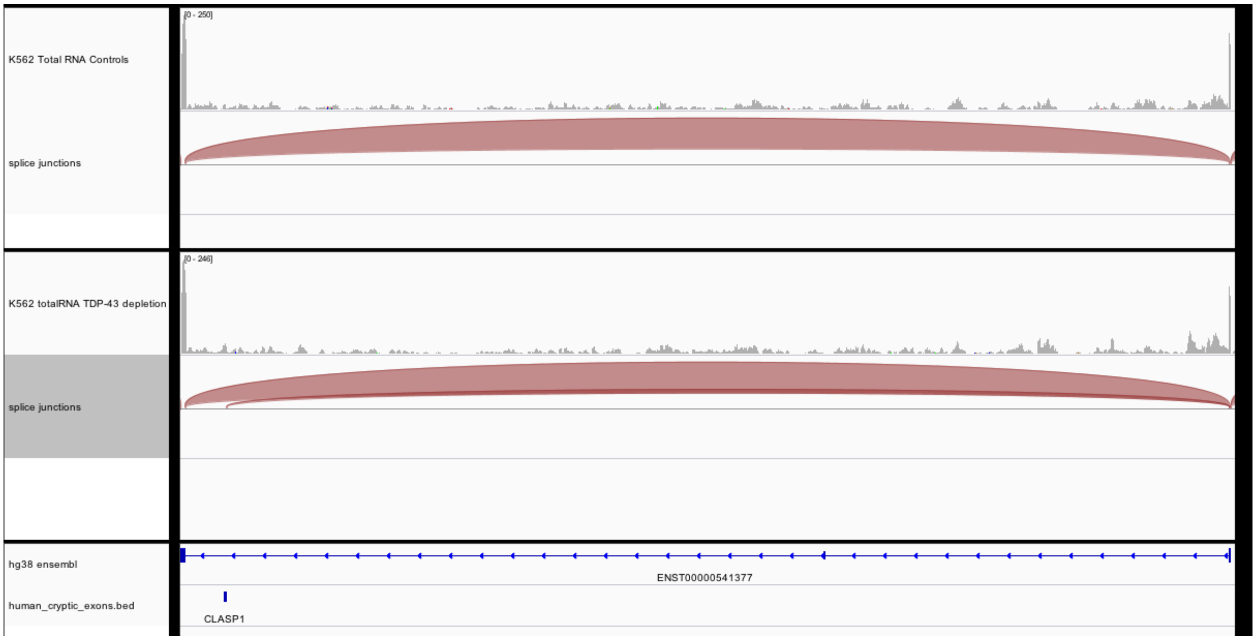

13 IST1 E053i1

Human K562 mRNA

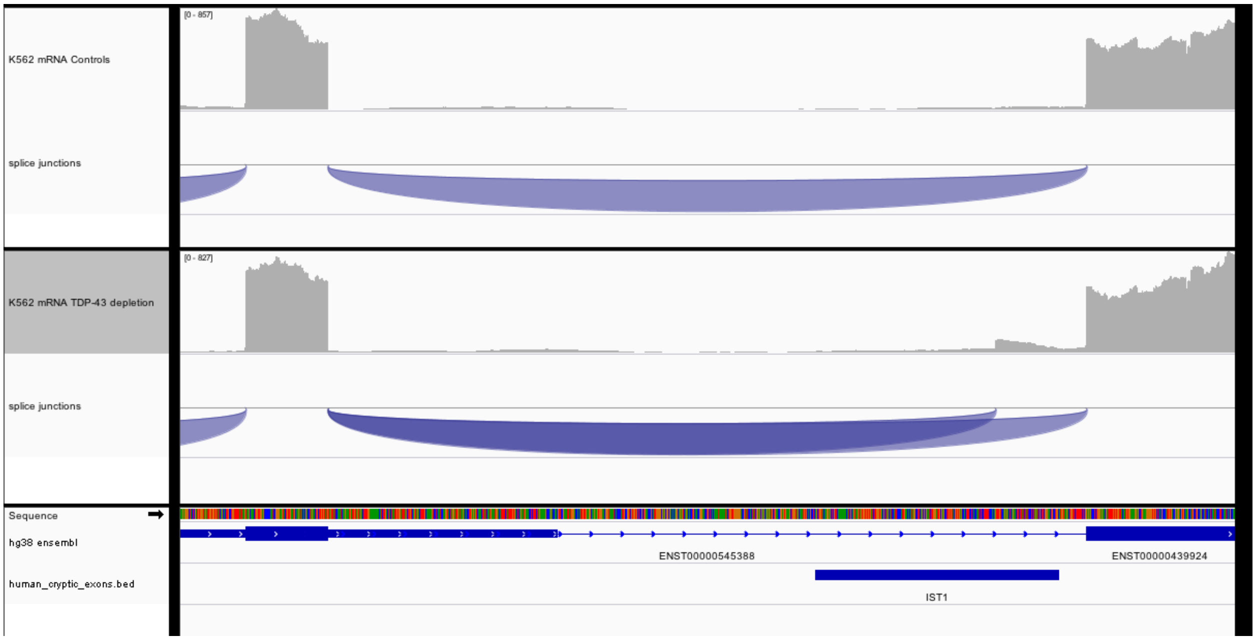

Human K562 total RNA

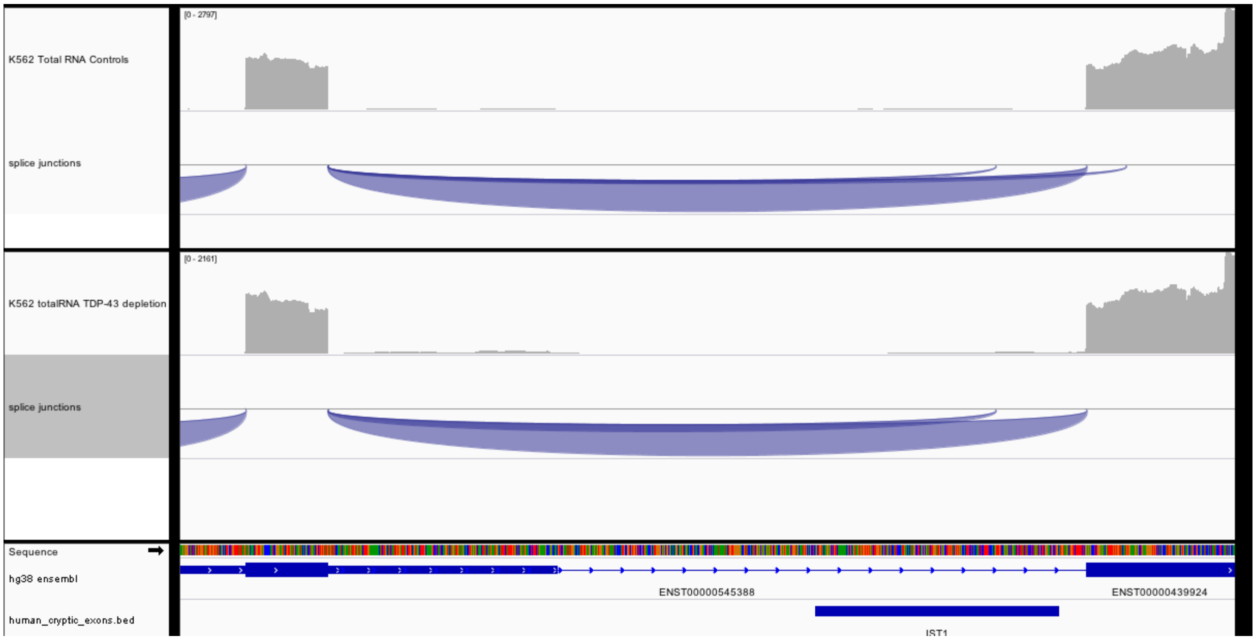

14 UBR4 E084i1

Human K562 mRNA

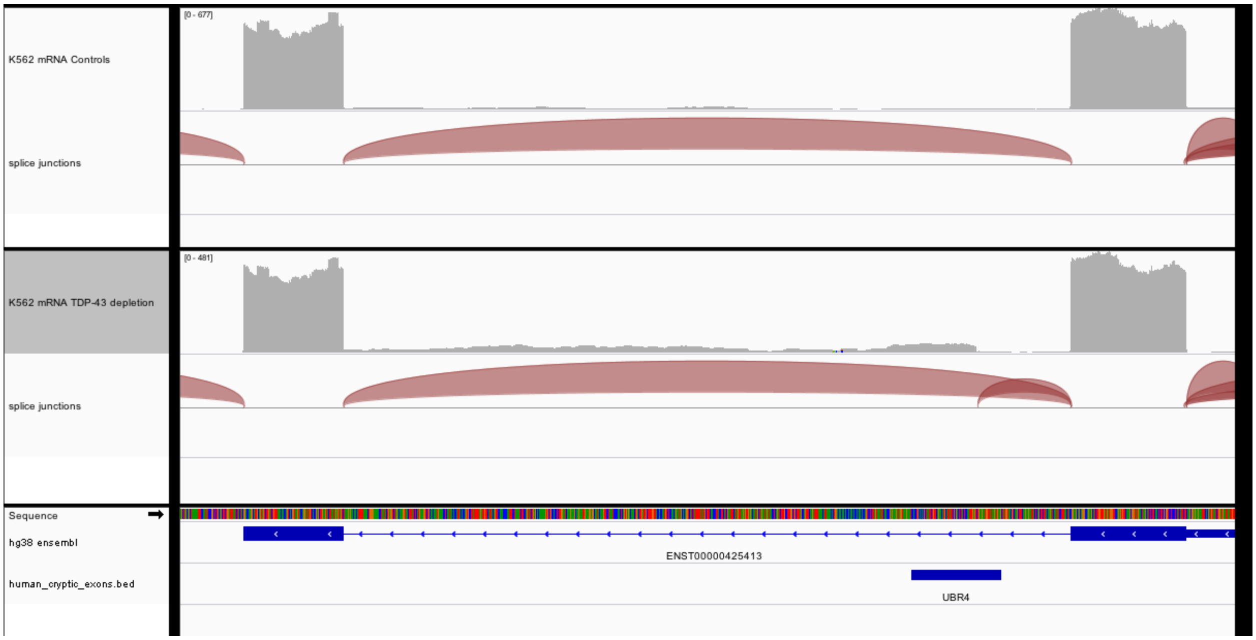

Human K562 total RNA

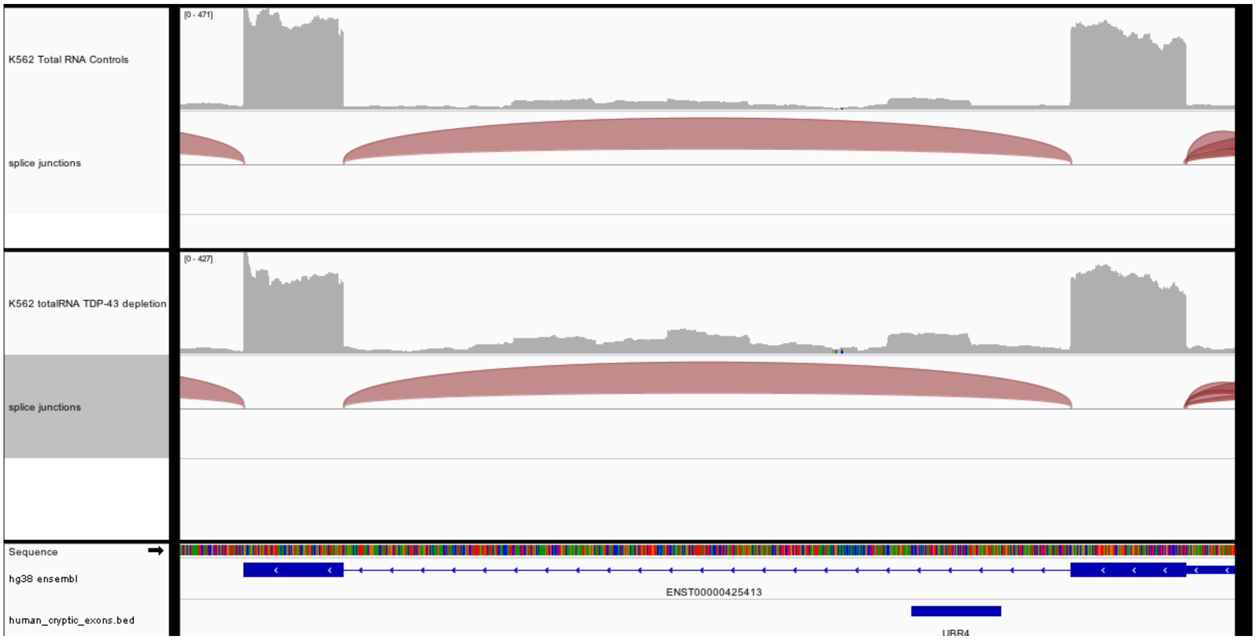

15 ANKRD27 E019i1

Human K562 mRNA

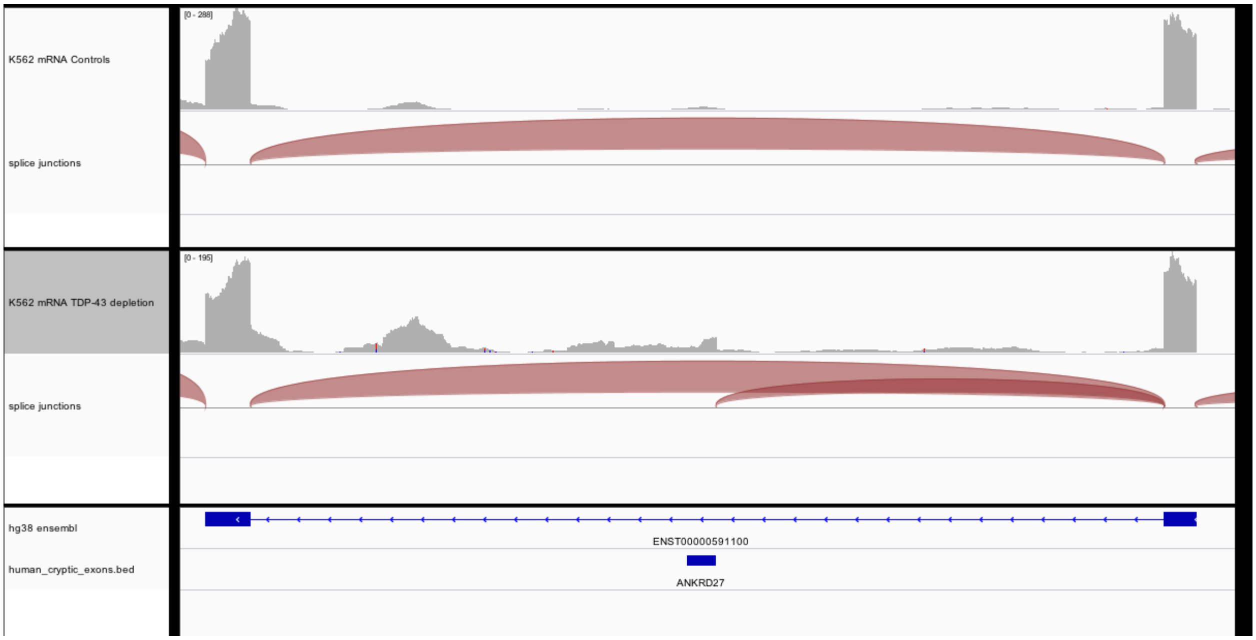

Human K562 total RNA

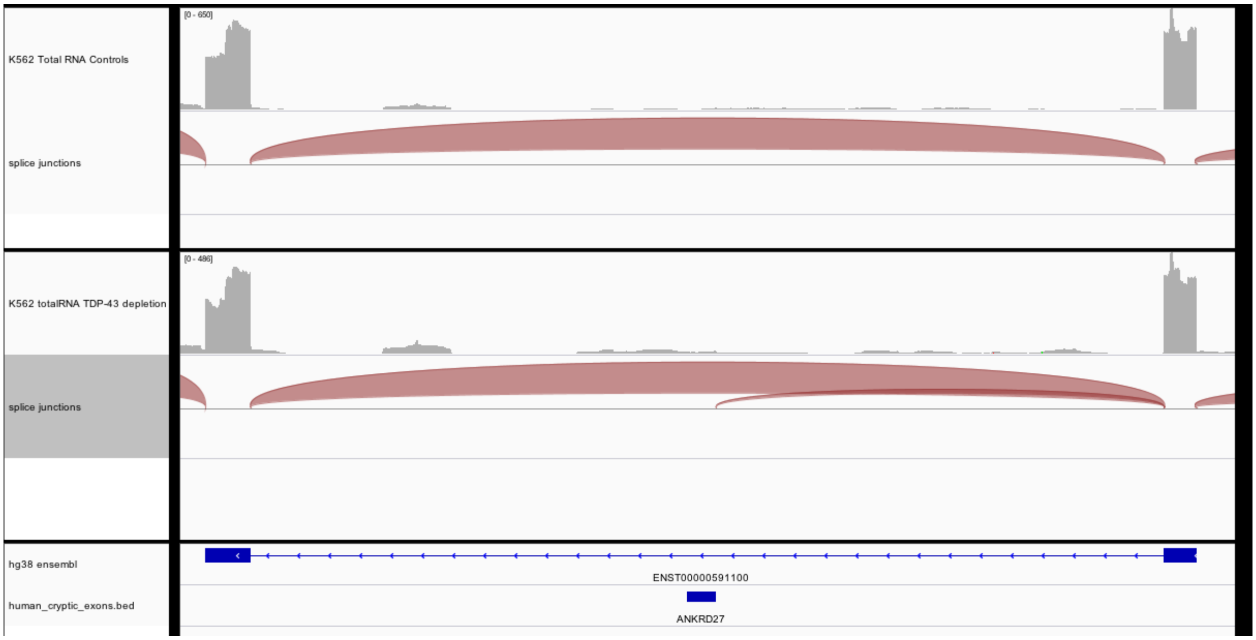

16 CCDC77 E020i1

Human K562 mRNA

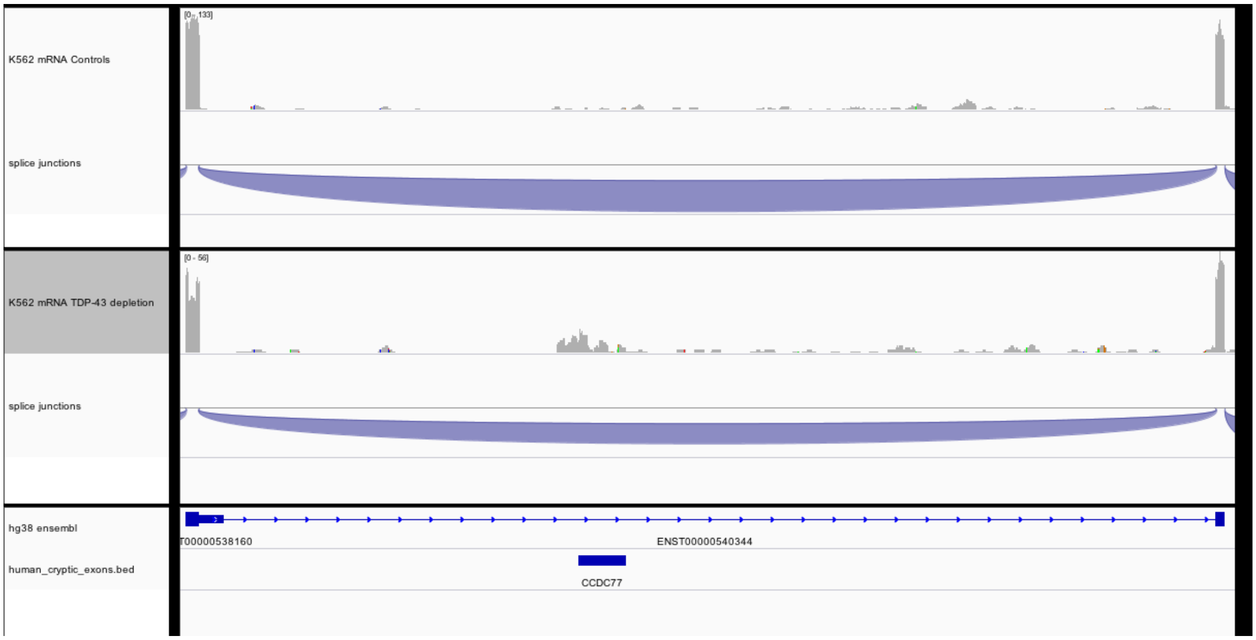

Human K562 total RNA

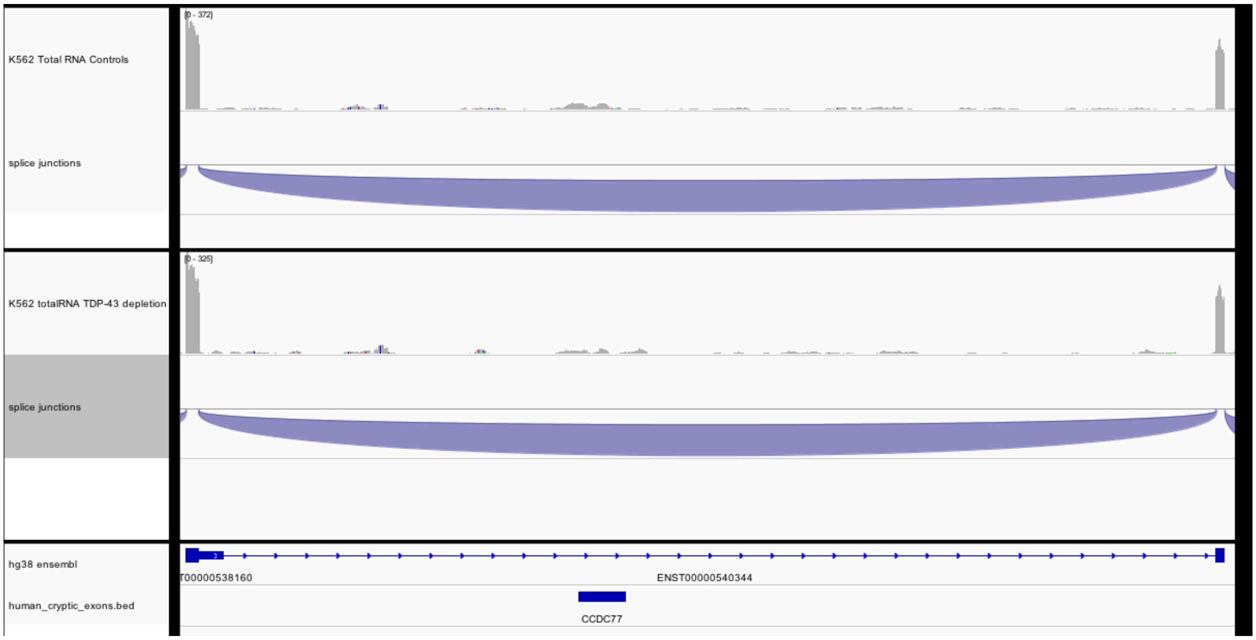

17 LRPPRC E024i1

Human K562 mRNA

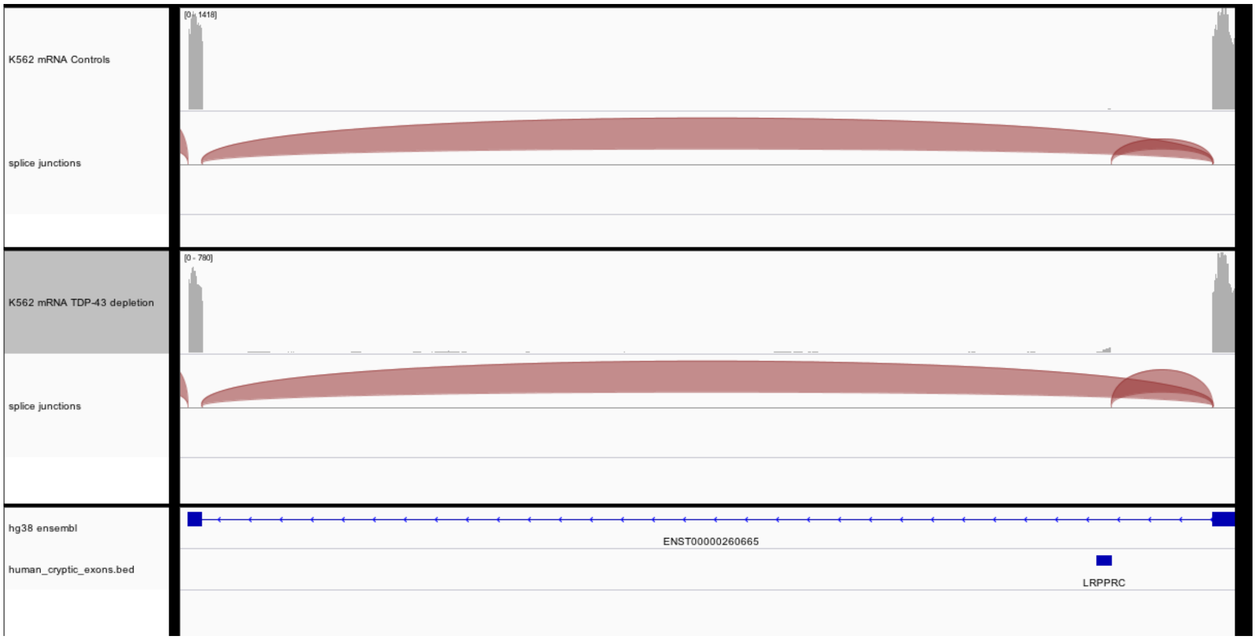

Human K562 total RNA

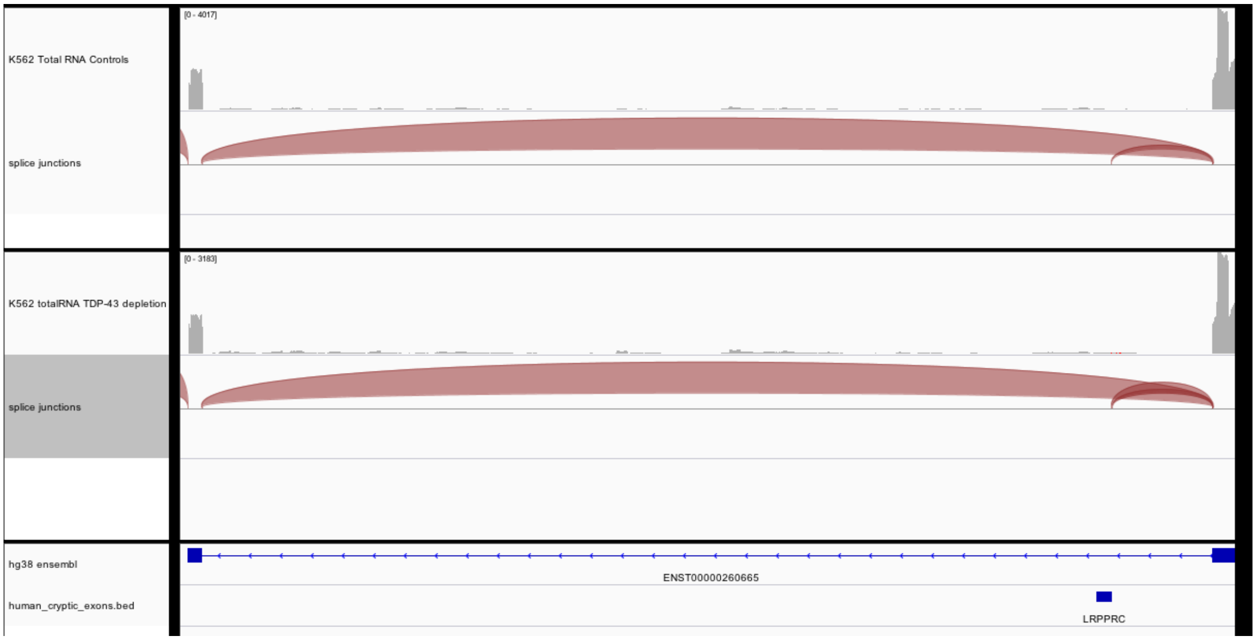

18 ADGRV1 E085i1

Human K562 mRNA

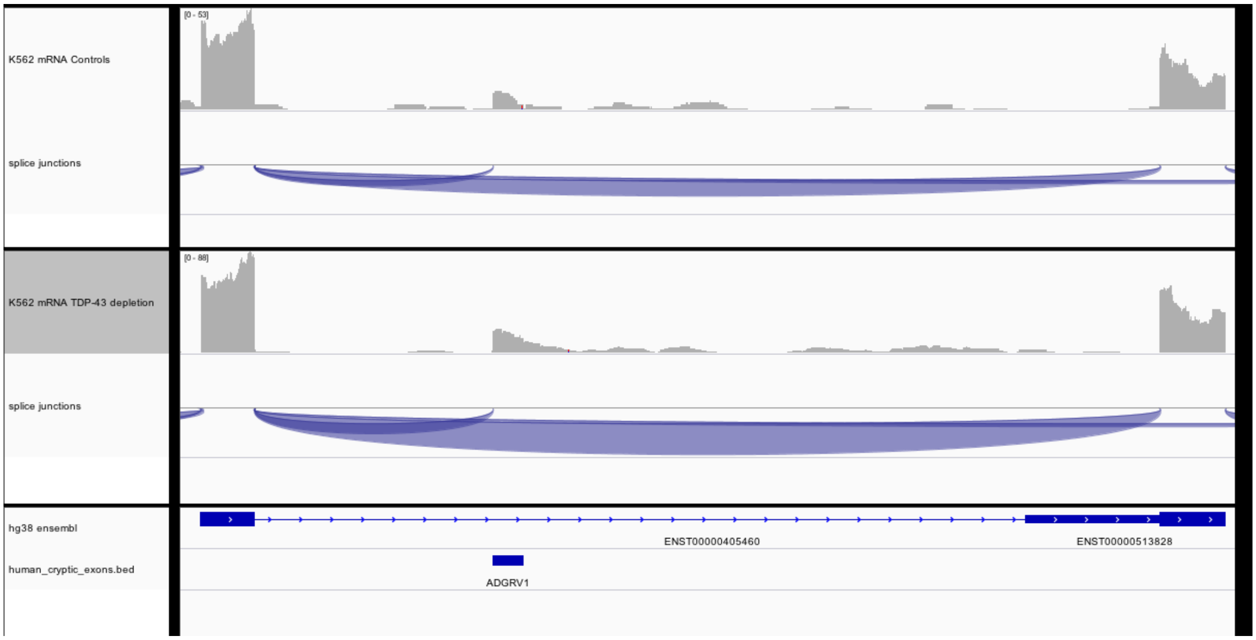

Human K562 total RNA

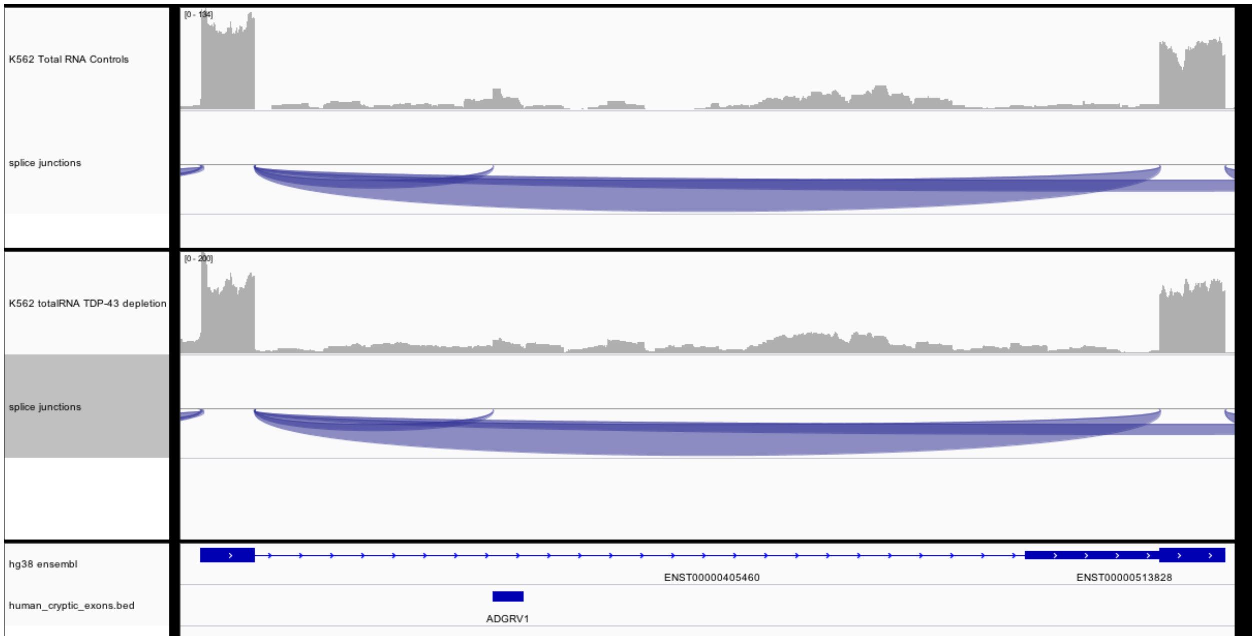

19 ASNSP1 E006i1

Human K562 mRNA

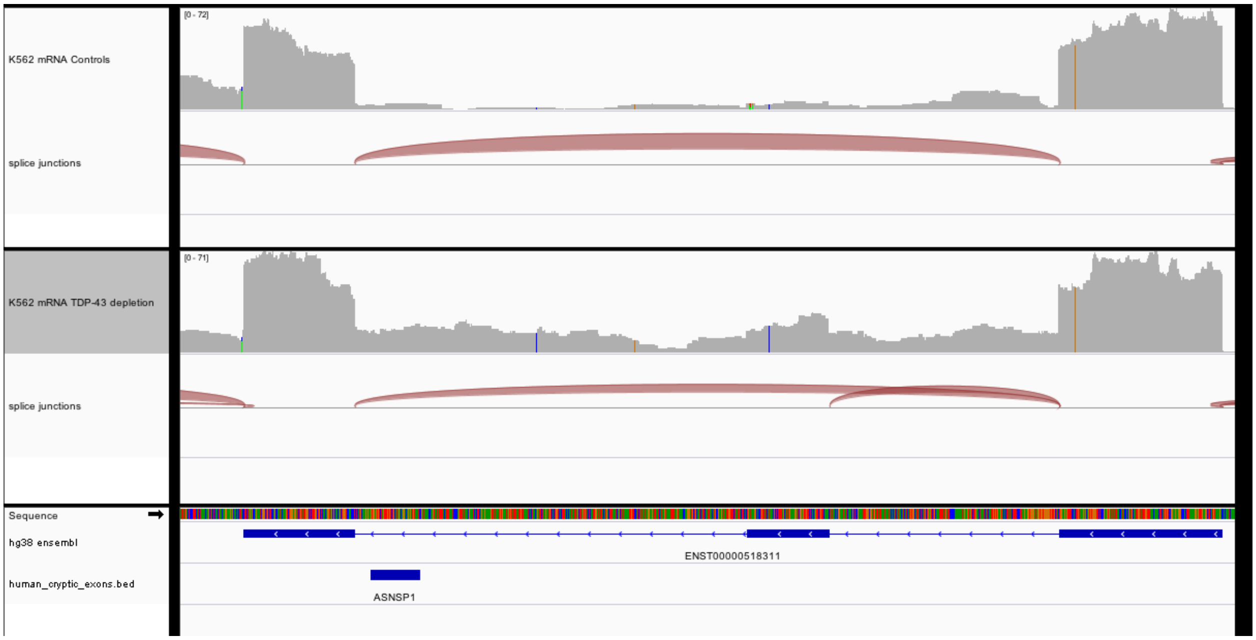

Human K562 total RNA

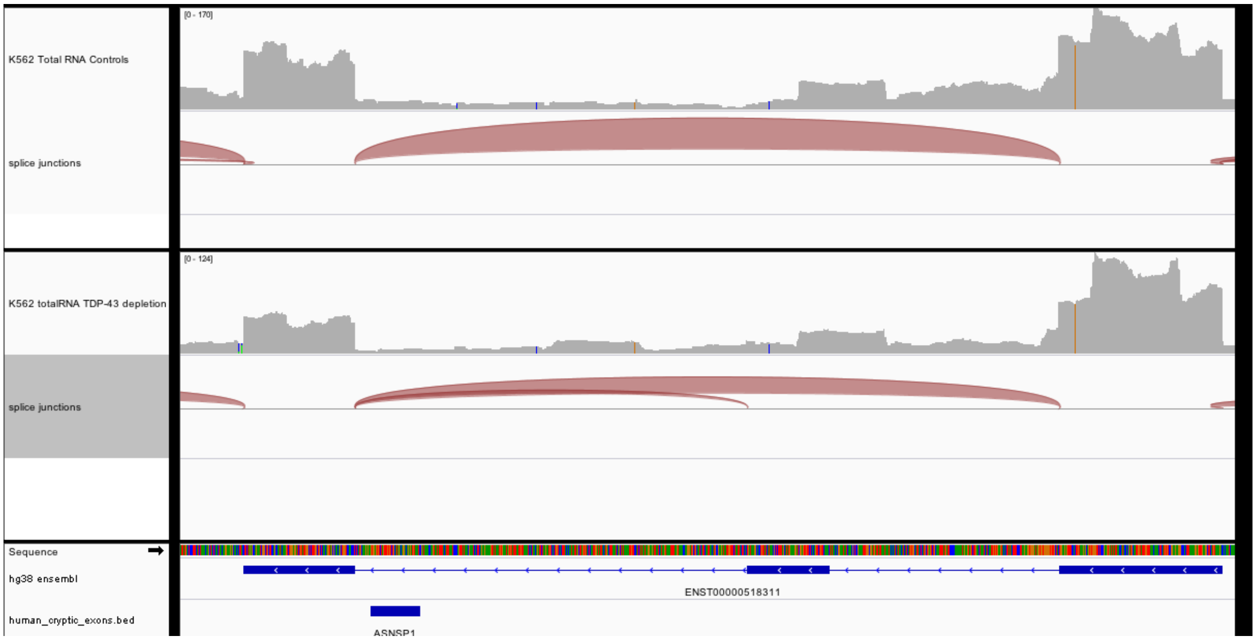

20 SMAD2 E010i1

Human K562 mRNA

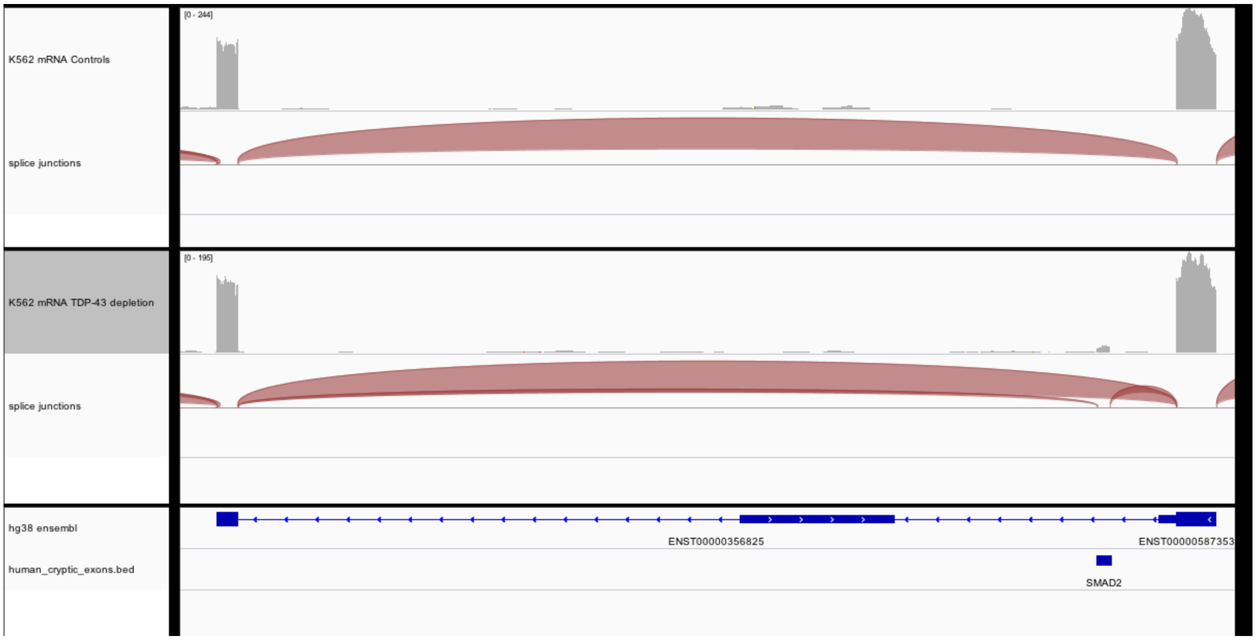

Human K562 total RNA

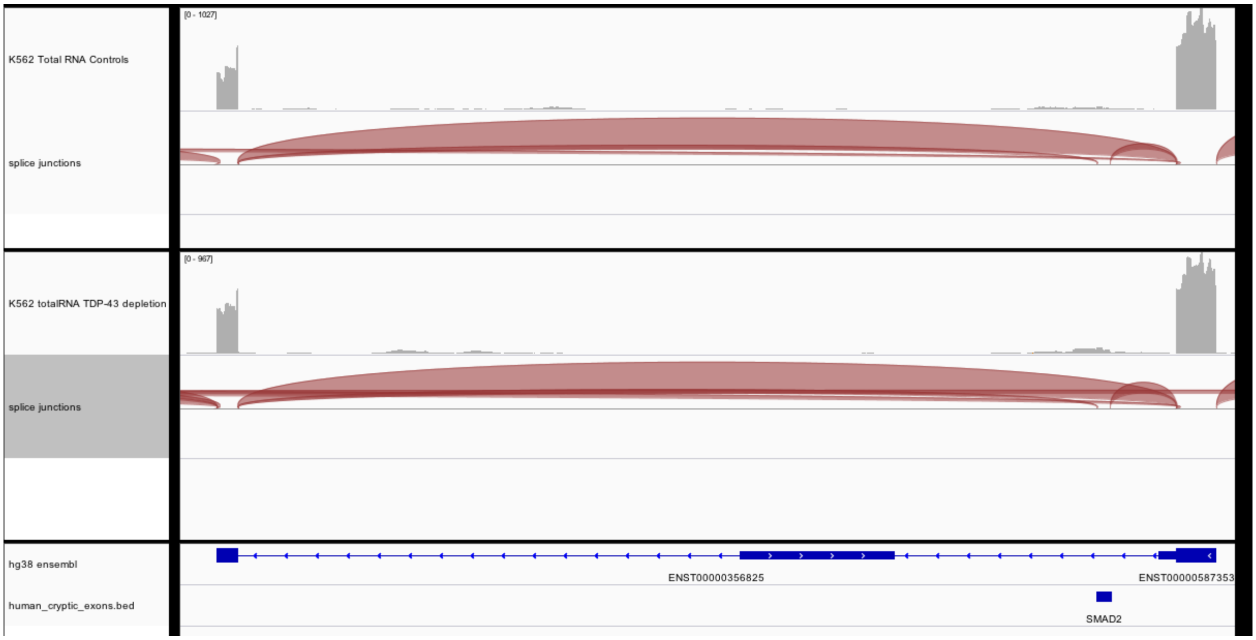

21 ZNHIT6 E003i1

Human K562 mRNA

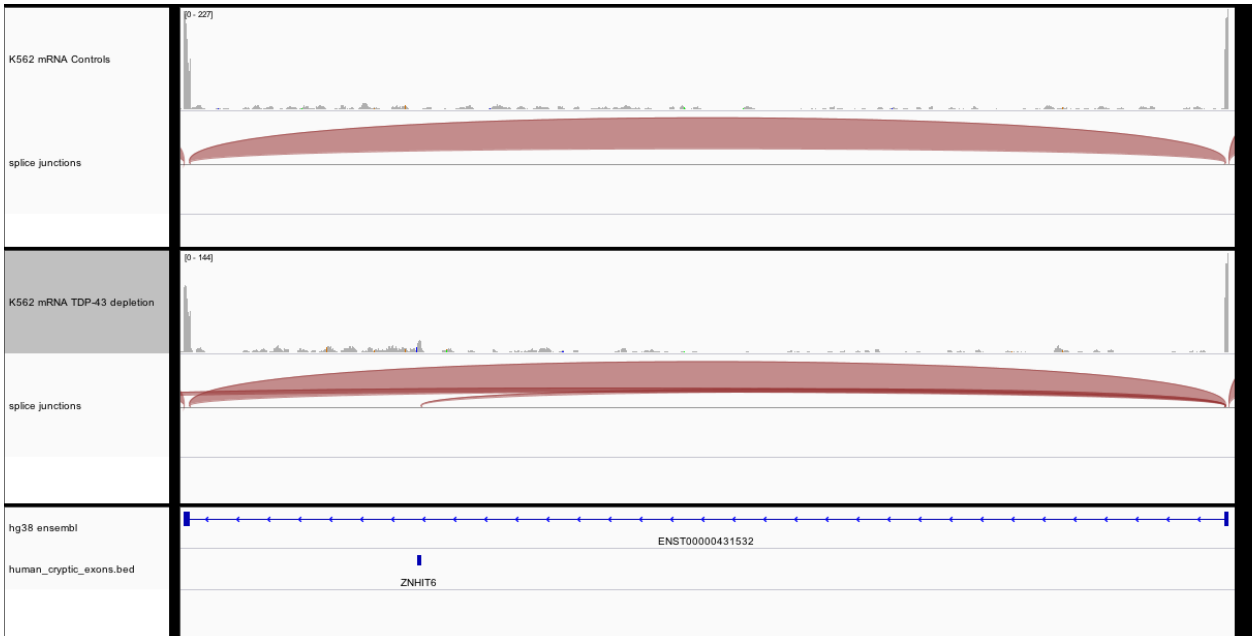

Human K562 total RNA

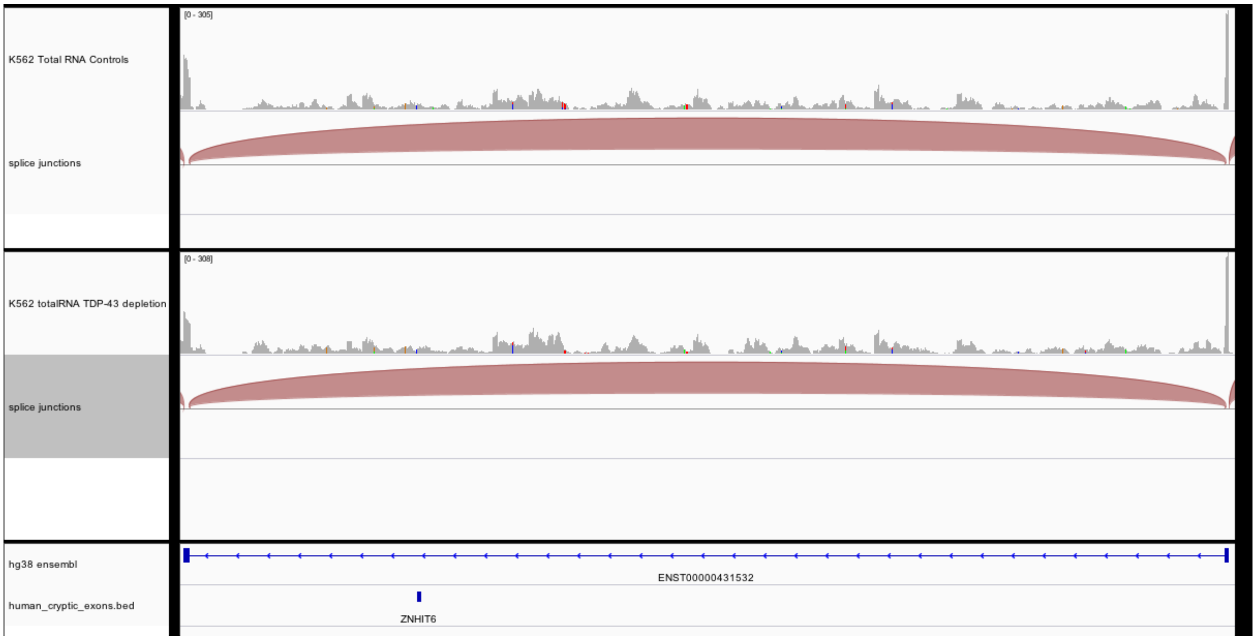

22 NLRP1 E045i1

Human K562 mRNA

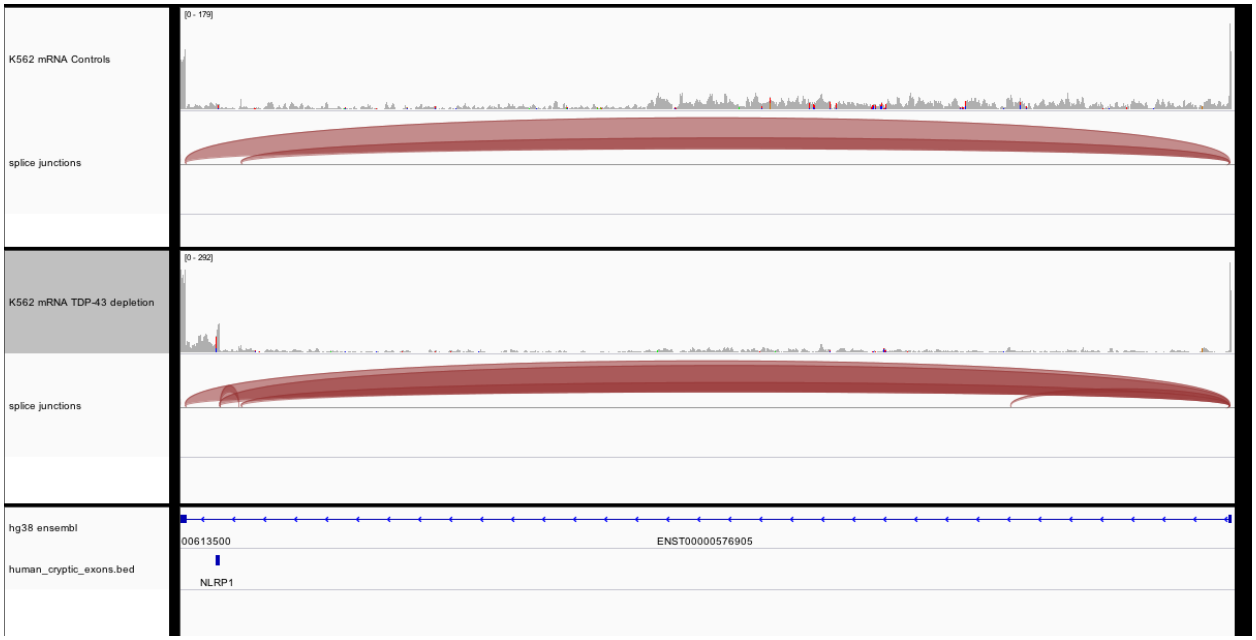

Human K562 total RNA

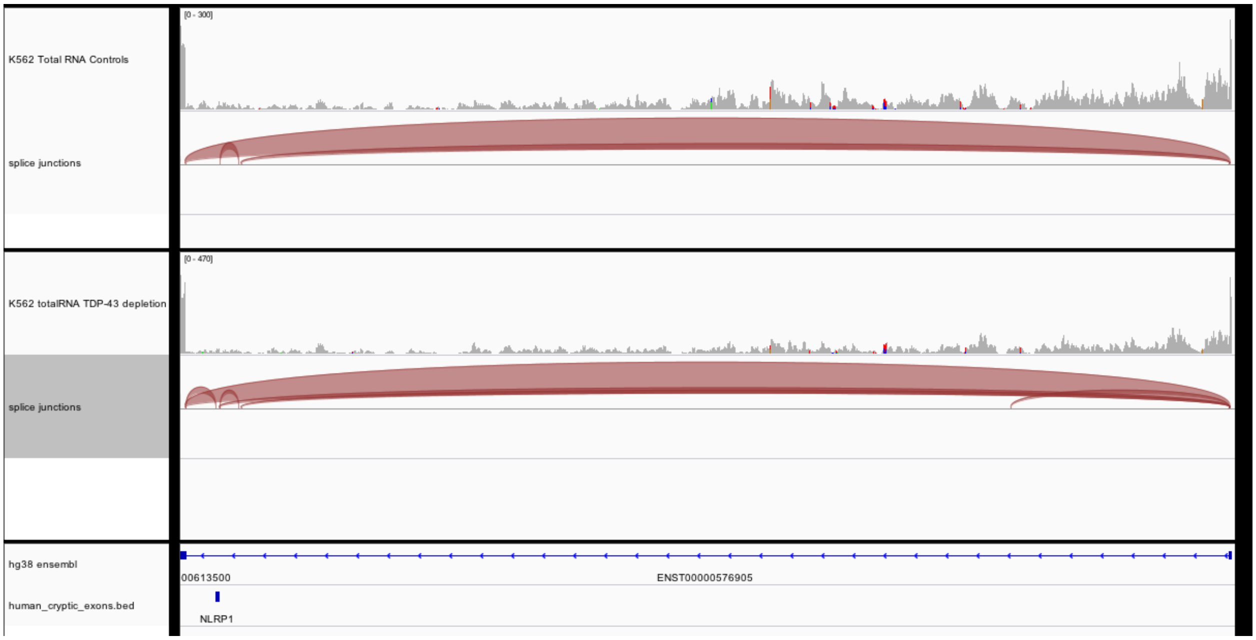

23 CEP290 E024i1

Human K562 mRNA

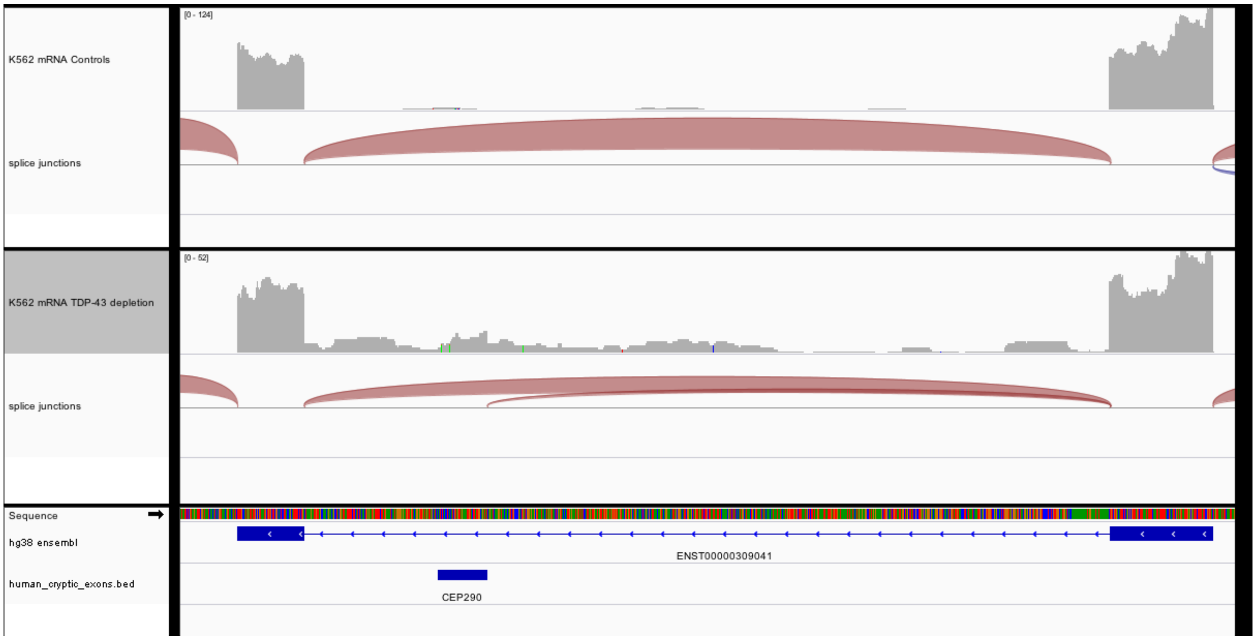

Human K562 total RNA

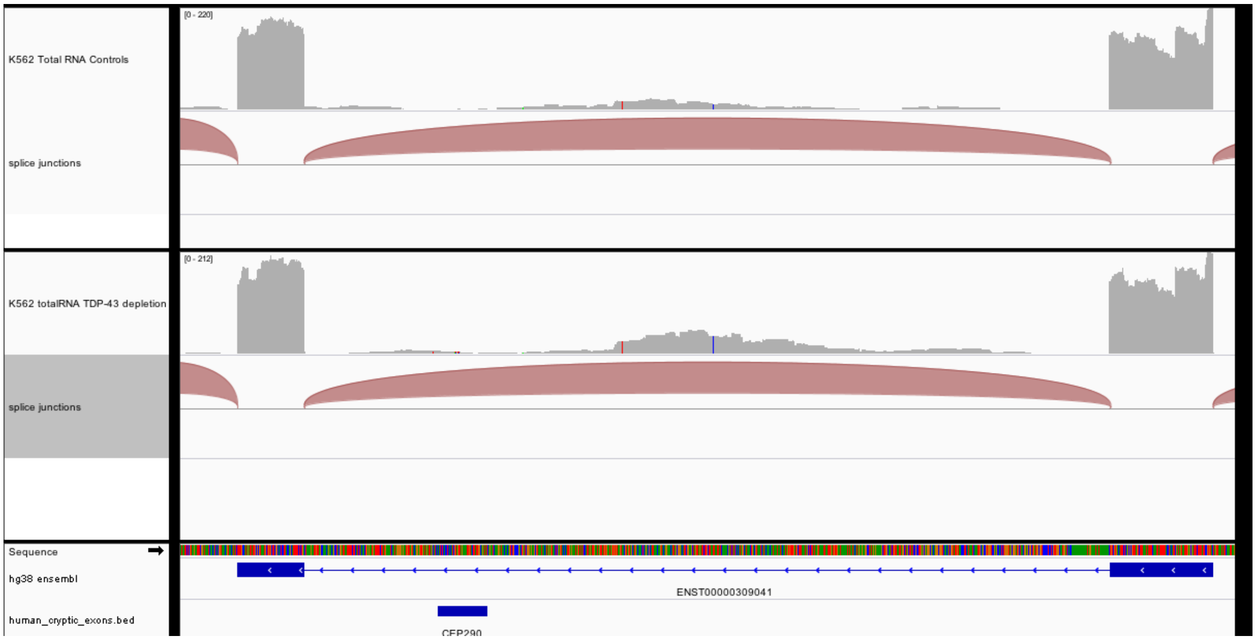

24 METTL8 E037i1

Human K562 mRNA

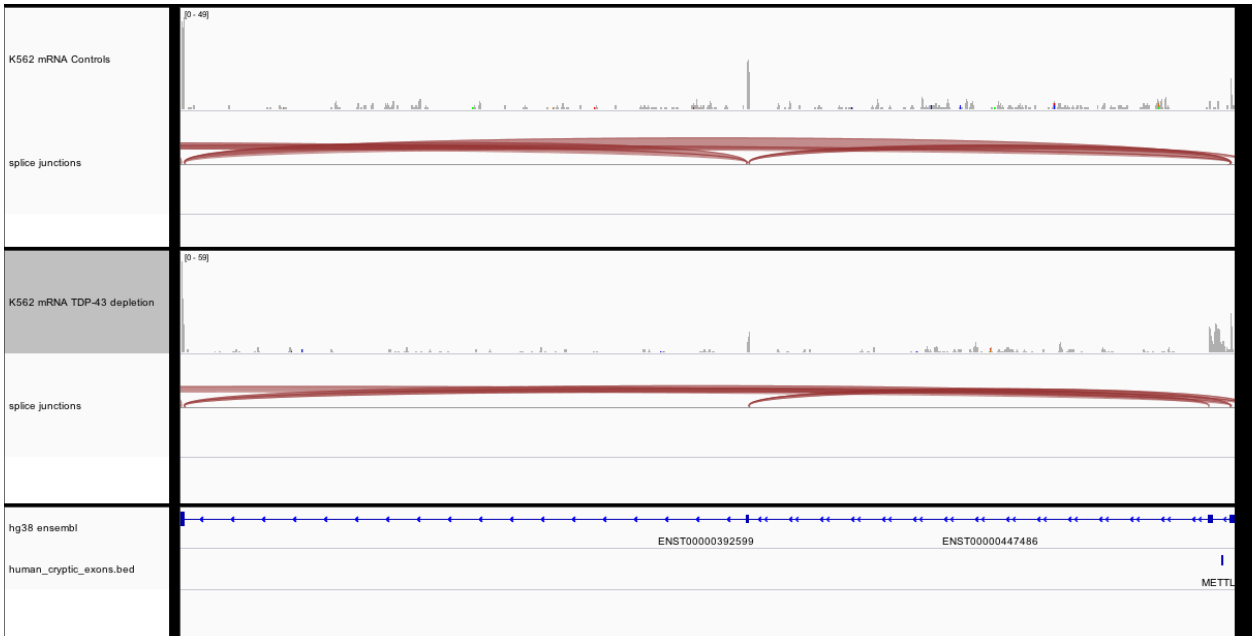

Human K562 total RNA

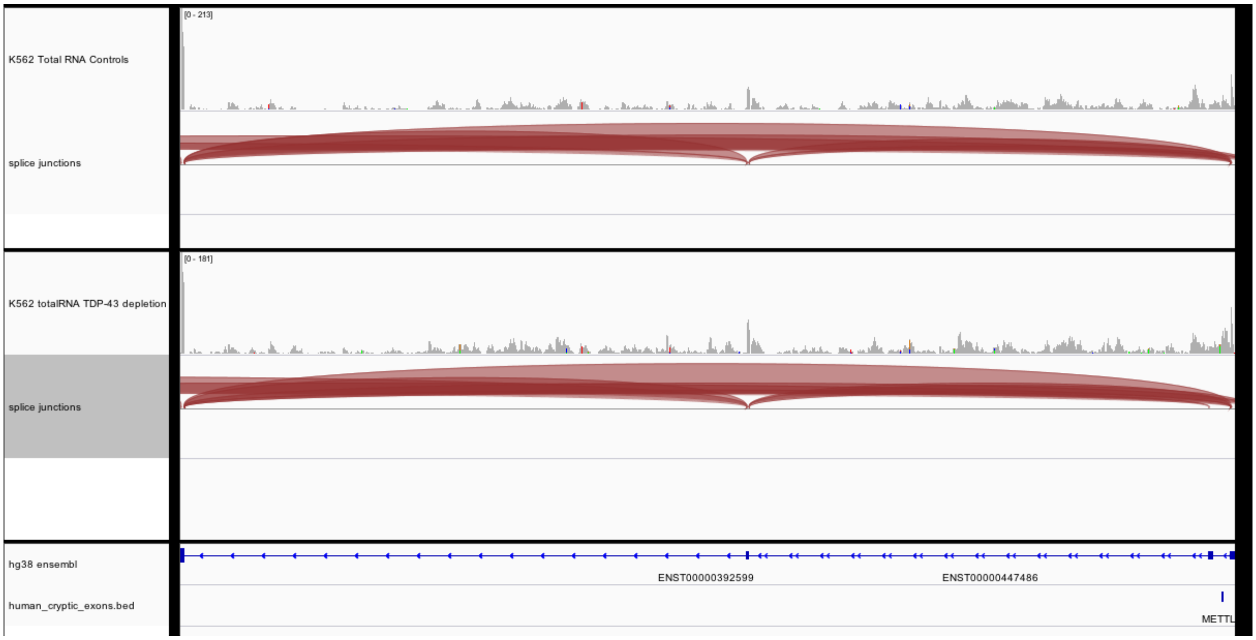

25 MPDZ E015i1

Human K562 mRNA

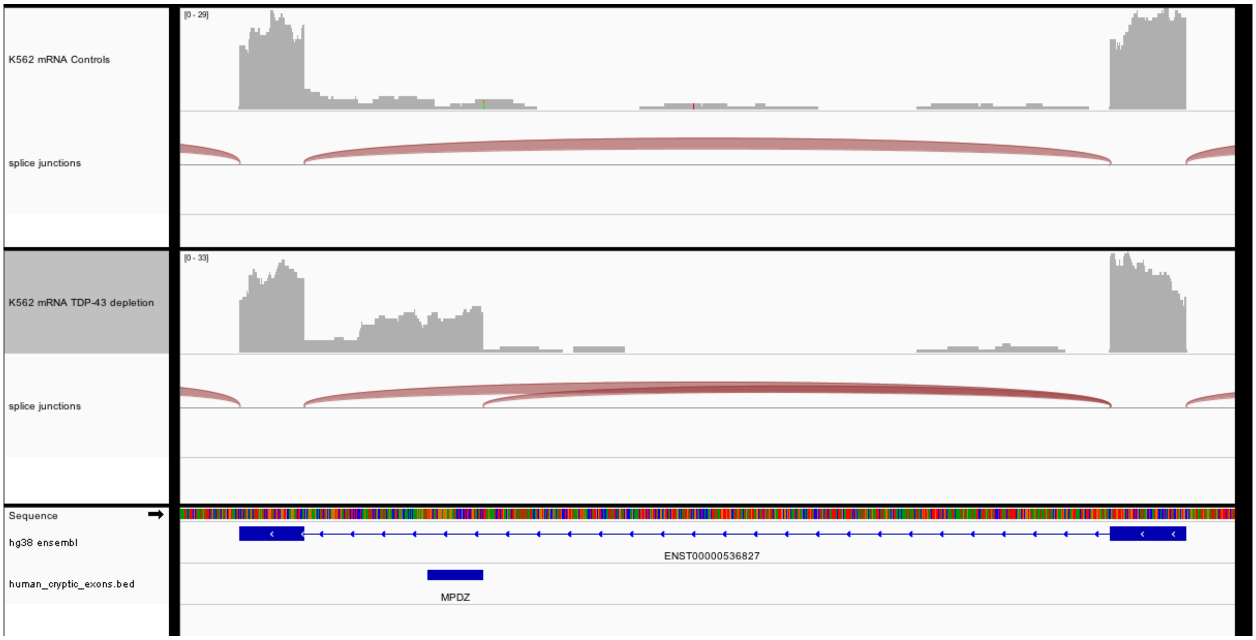

Human K562 total RNA

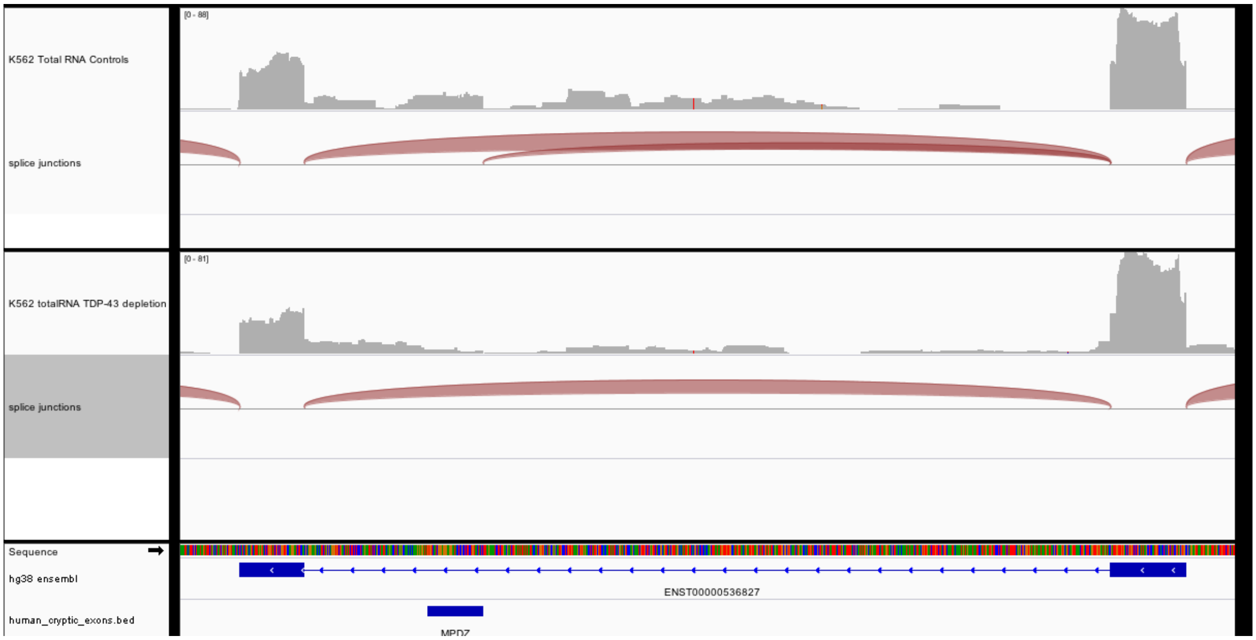

26 ERBB2IP E013i2

Human K562 mRNA

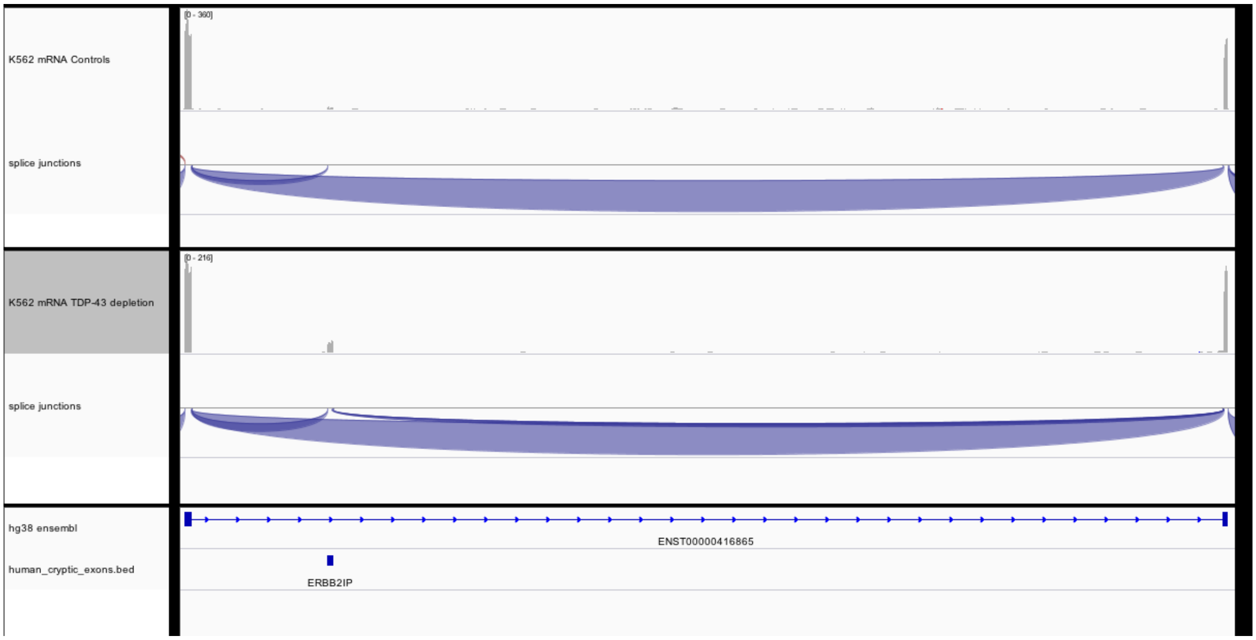

Human K562 total RNA

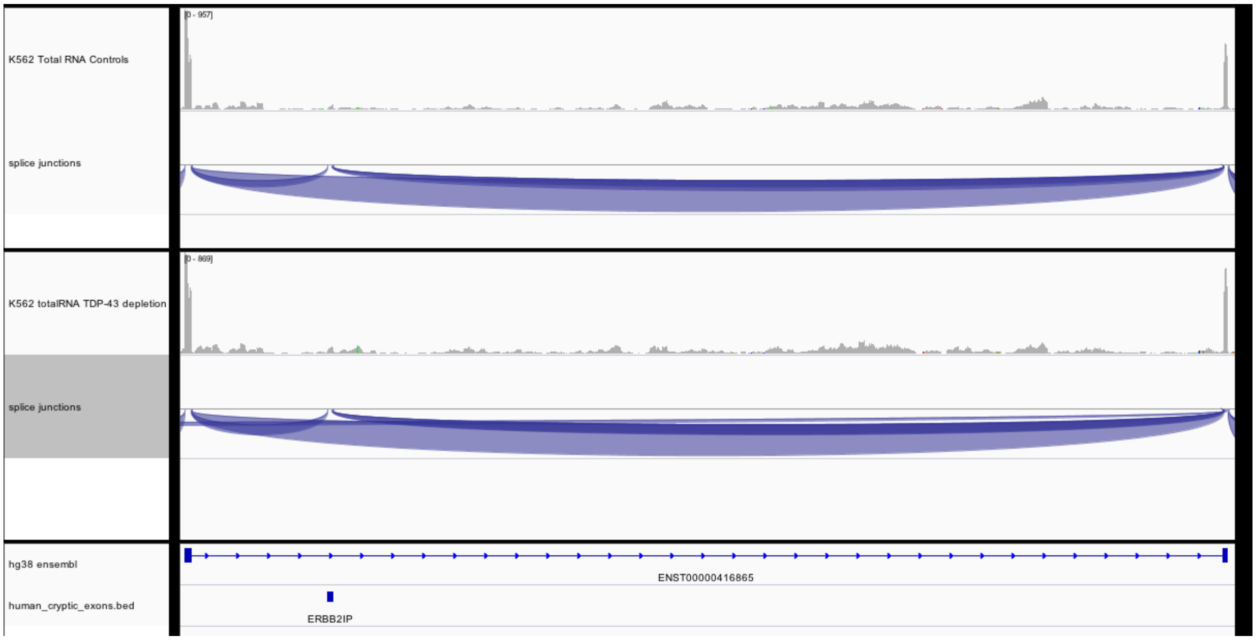

27 PRPF40A E001i1

Human K562 mRNA

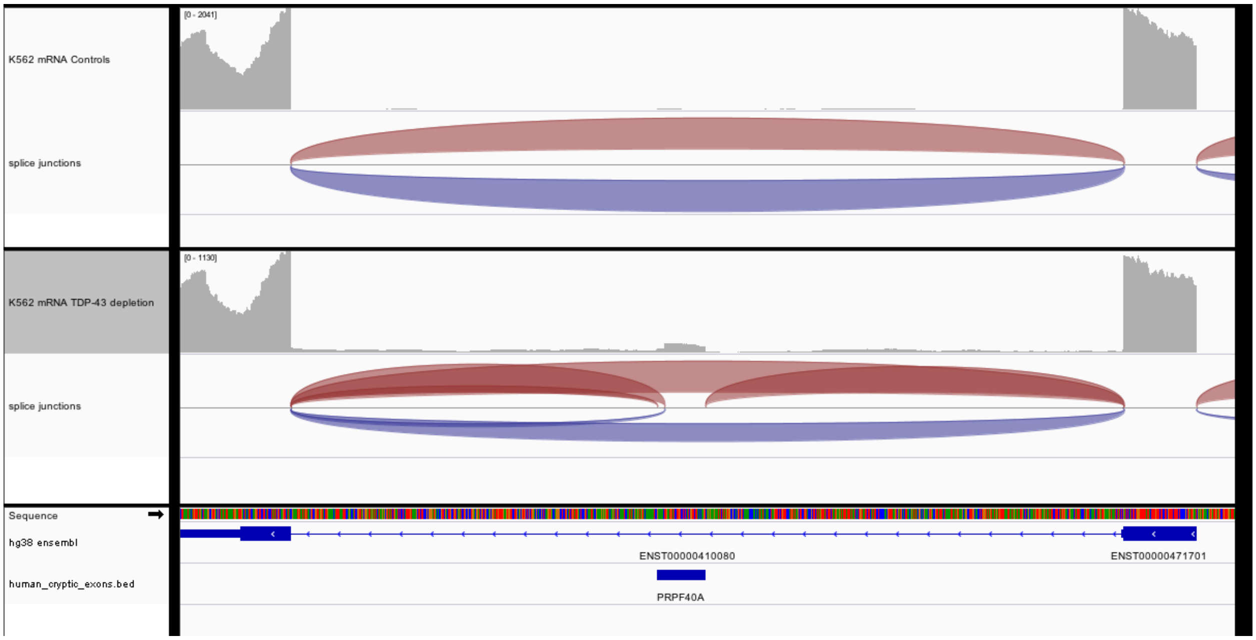

Human K562 total RNA

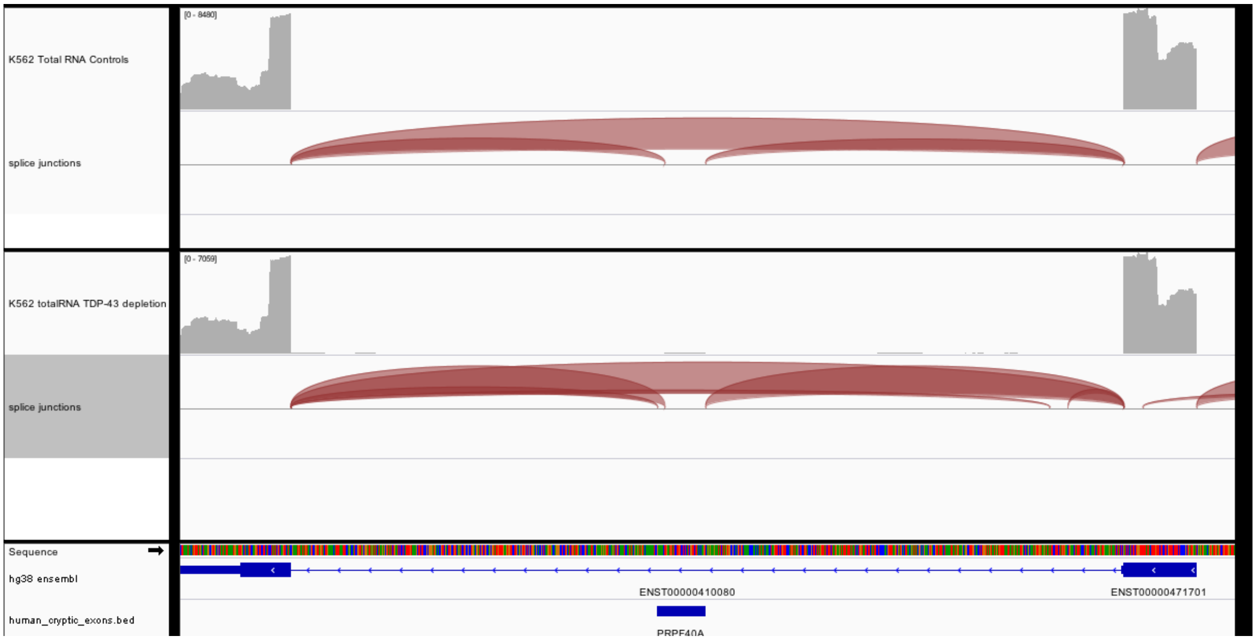

28 UPF2 E002i1

Human K562 mRNA

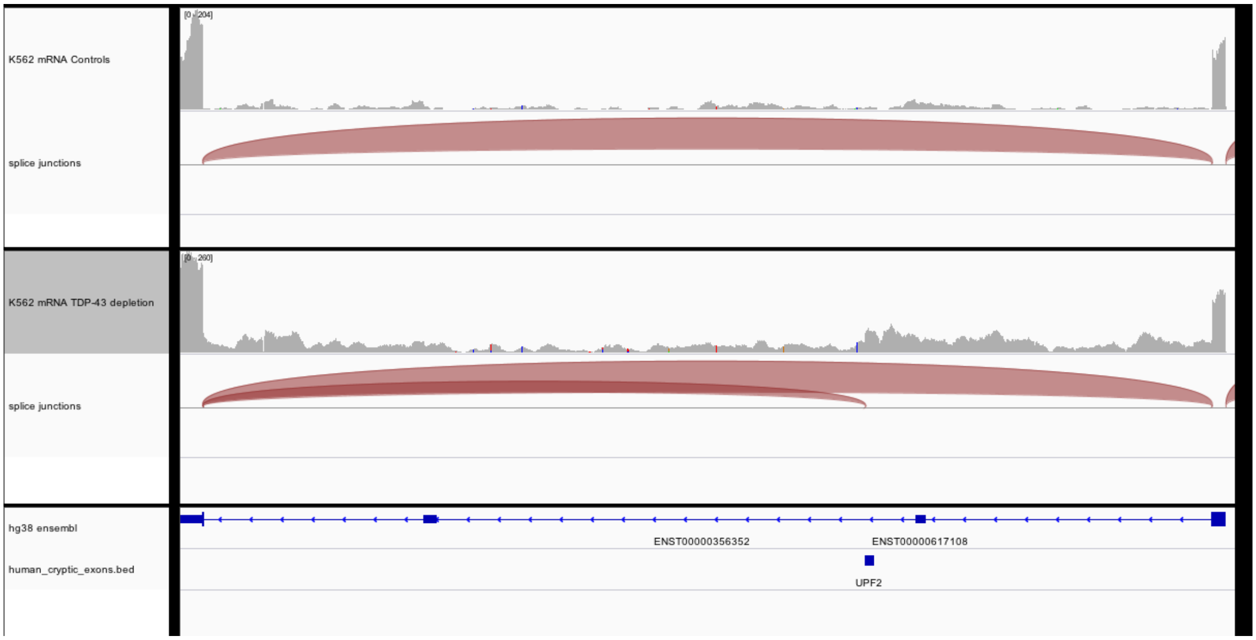

Human K562 total RNA

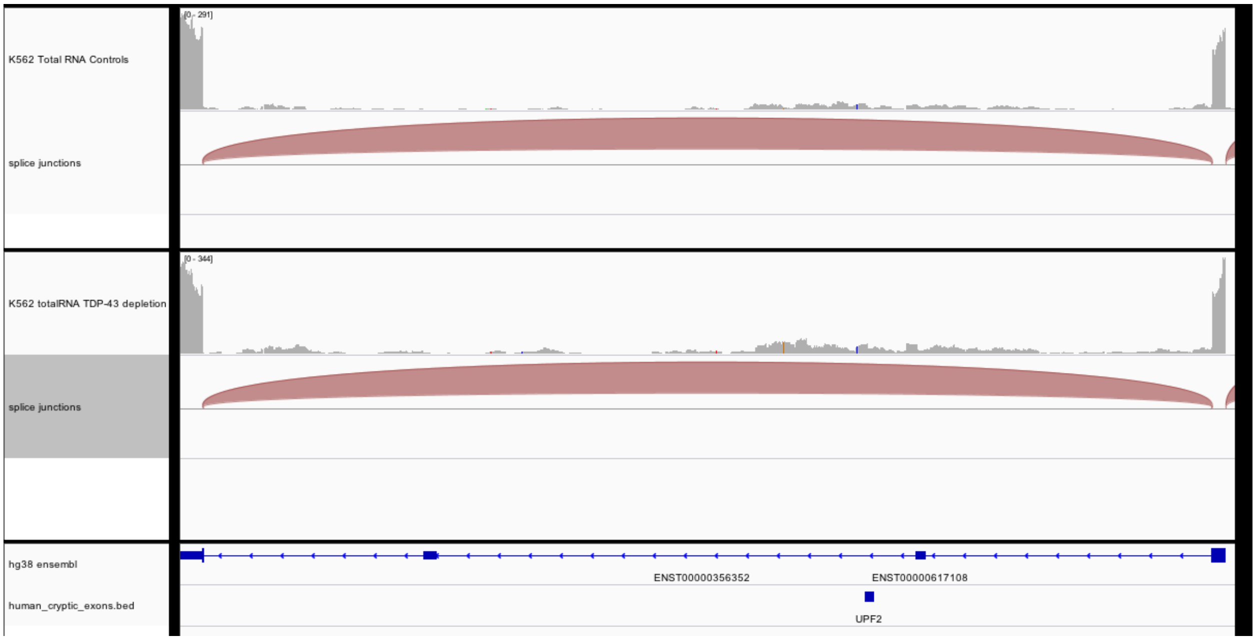

29 RRP1 E008i1

Human K562 mRNA

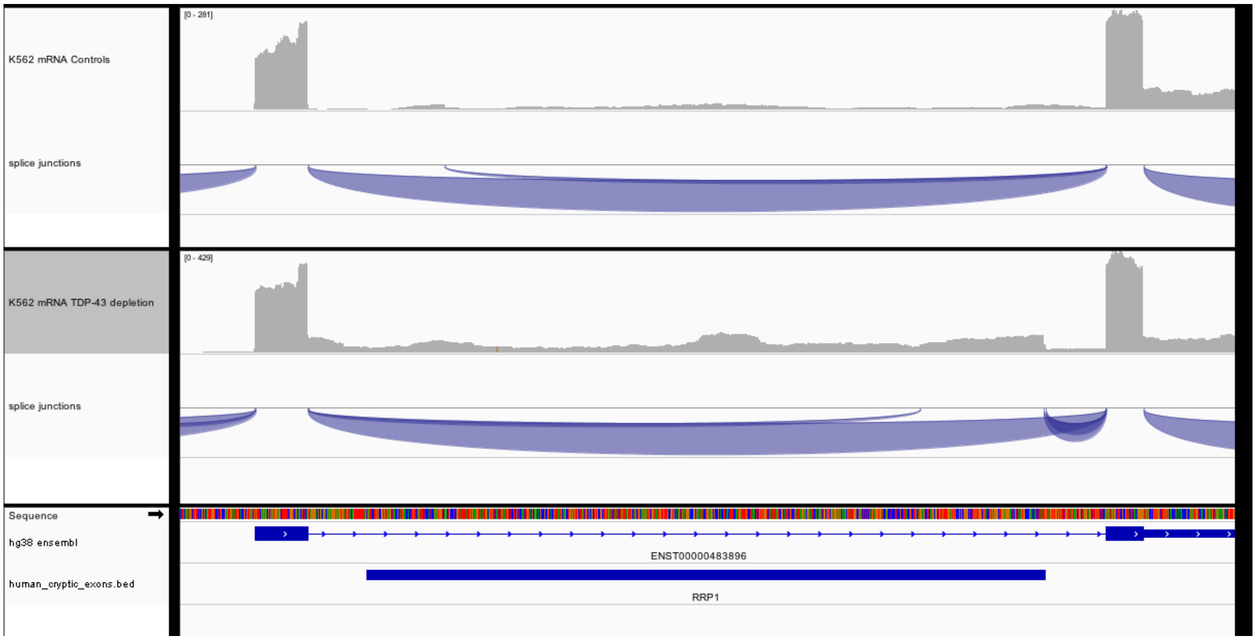

Human K562 total RNA

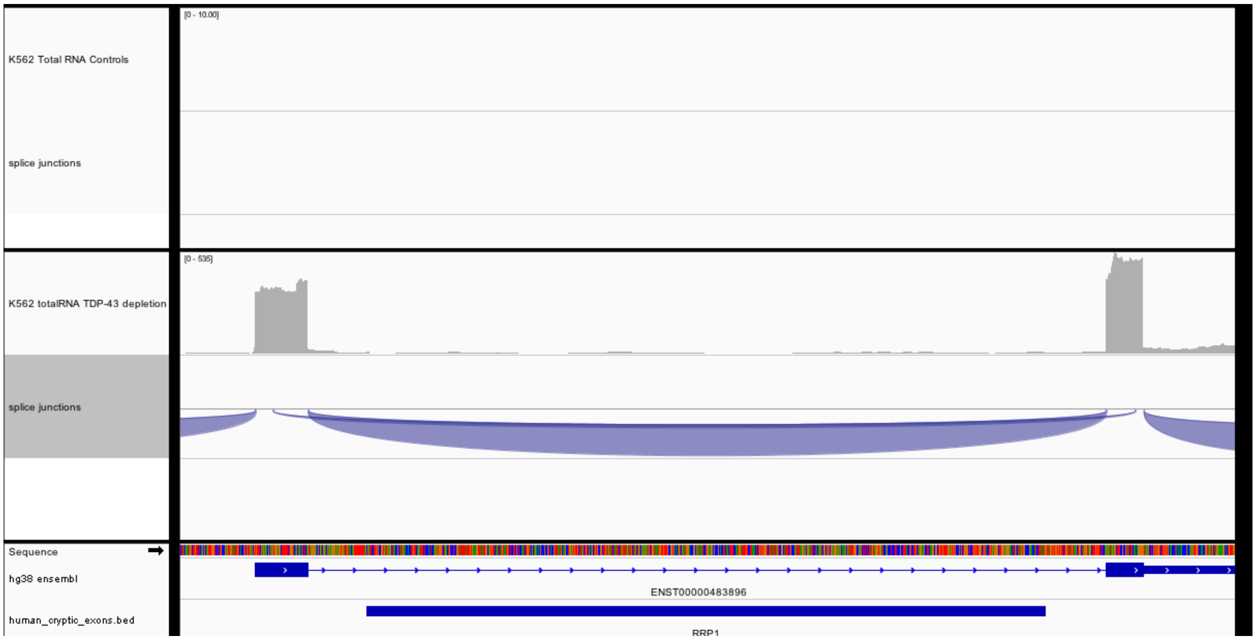

30 LRP8 E033i1

Human K562 mRNA

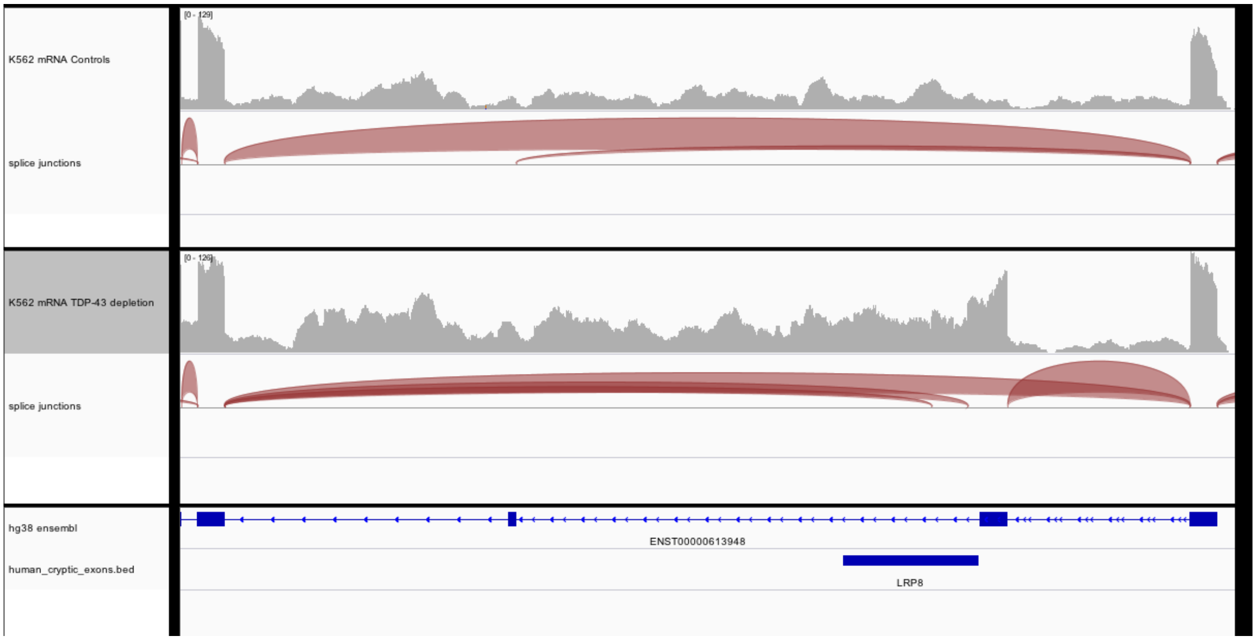

Human K562 total RNA

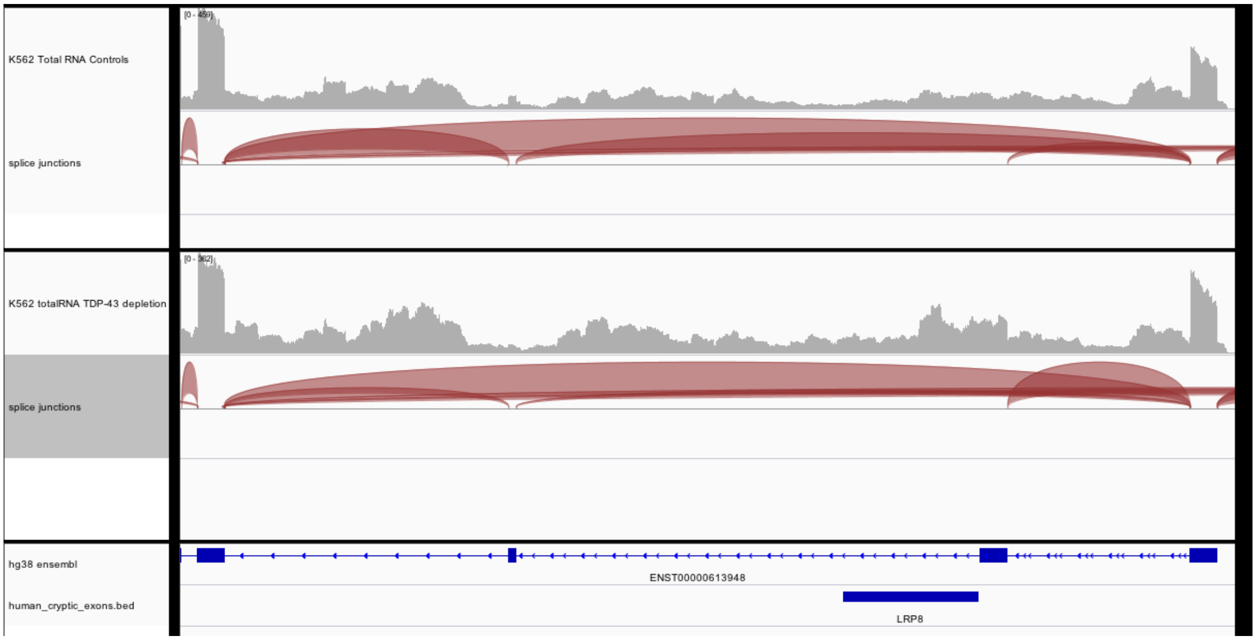

31 PKP4 E020i1

Human K562 mRNA

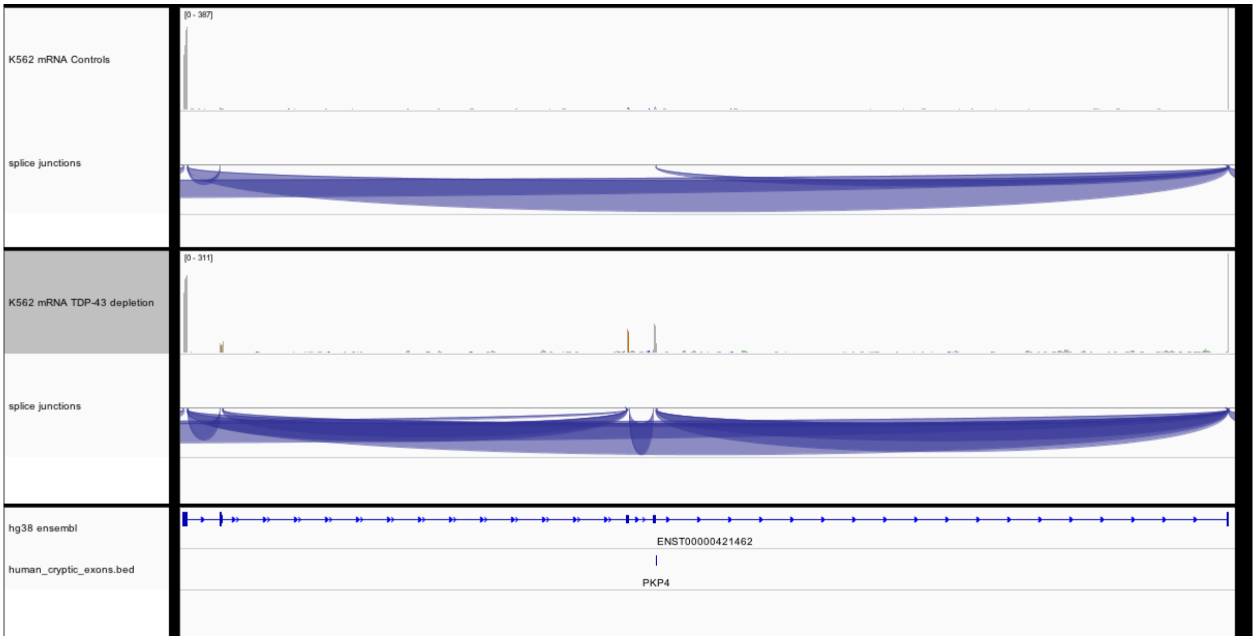

Human K562 total RNA

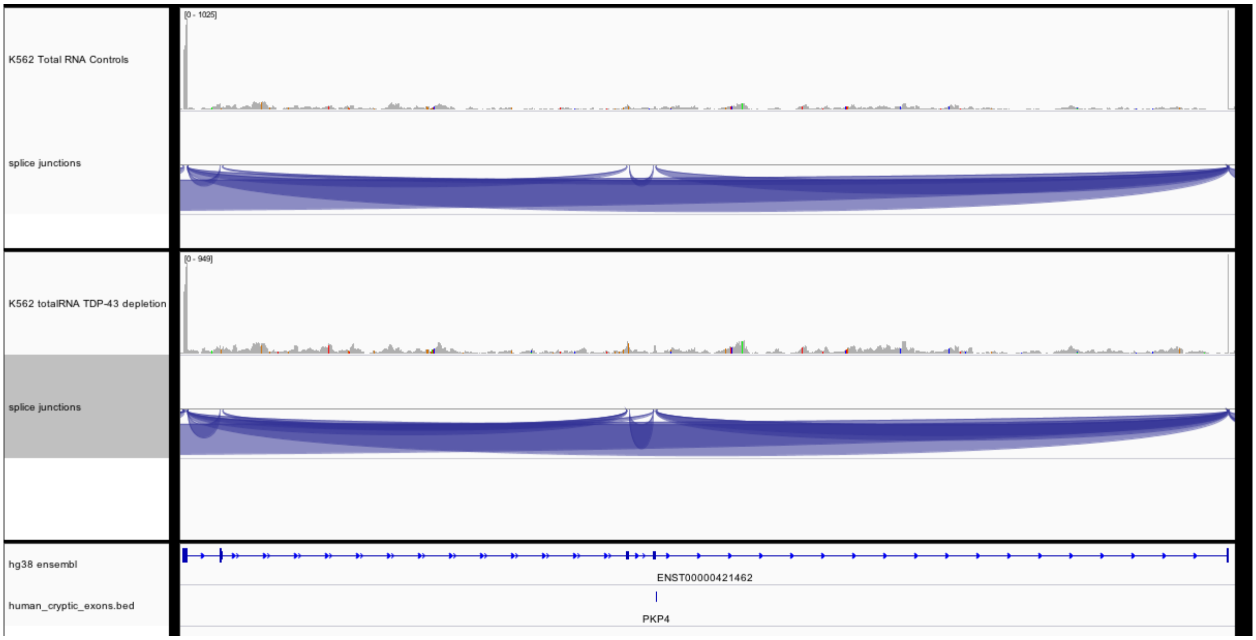

32 DDX52 E022i1

Human K562 mRNA

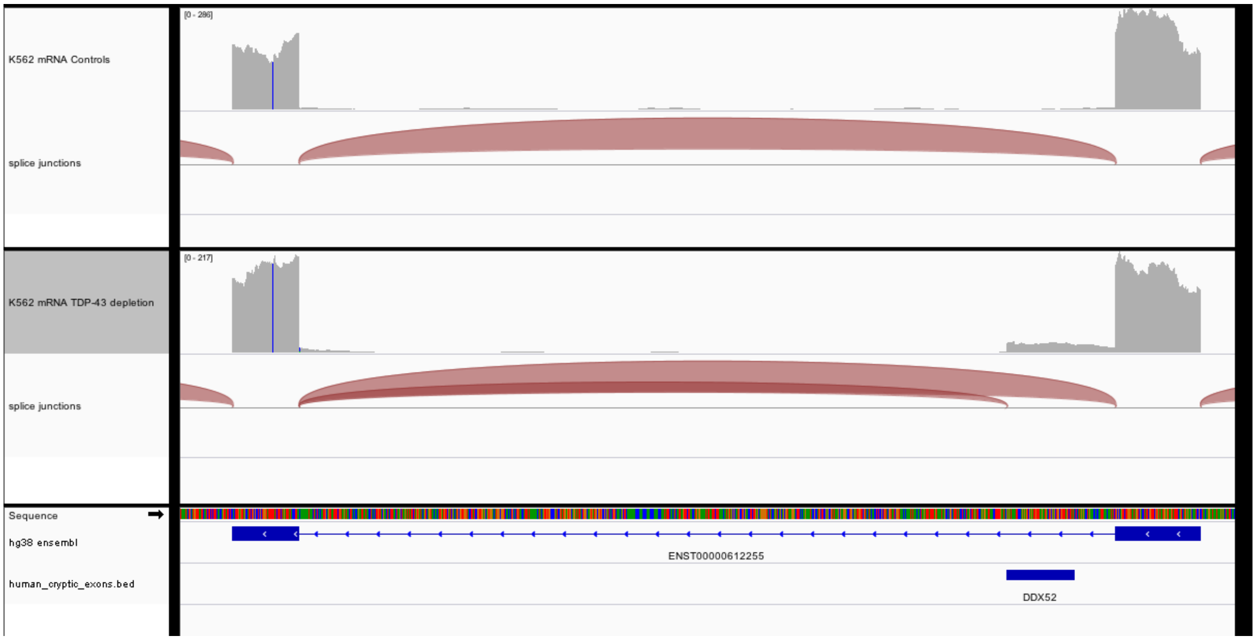

Human K562 total RNA

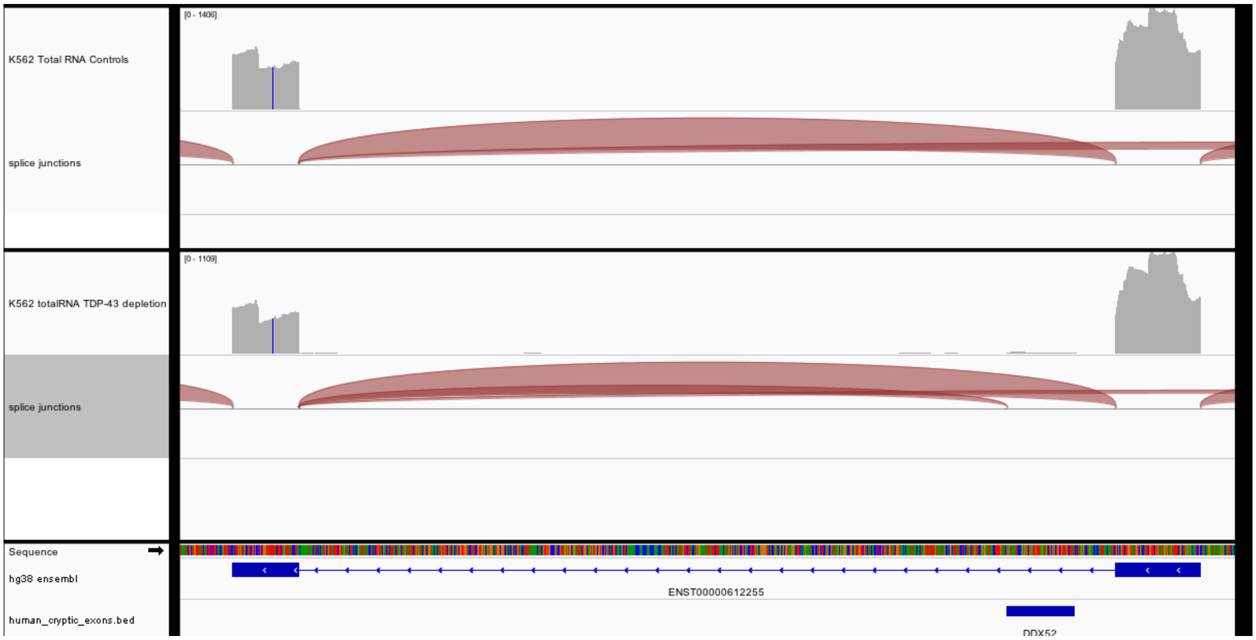

33 SETD5 E065i1

Human K562 mRNA

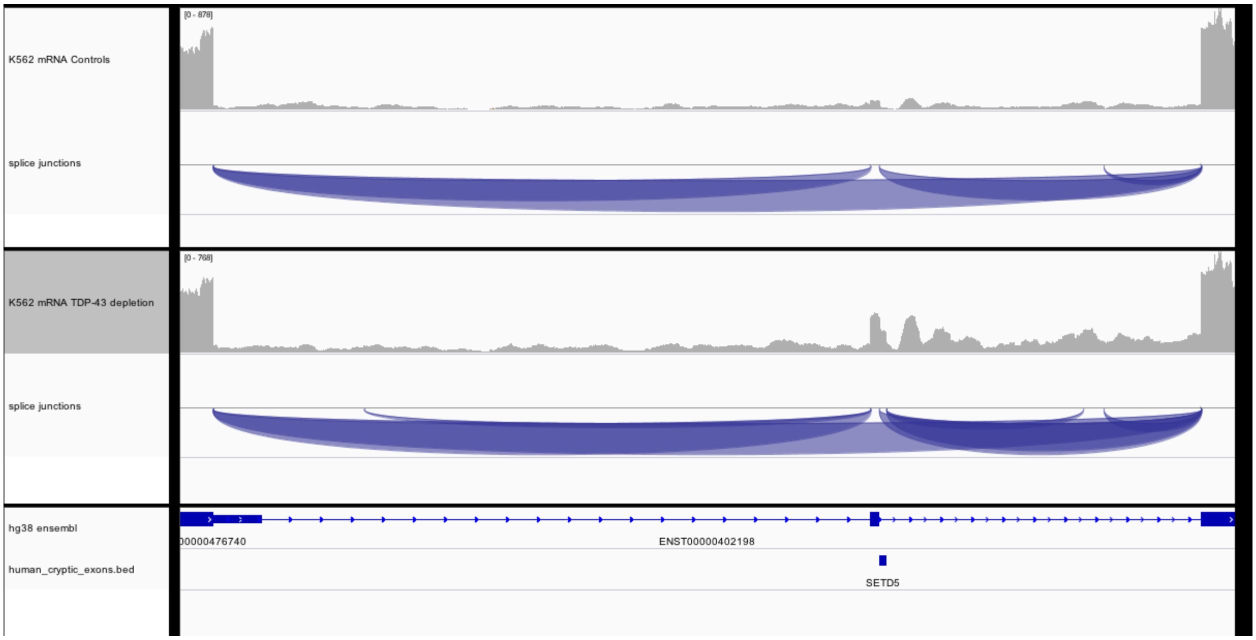

Human K562 total RNA

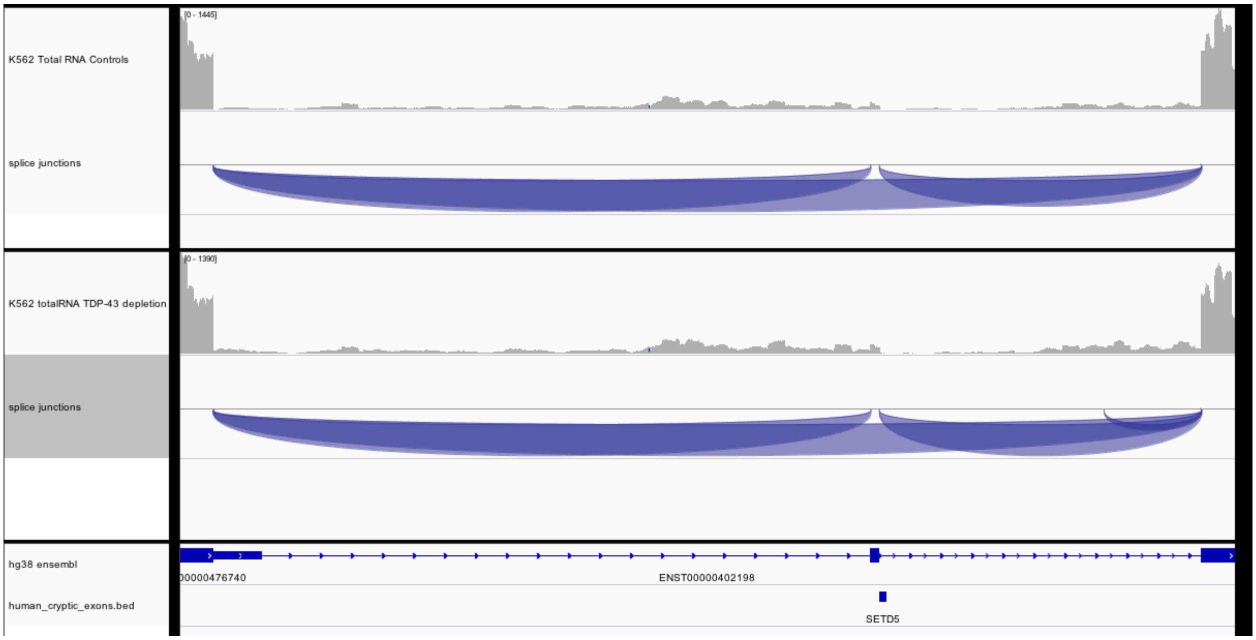

34 KDELC2 E018i2

Human K562 mRNA

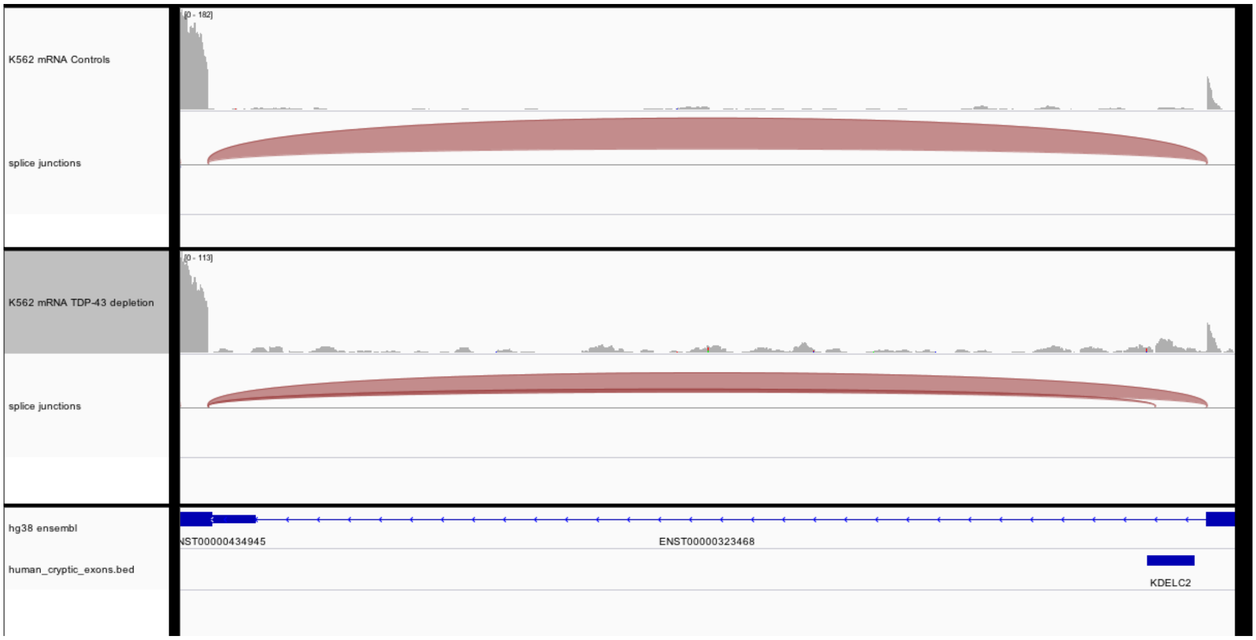

Human K562 total RNA

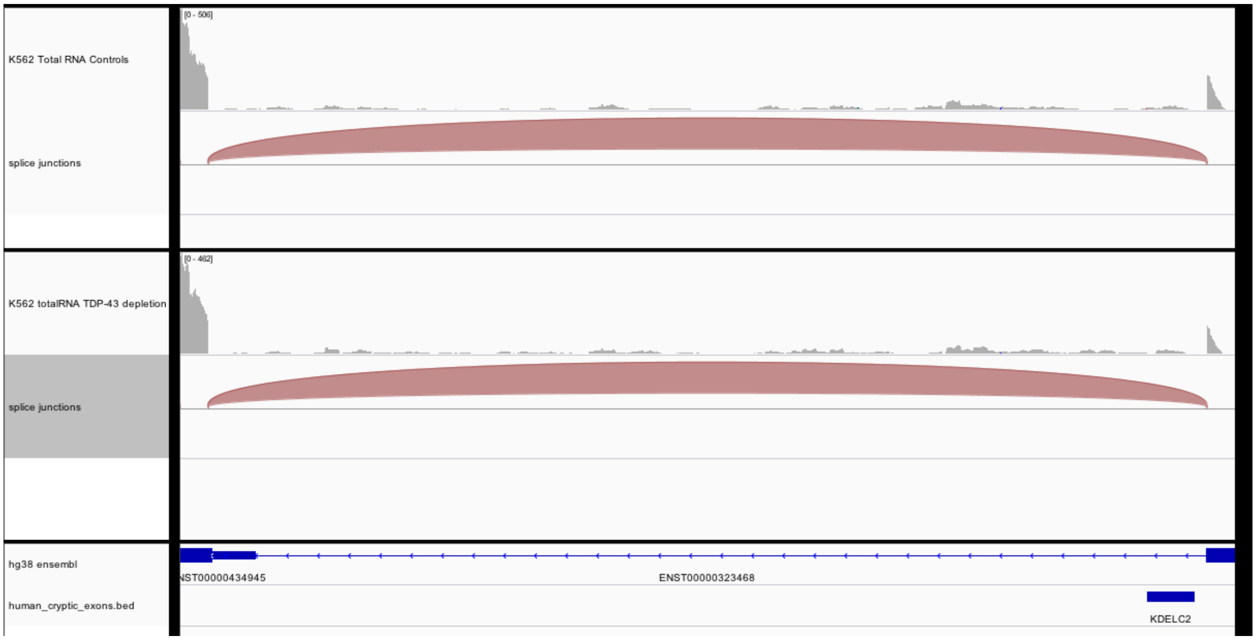

35 GPT2 E009i1

Human K562 mRNA

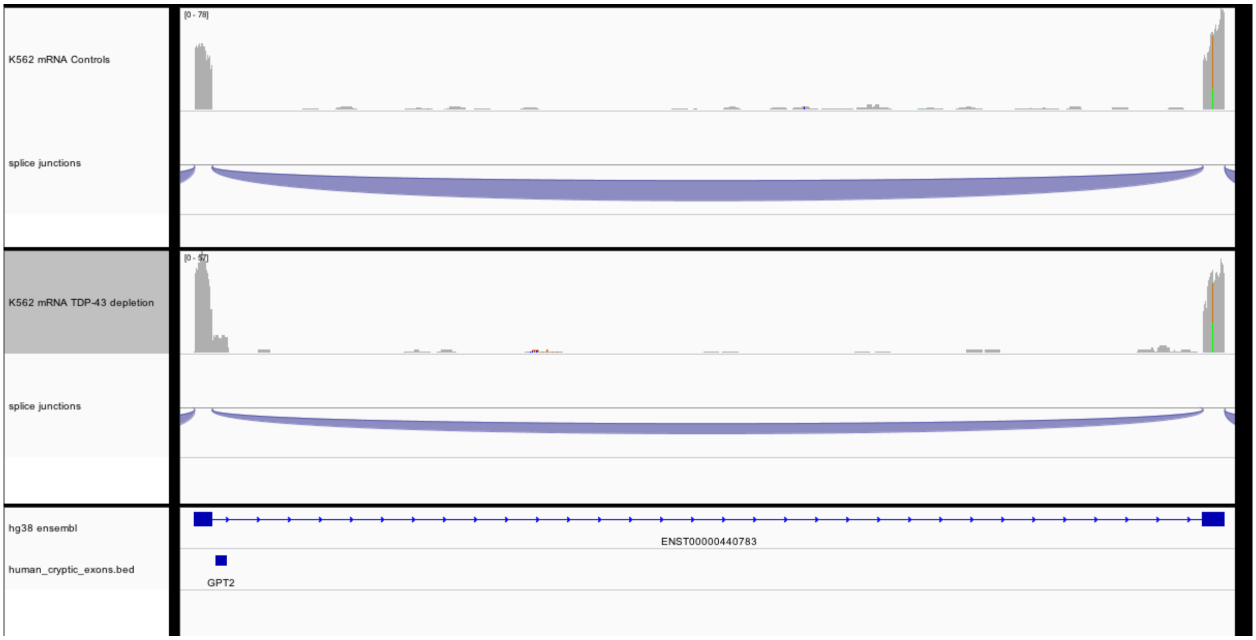

Human K562 total RNA

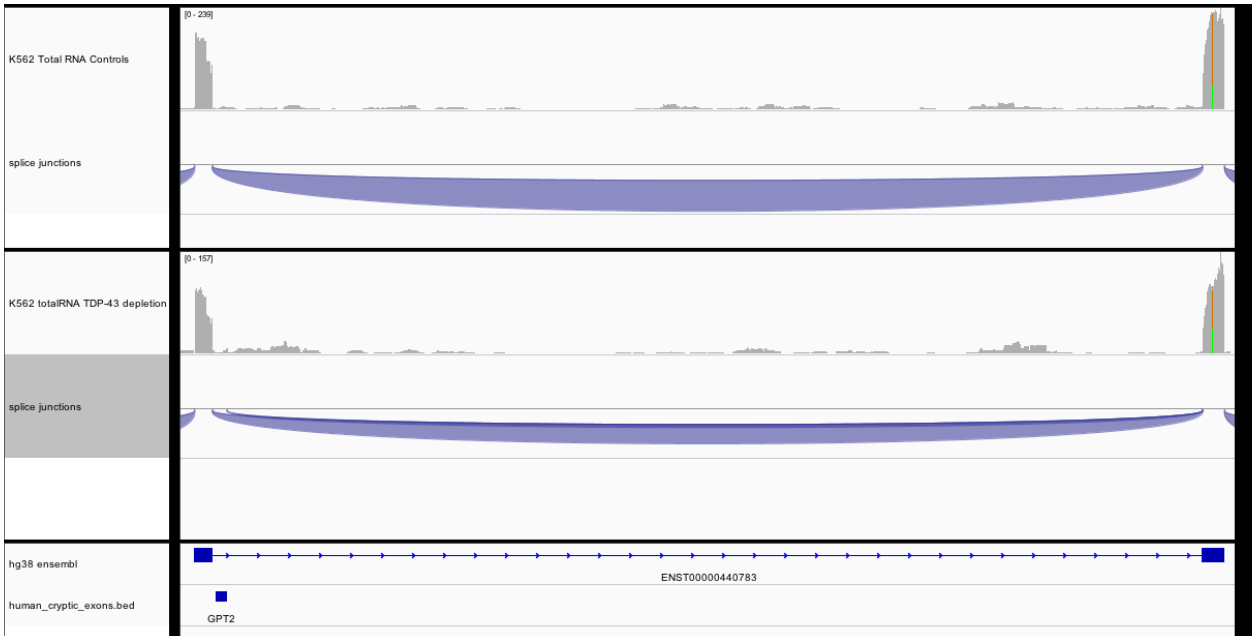

36 GSE1 E007i1

Human K562 mRNA

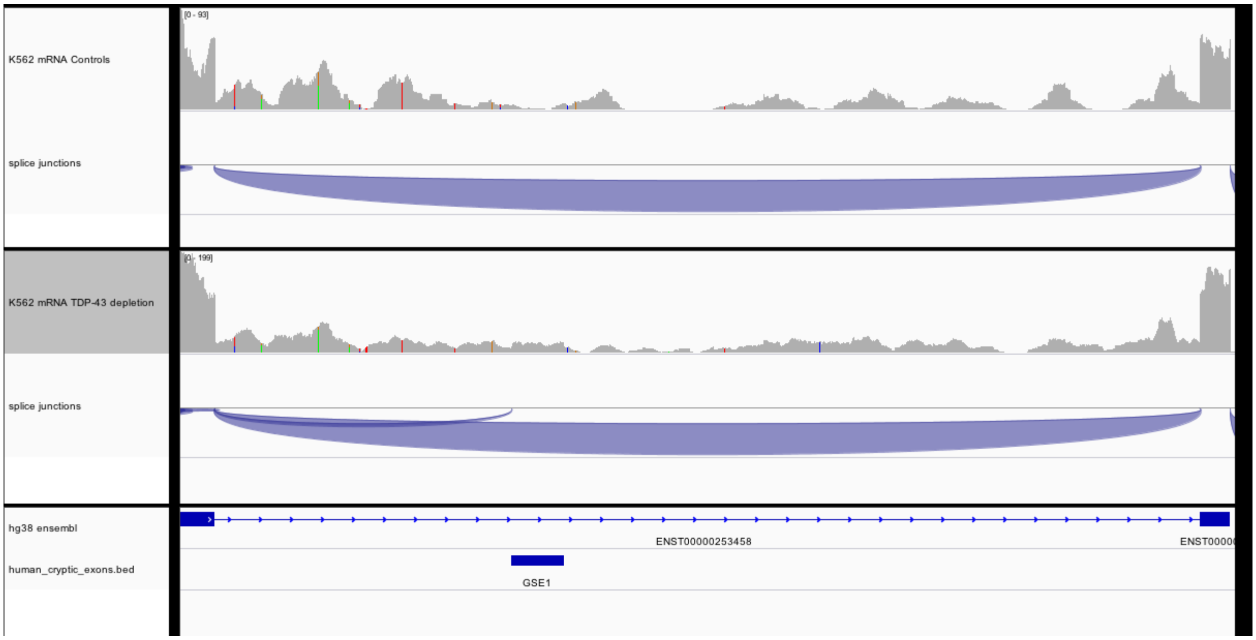

Human K562 total RNA

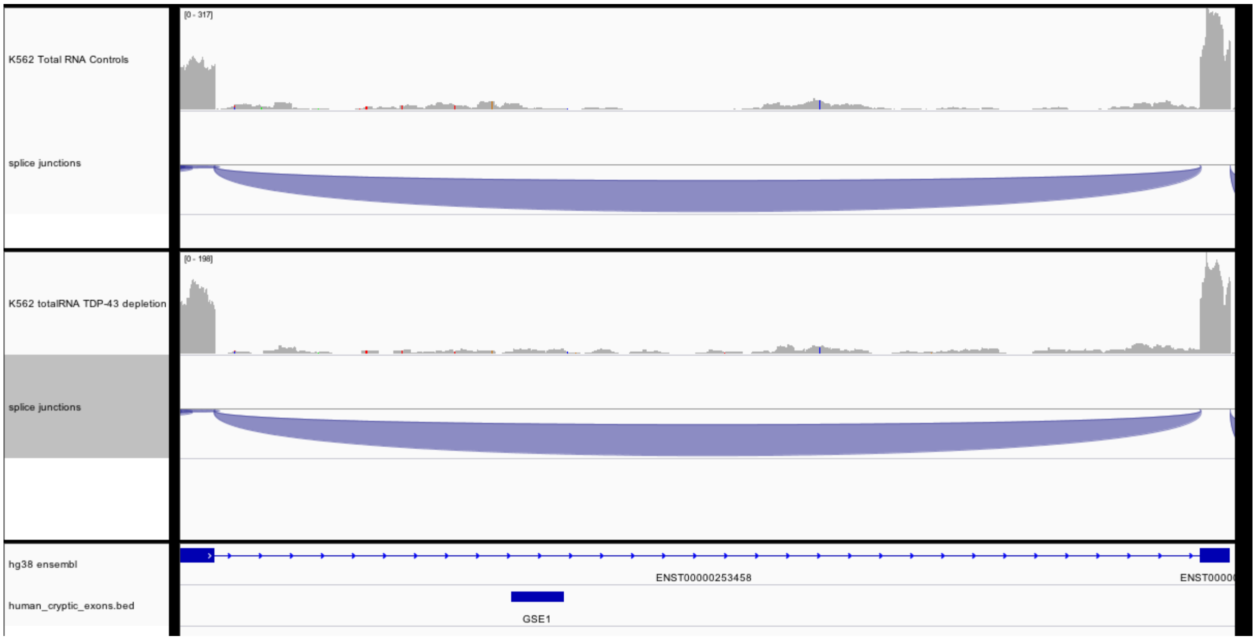

37 MBOAT2 E029i2

Human K562 mRNA

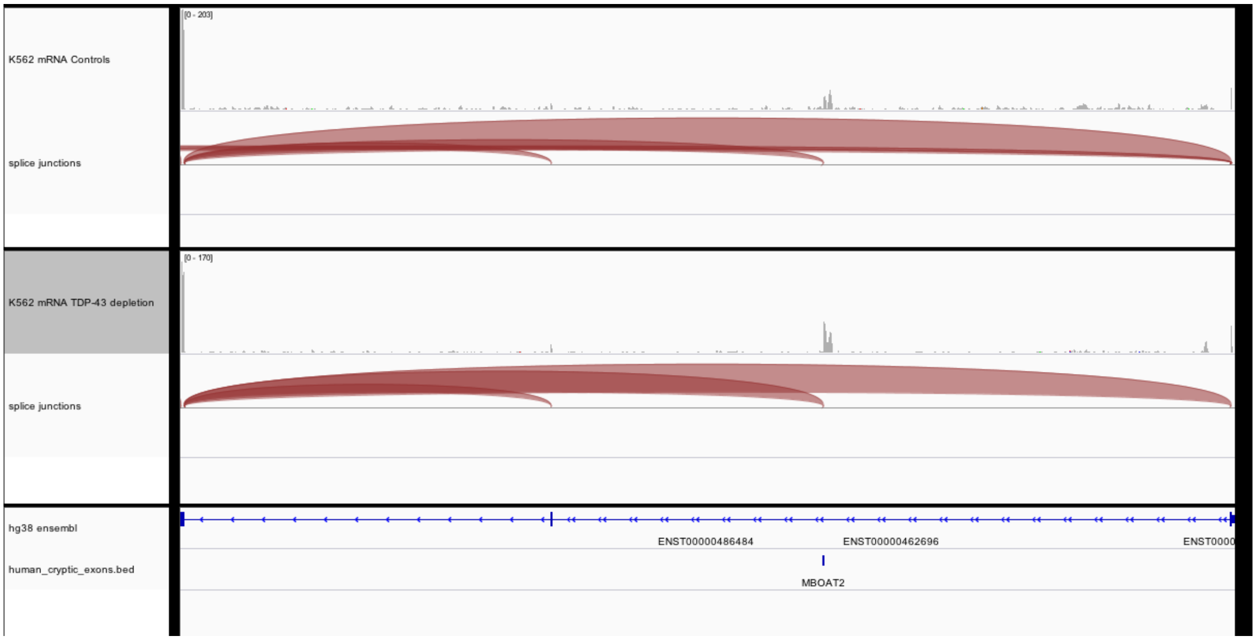

Human K562 total RNA

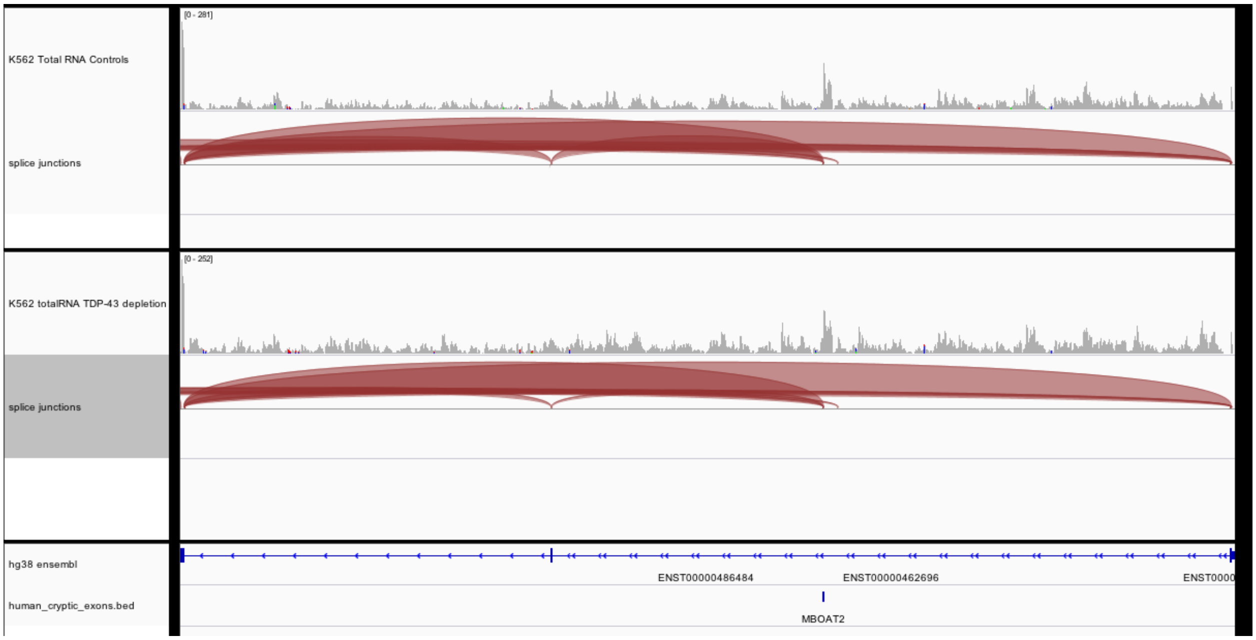

38 DNAAF5 E024i1

Human K562 mRNA

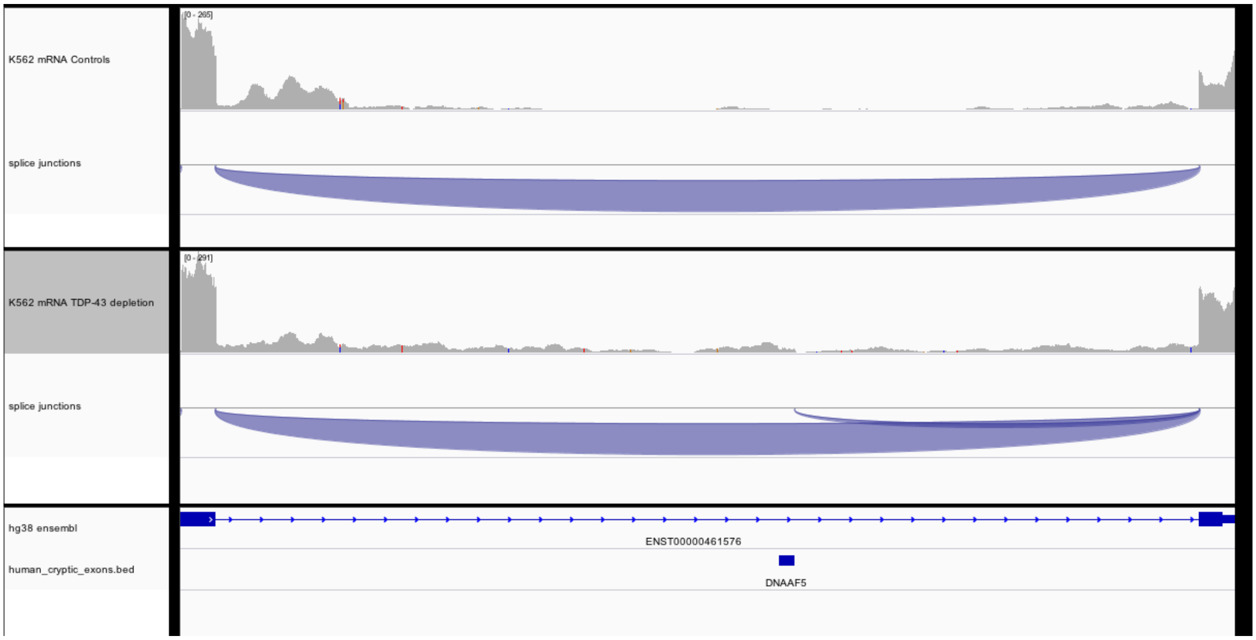

Human K562 total RNA

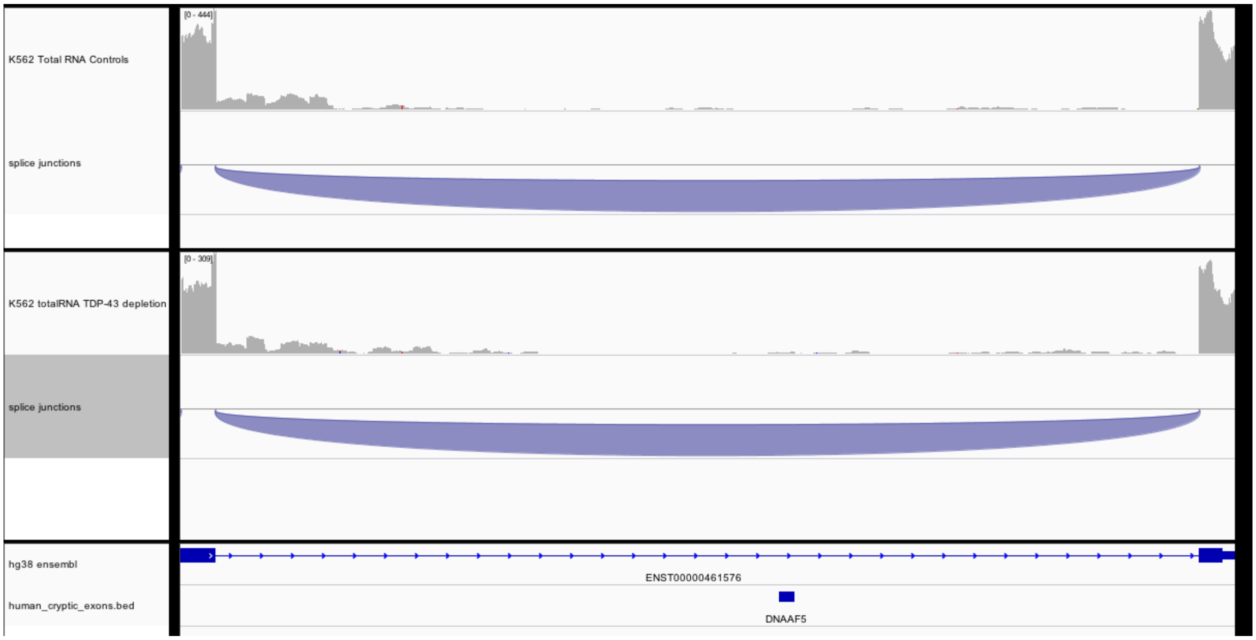

39 CLASP1 E012i1

Human K562 mRNA

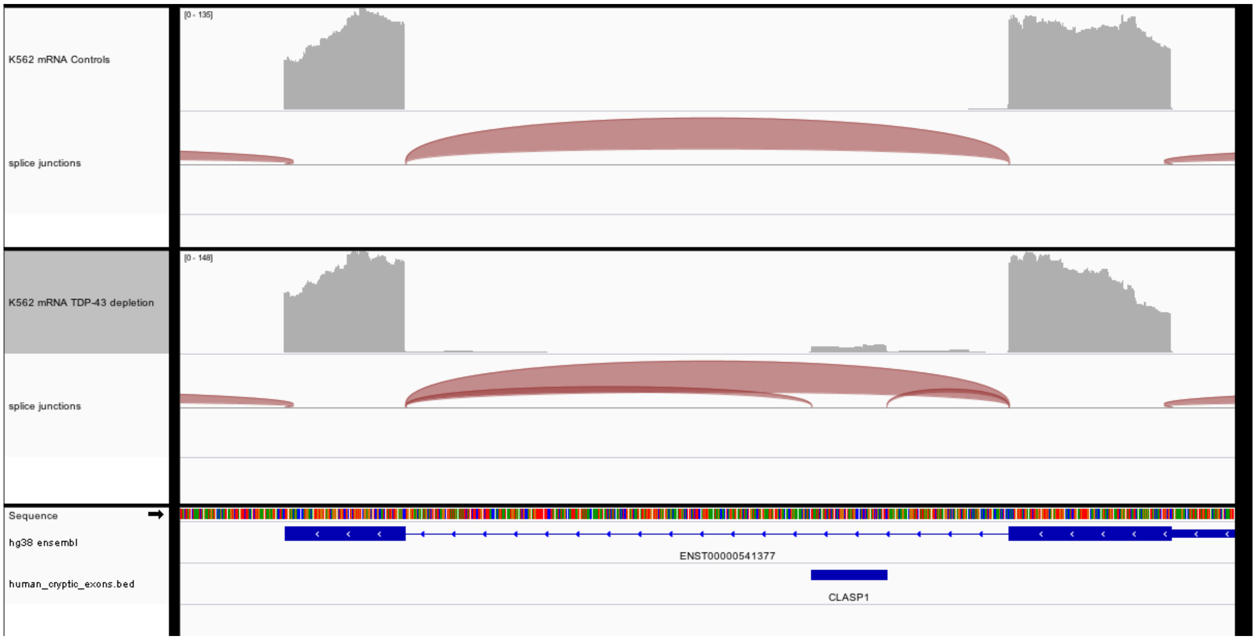

Human K562 total RNA

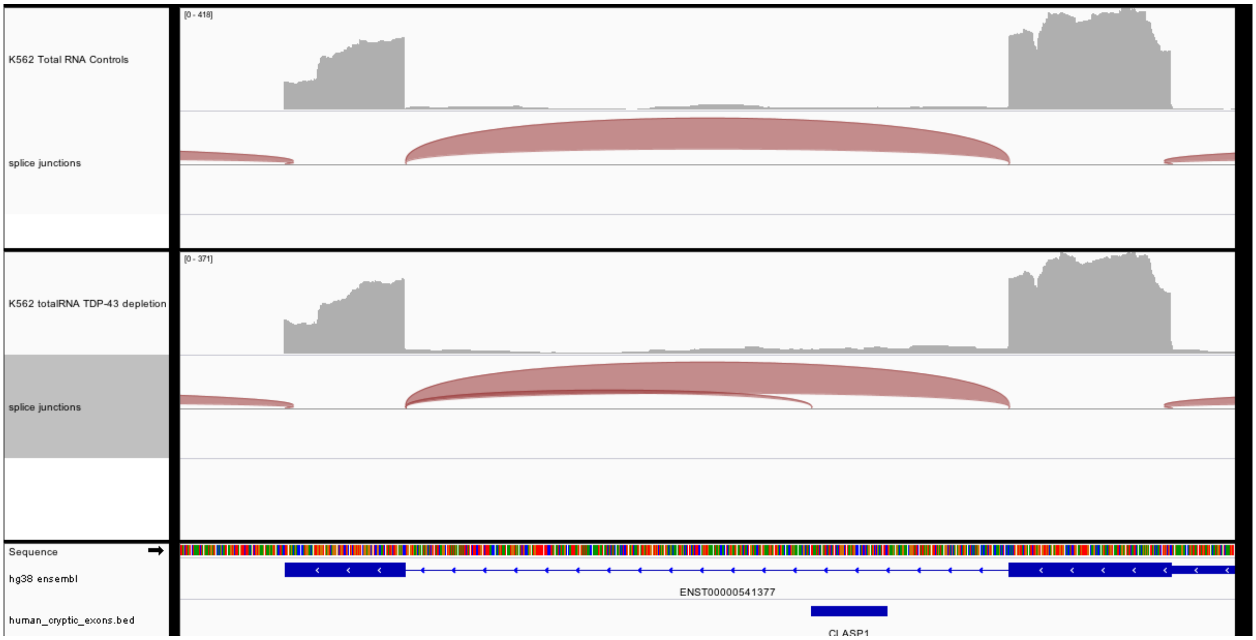

40 ZFP91+ZFP91-CNTF E012i1

Human K562 mRNA

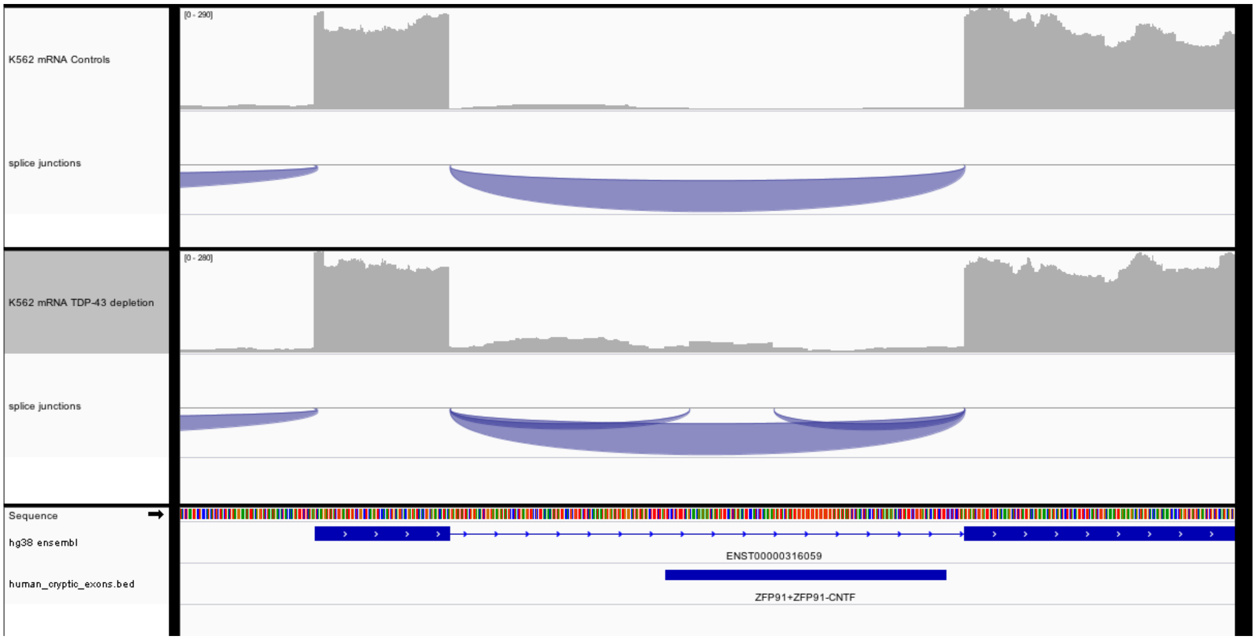

Human K562 total RNA

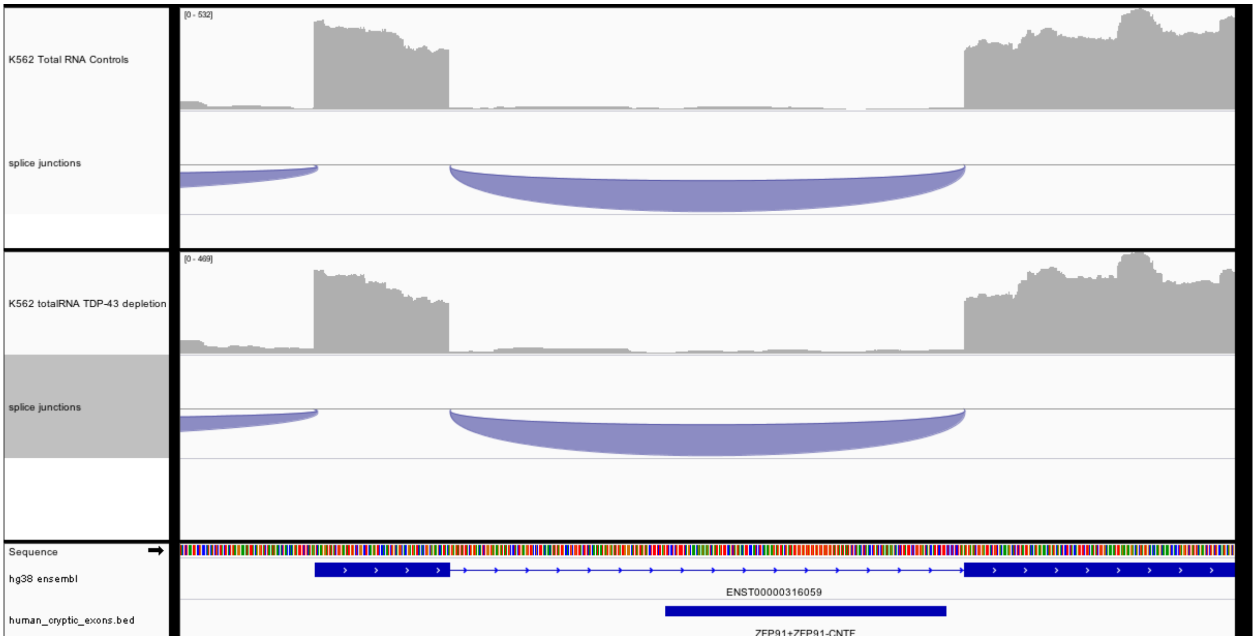

41 FAM178B E017i1

Human K562 mRNA

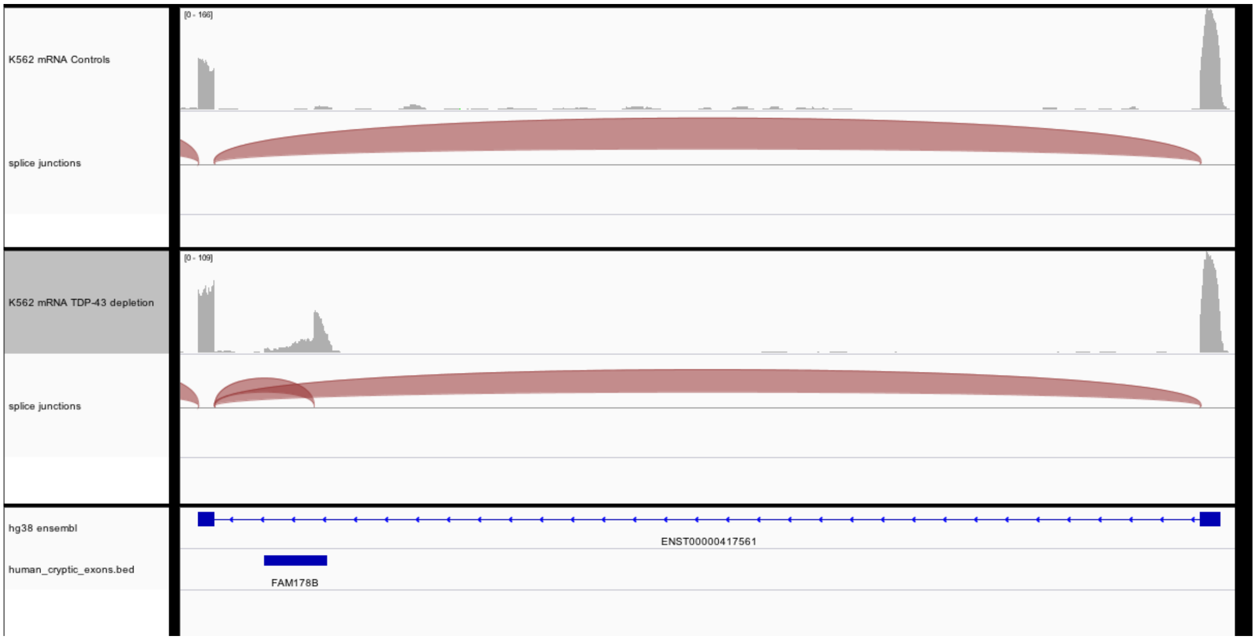

Human K562 total RNA

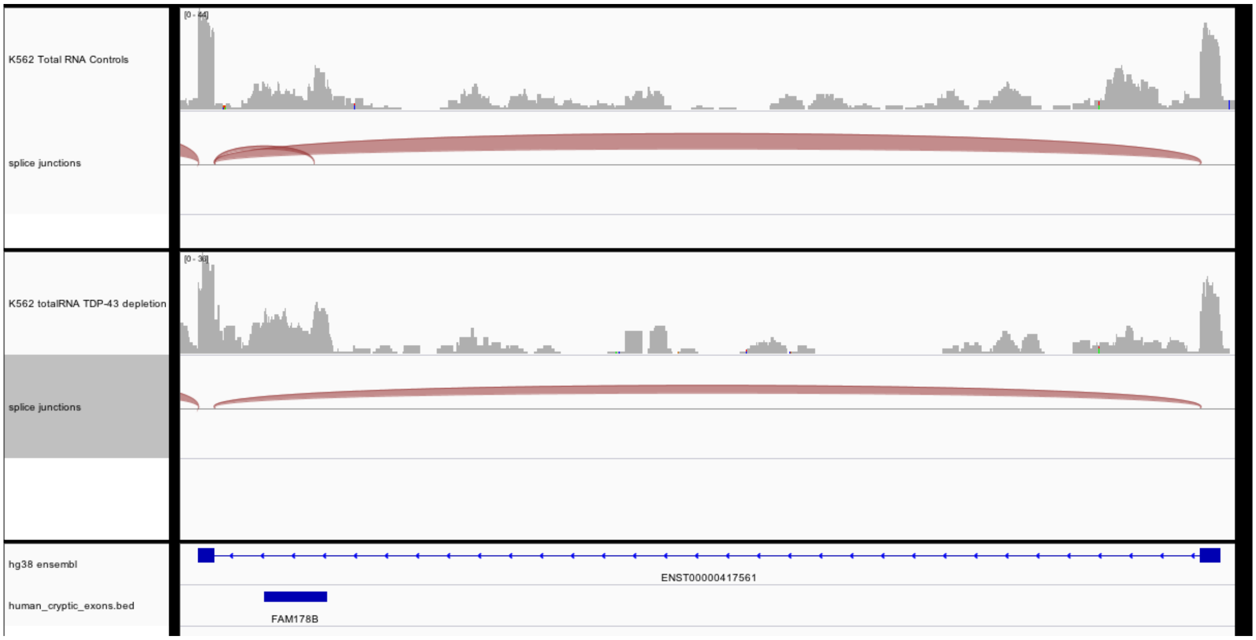

42 ANAPC1 E020i1

Human K562 mRNA

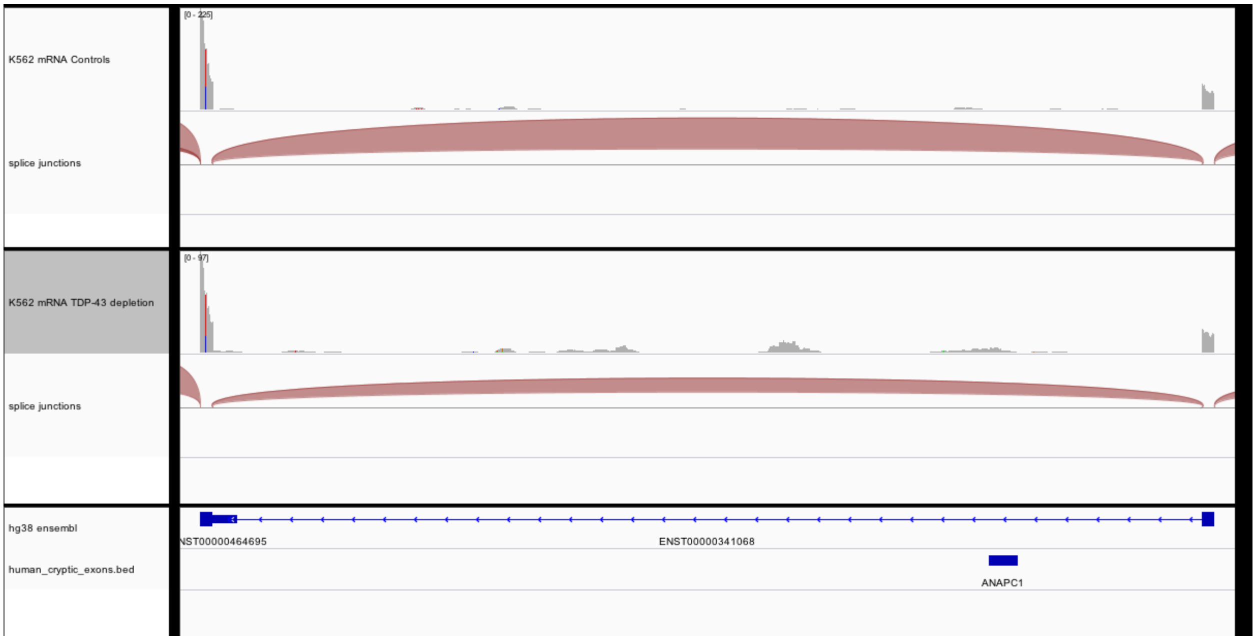

Human K562 total RNA

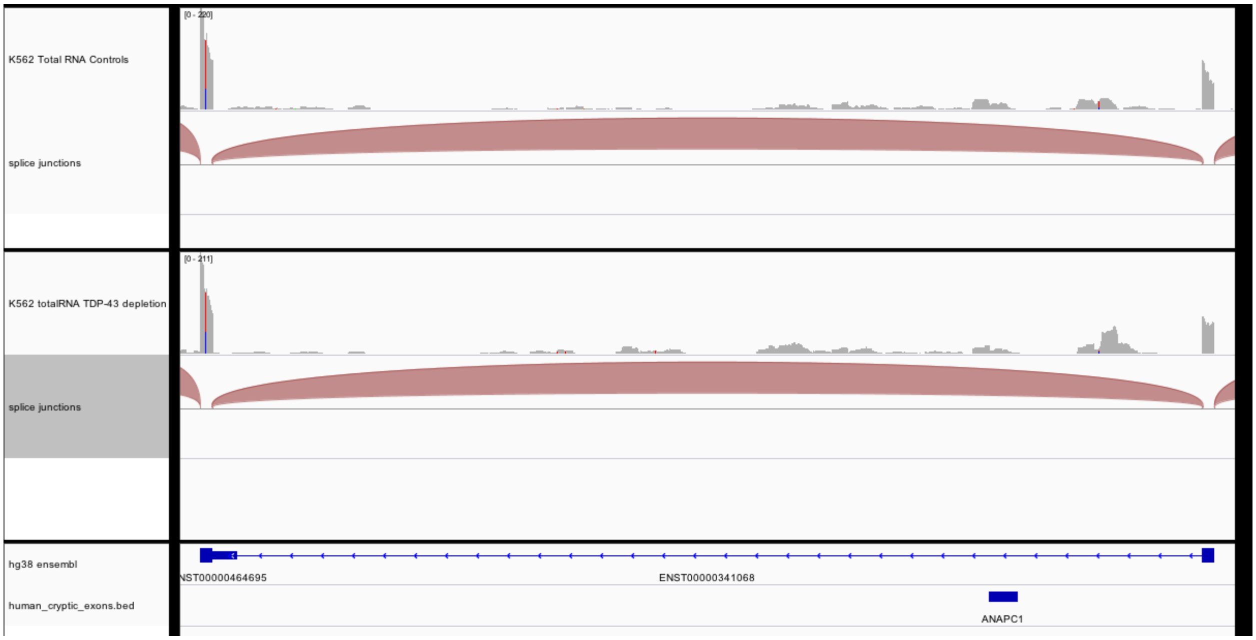

43 CERK E005i1

Human K562 mRNA

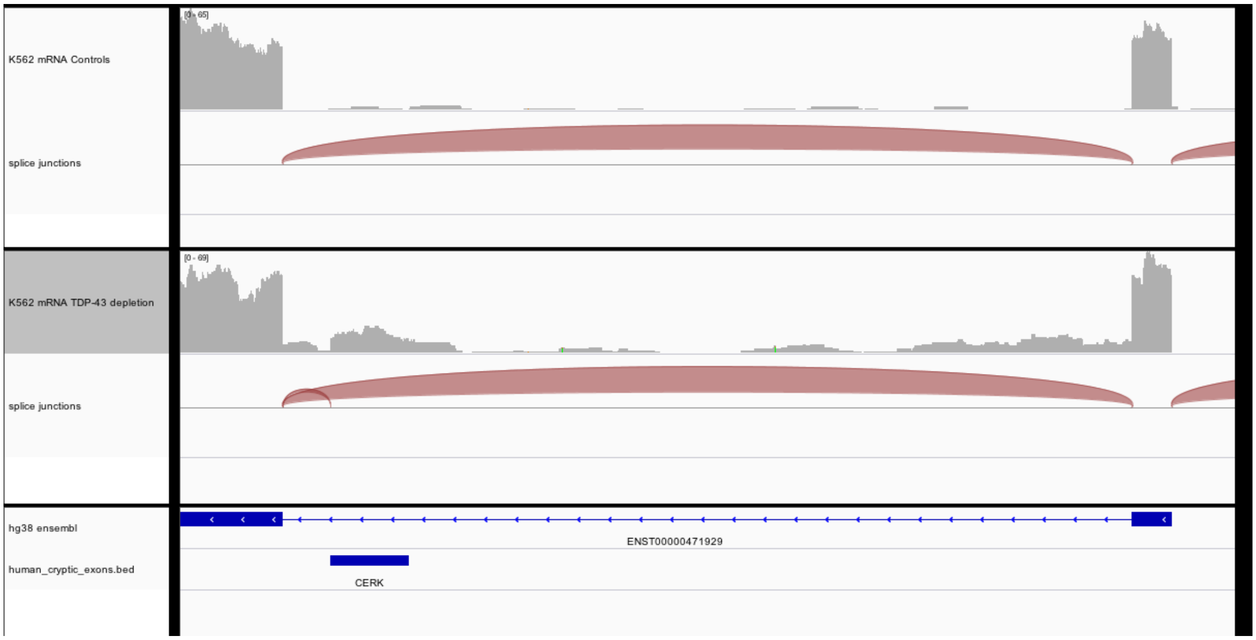

Human K562 total RNA

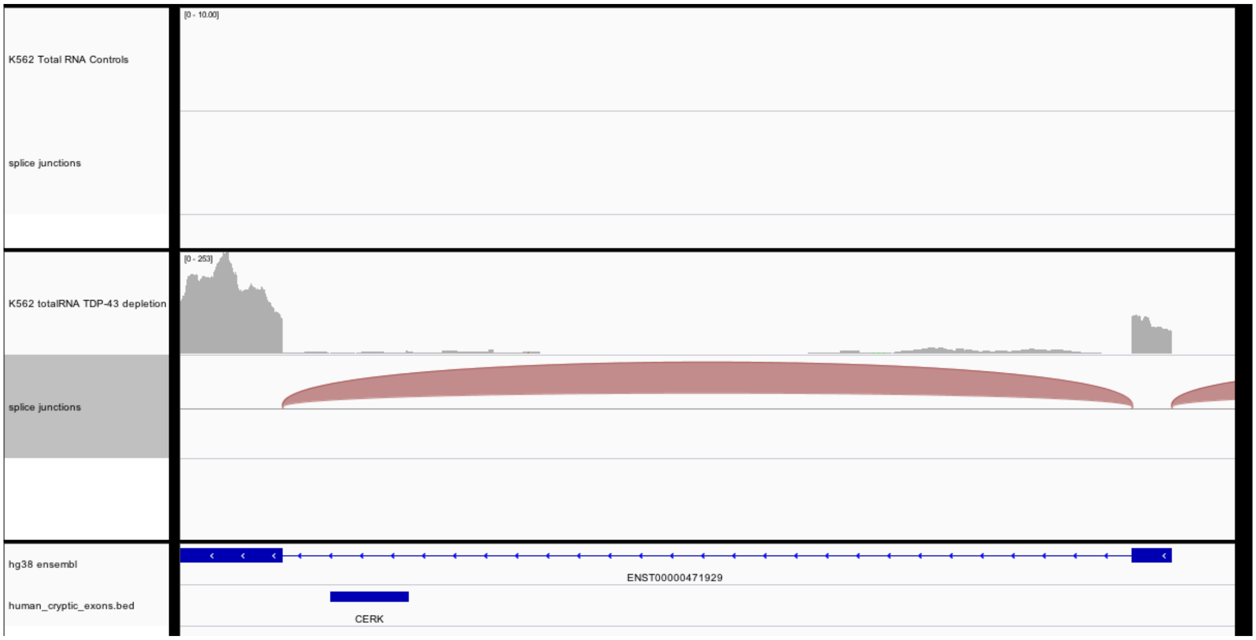

44 ARHGAP11B E033i1

Human K562 mRNA

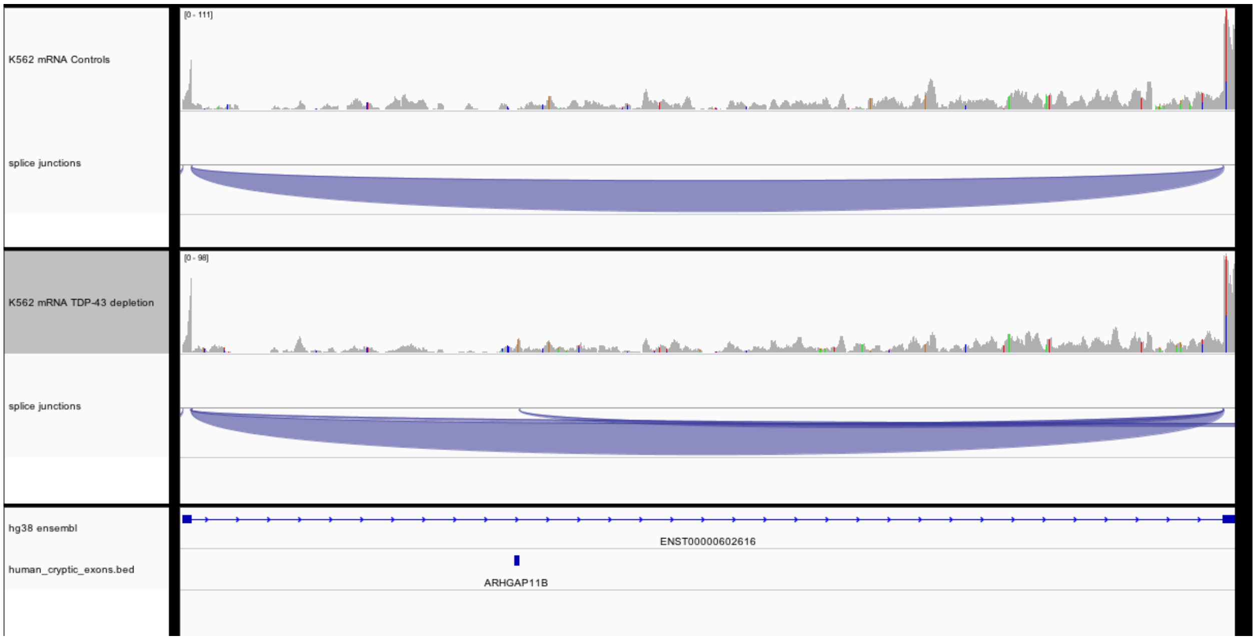

Human K562 total RNA

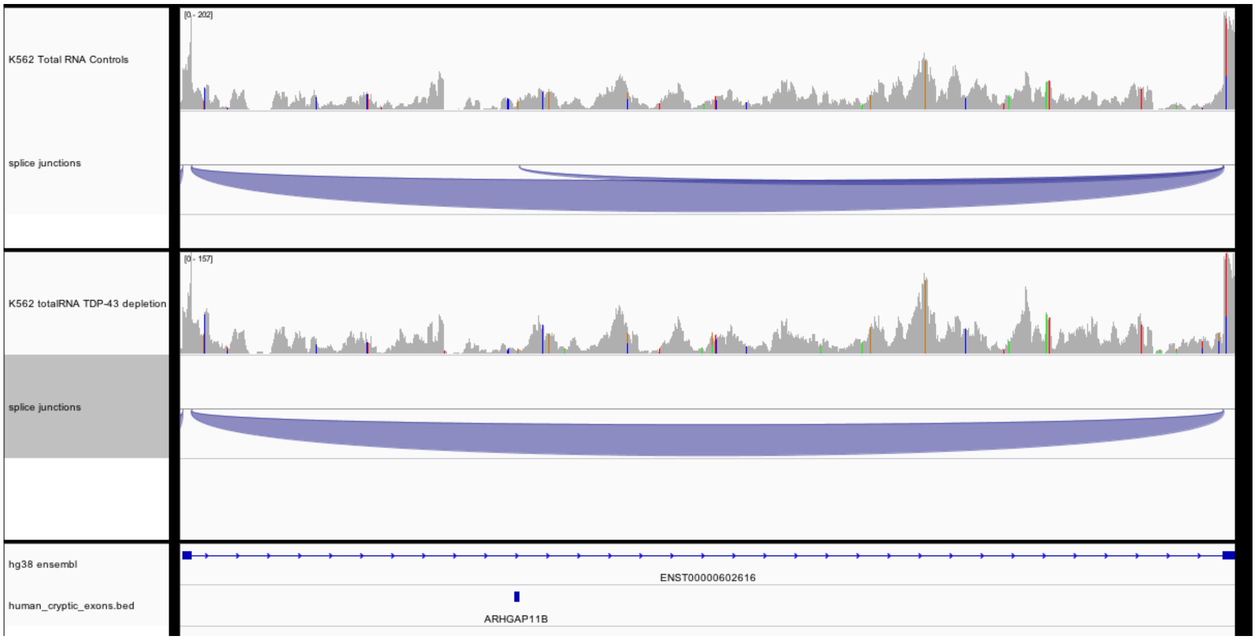

45 GOLGA8A E037i1

Human K562 mRNA

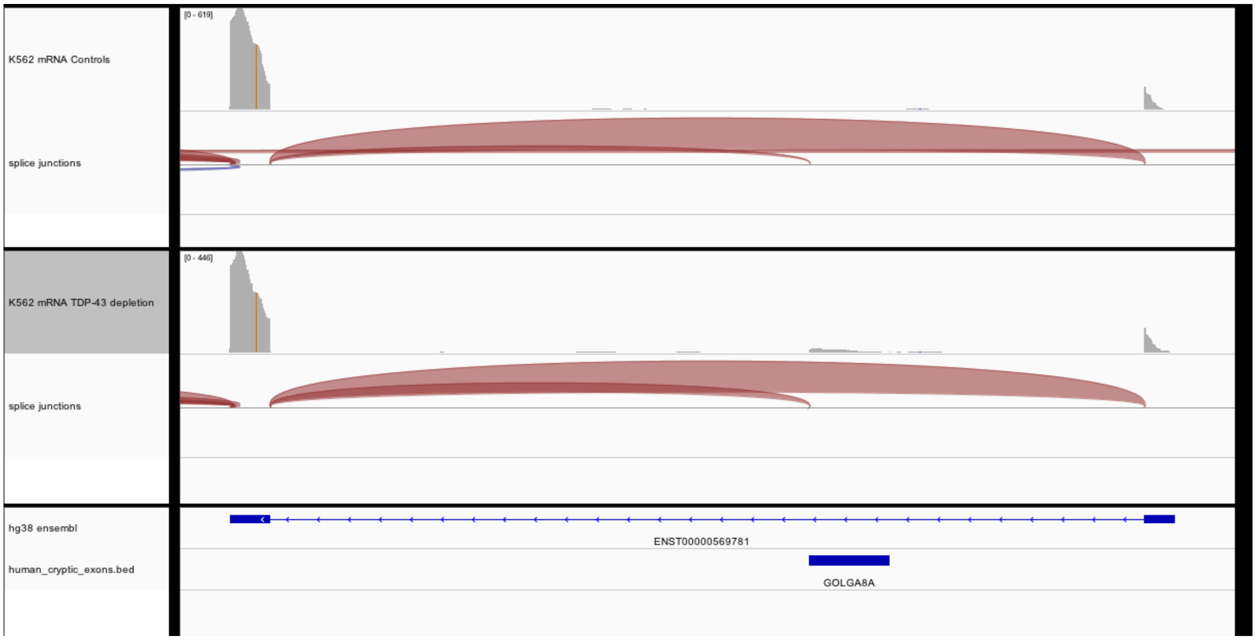

Human K562 total RNA

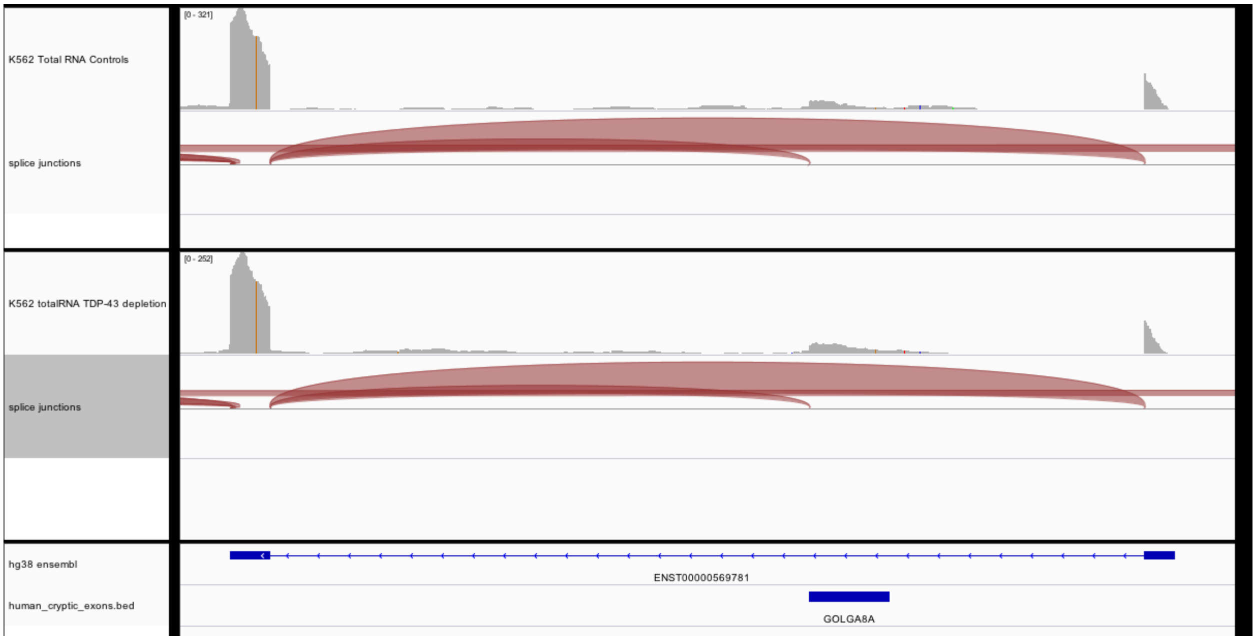

46 TGFBRAP1 E014i2

Human K562 mRNA

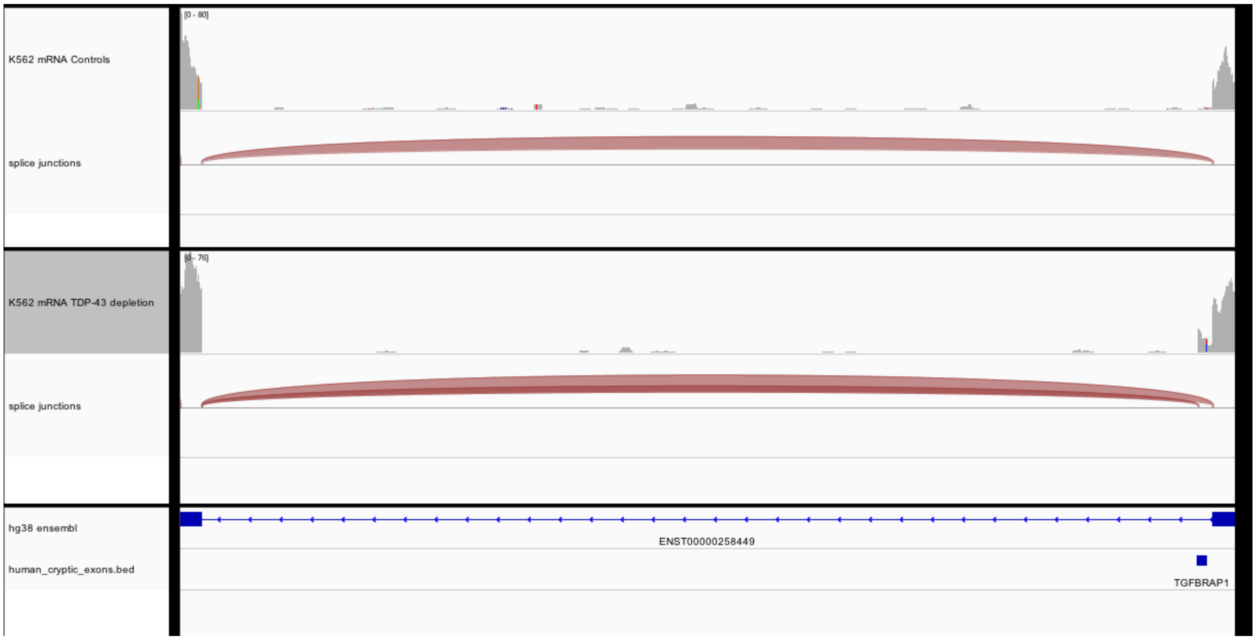

Human K562 total RNA

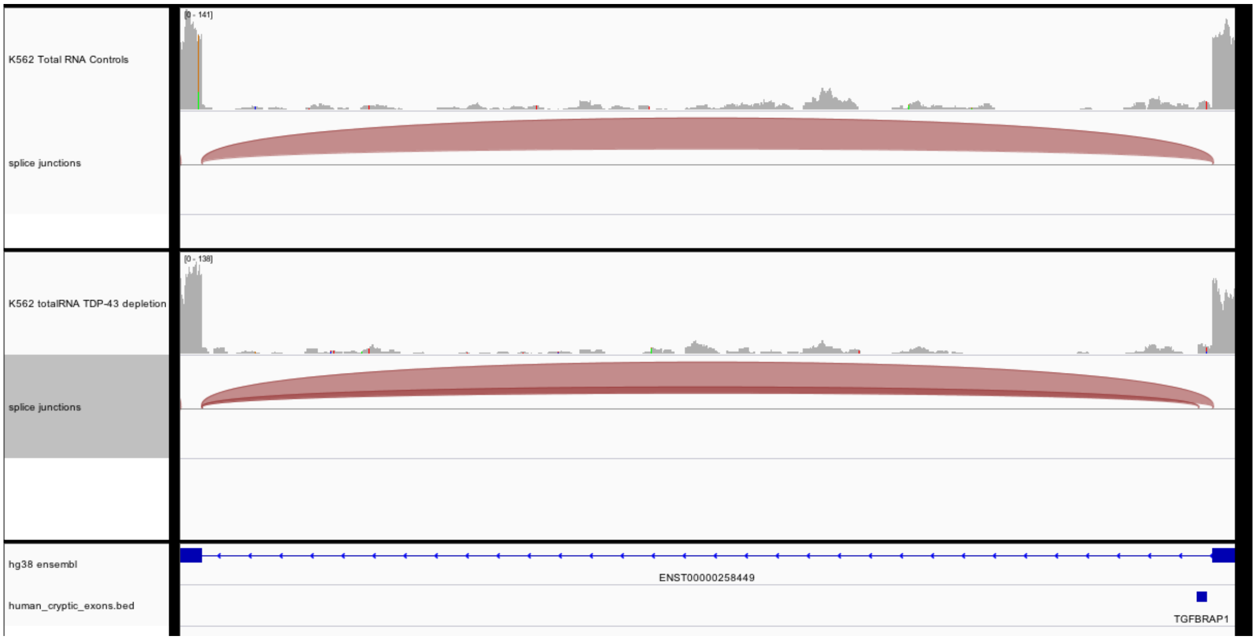

47 CAMK2G E039i1

Human K562 mRNA

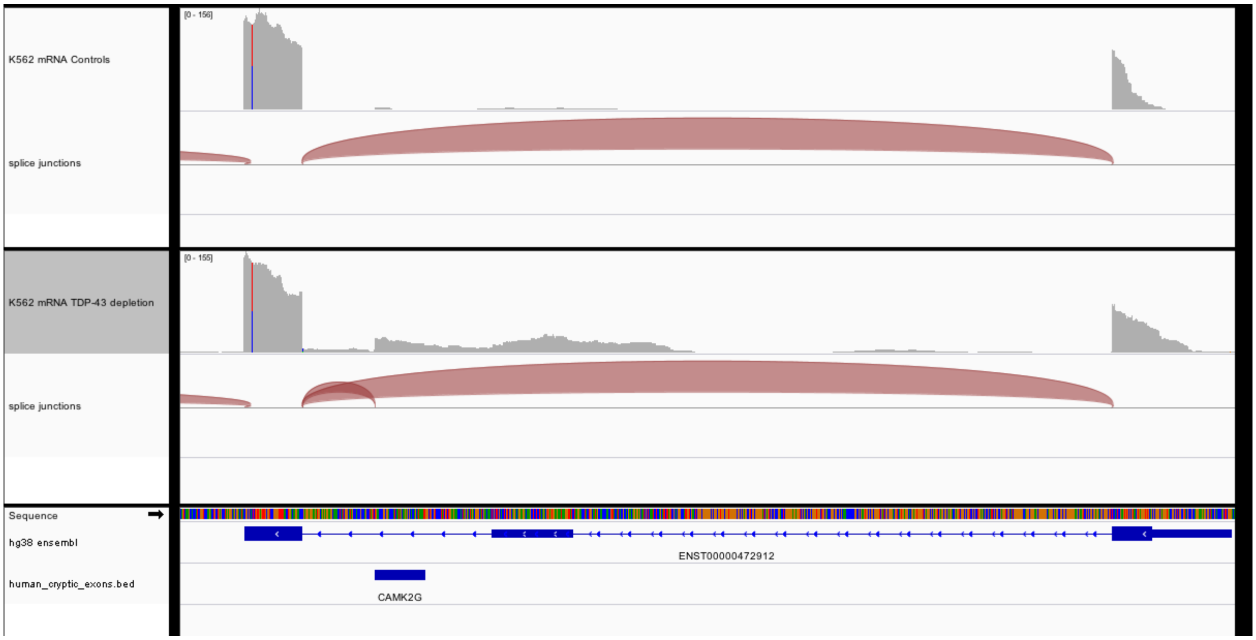

Human K562 total RNA

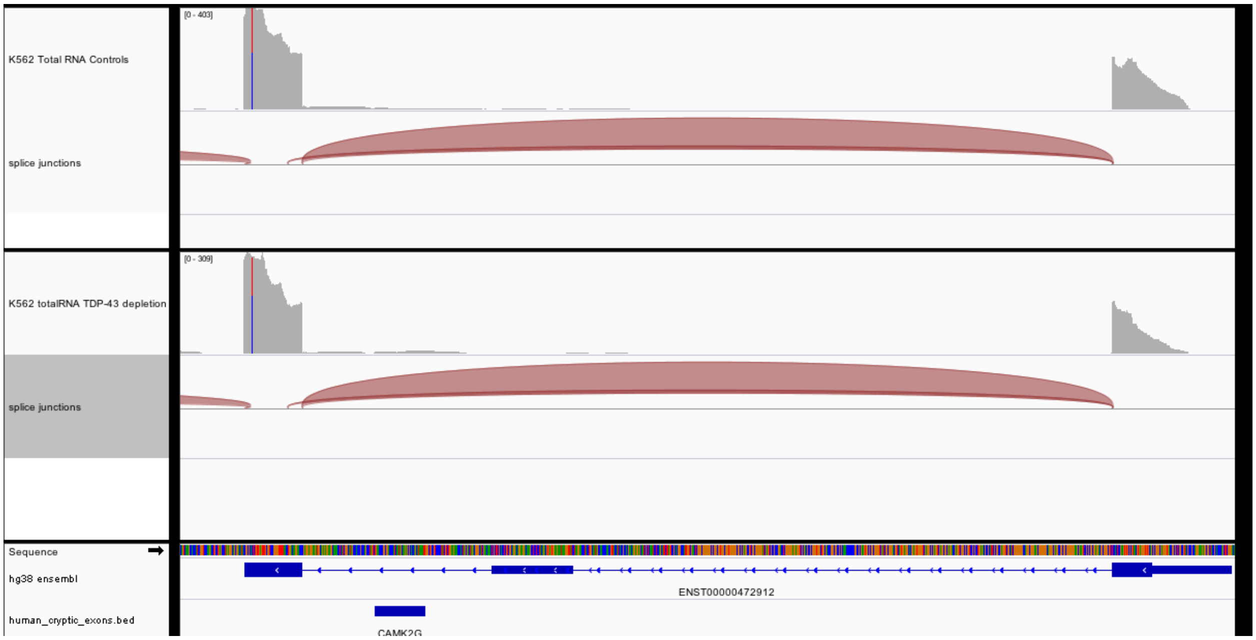

48 DCAF17 E008i1

Human K562 mRNA

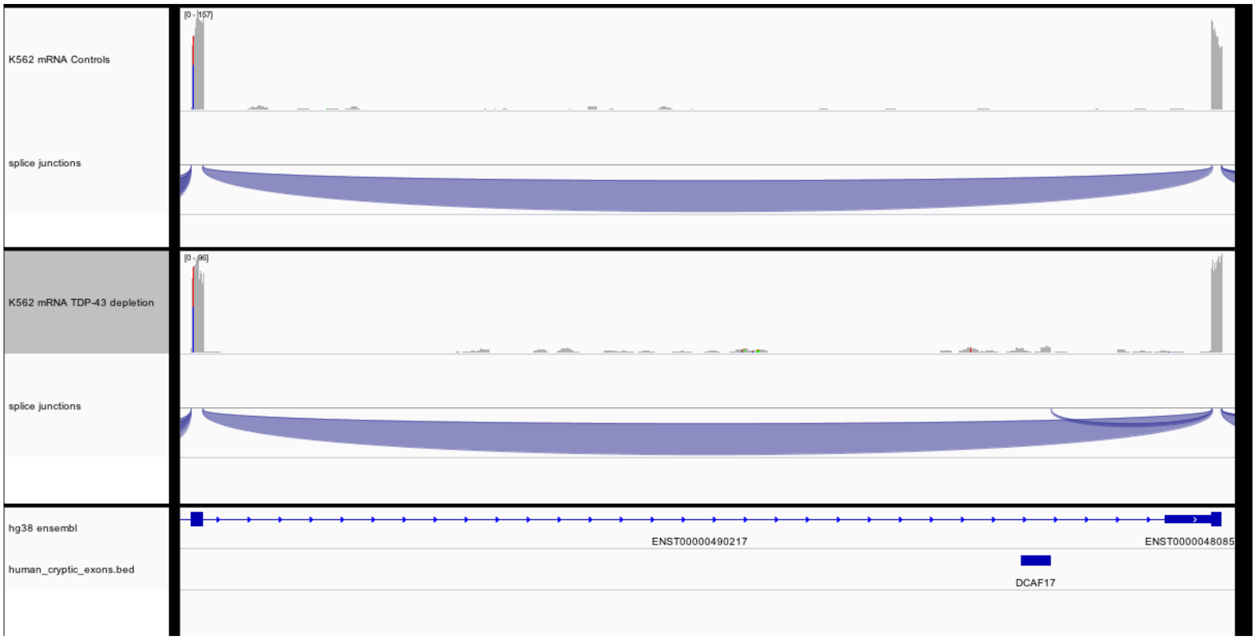

Human K562 total RNA

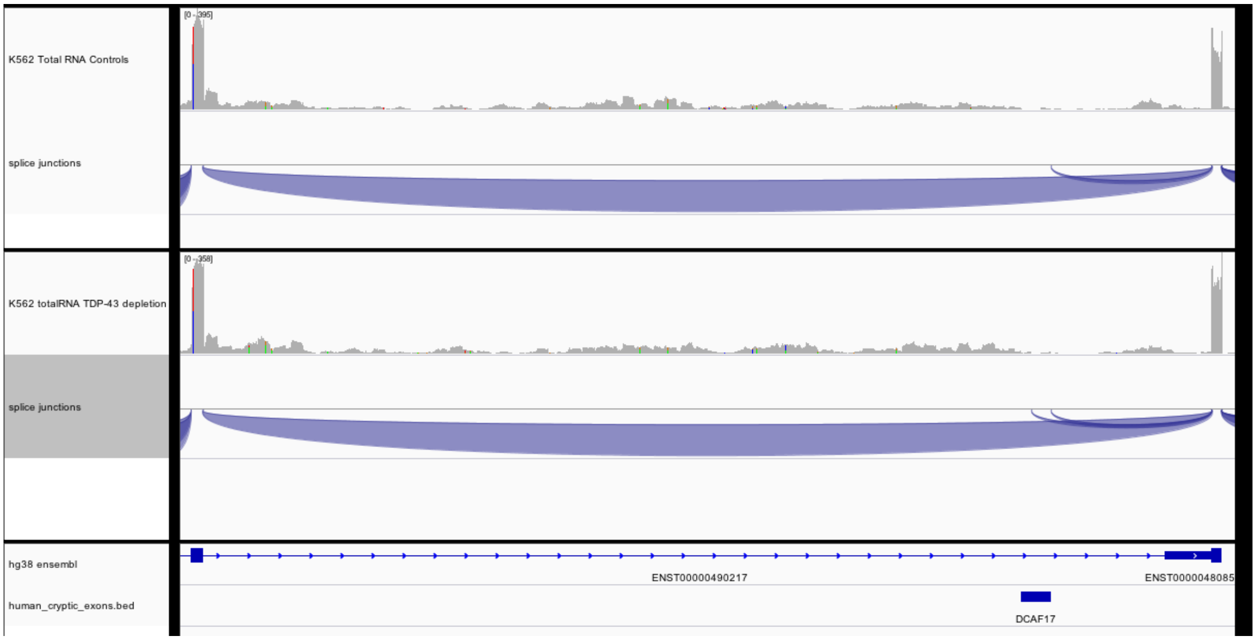

49 RP1-179N16-6 E002i2

Human K562 mRNA

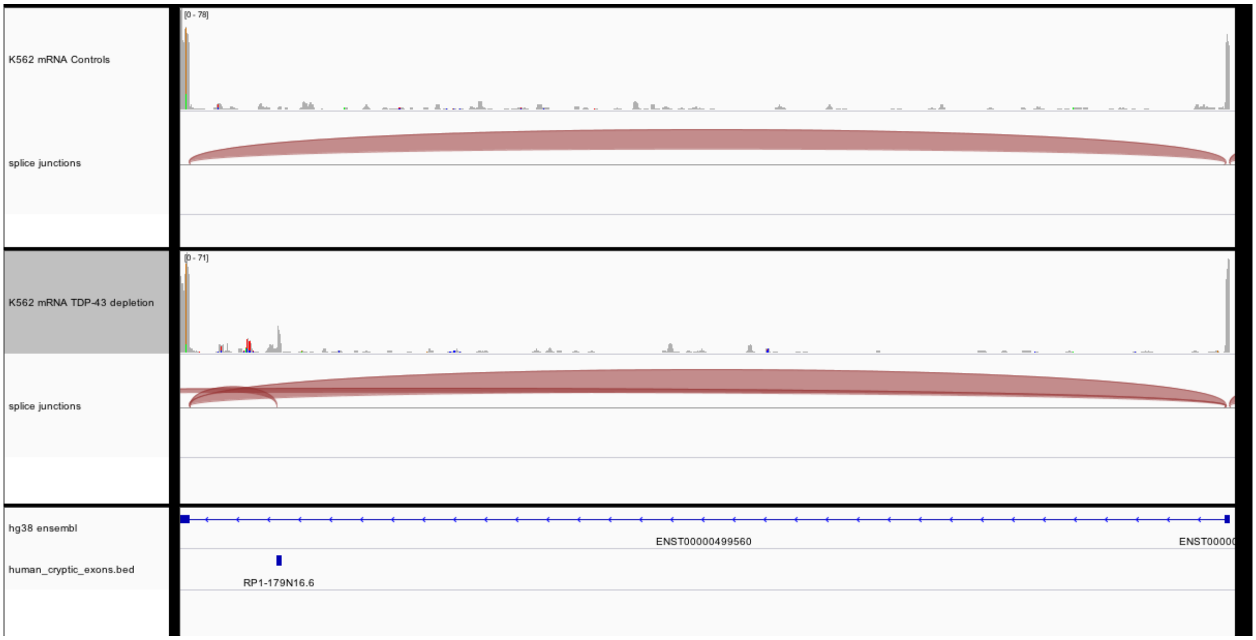

Human K562 total RNA

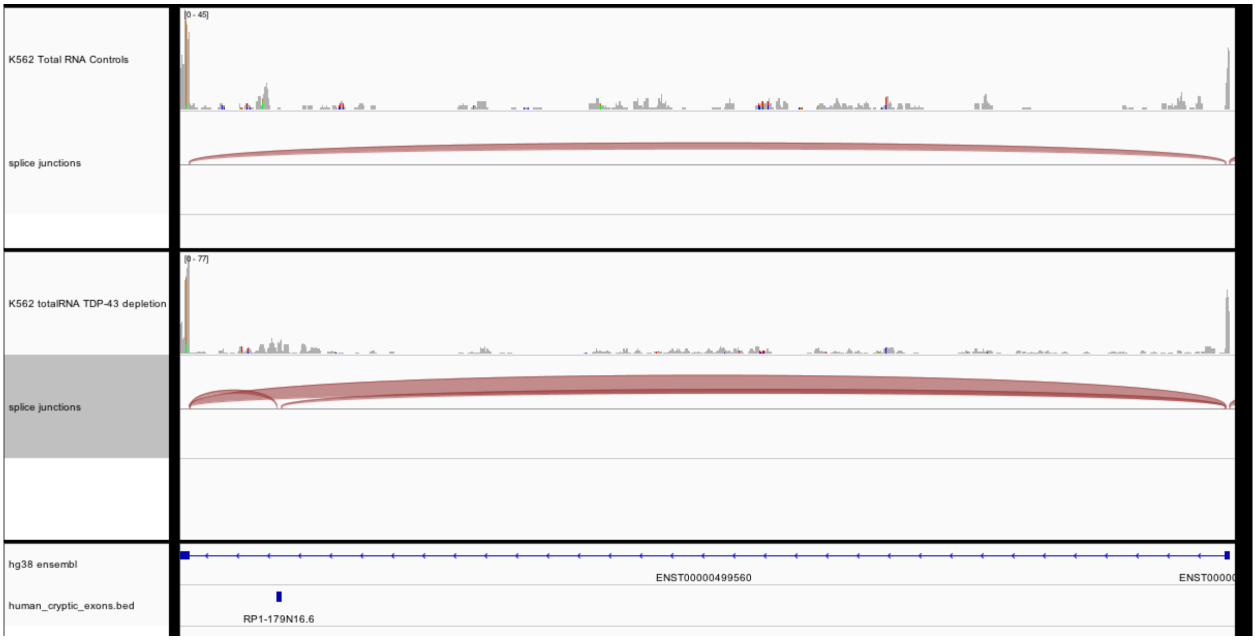

50 RNFT2 E016i2

Human K562 mRNA

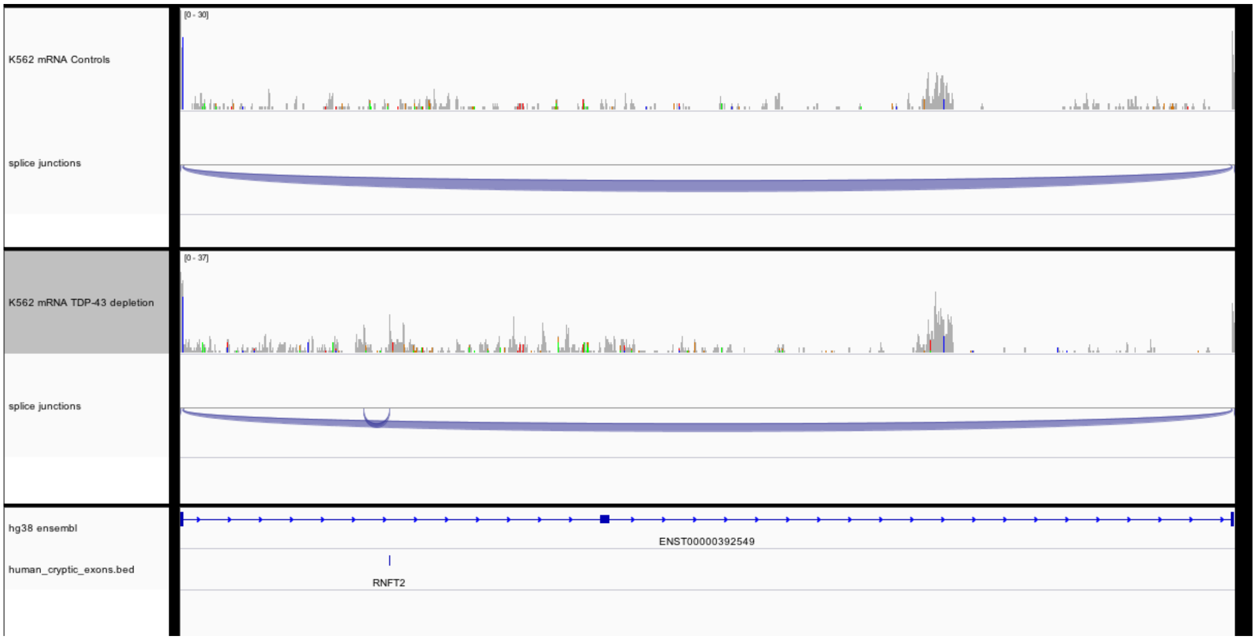

Human K562 total RNA

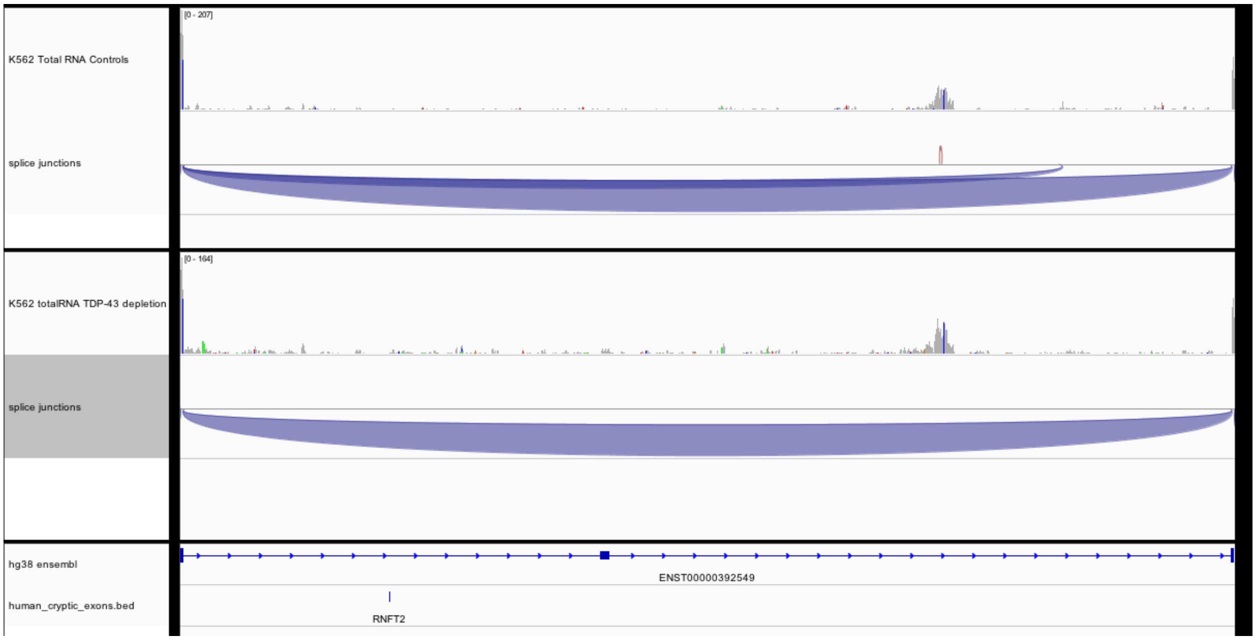

51 SH2B3 E005i1

Human K562 mRNA

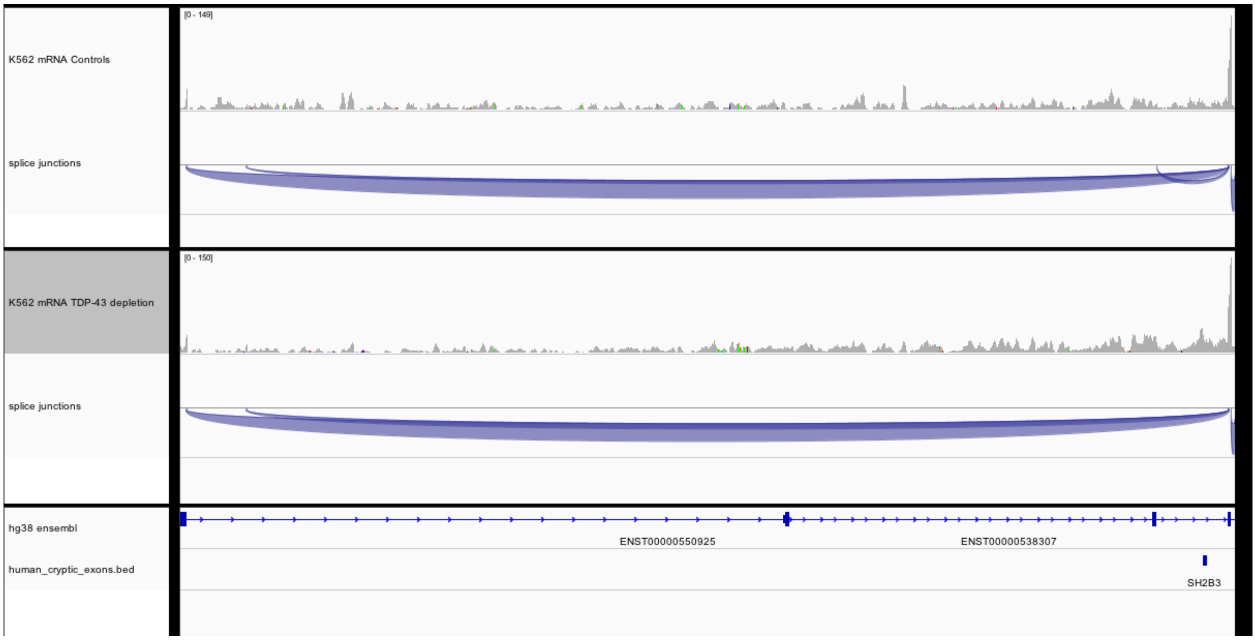

Human K562 total RNA

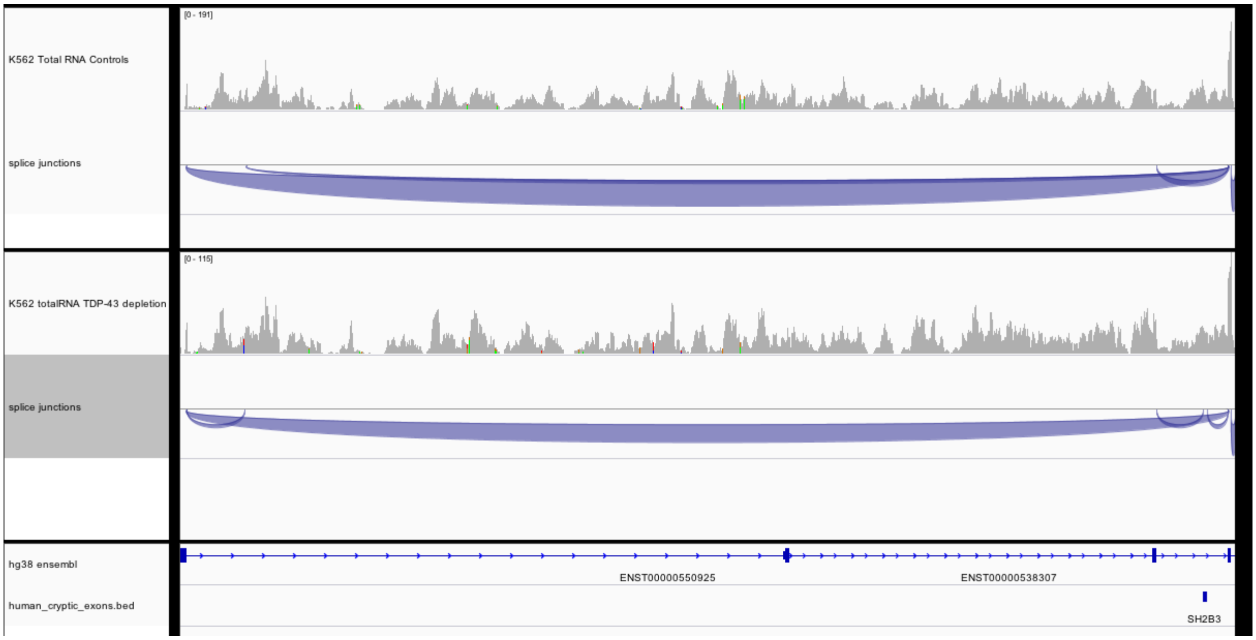

52 SENP7 E020i1

Human K562 mRNA

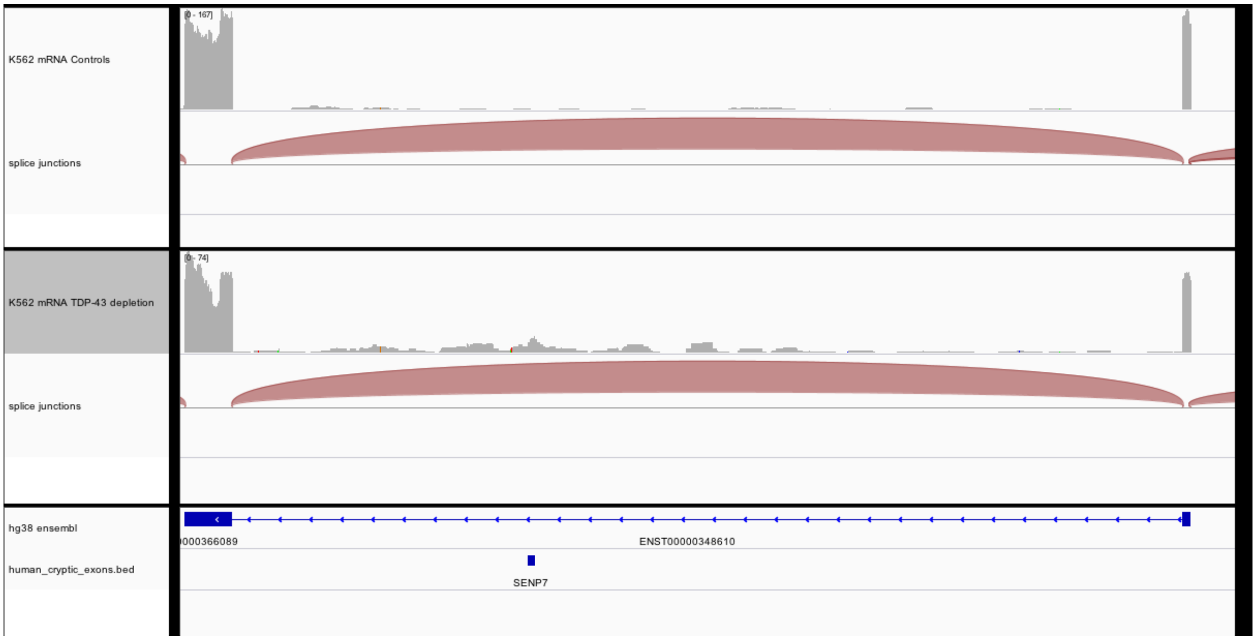

Human K562 total RNA

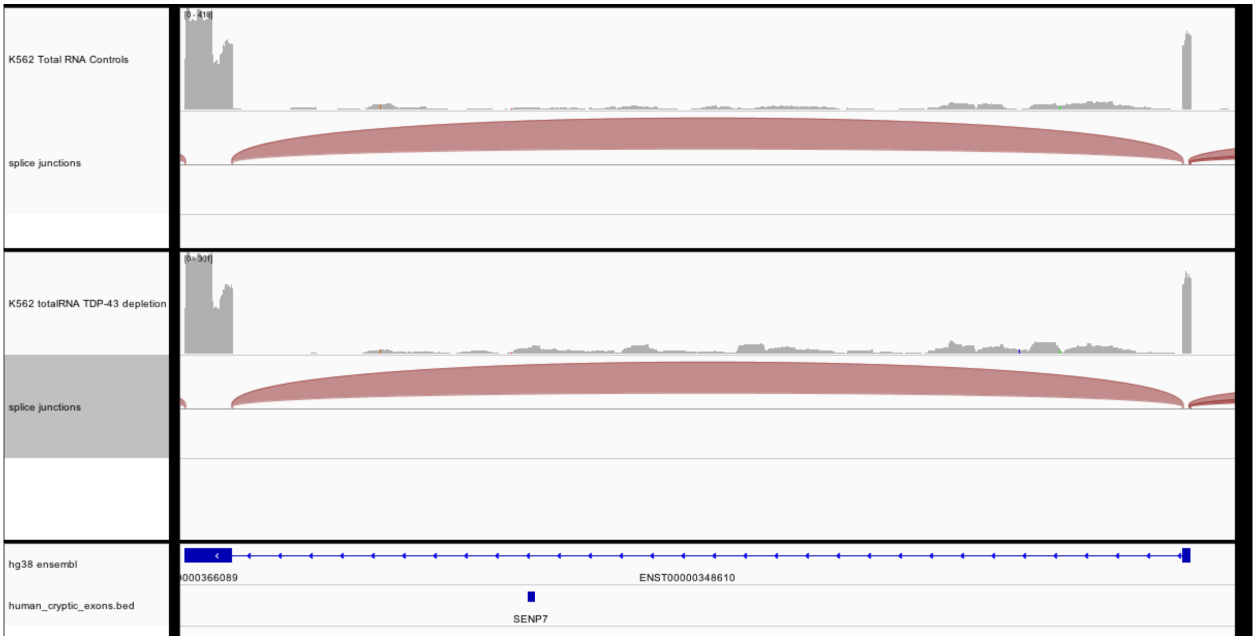

53 GOLGB1 E013i1

Human K562 mRNA

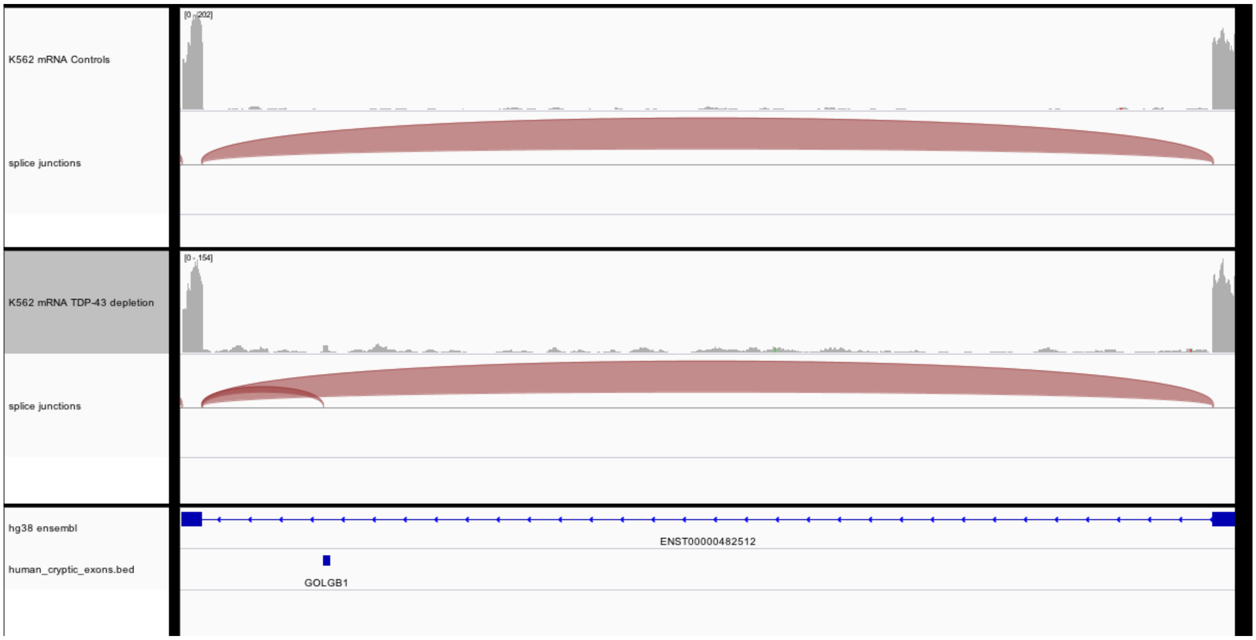

Human K562 total RNA

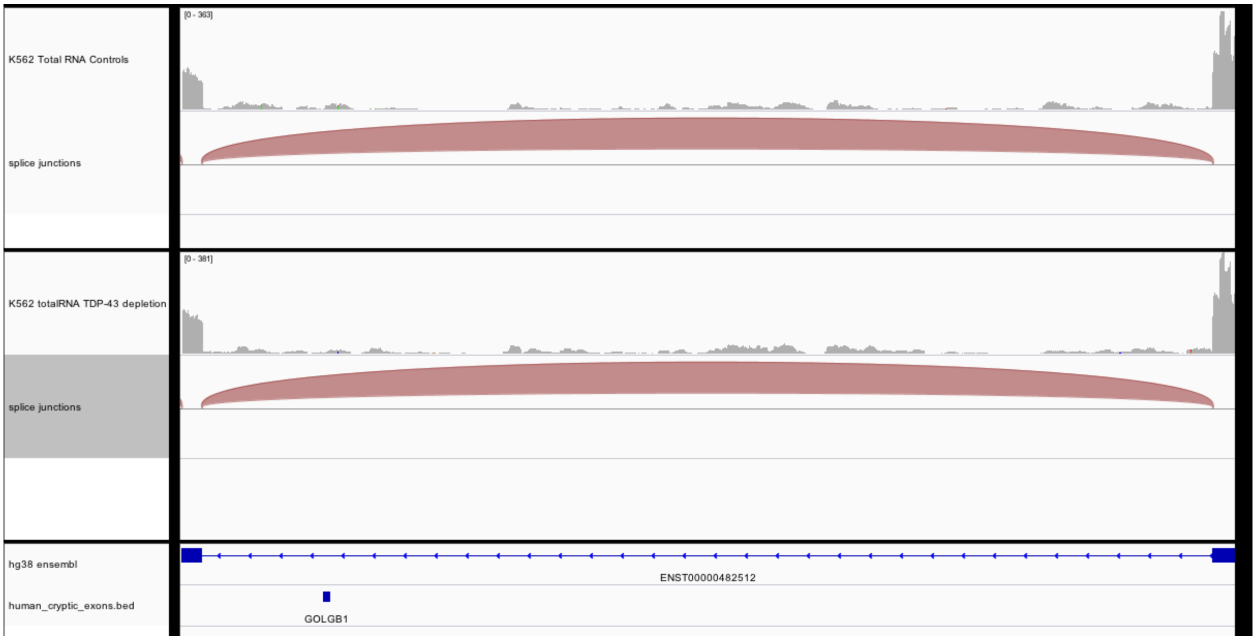

54 AGRN E015i1

Human K562 mRNA

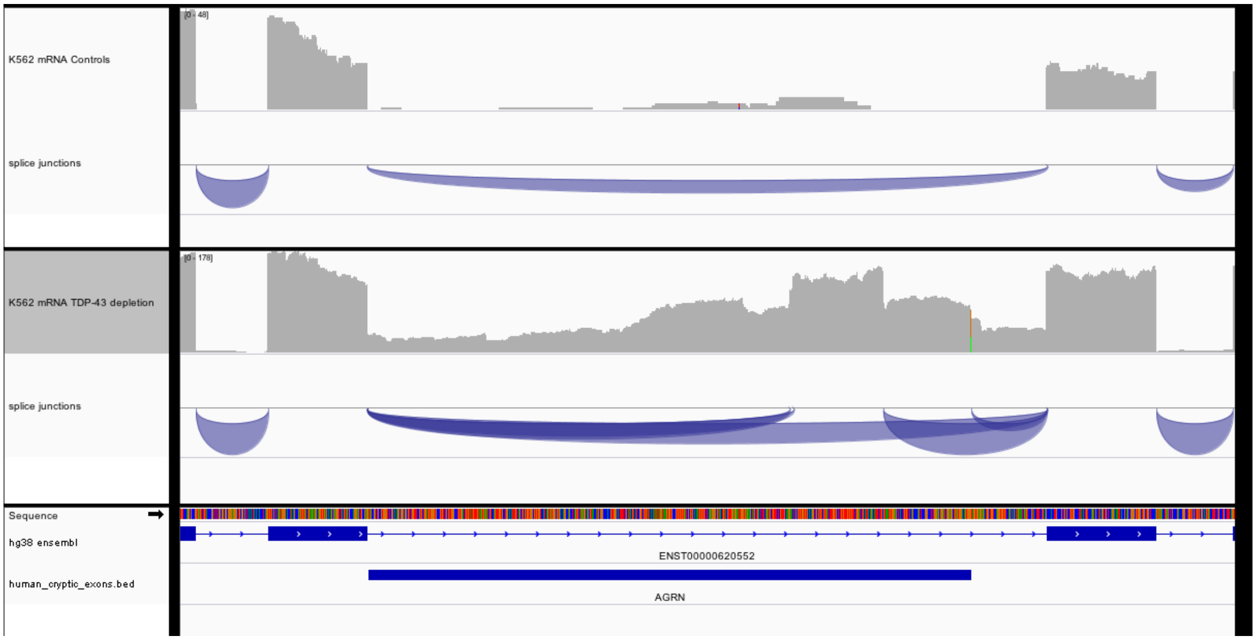

Human K562 total RNA

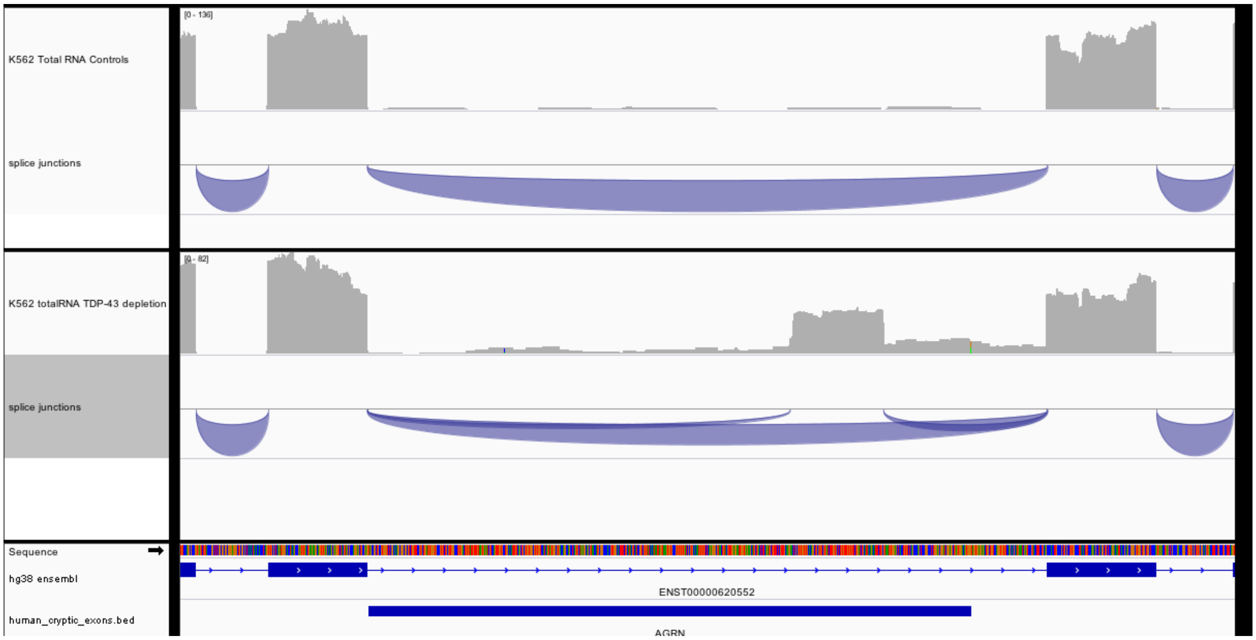

55 CEP72 E012i1

Human K562 mRNA

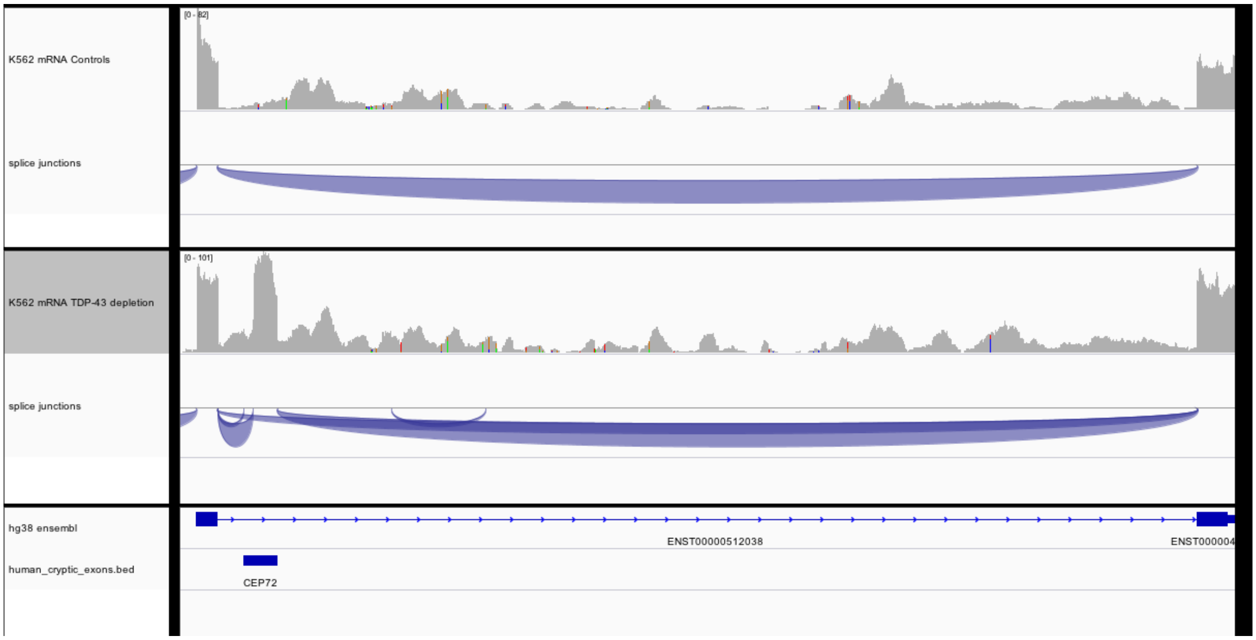

Human K562 total RNA

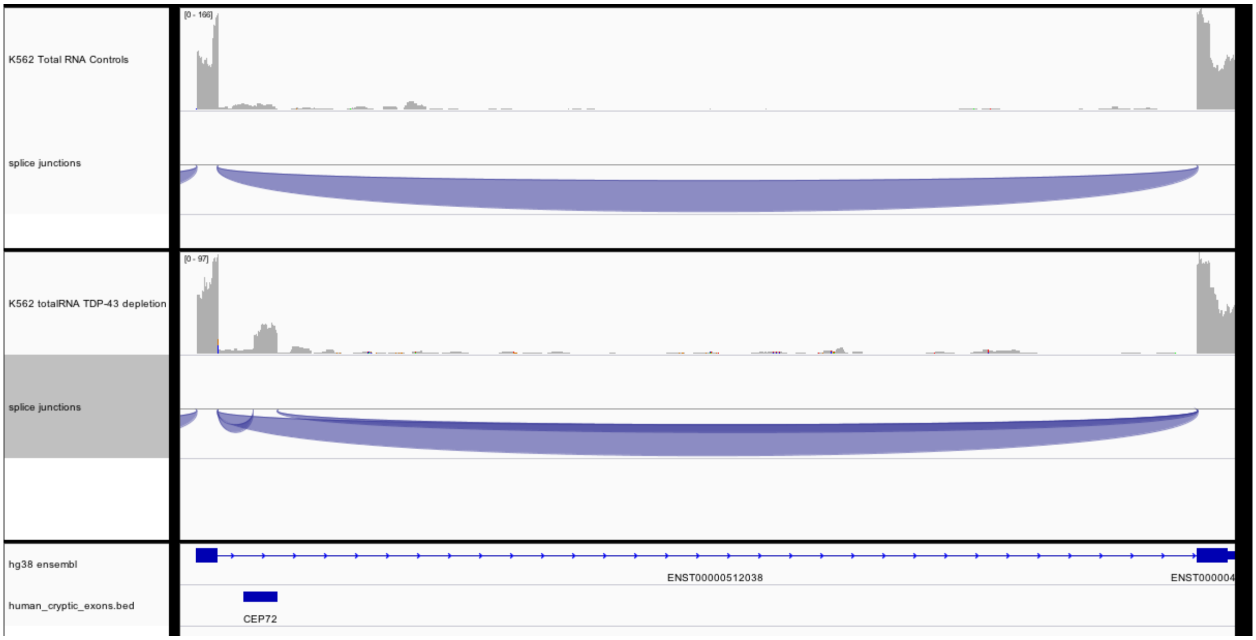

56 RAP1GAP E010i1

Human K562 mRNA

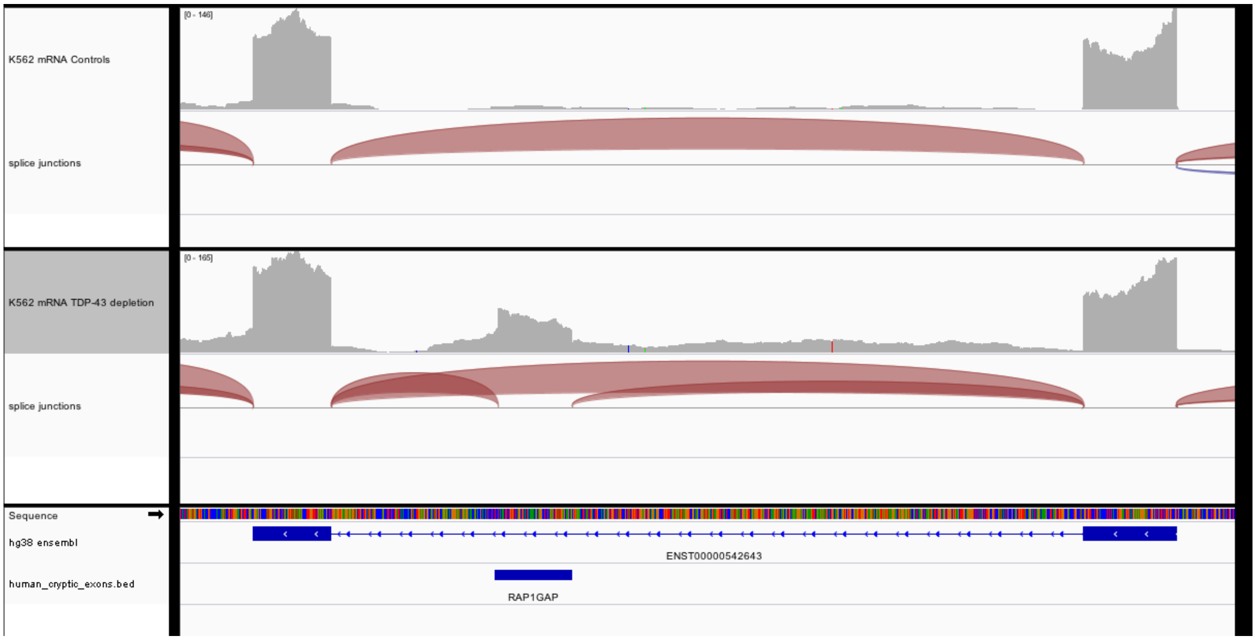

Human K562 total RNA

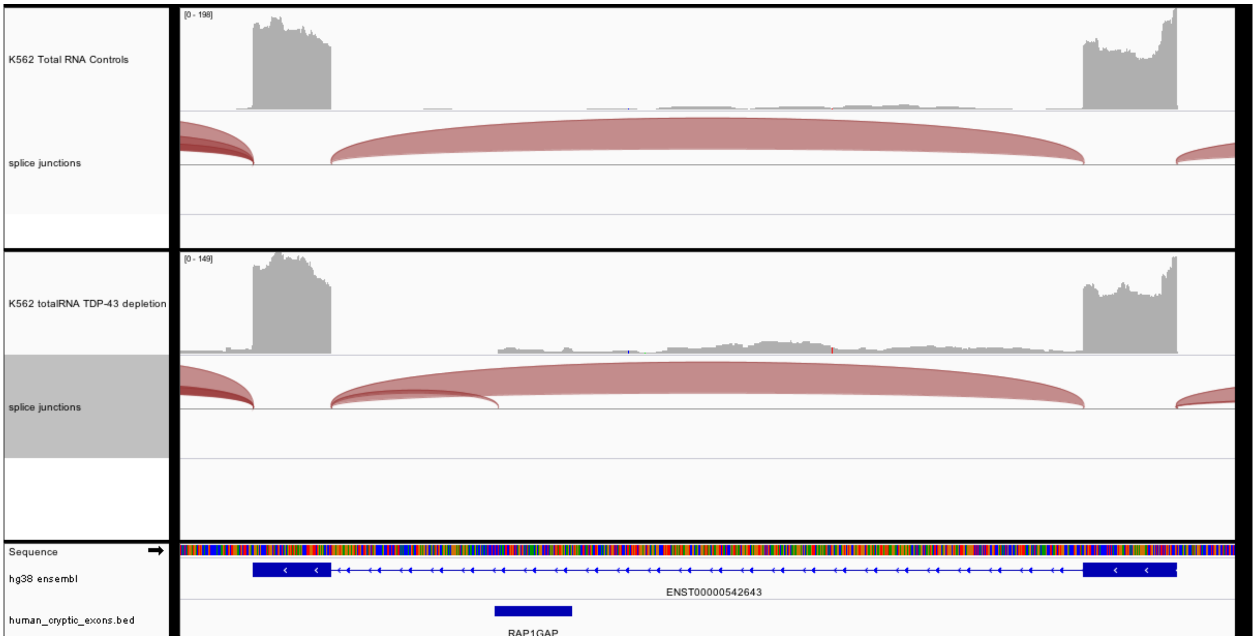

57 PFKP E010i1

Human K562 mRNA

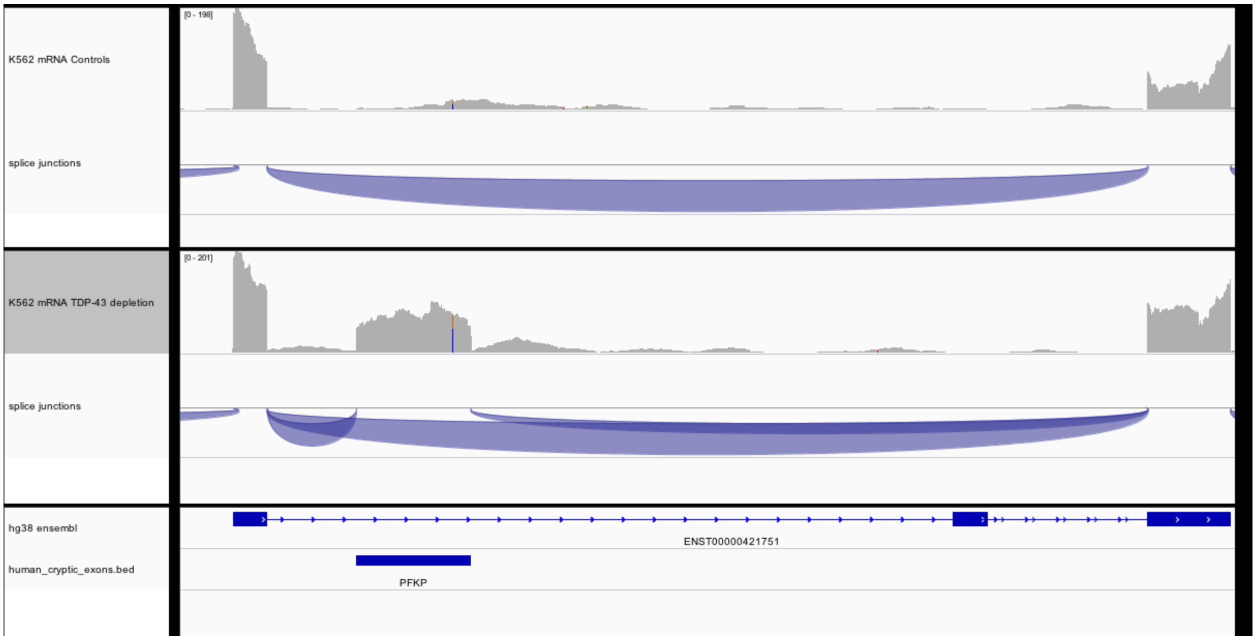

Human K562 total RNA

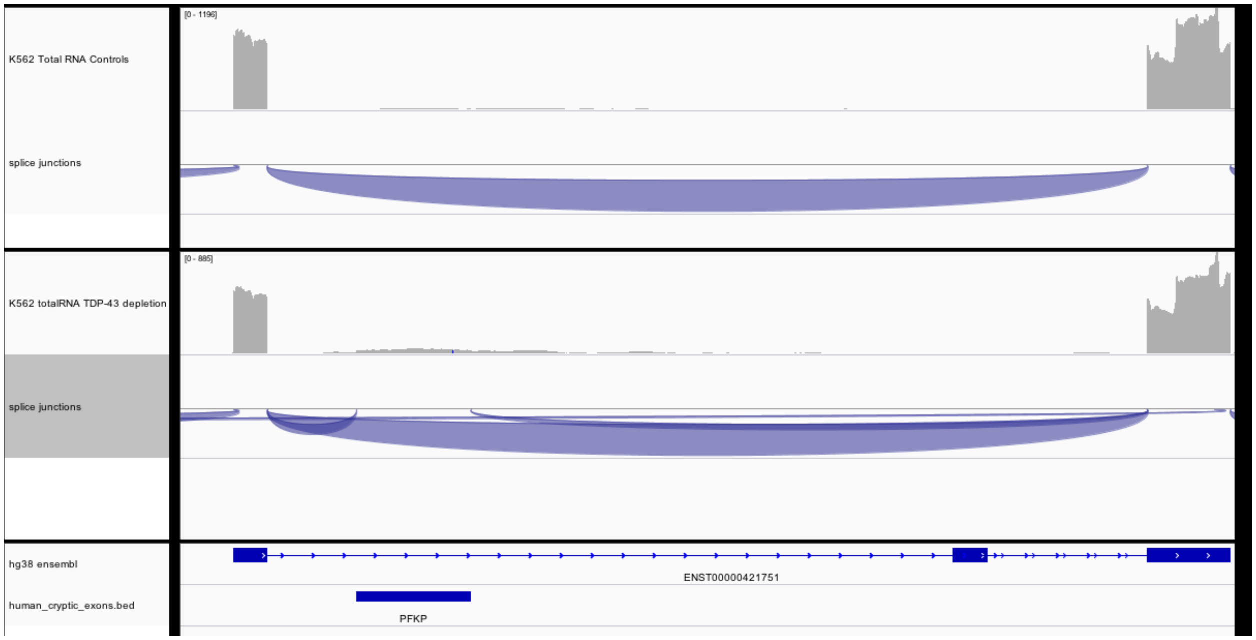

58 PKN1 E016i2

Human K562 mRNA

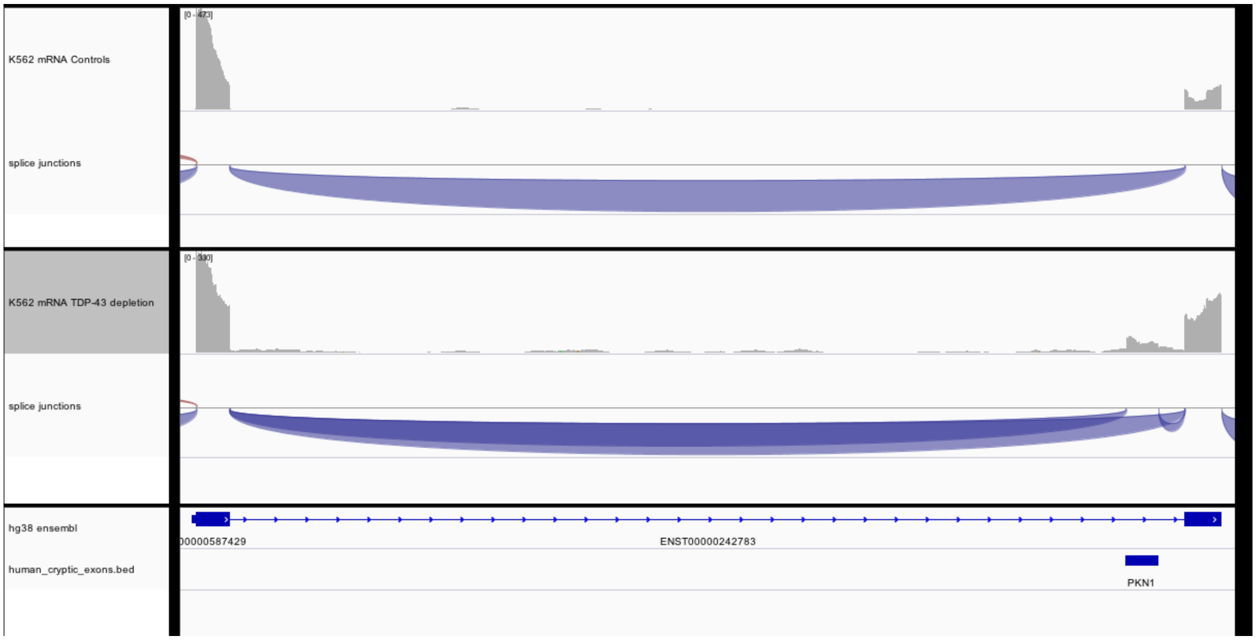

Human K562 total RNA

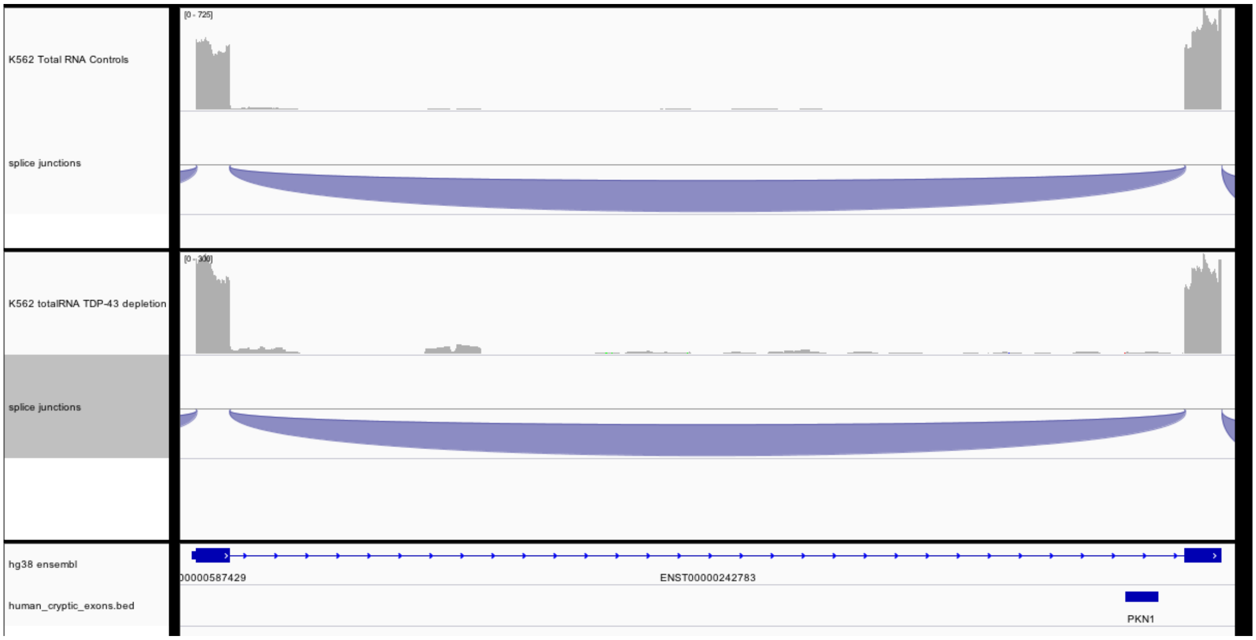

59 FAM114A2 E040i1

Human K562 mRNA

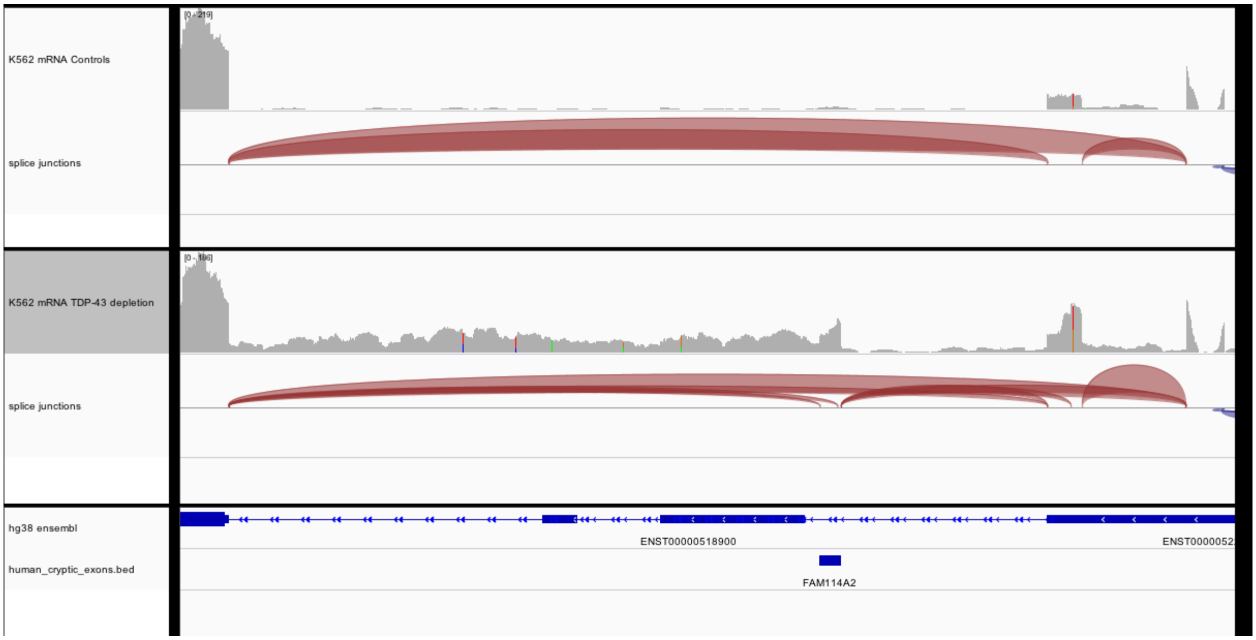

Human K562 total RNA

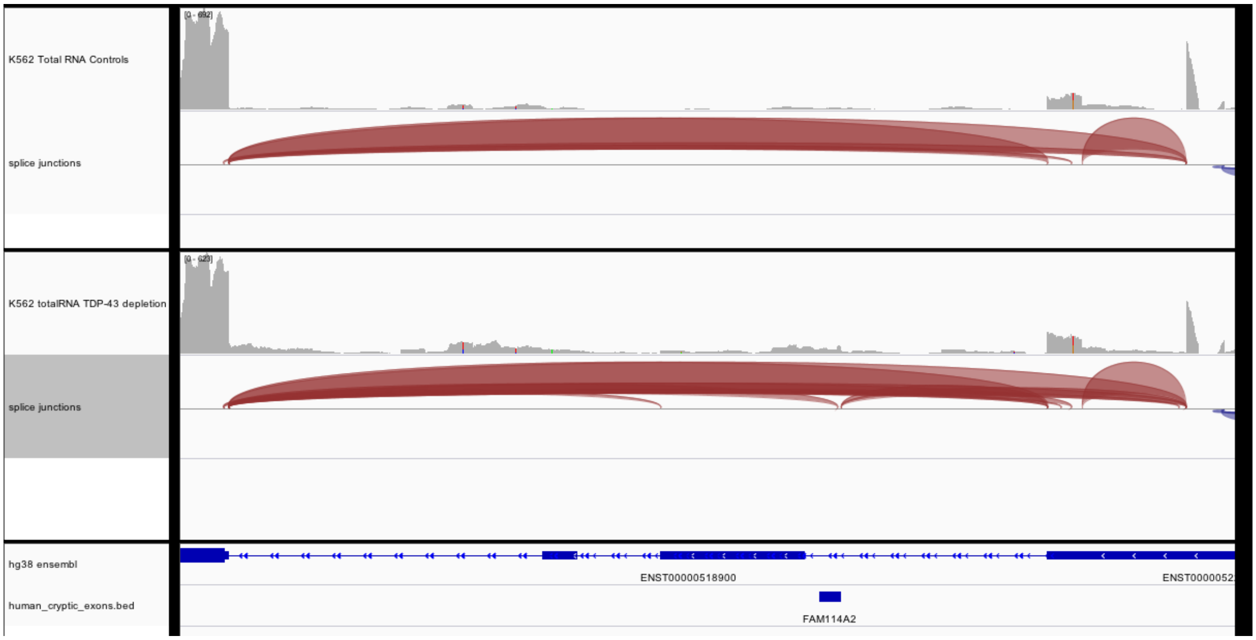

60 HDGFRP2 E015i1

Human K562 mRNA

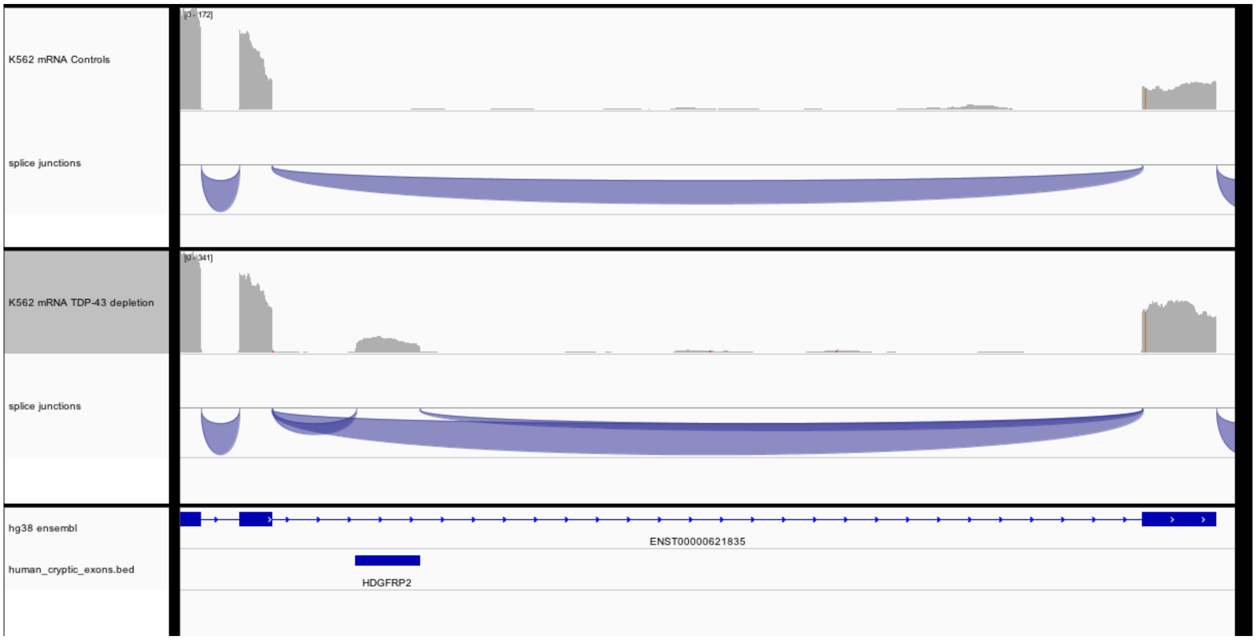

Human K562 total RNA

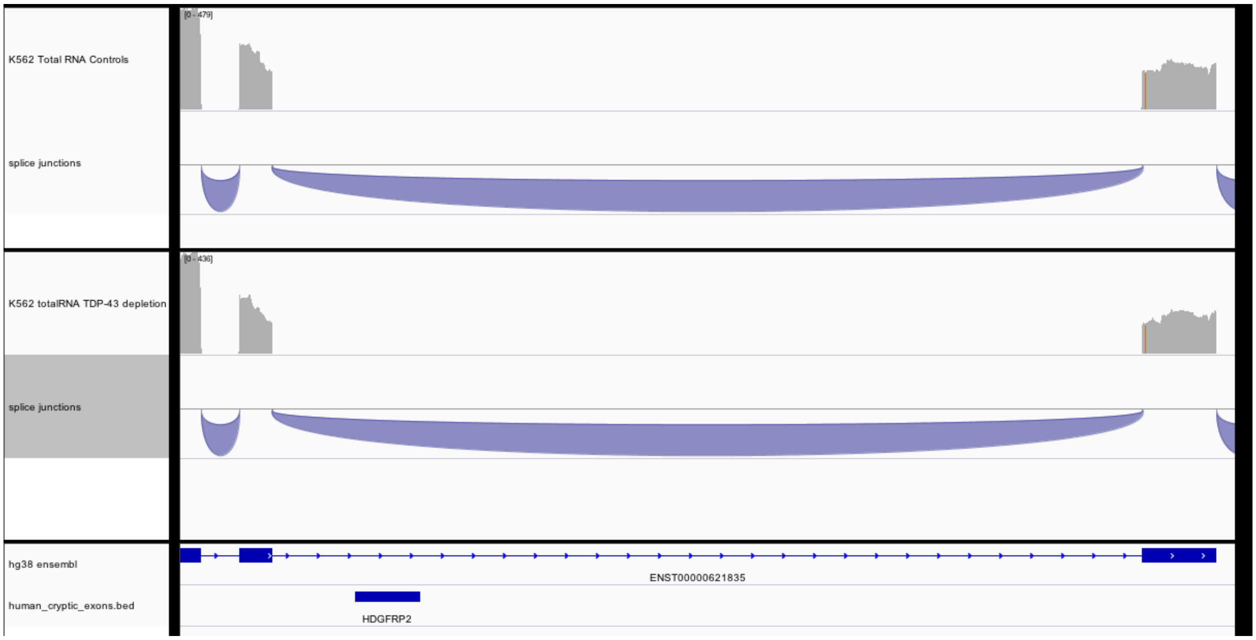

61 PRPF38A E003i1

Human K562 mRNA

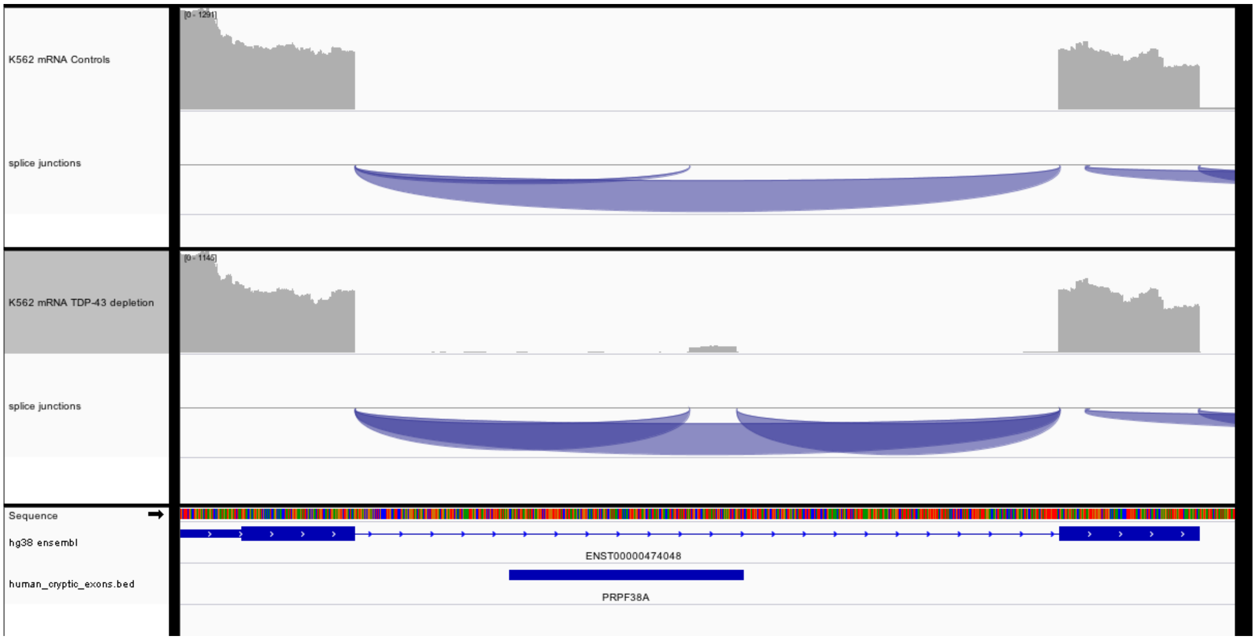

Human K562 total RNA

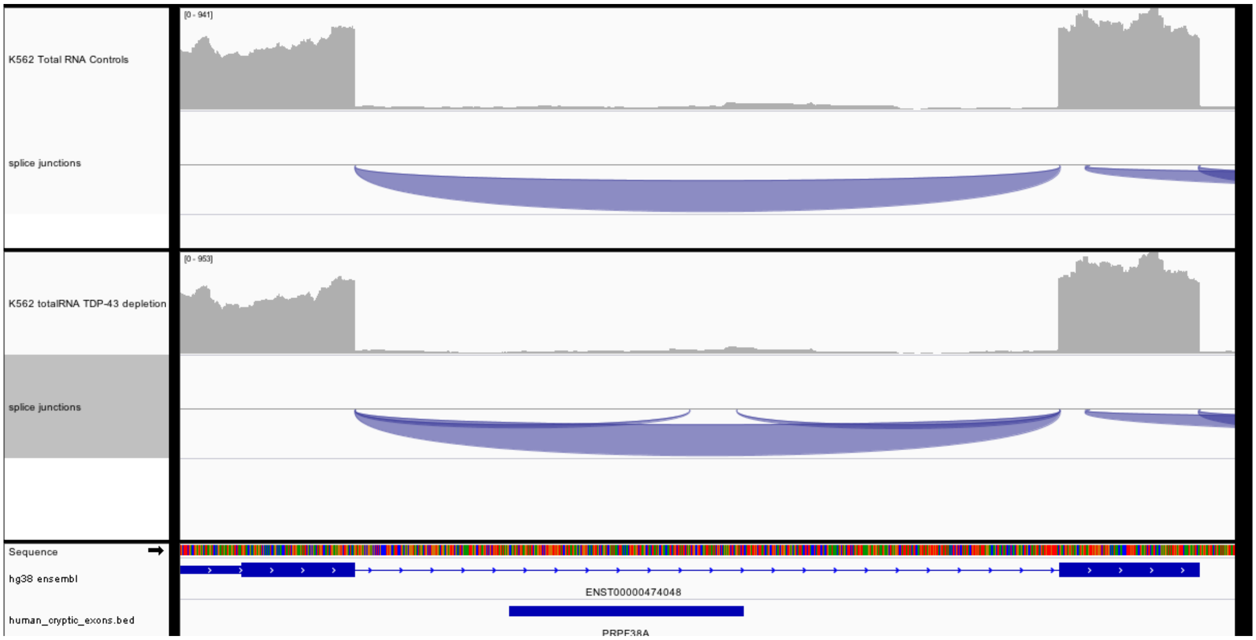

62 ATG4B E047i1

Human K562 mRNA

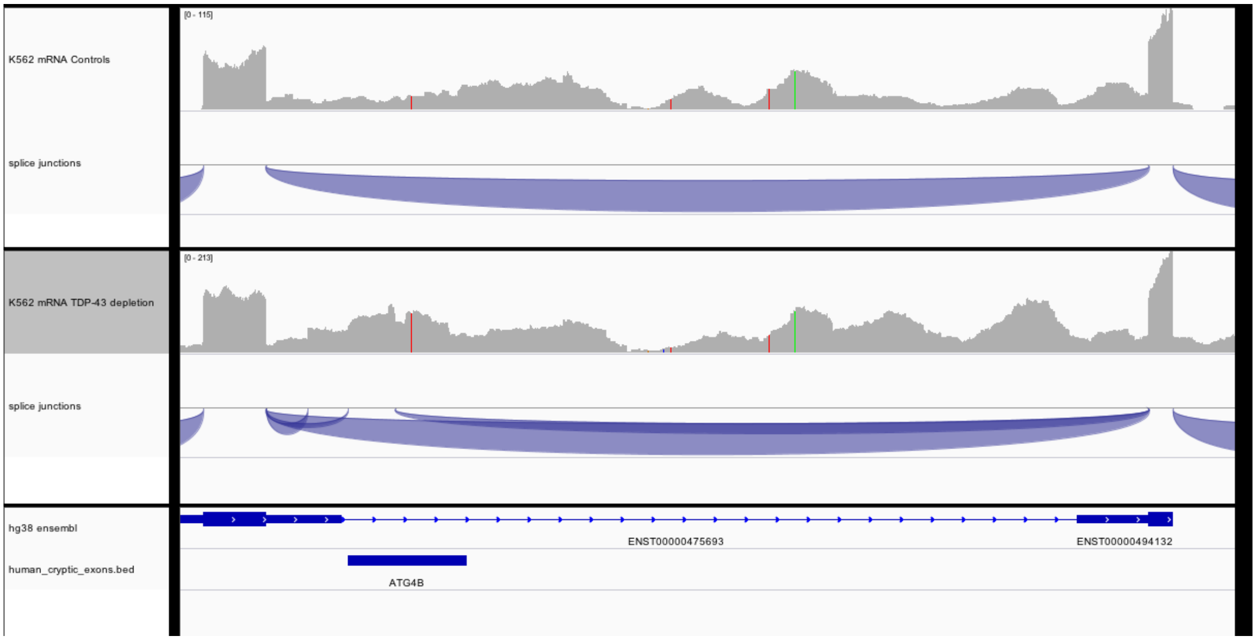

Human K562 total RNA

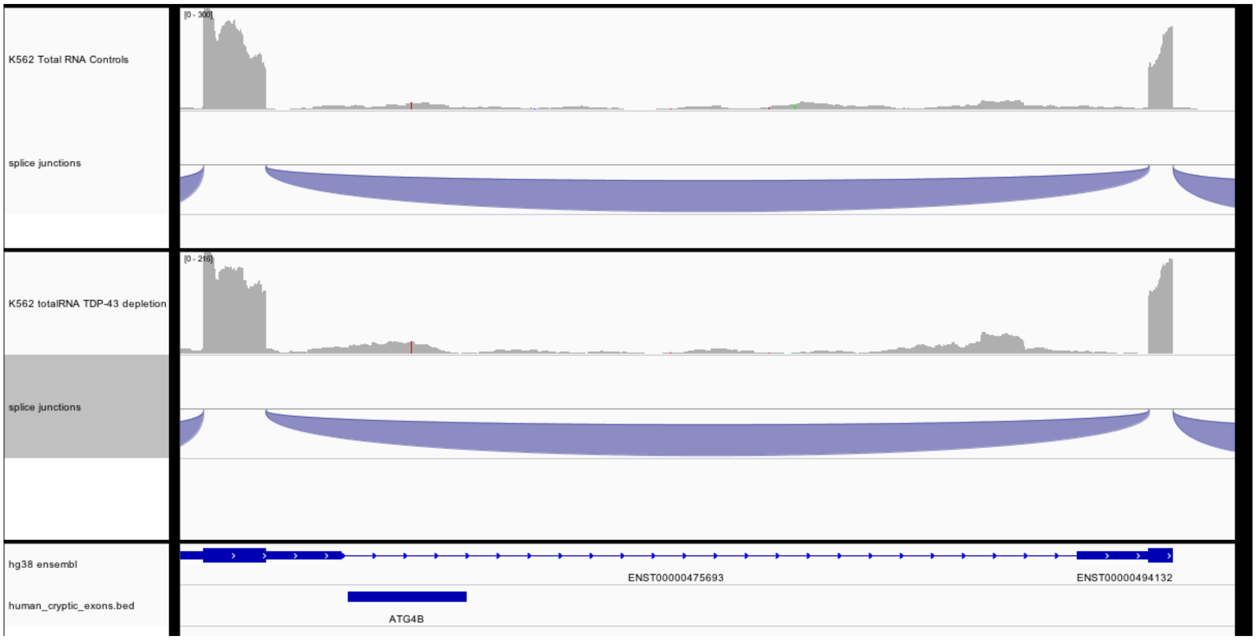

63 ADSS E020i1

Human K562 mRNA

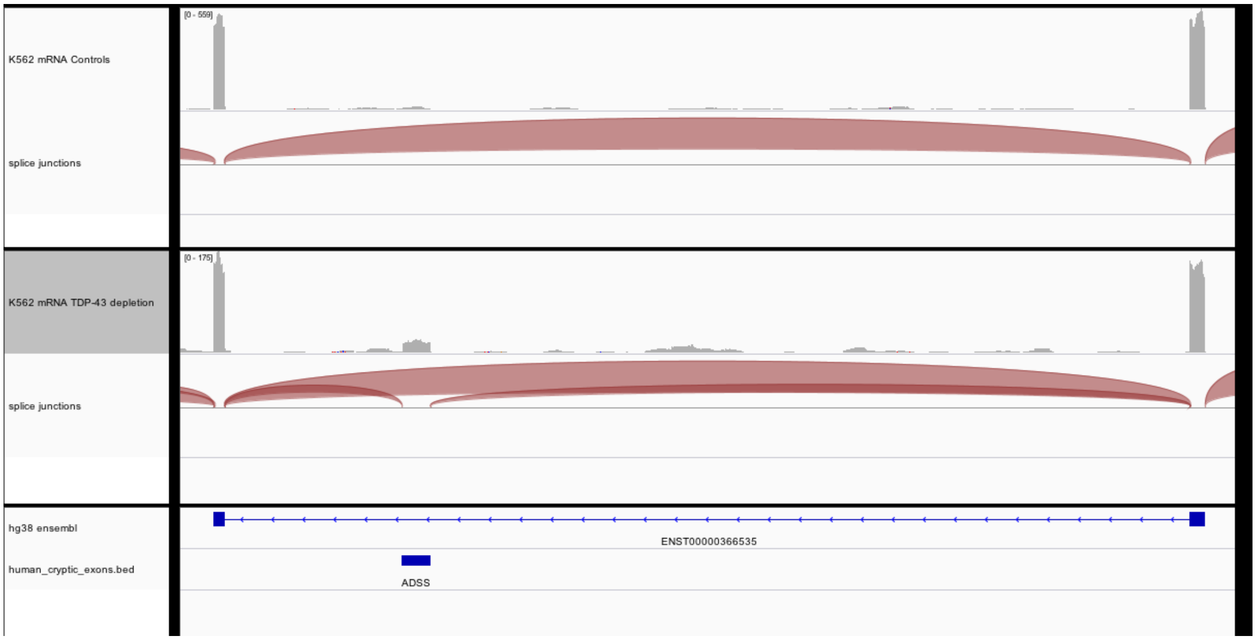

Human K562 total RNA

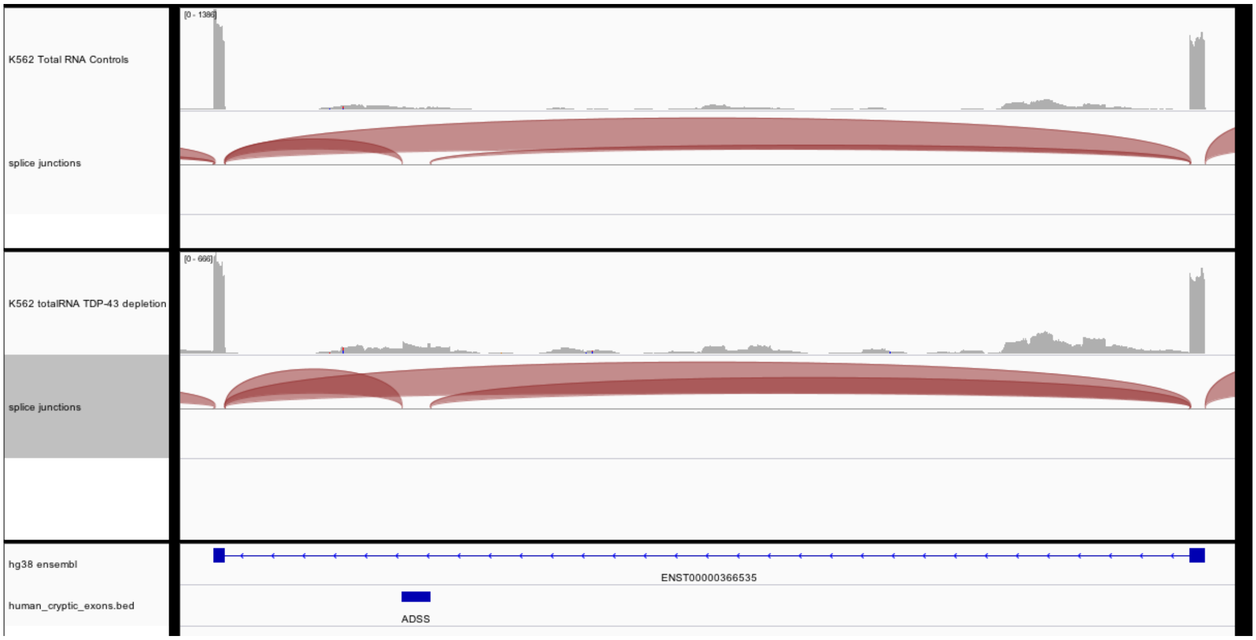

64 NSUN2 E024i1

Human K562 mRNA

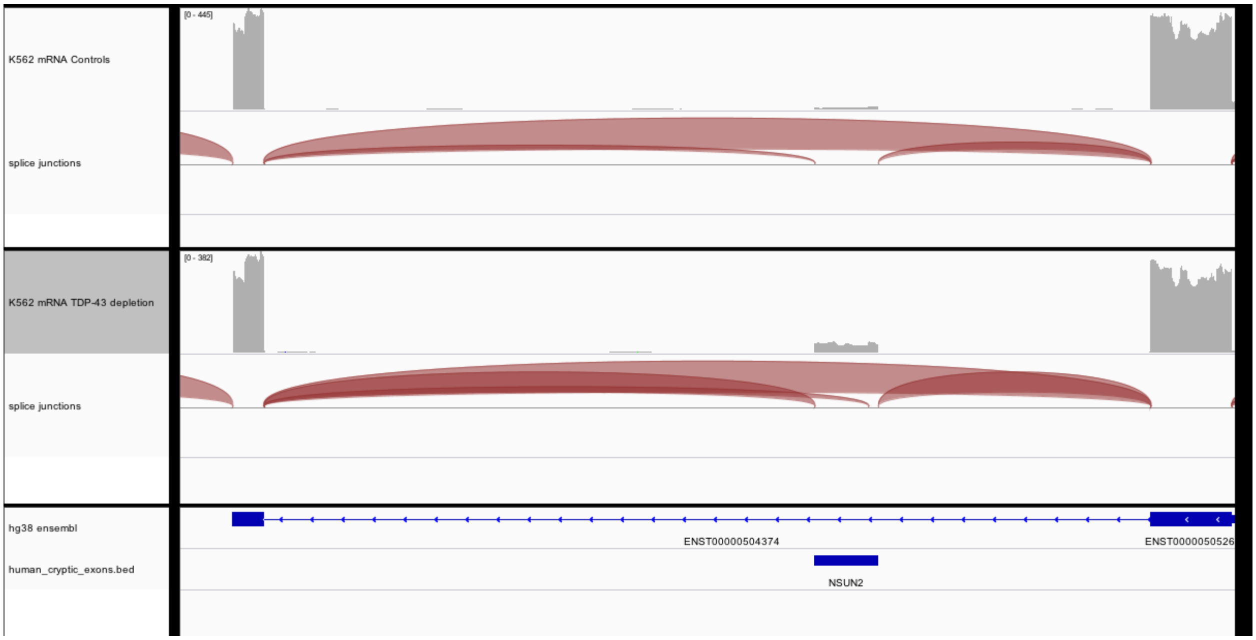

Human K562 total RNA

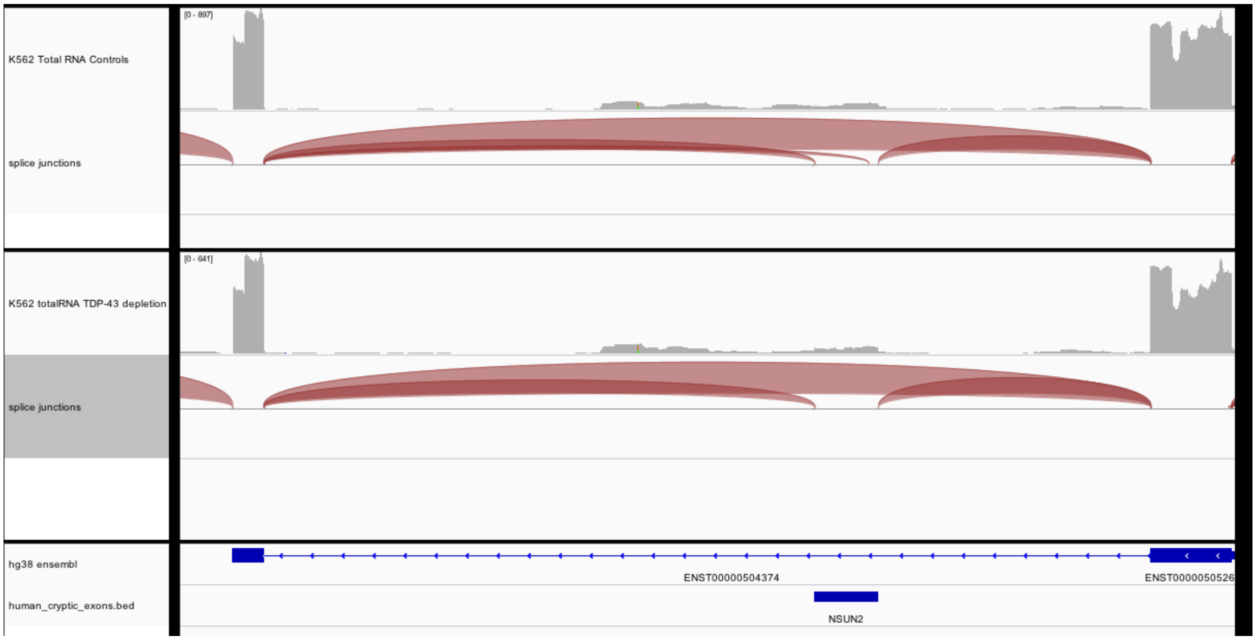

65 TAOK3 E007i1

Human K562 mRNA

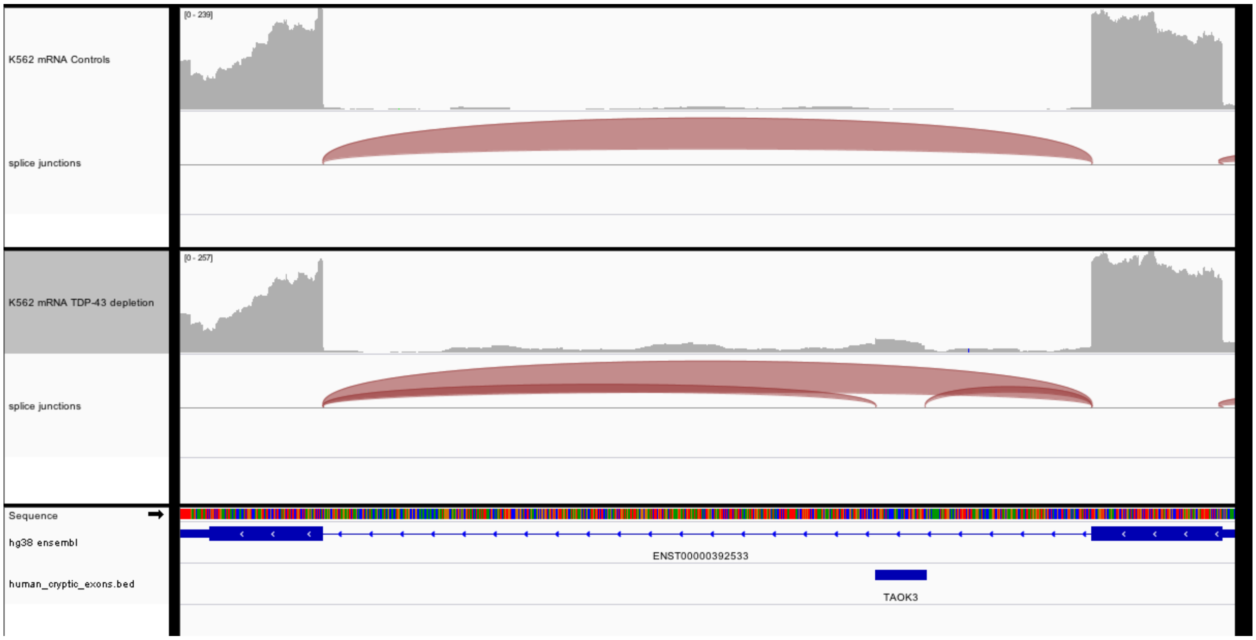

Human K562 total RNA

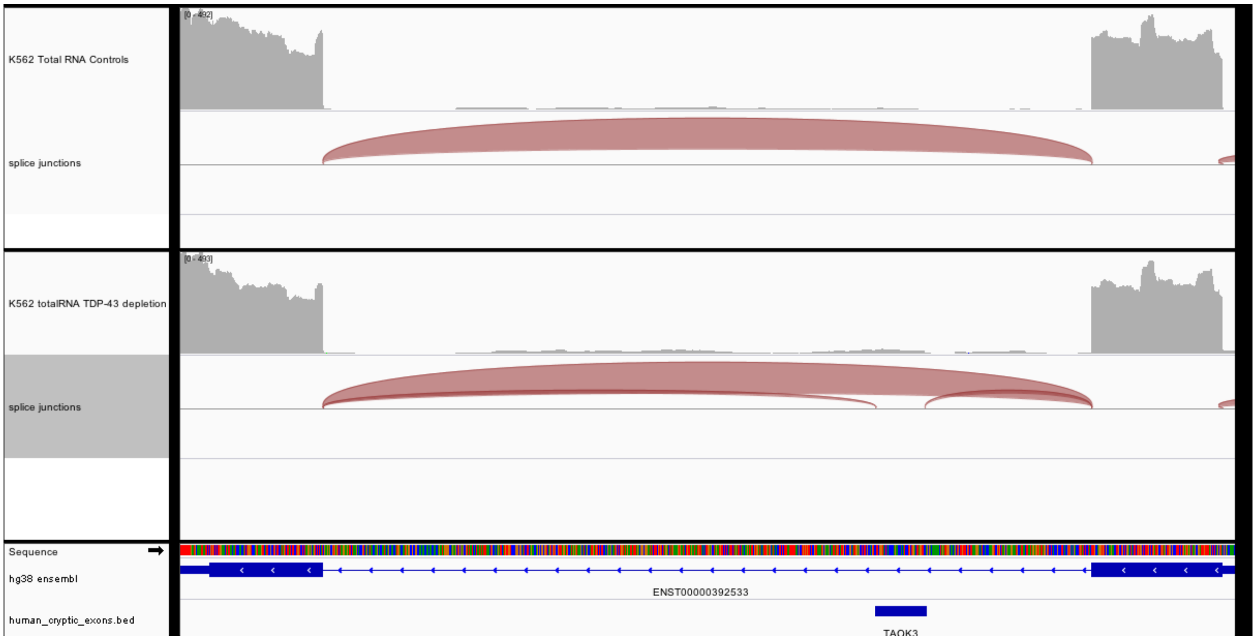

66 CORO7-PAM16+CORO7 E081i1

Human K562 mRNA

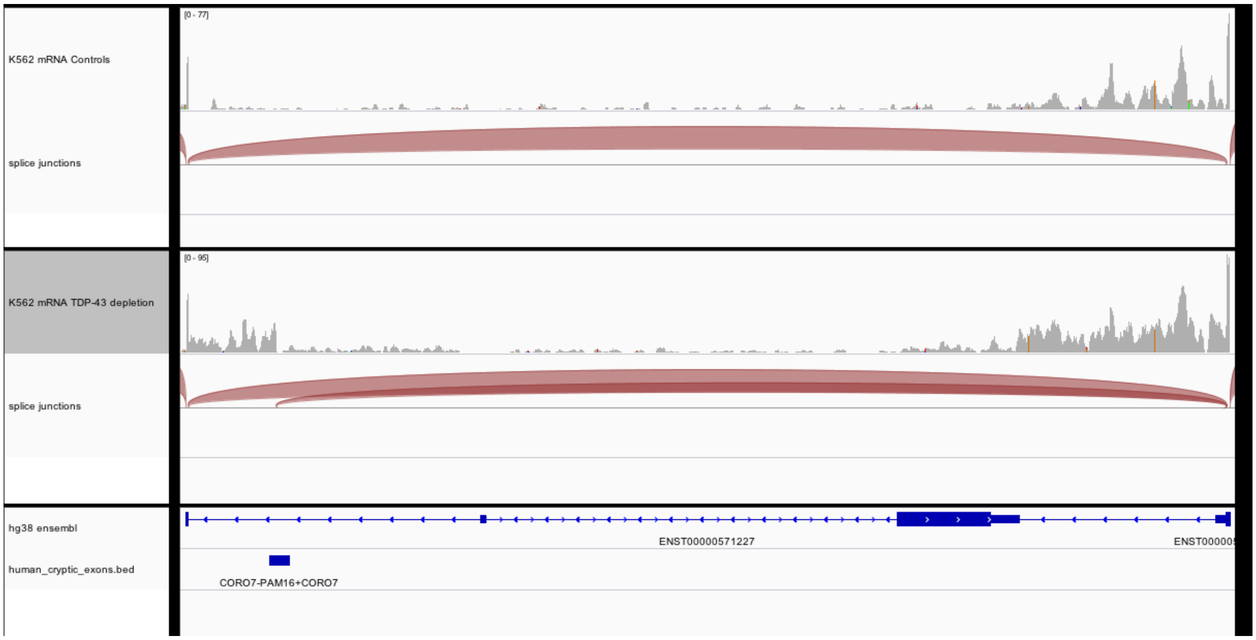

Human K562 total RNA

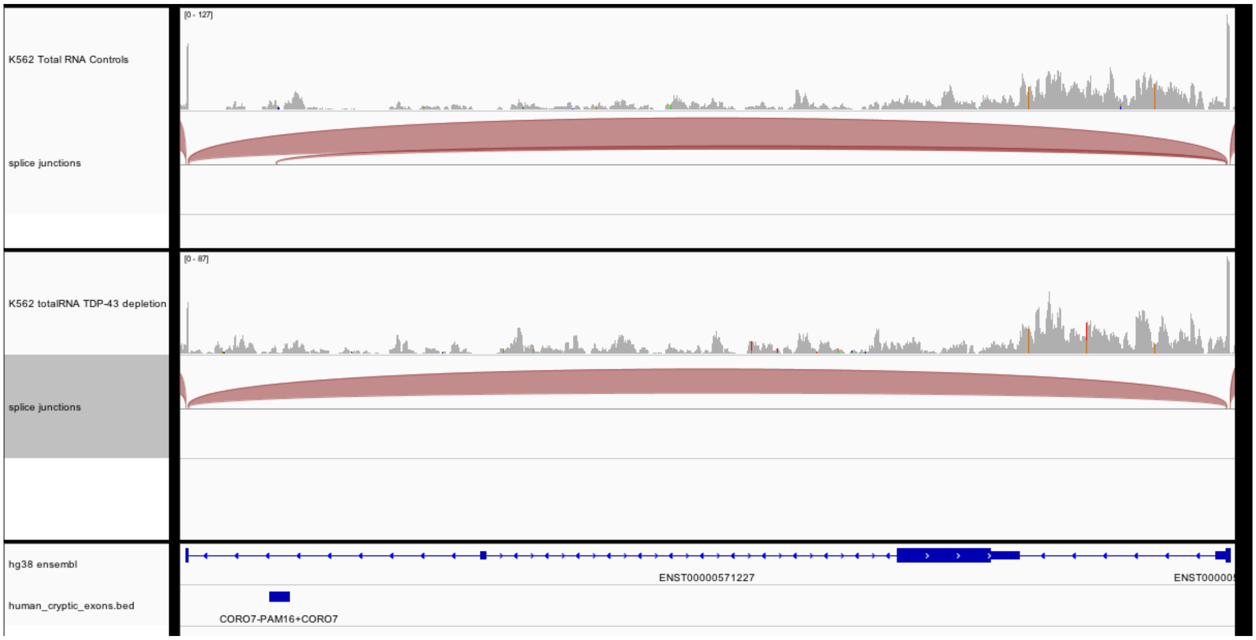

67 HBG2 E016i1

Human K562 mRNA

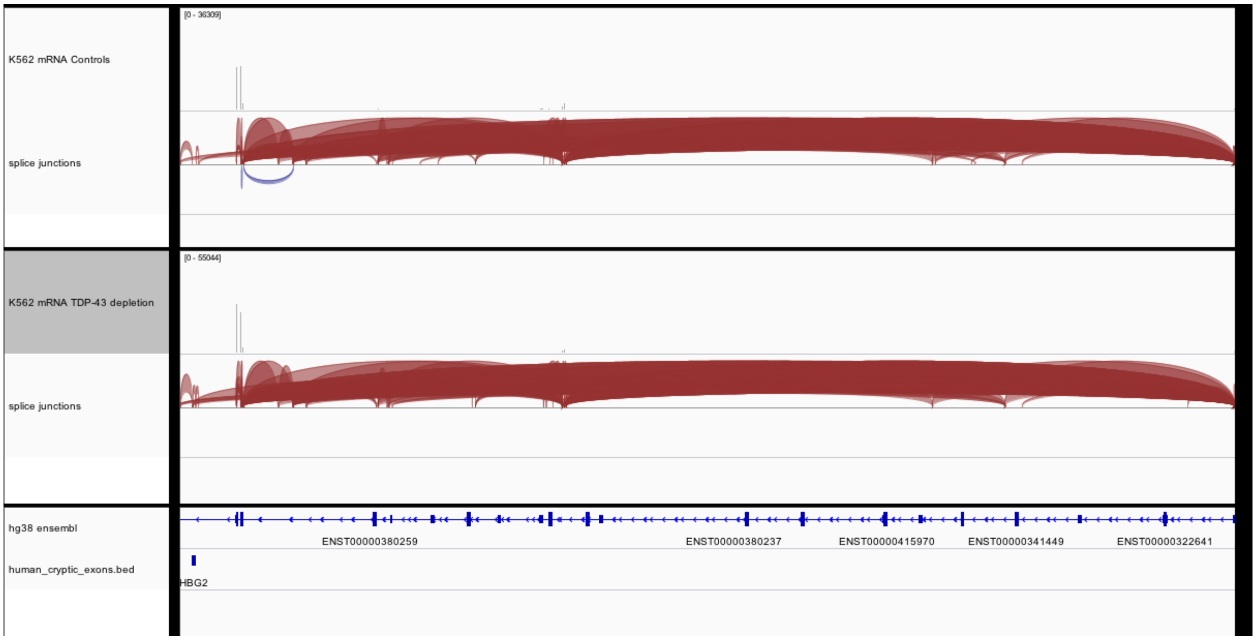

Human K562 total RNA

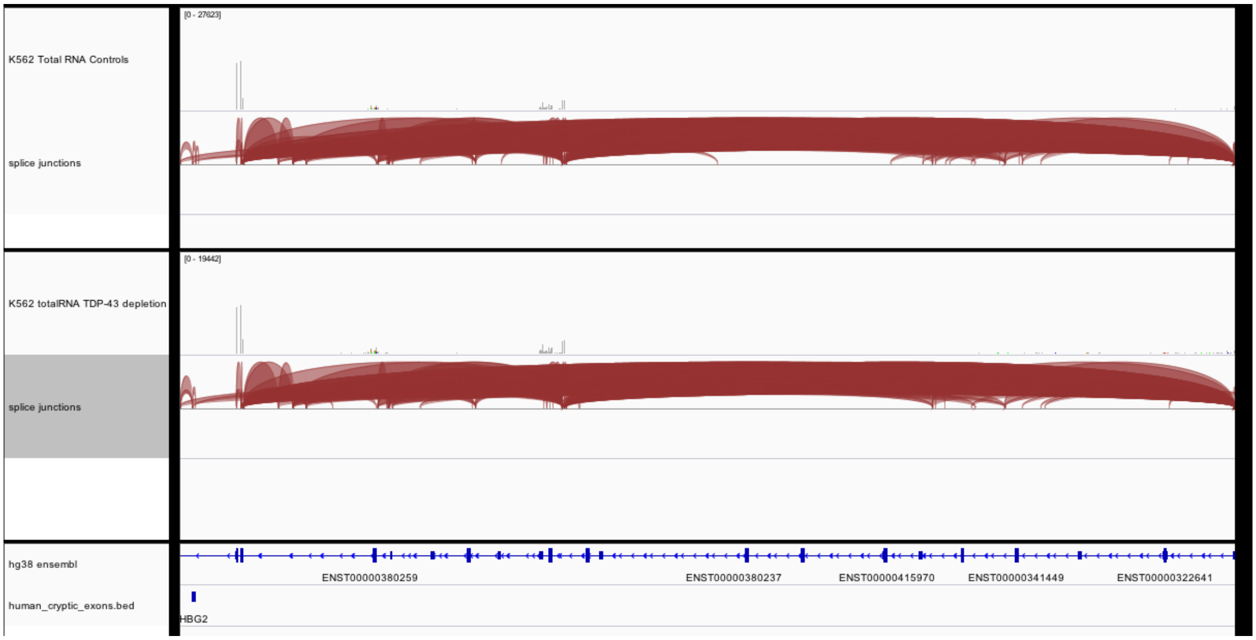

68 SSFA2 E024i1

Human K562 mRNA

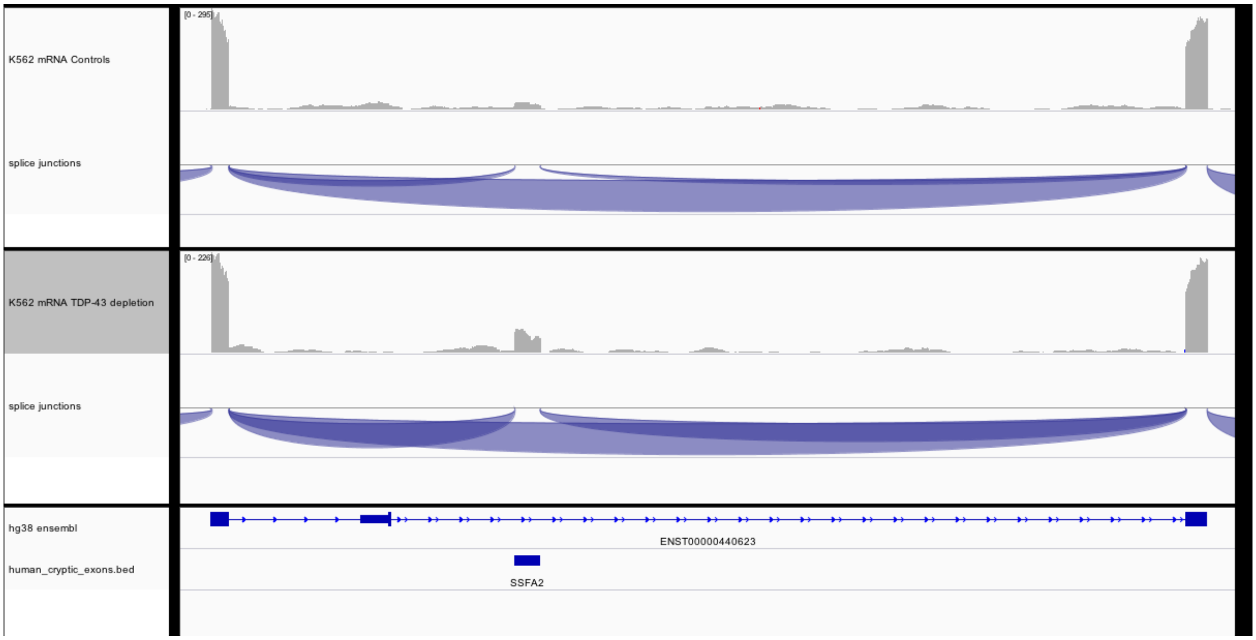

Human K562 total RNA

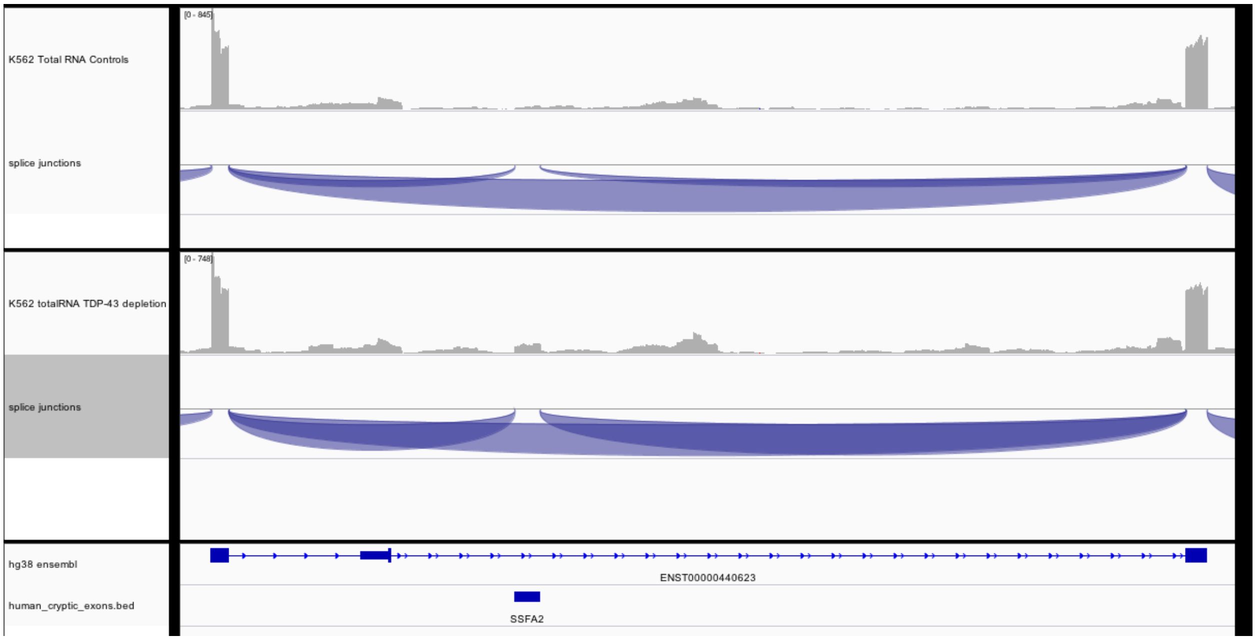

69 MARCH3 E009i1

Human K562 mRNA

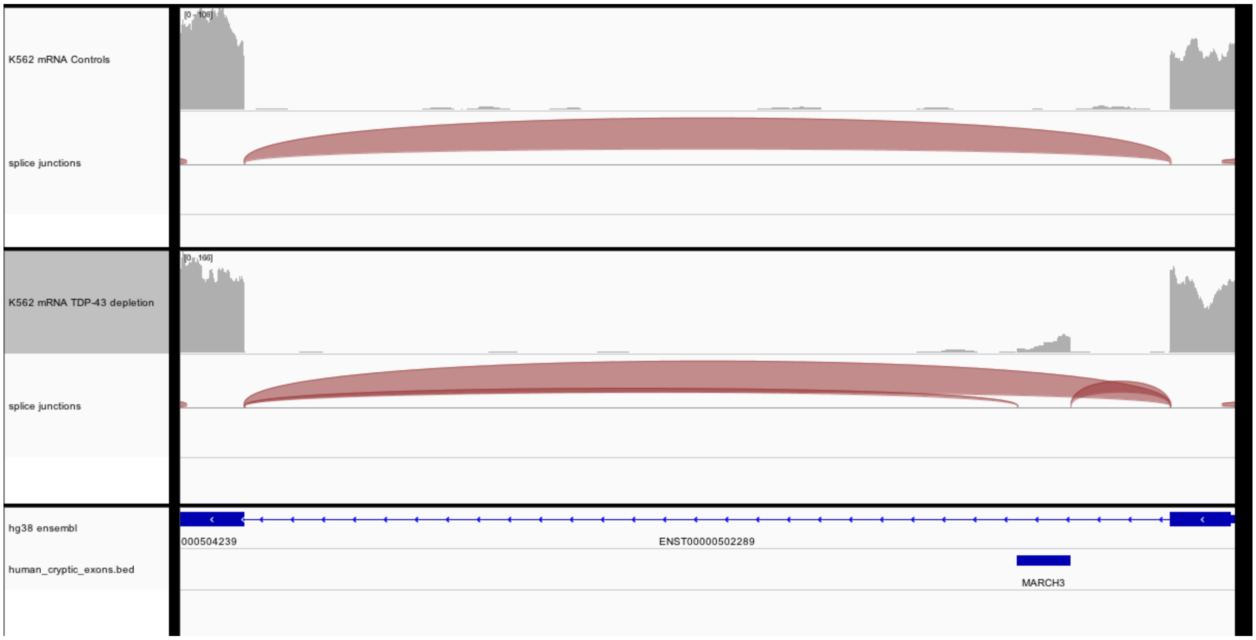

Human K562 total RNA

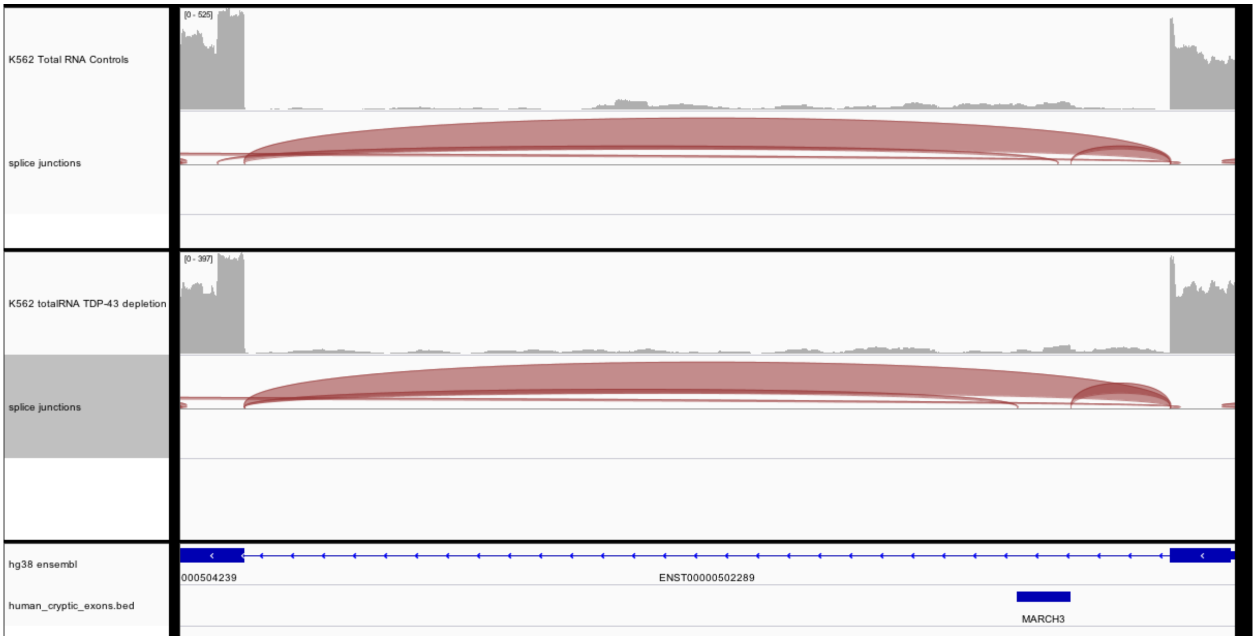

70 HERC1 E014i1

Human K562 mRNA

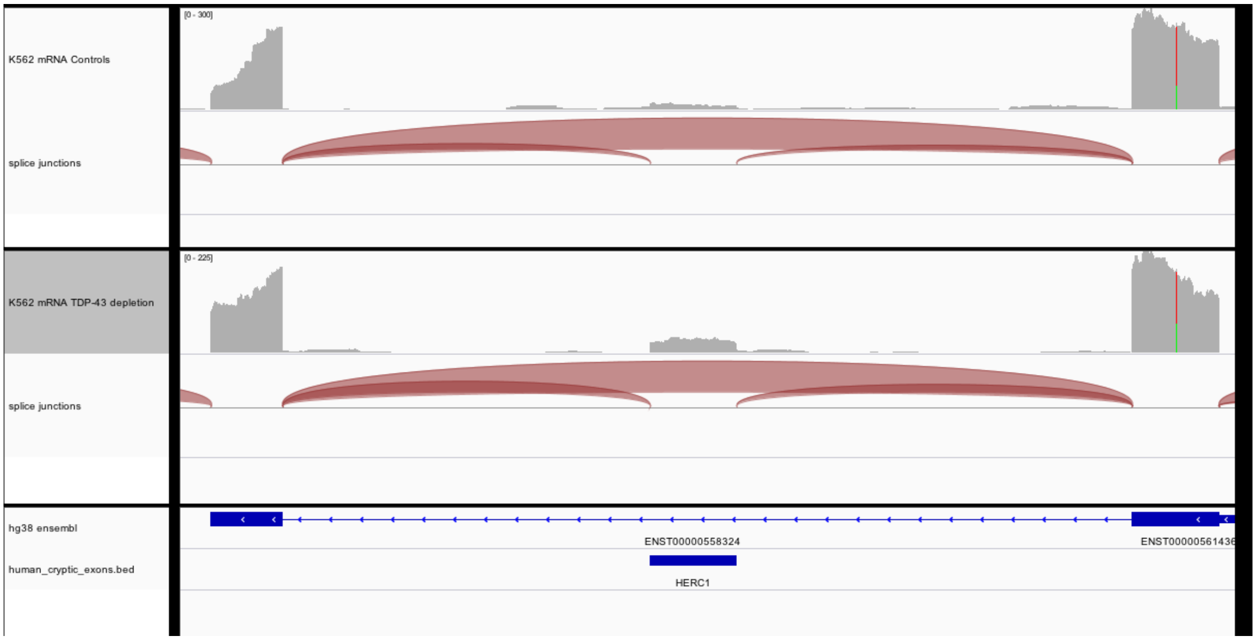

Human K562 total RNA

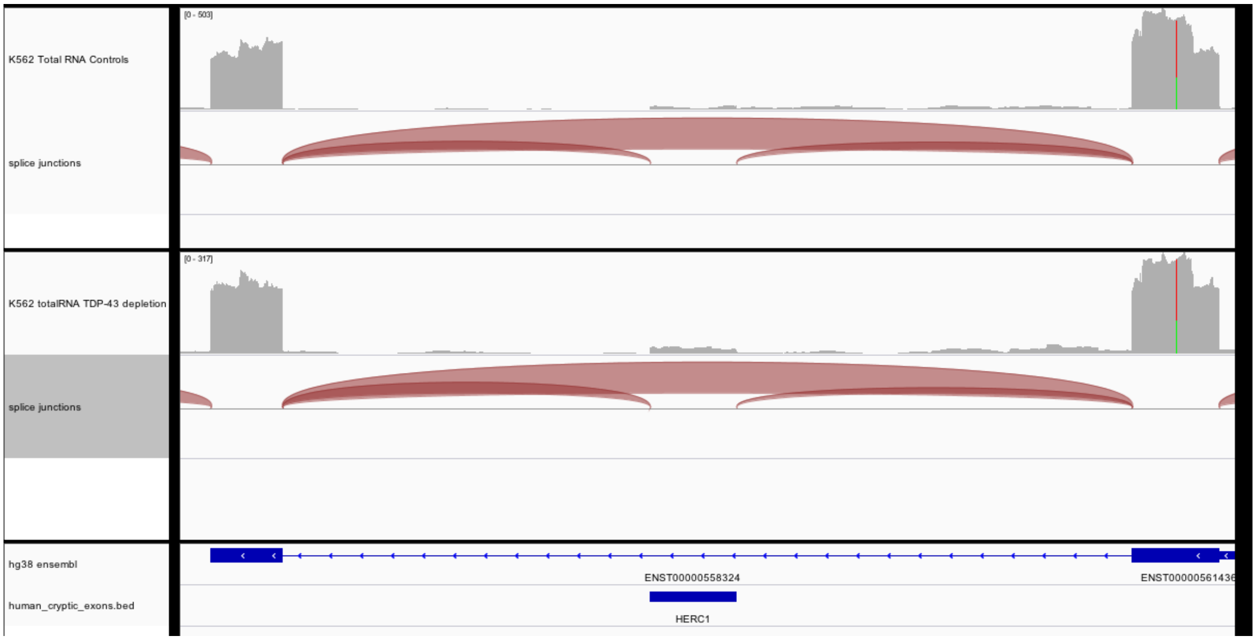

71 SP3 E011i1

Human K562 mRNA

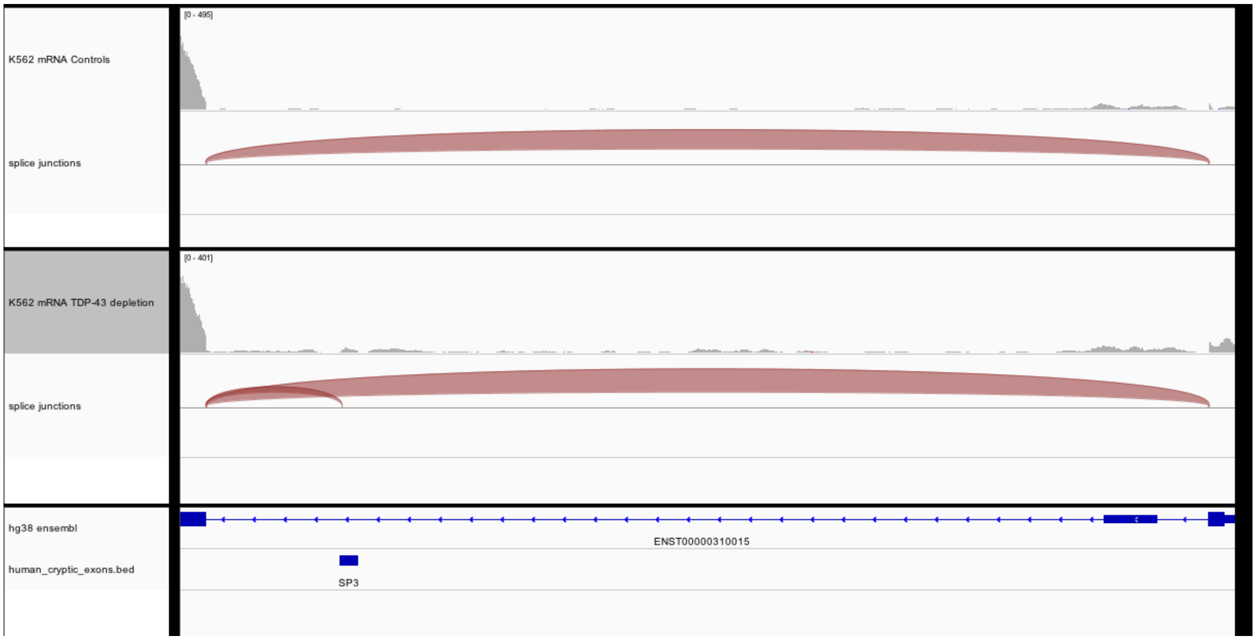

Human K562 total RNA

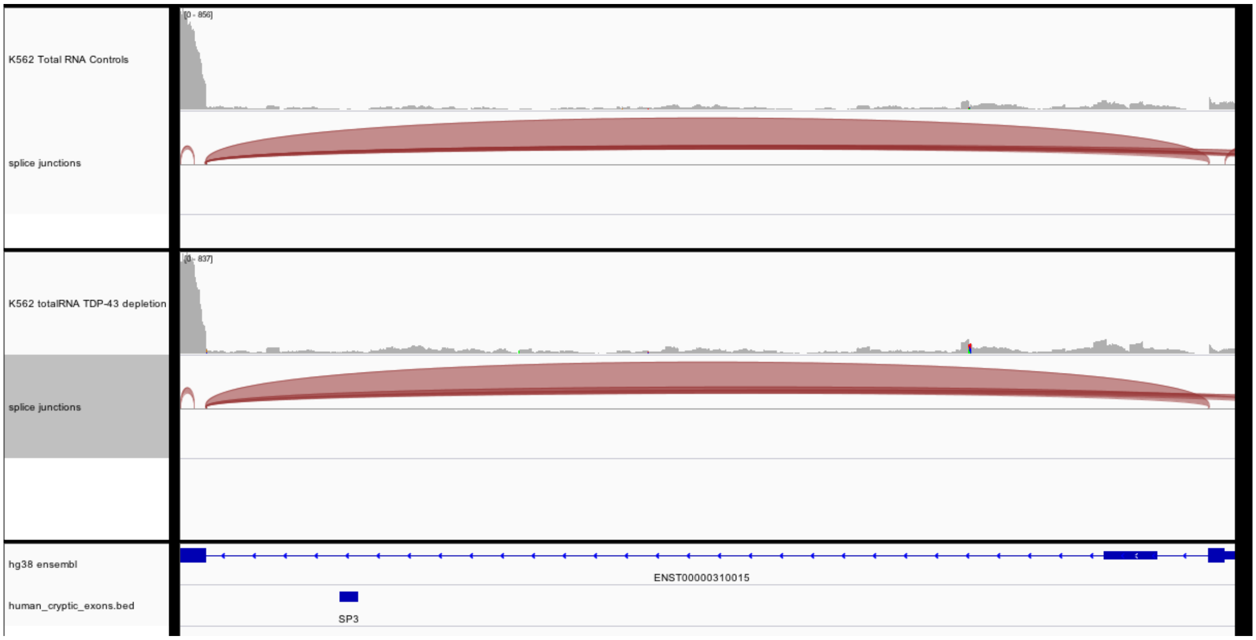

72 DDHD1 E023i1

Human K562 mRNA

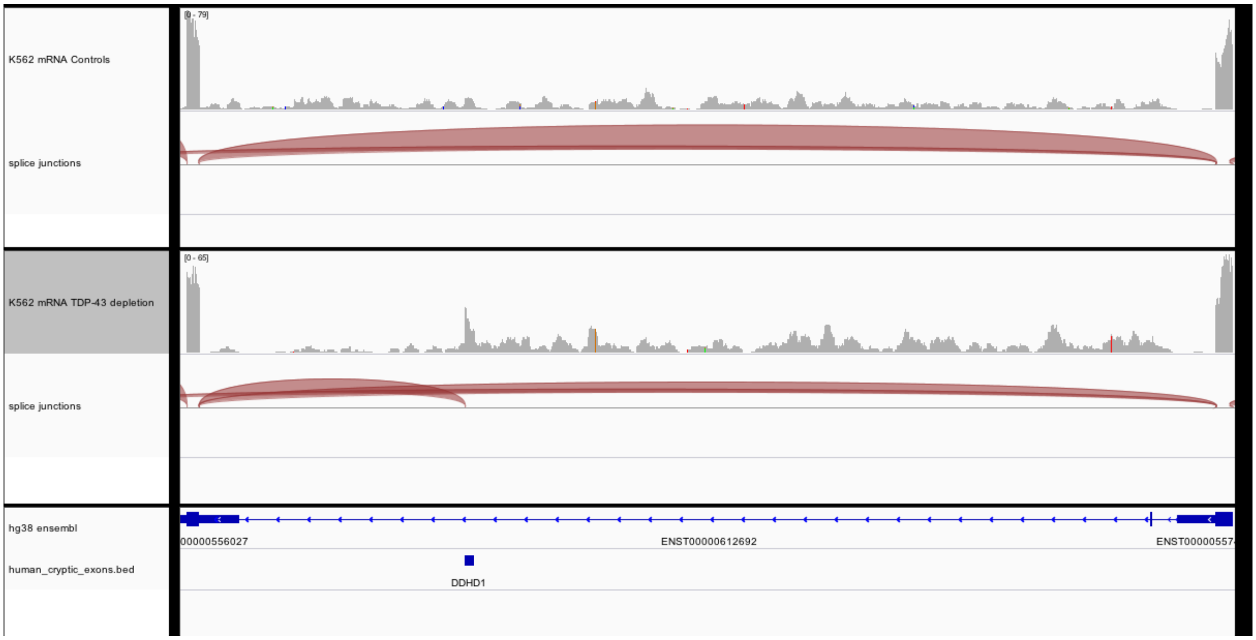

Human K562 total RNA

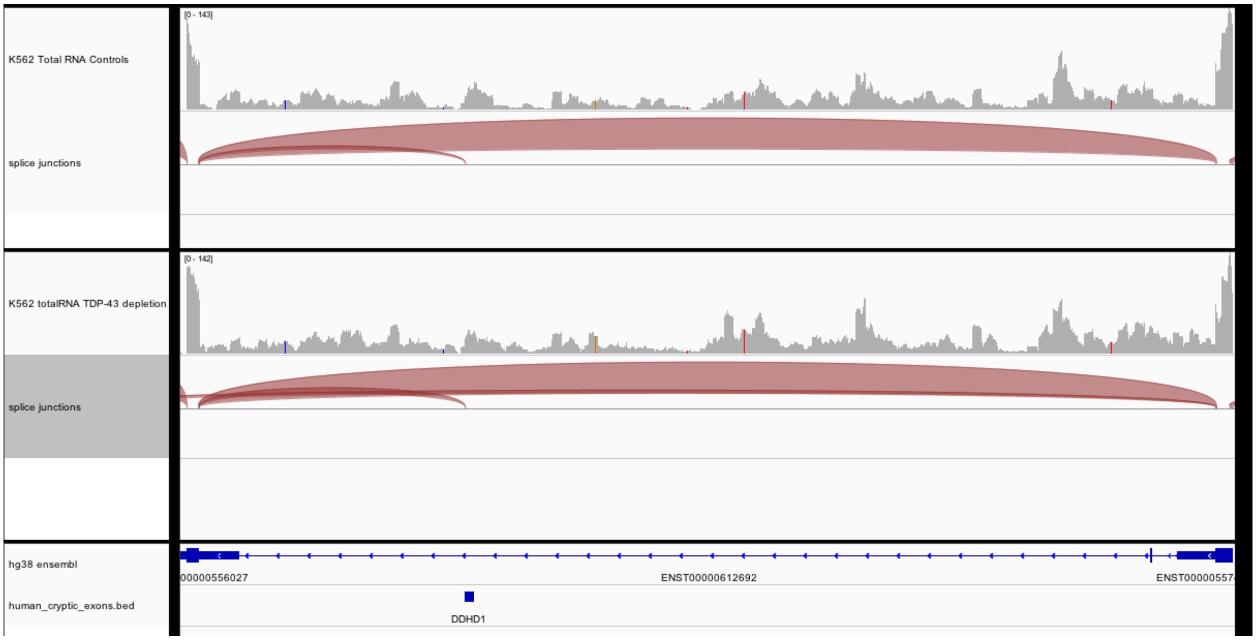

73 RP11-488L18-4 E001i4

Human K562 mRNA

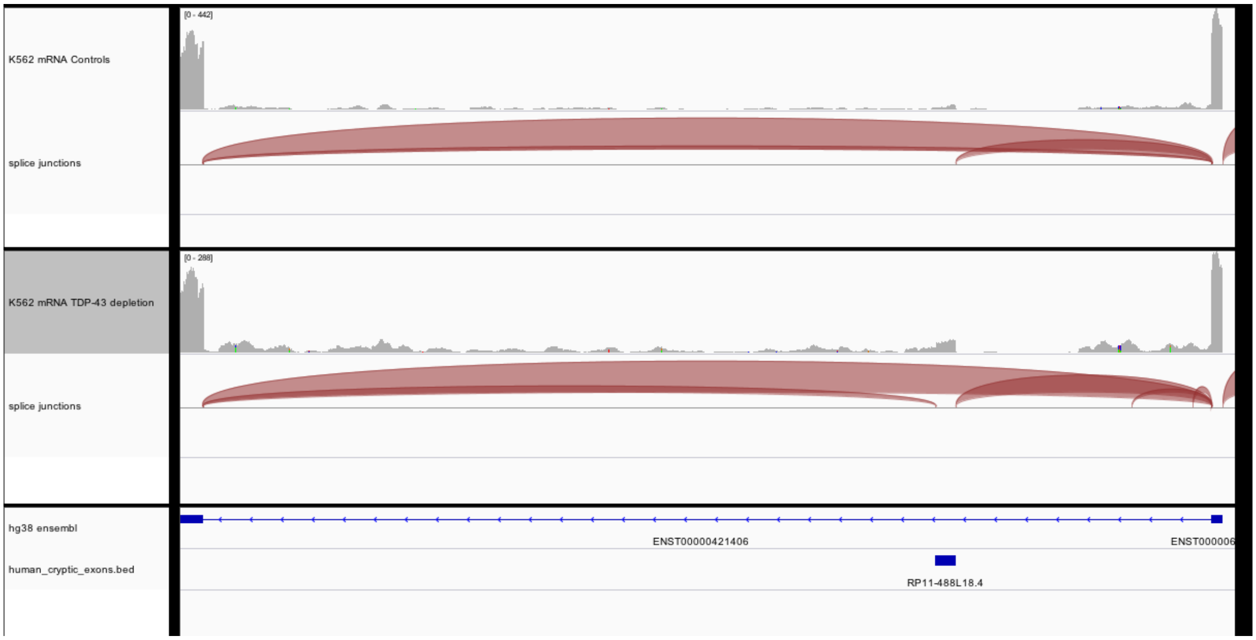

Human K562 total RNA

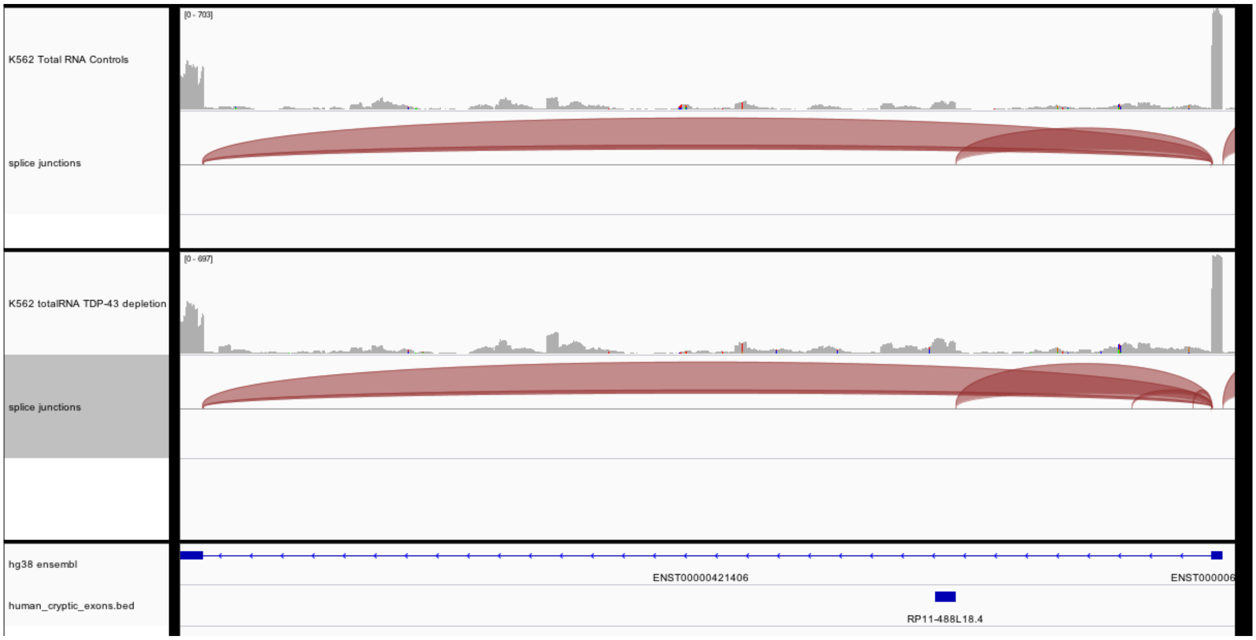

74 UHRF2 E017i2

Human K562 mRNA

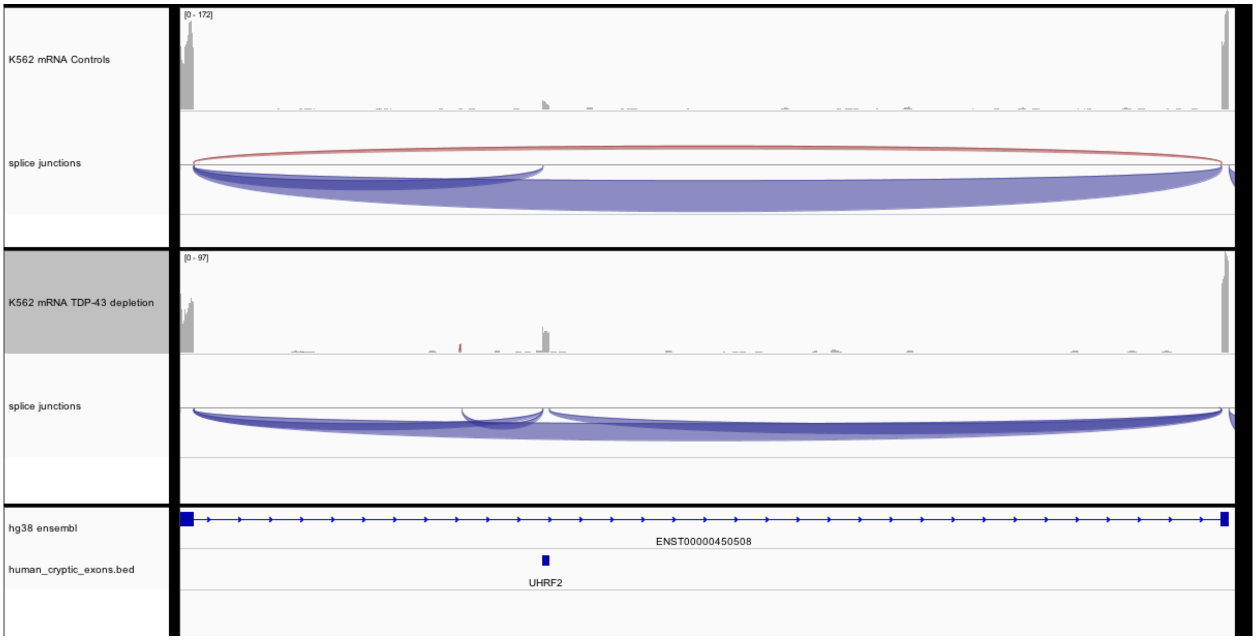

Human K562 total RNA

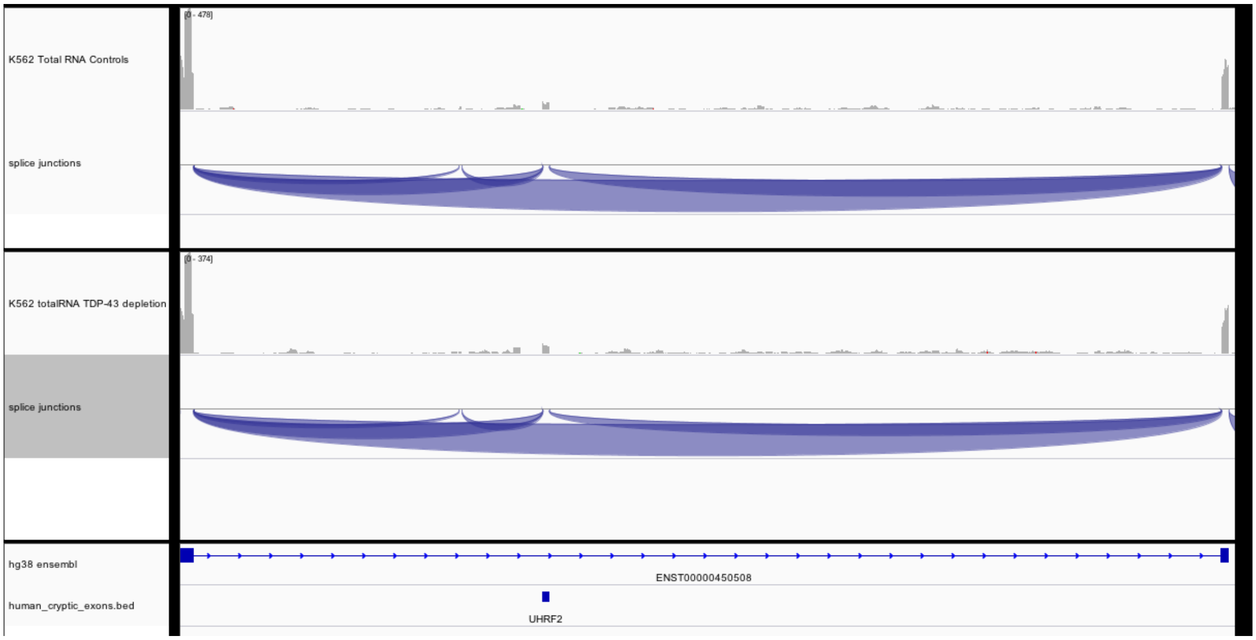

75 REV3L E028i1

Human K562 mRNA

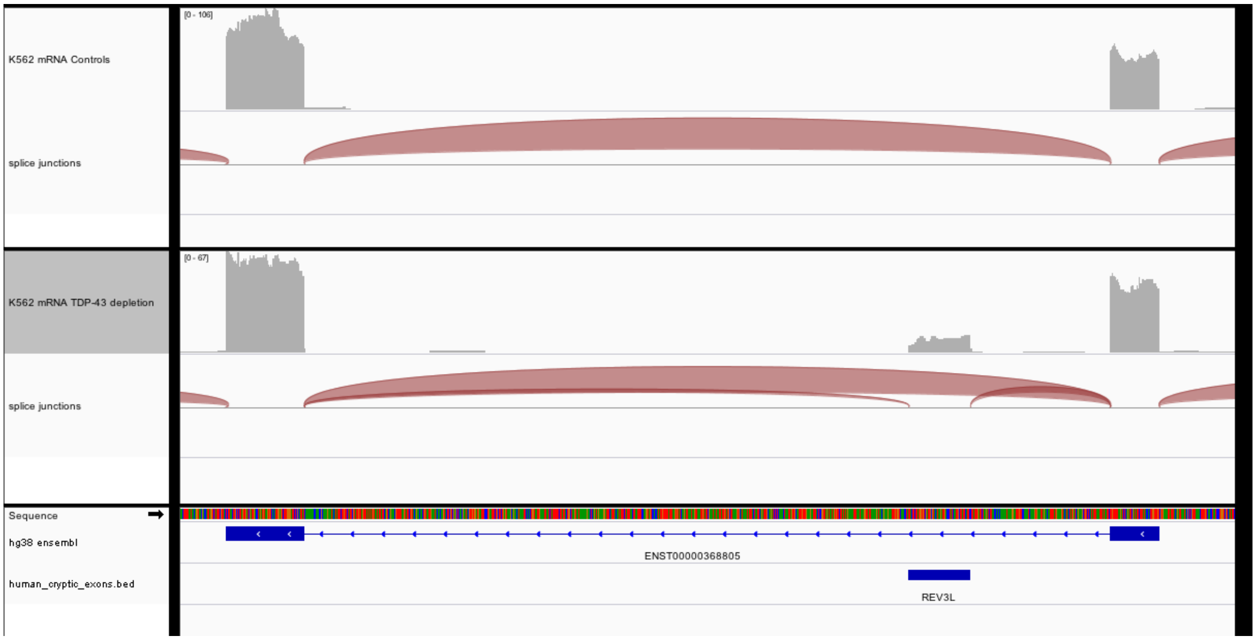

Human K562 total RNA

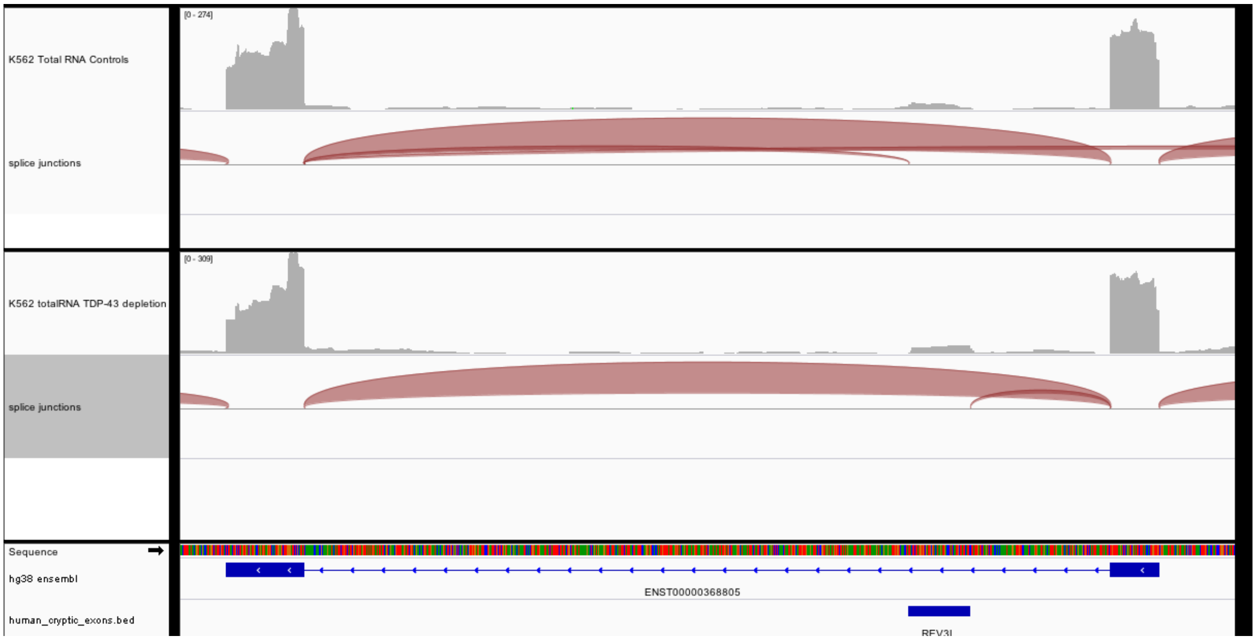

76 SLC12A2 E012i1

Human K562 mRNA

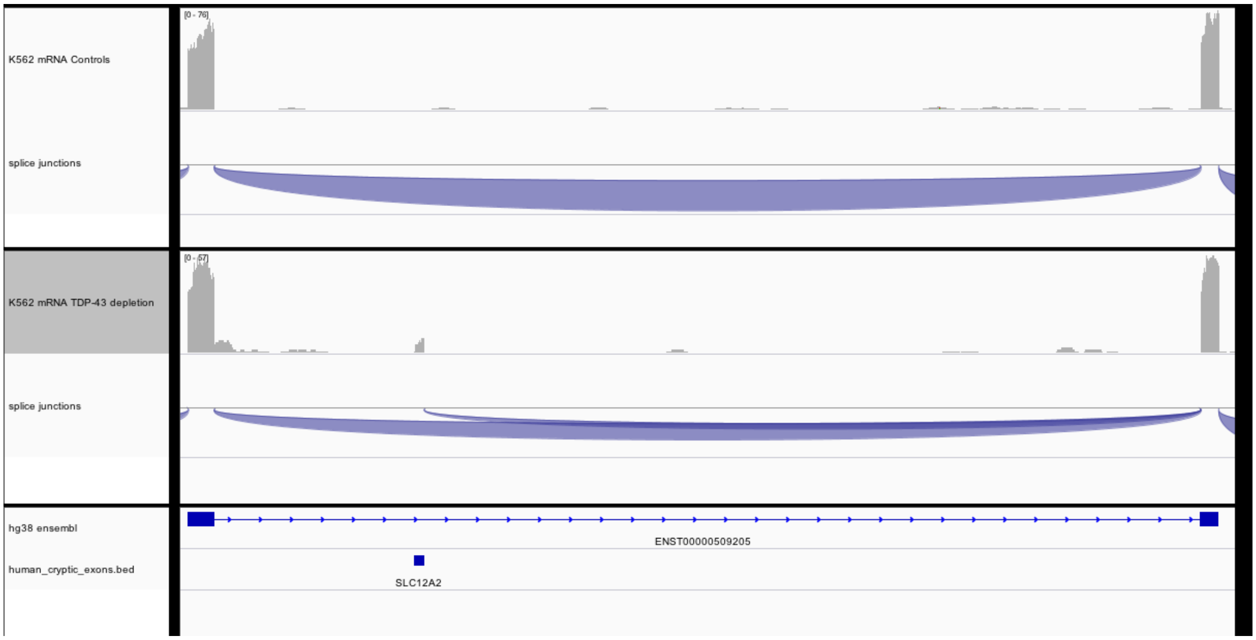

Human K562 total RNA

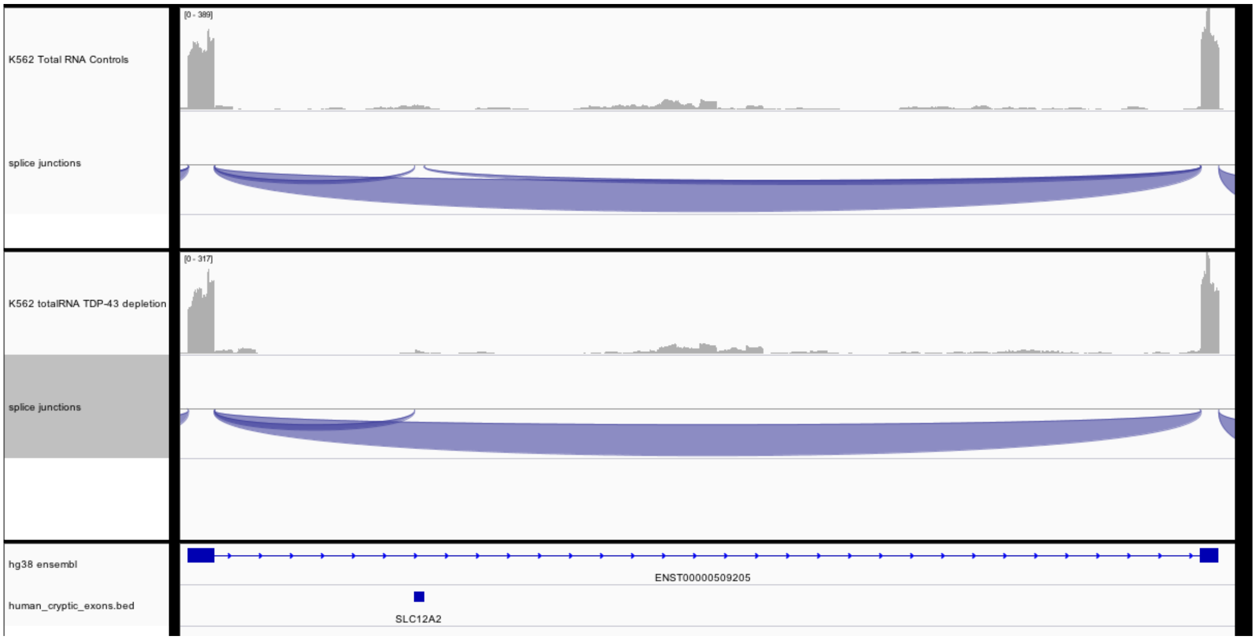

77 MED23 E025i1

Human K562 mRNA

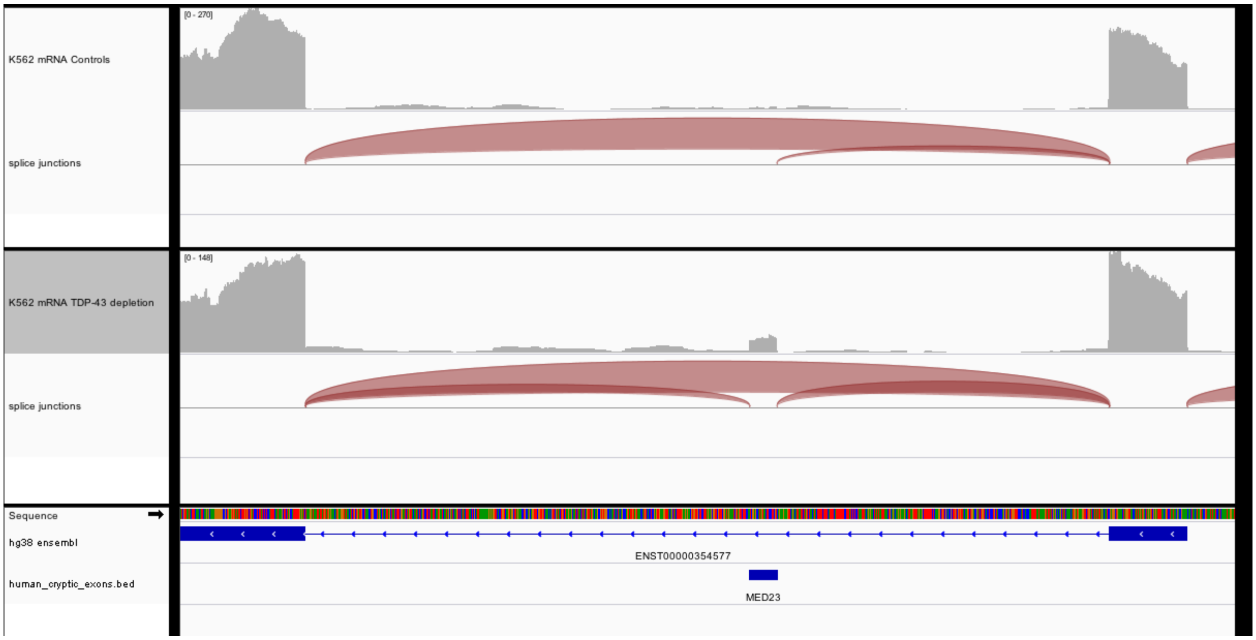

Human K562 total RNA

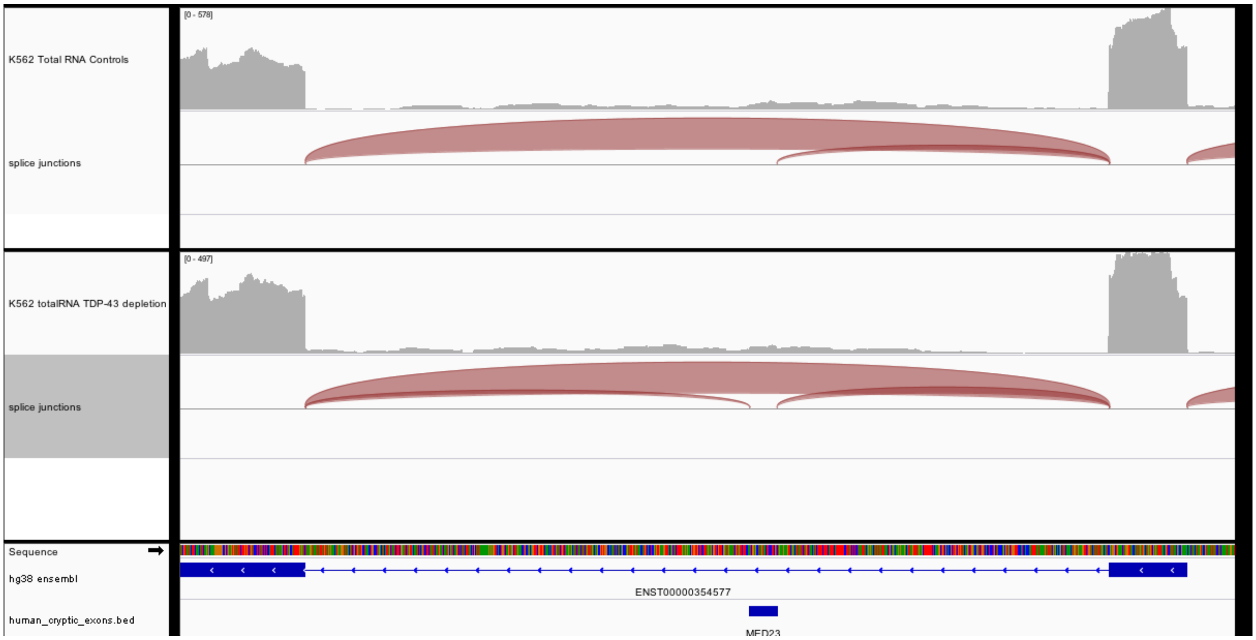

78 POLK E019i1

Human K562 mRNA

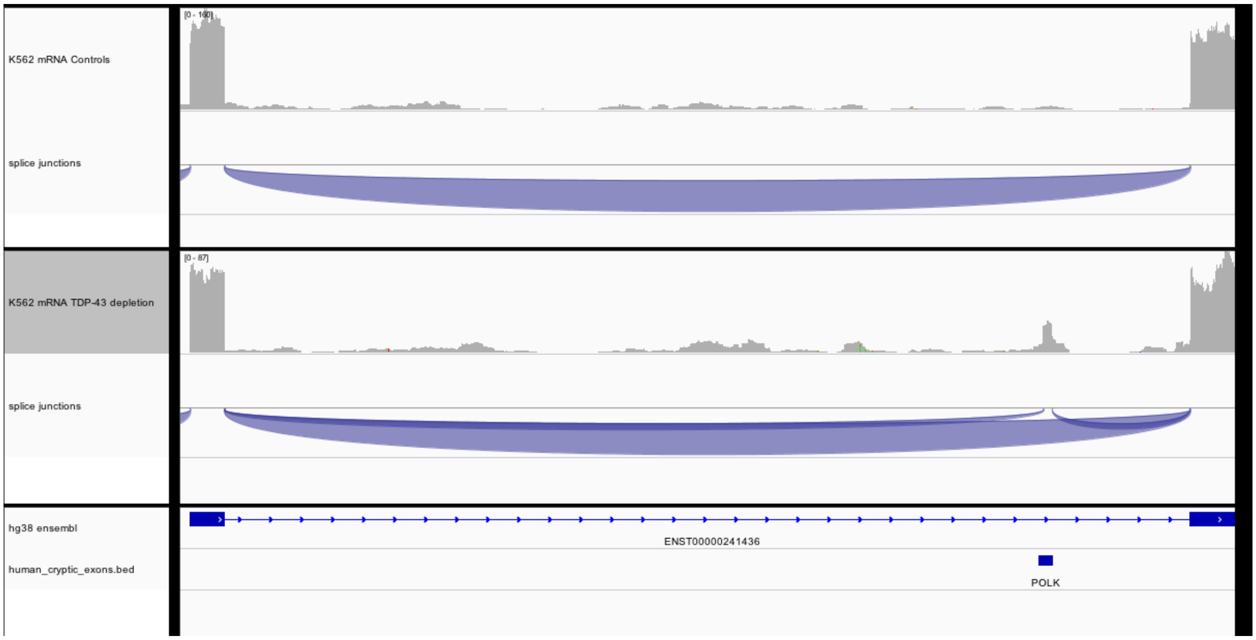

Human K562 total RNA

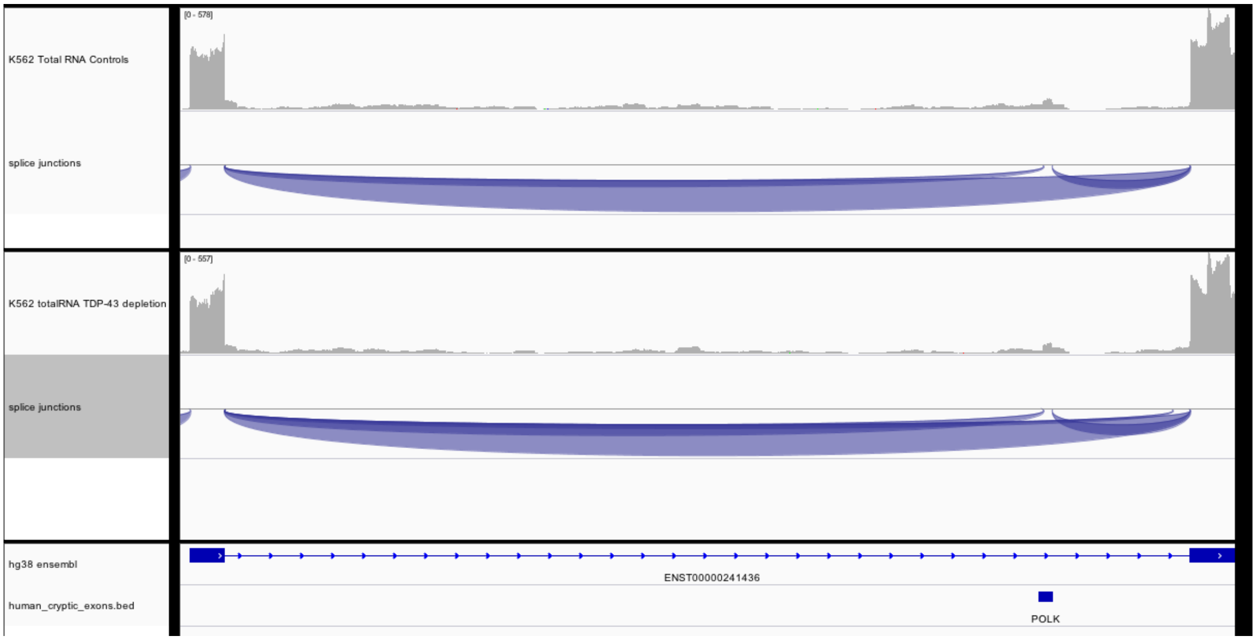

79 TRPM7 E027i1

Human K562 mRNA

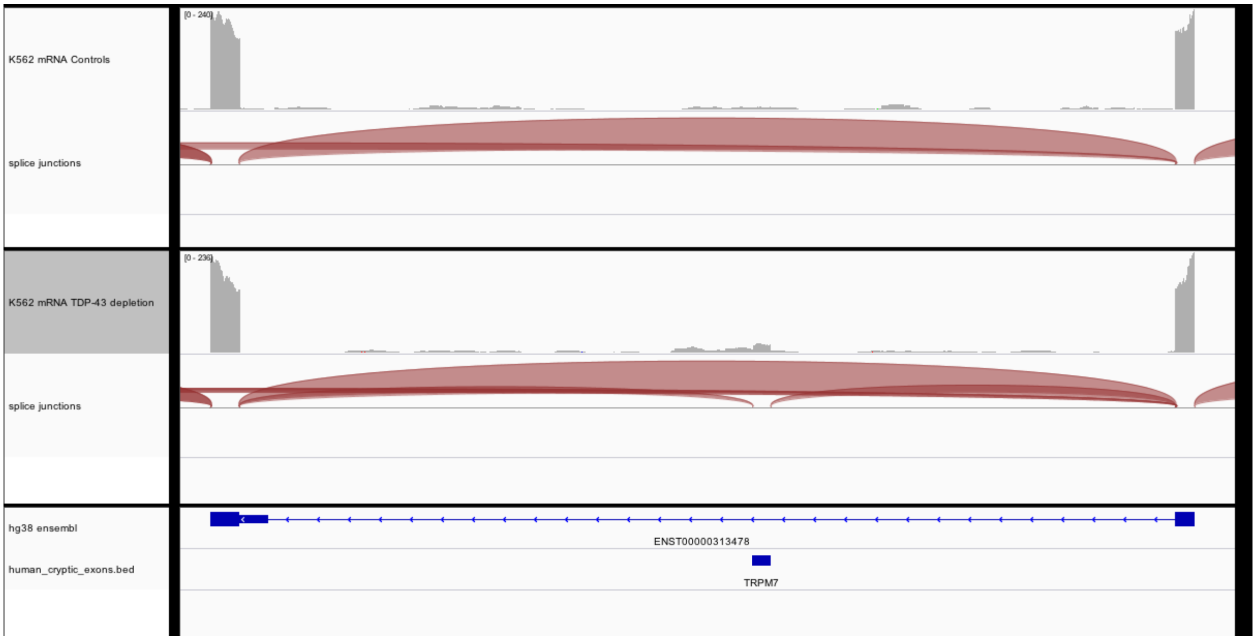

Human K562 total RNA

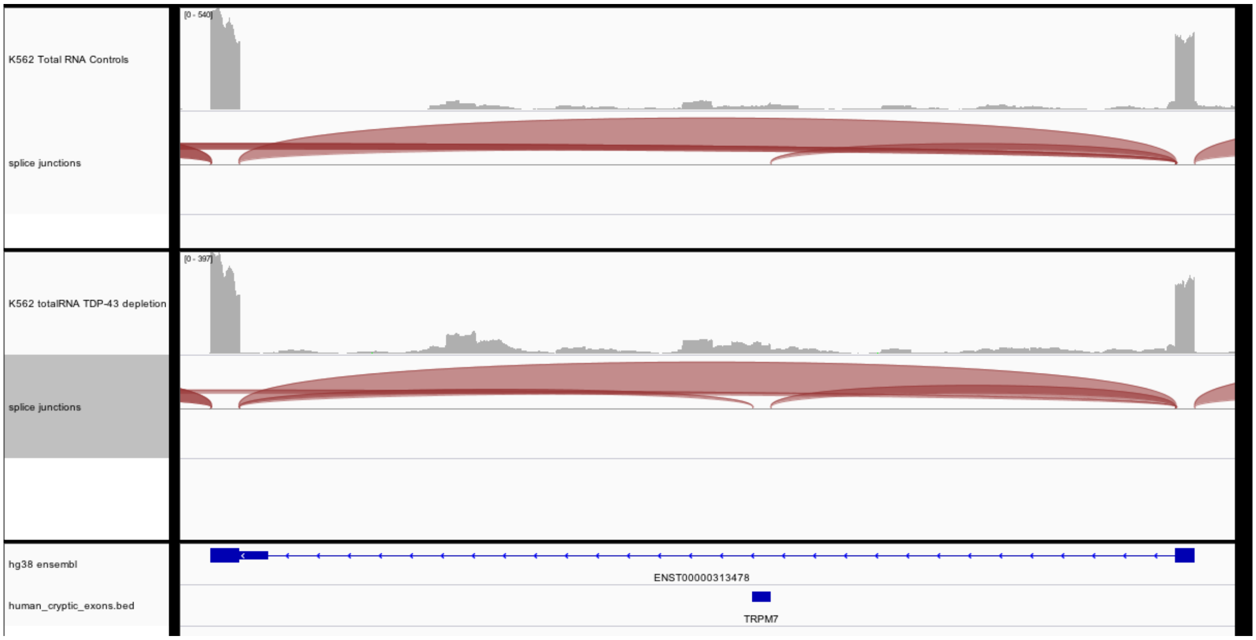

80 TRIM37 E029i1

Human K562 mRNA

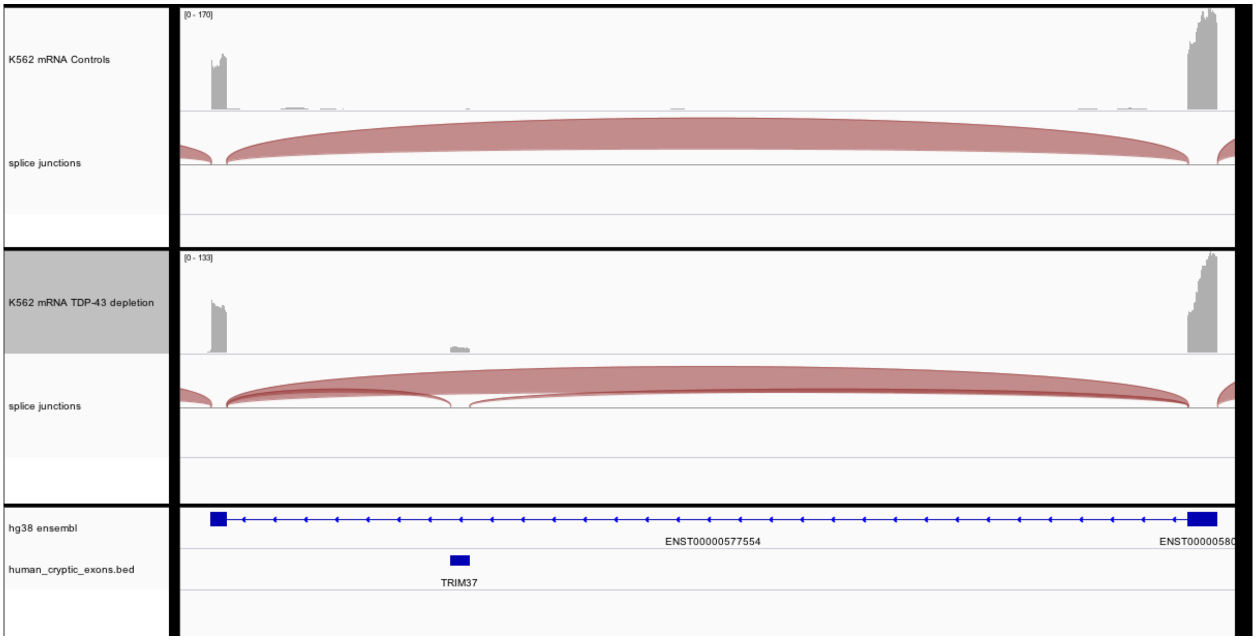

Human K562 total RNA

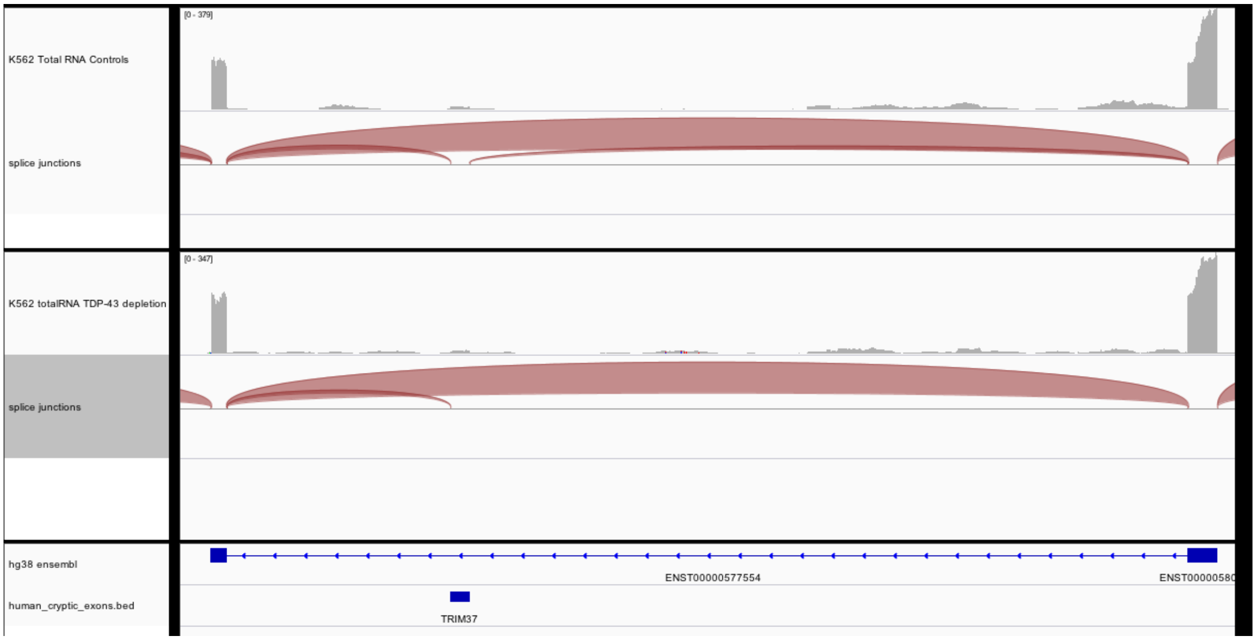

81 C4orf32 E001i1

Human K562 mRNA

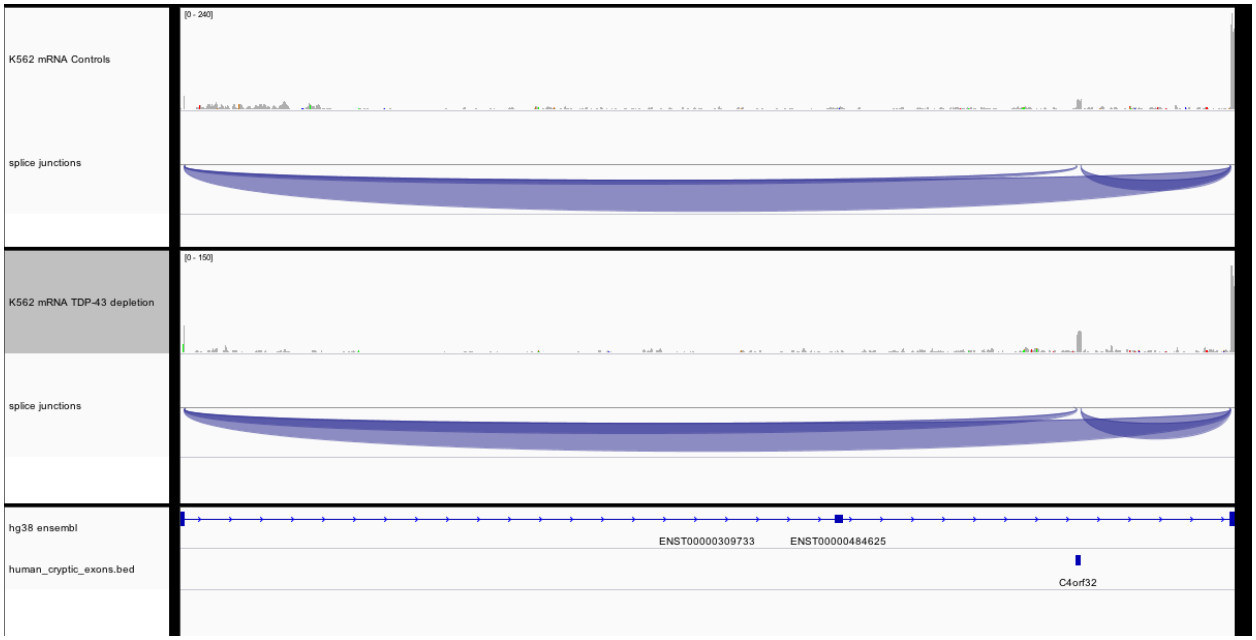

Human K562 total RNA

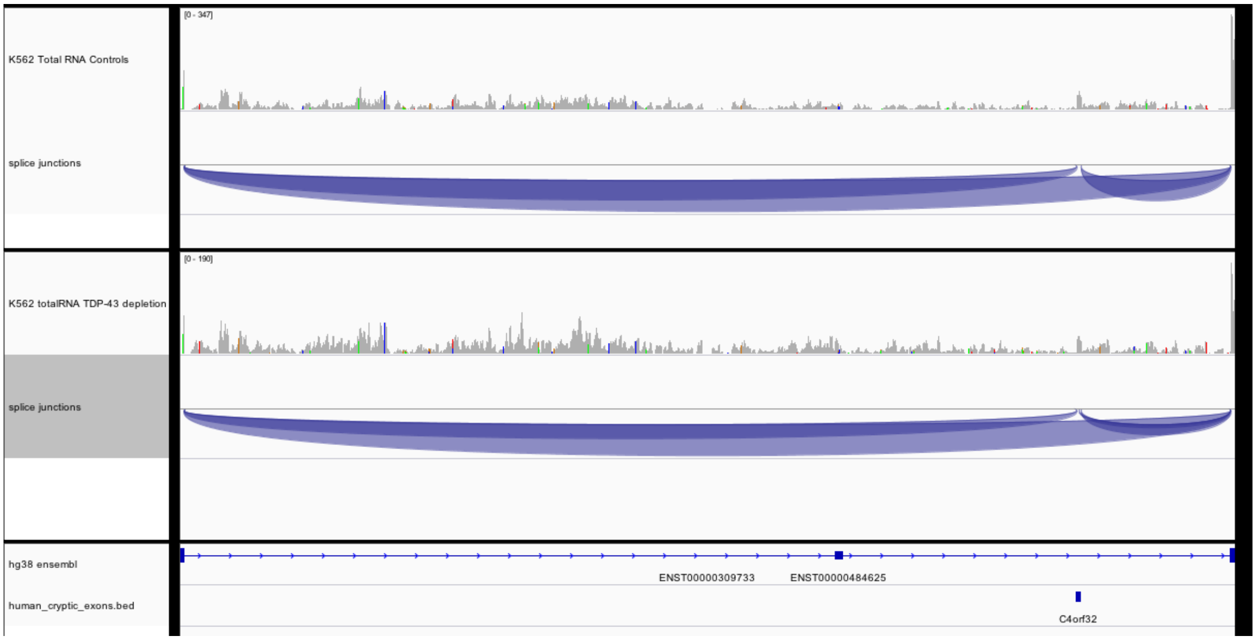

82 ZCCHC6 E004i1

Human K562 mRNA

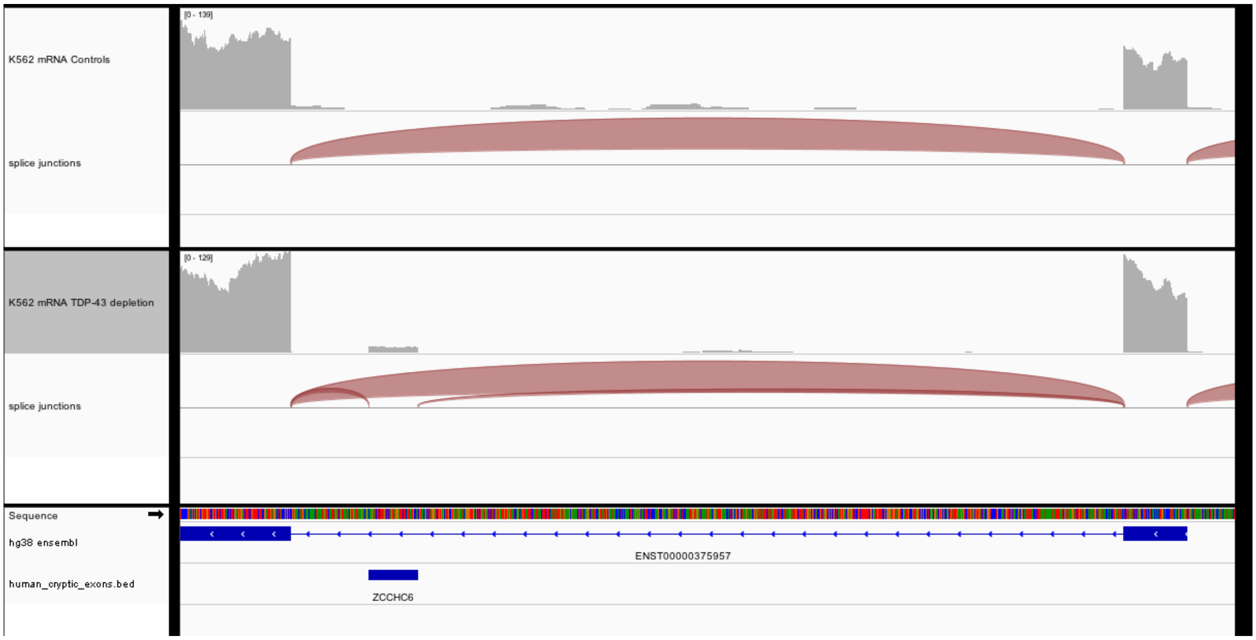

Human K562 total RNA

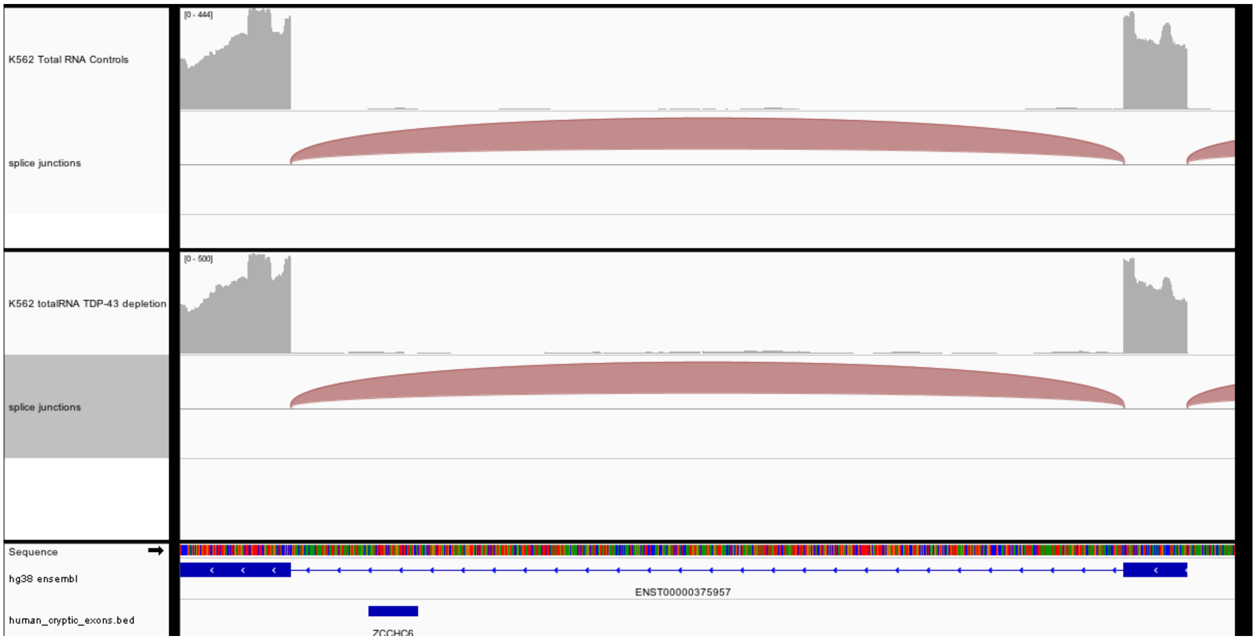

83 ANKRD26 E045i1

Human K562 mRNA

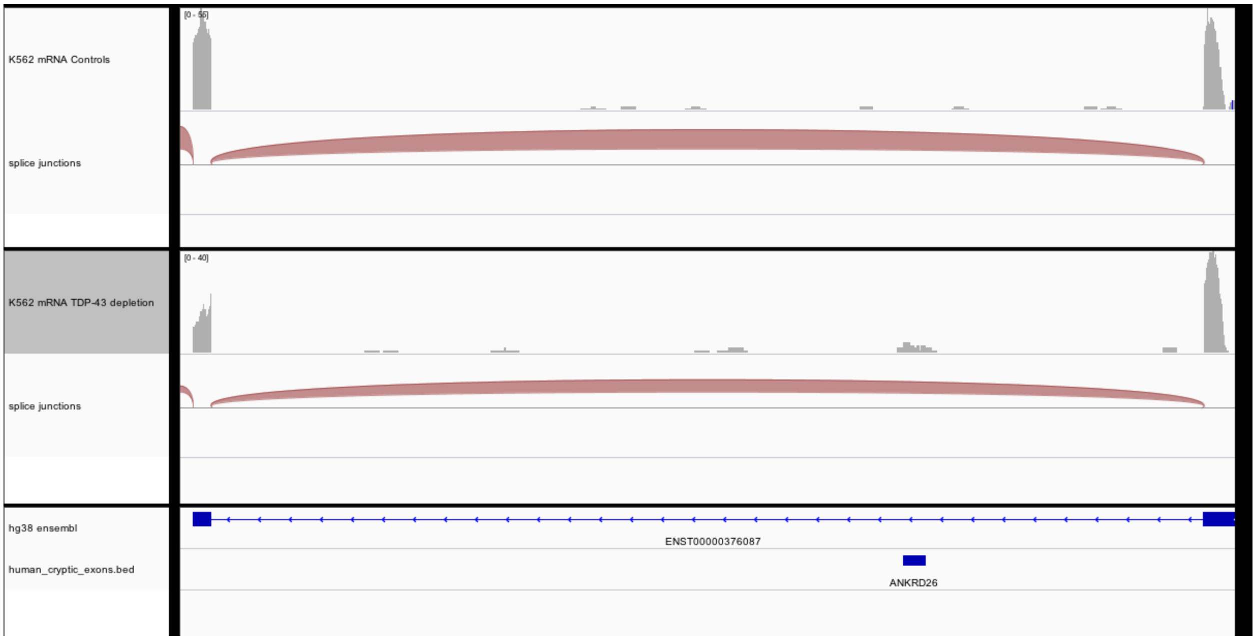

Human K562 total RNA

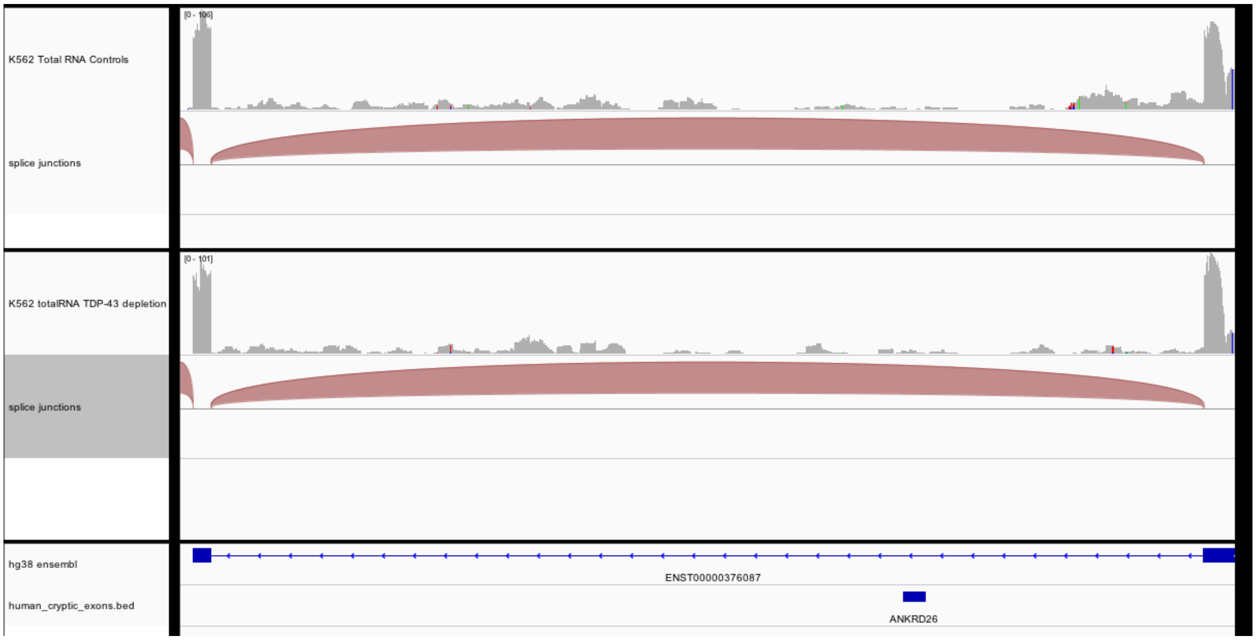

84 KIAA1429 E039i3

Human K562 mRNA

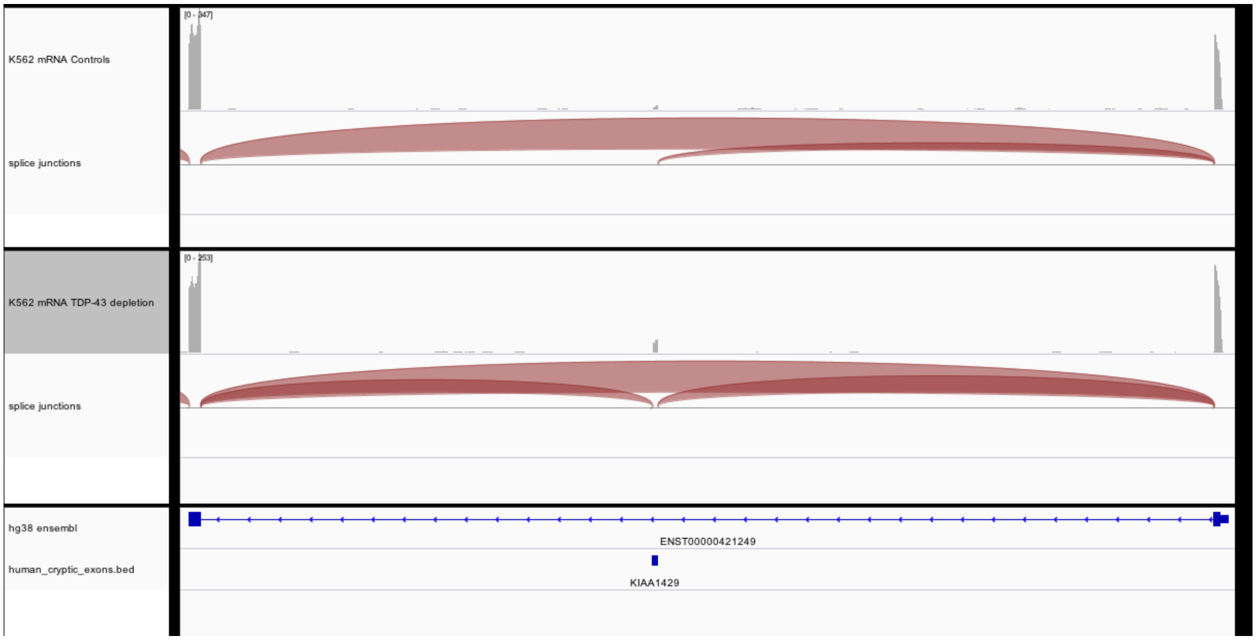

Human K562 total RNA

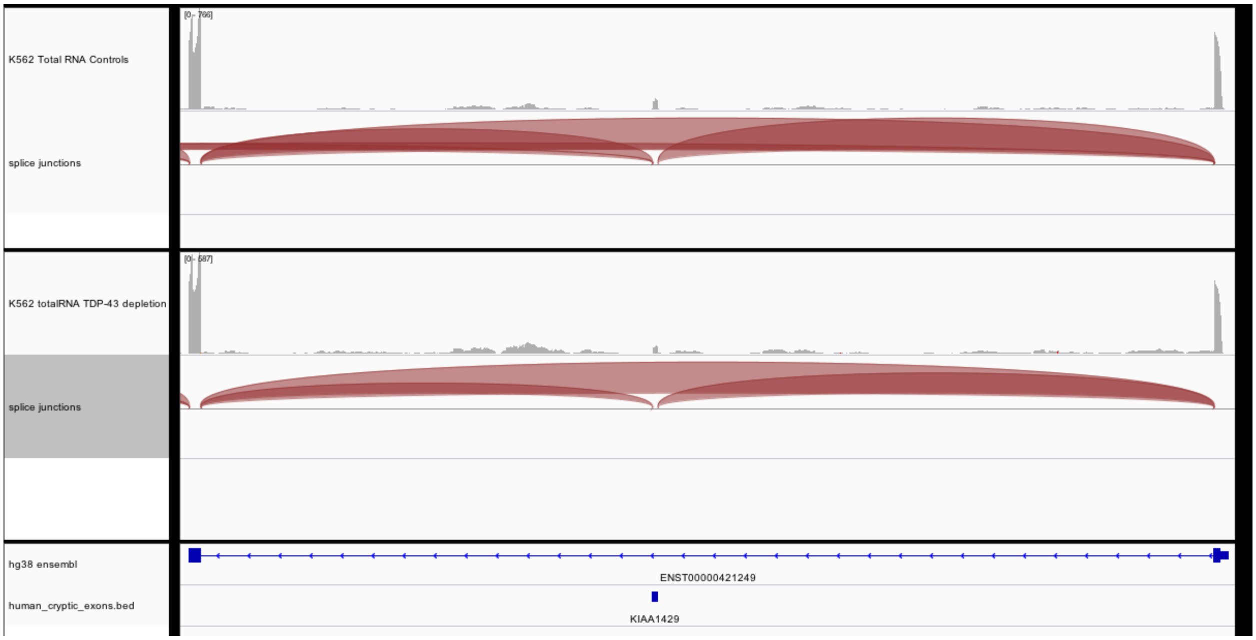

85 ZFYVE27 E020i1

Human K562 mRNA

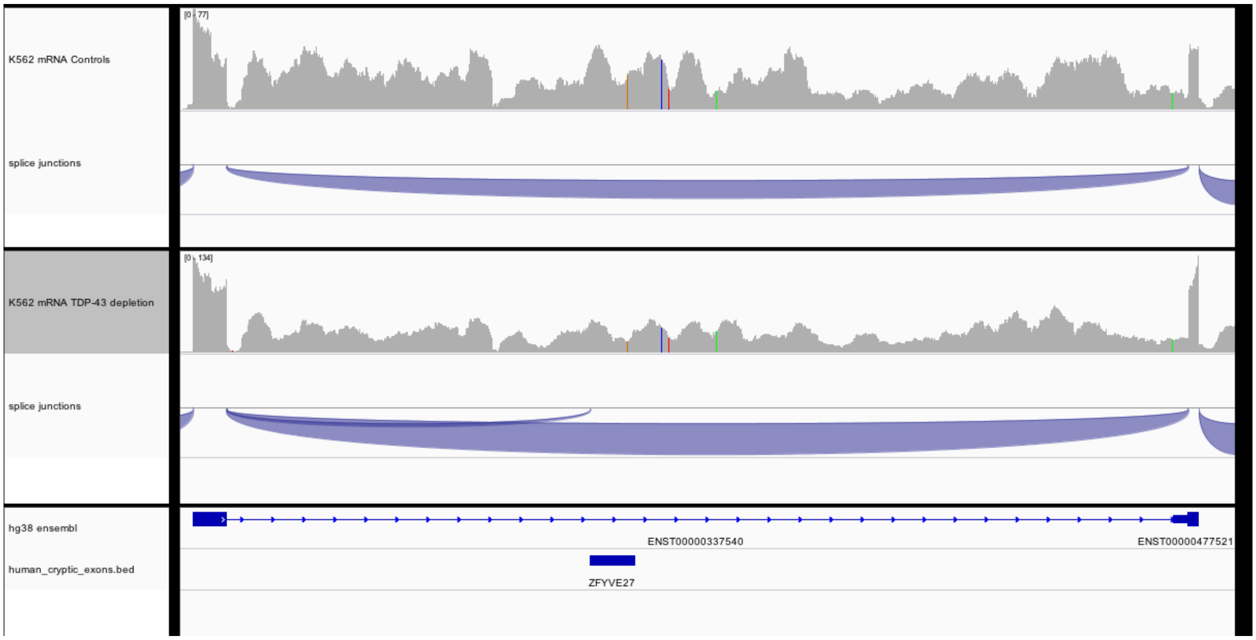

Human K562 total RNA

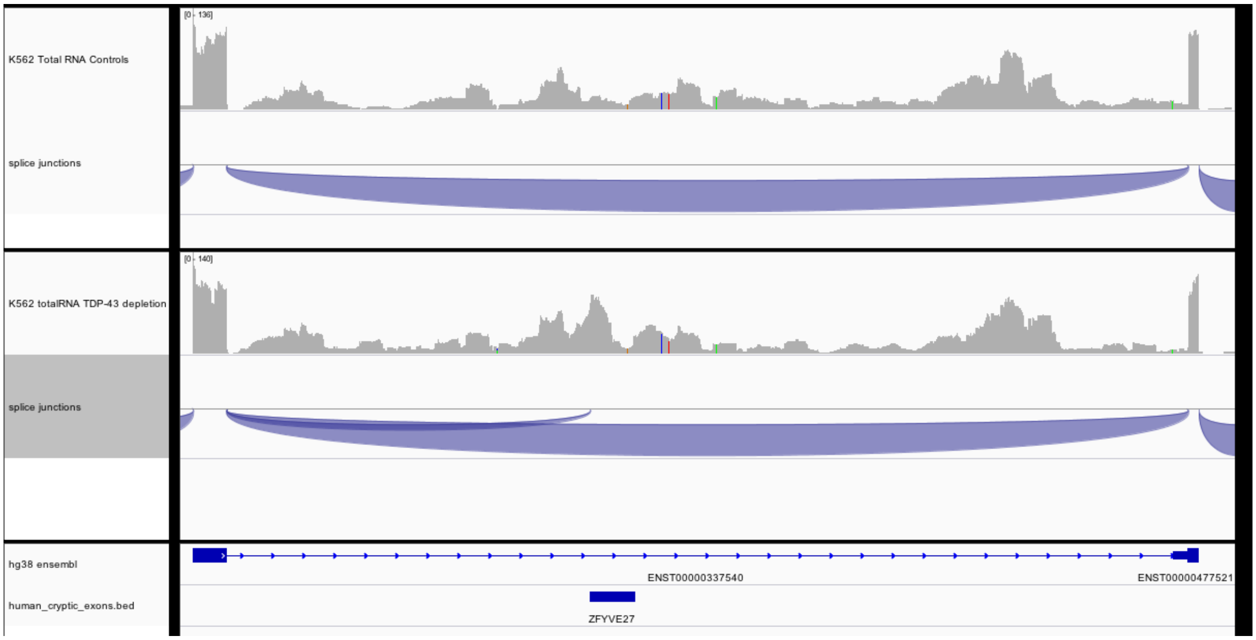

86 MYO16 E036i1

Human K562 mRNA

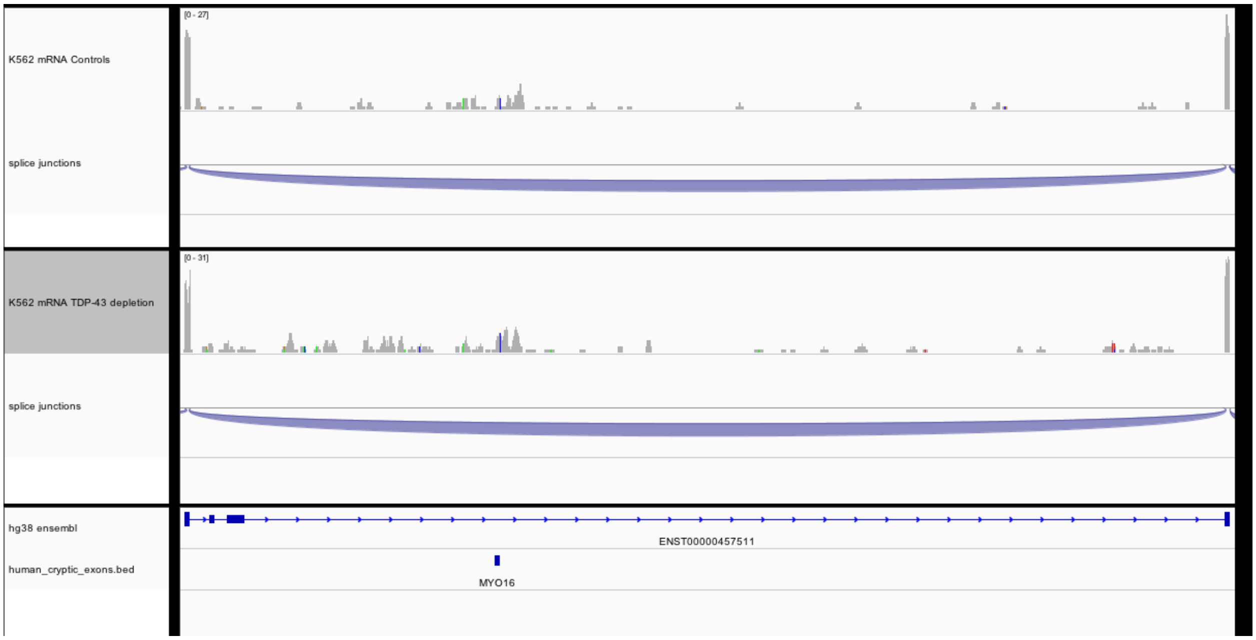

Human K562 total RNA

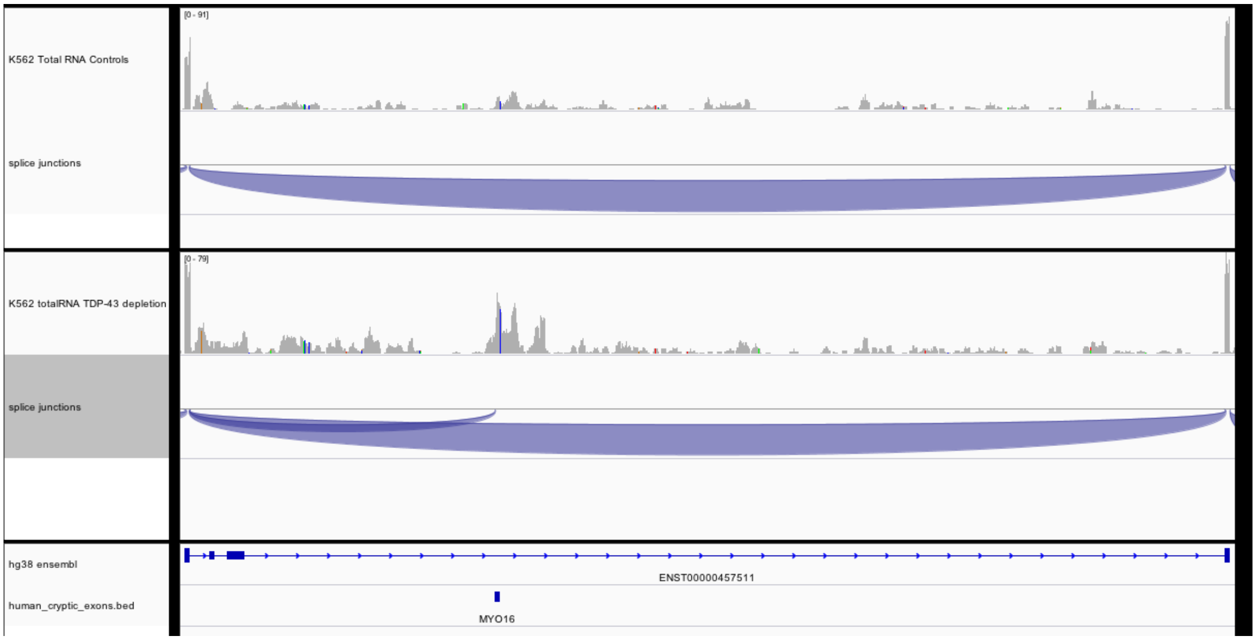

87 INPP5A E009i1

Human K562 mRNA

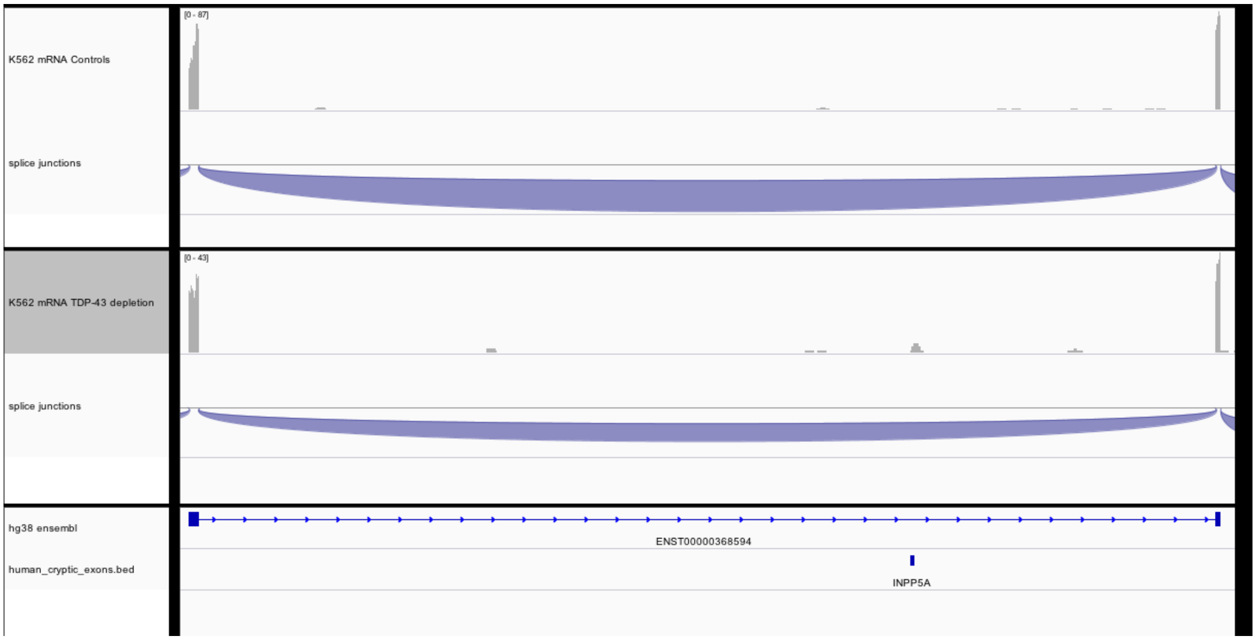

Human K562 total RNA

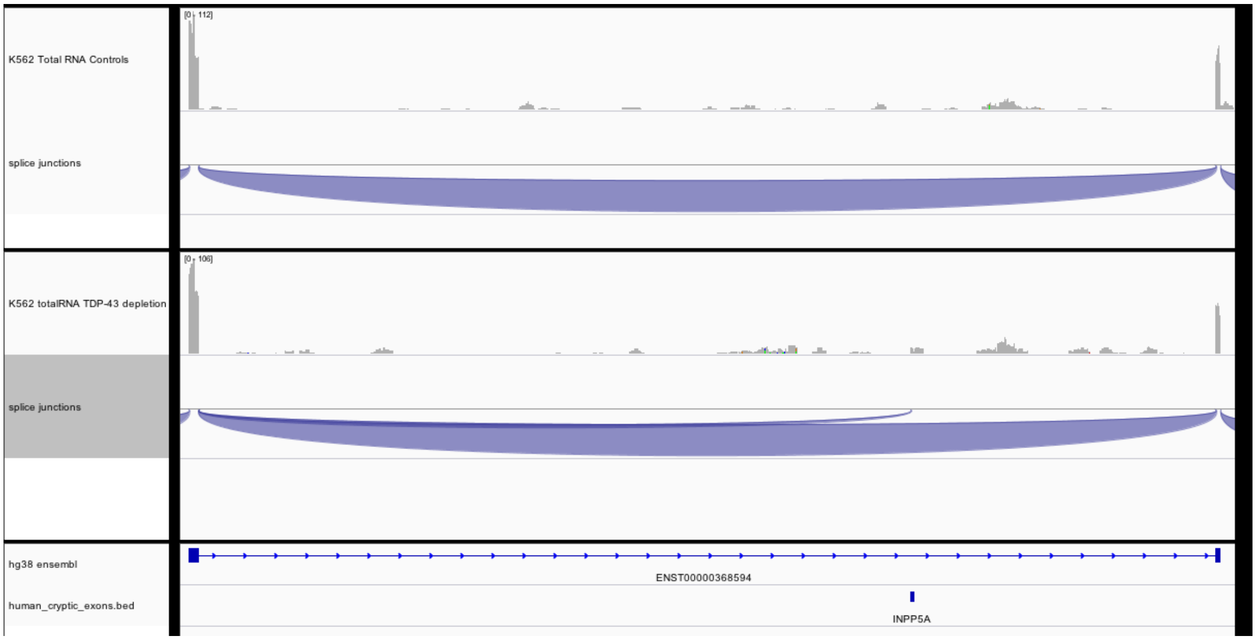

88 SCP2 E024i1

Human K562 mRNA

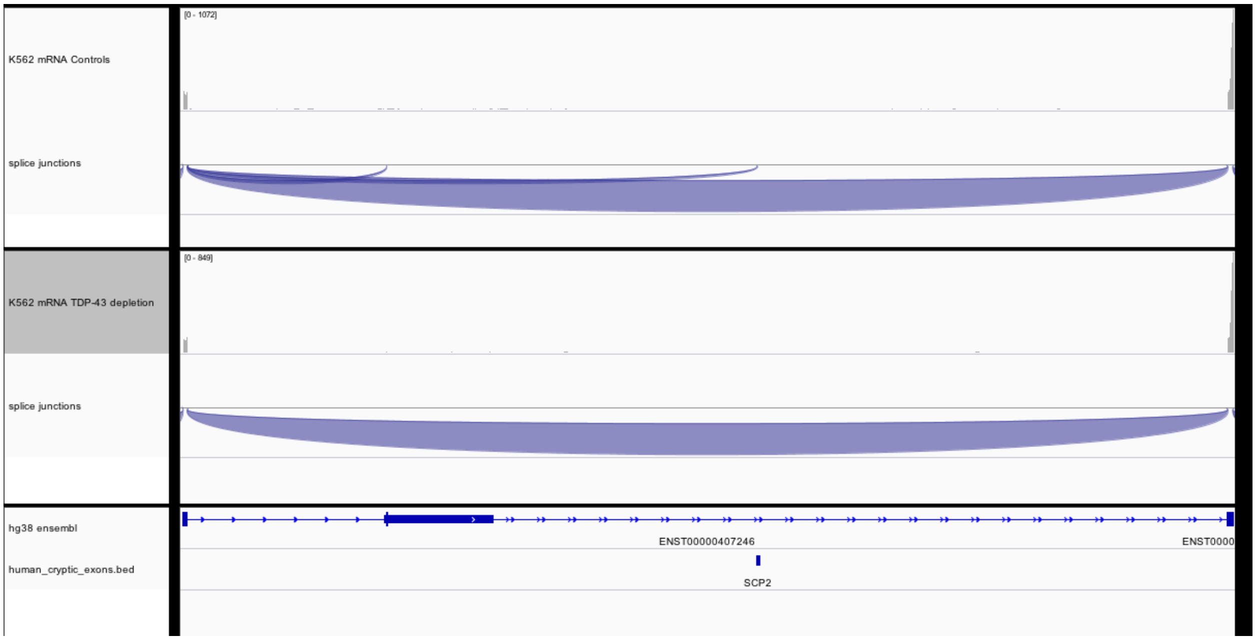

Human K562 total RNA

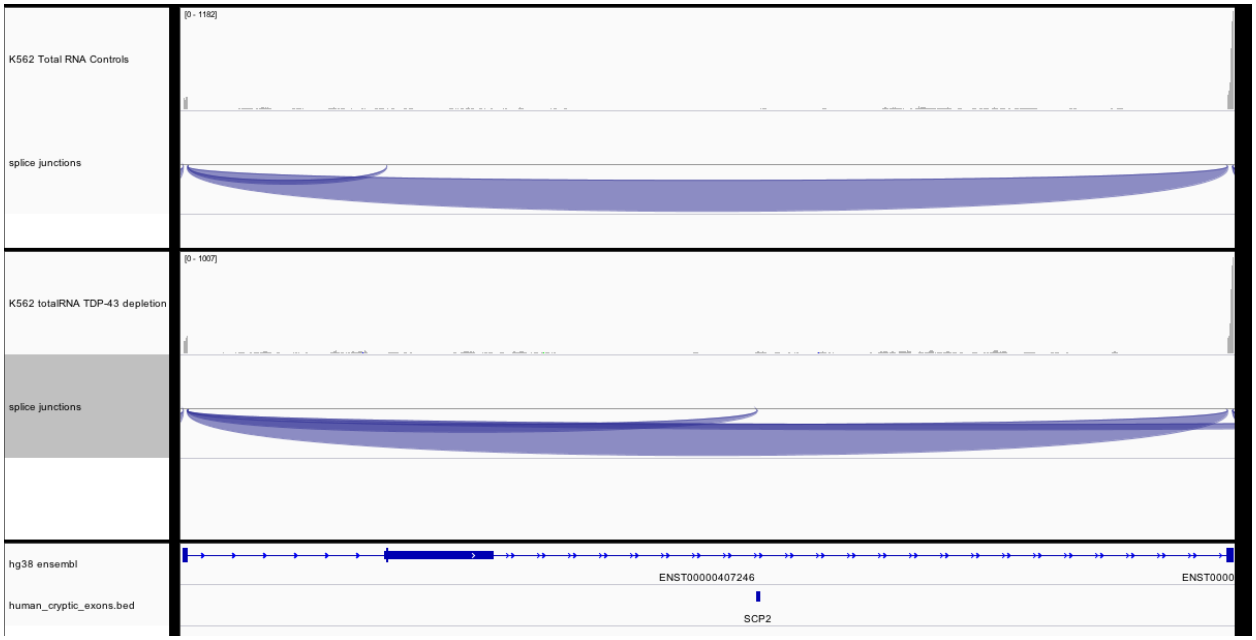

89 DEAF1 E006i2

Human K562 mRNA

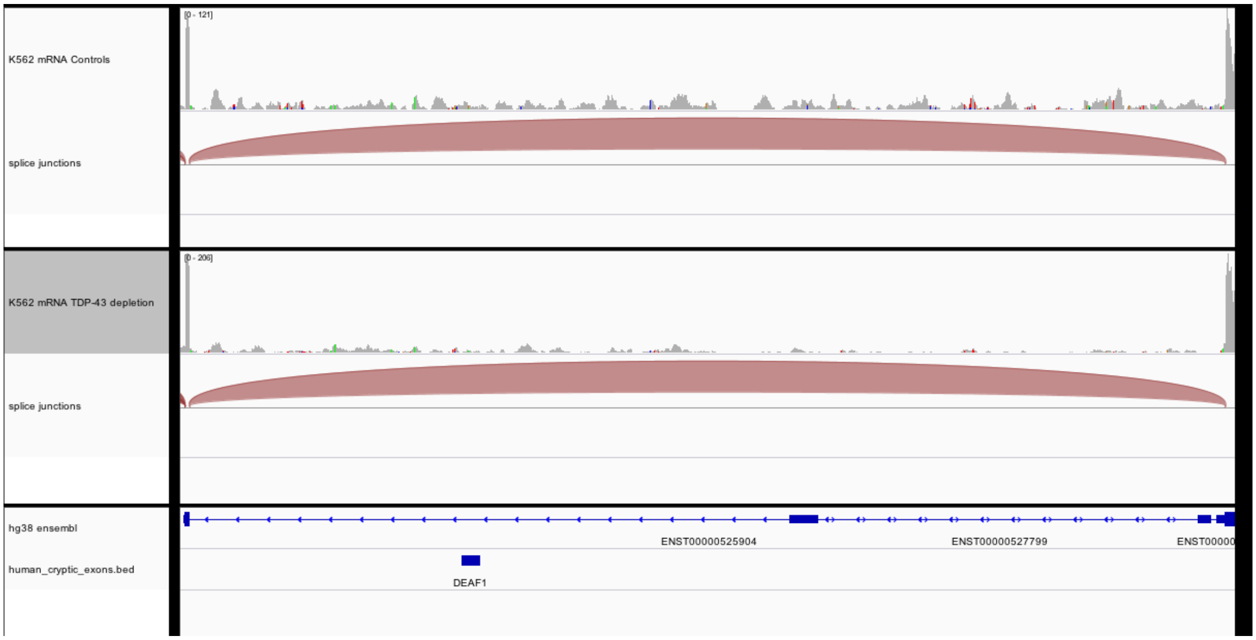

Human K562 total RNA

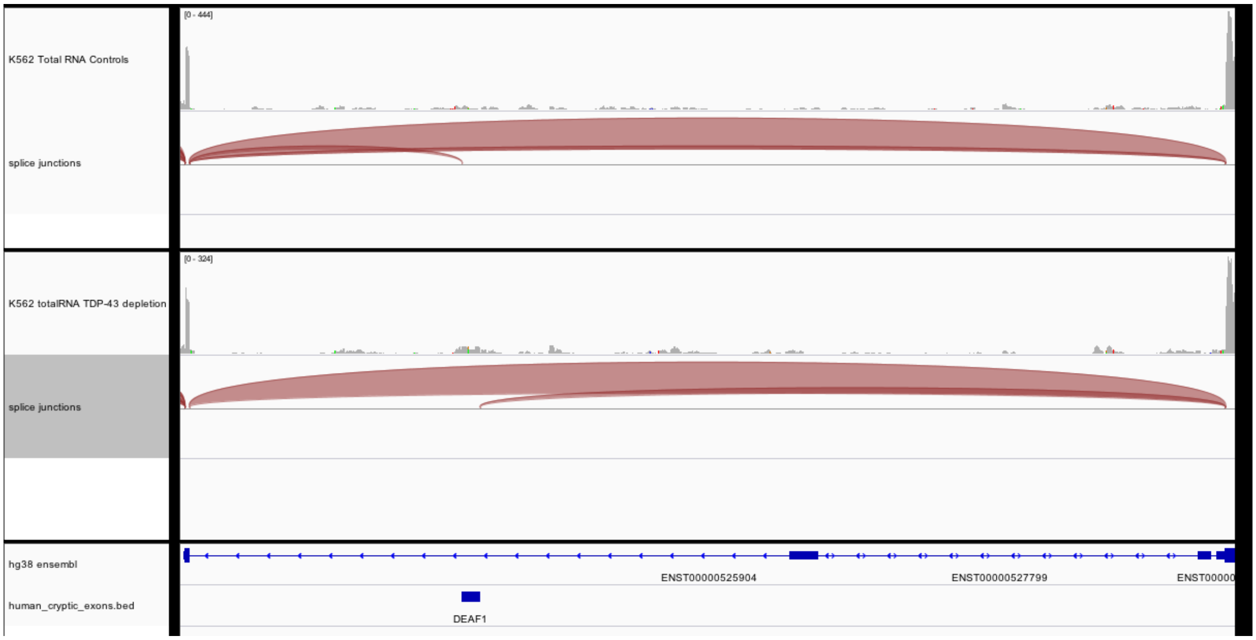

90 0 E012i1

Human K562 mRNA

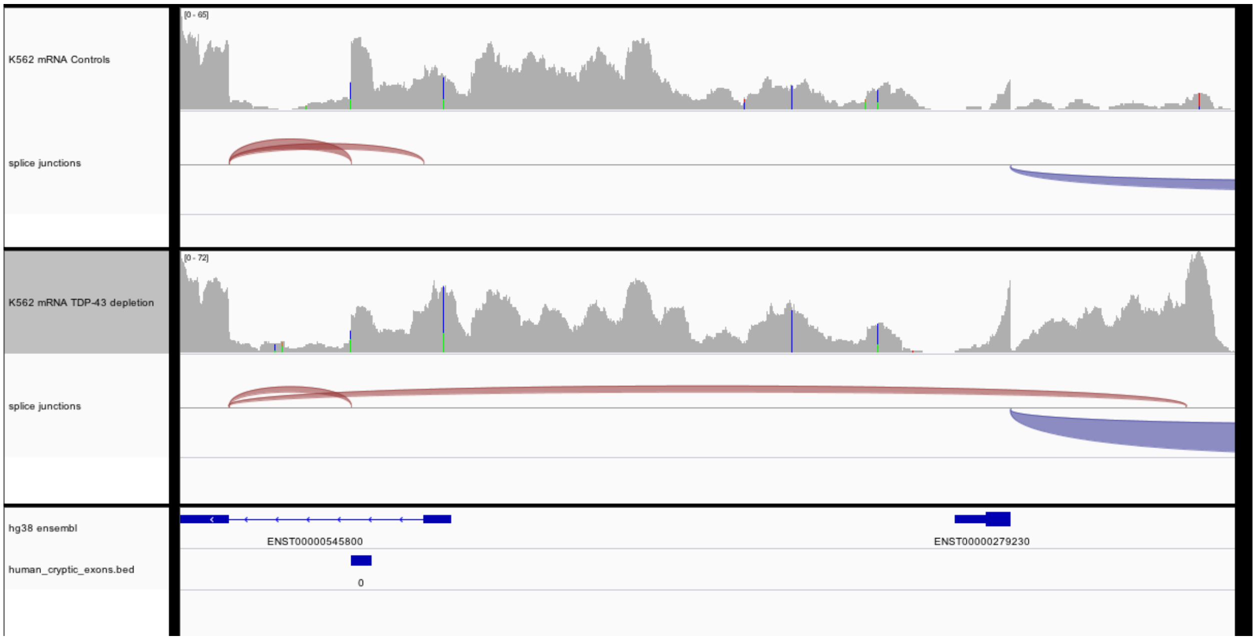

Human K562 total RNA

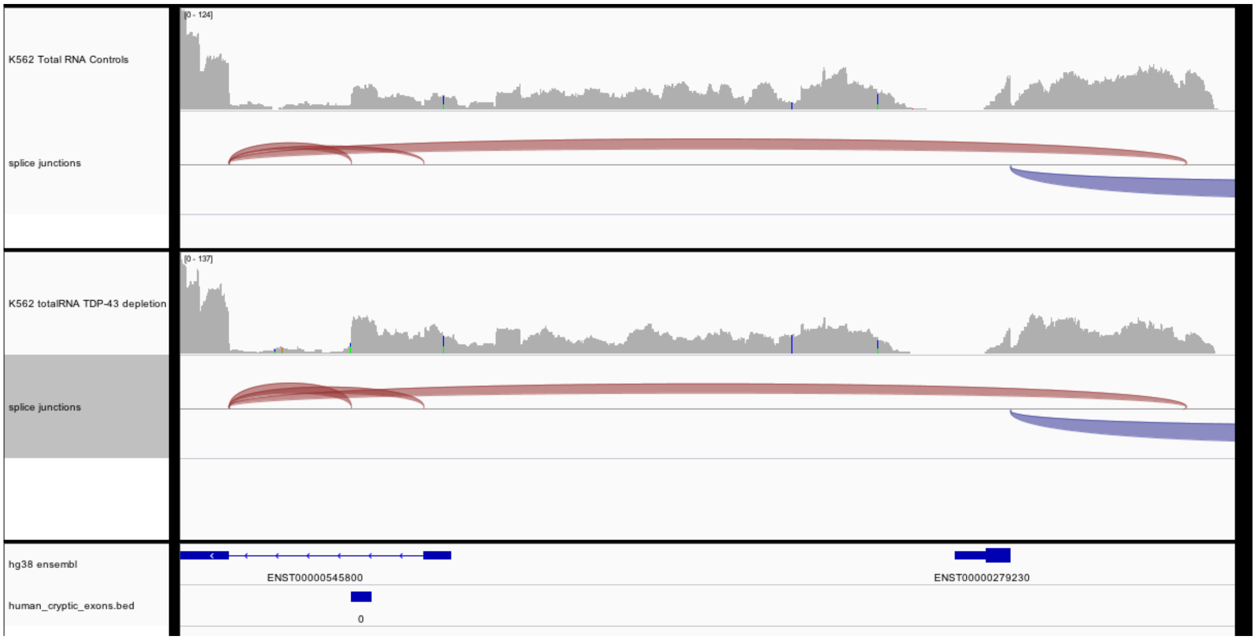

91 SLC19A1 E028i1

Human K562 mRNA

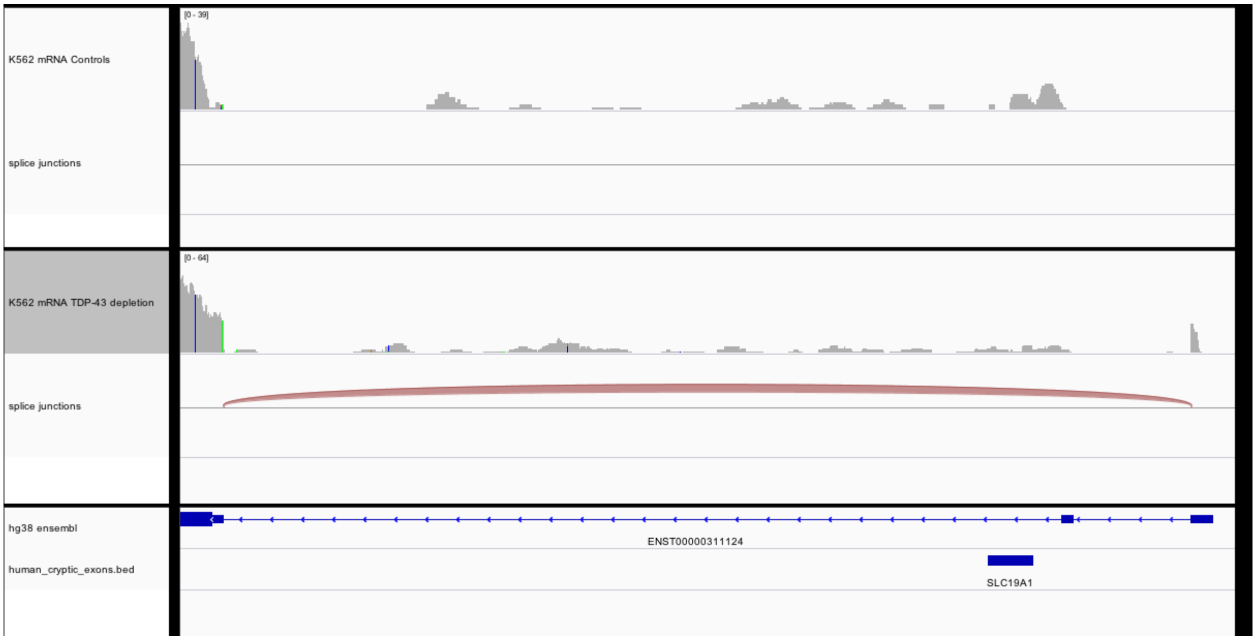

Human K562 total RNA

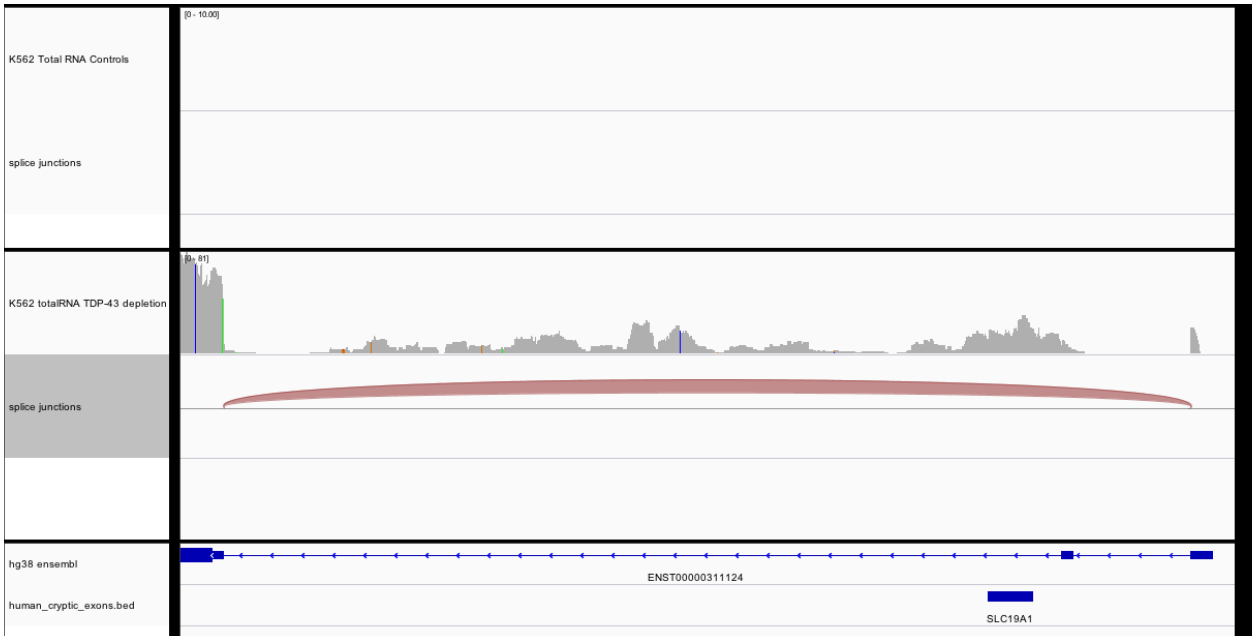

92 HNRNPH3 E009i1

Human K562 mRNA

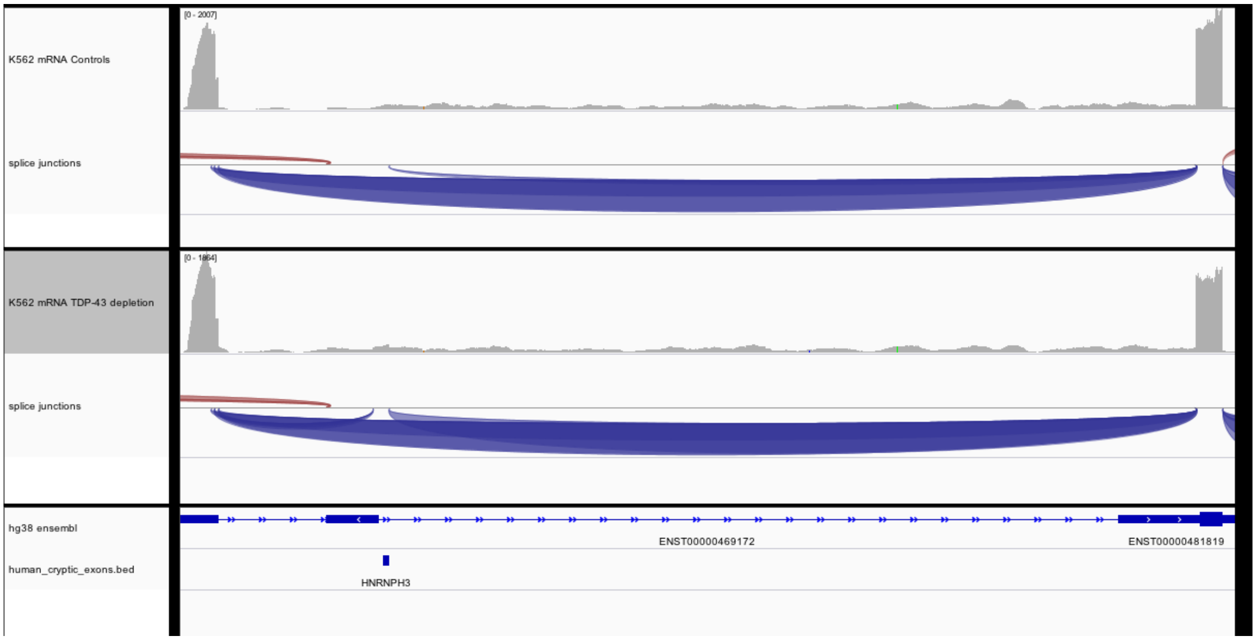

Human K562 total RNA

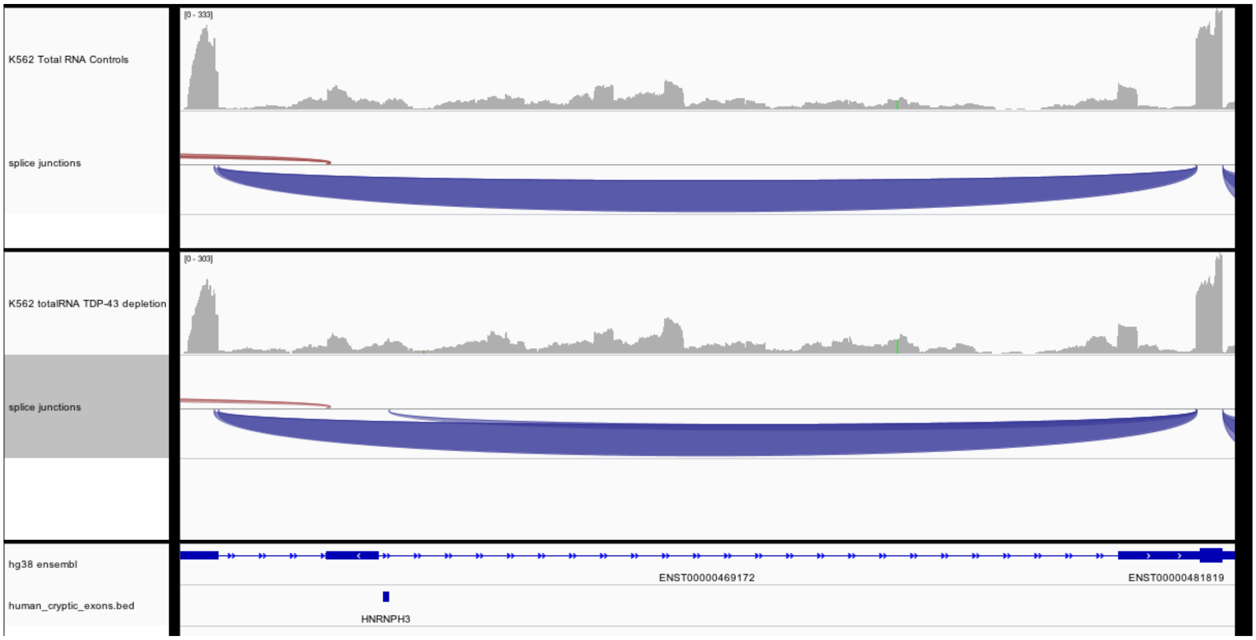

93 SLC39A8 E016i2

Human K562 mRNA

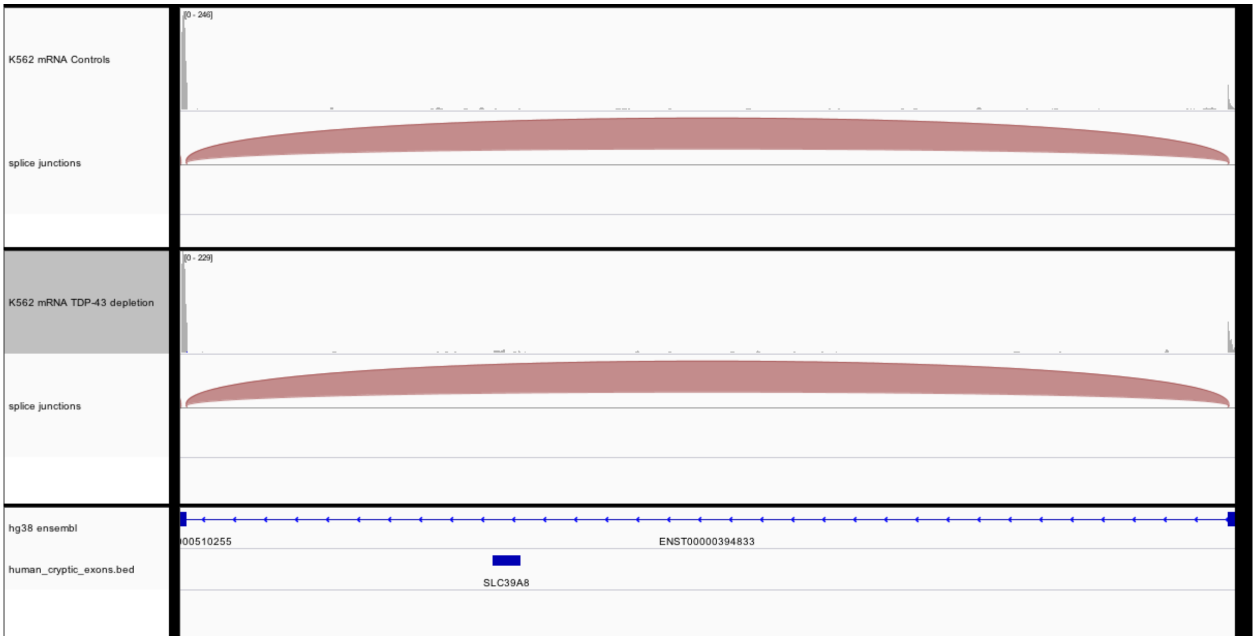

Human K562 total RNA

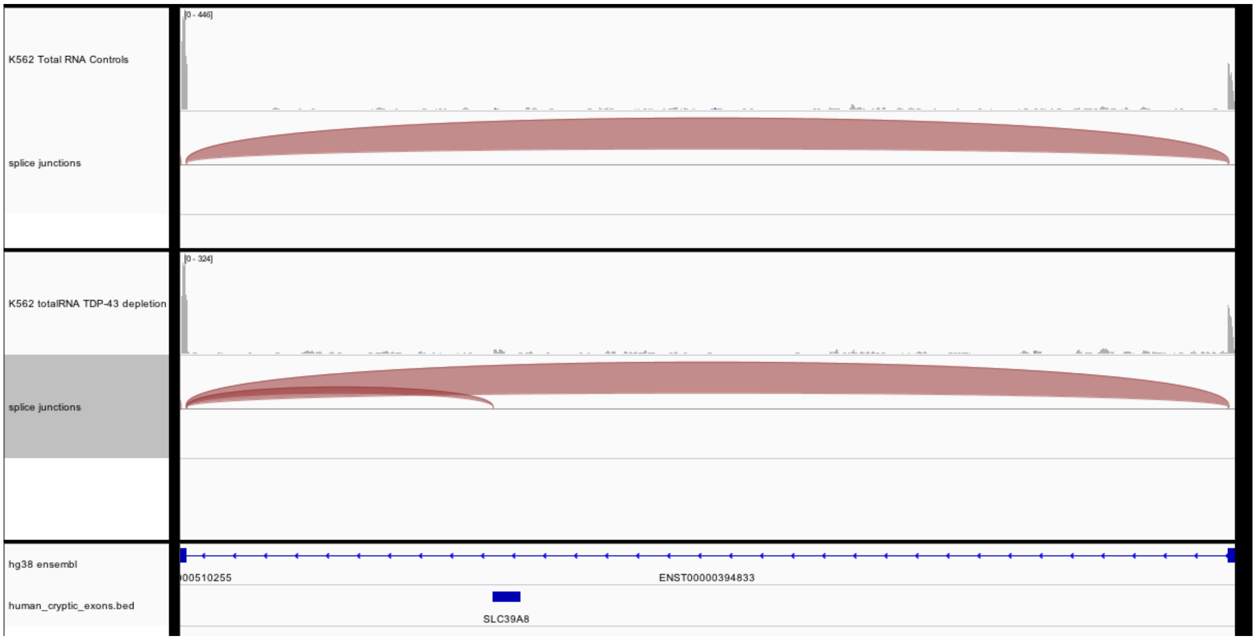

94 DSC2 E017i1

Human K562 mRNA

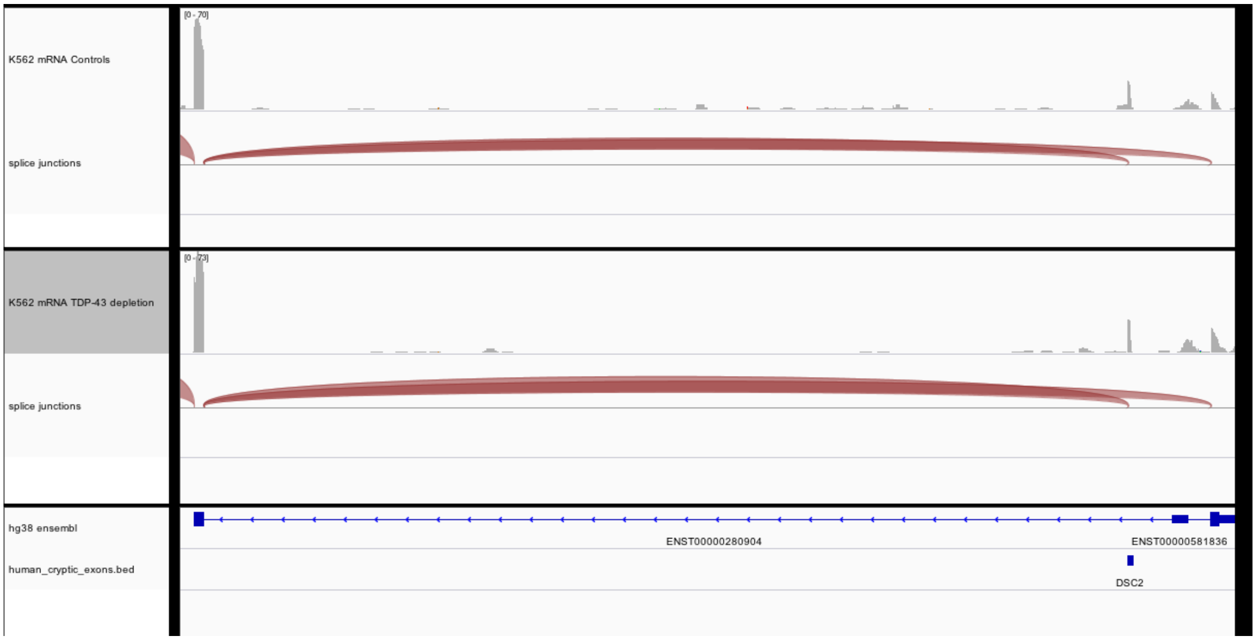

Human K562 total RNA

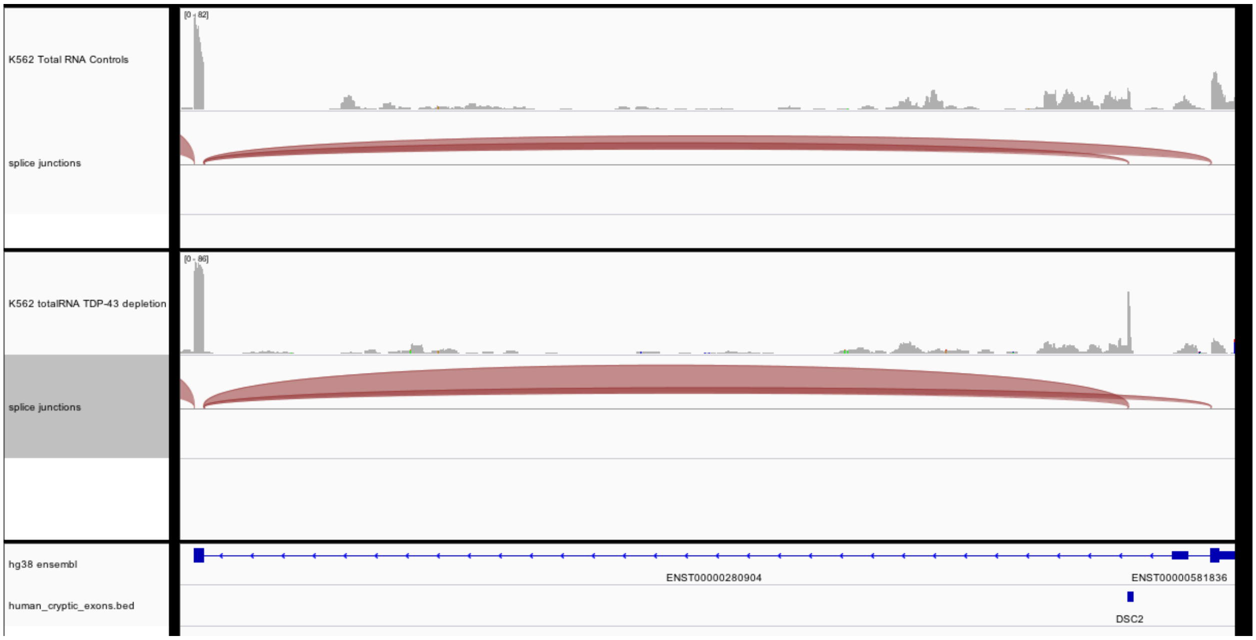

95 ANXA3 E023i1

Human K562 mRNA

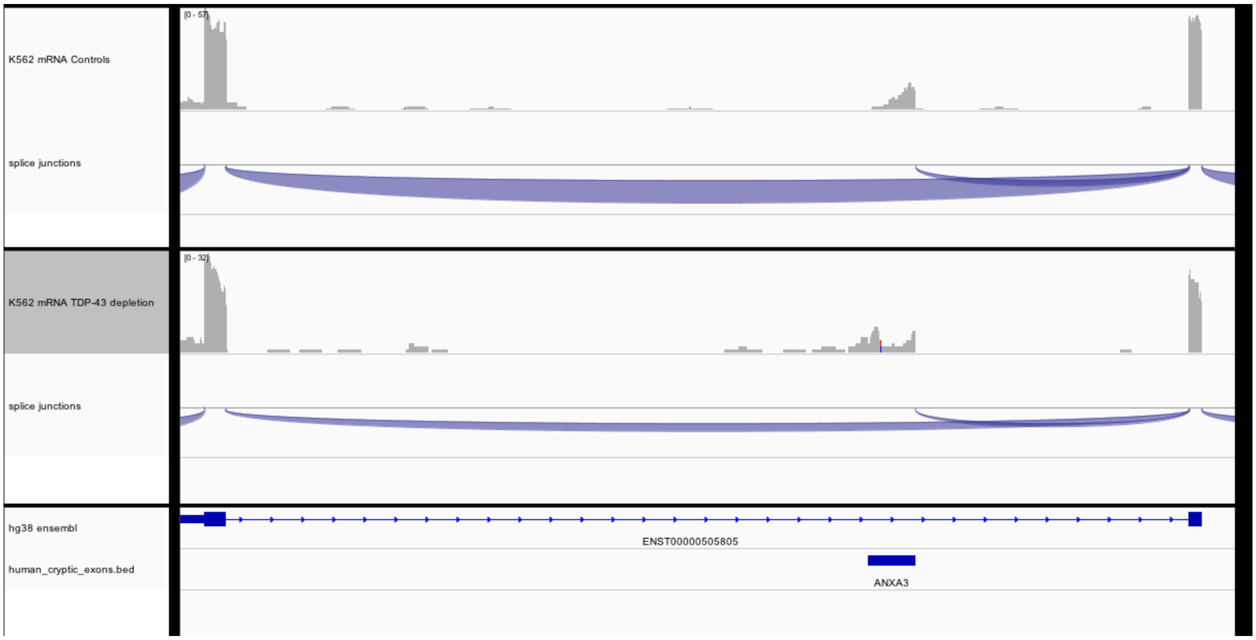

Human K562 total RNA

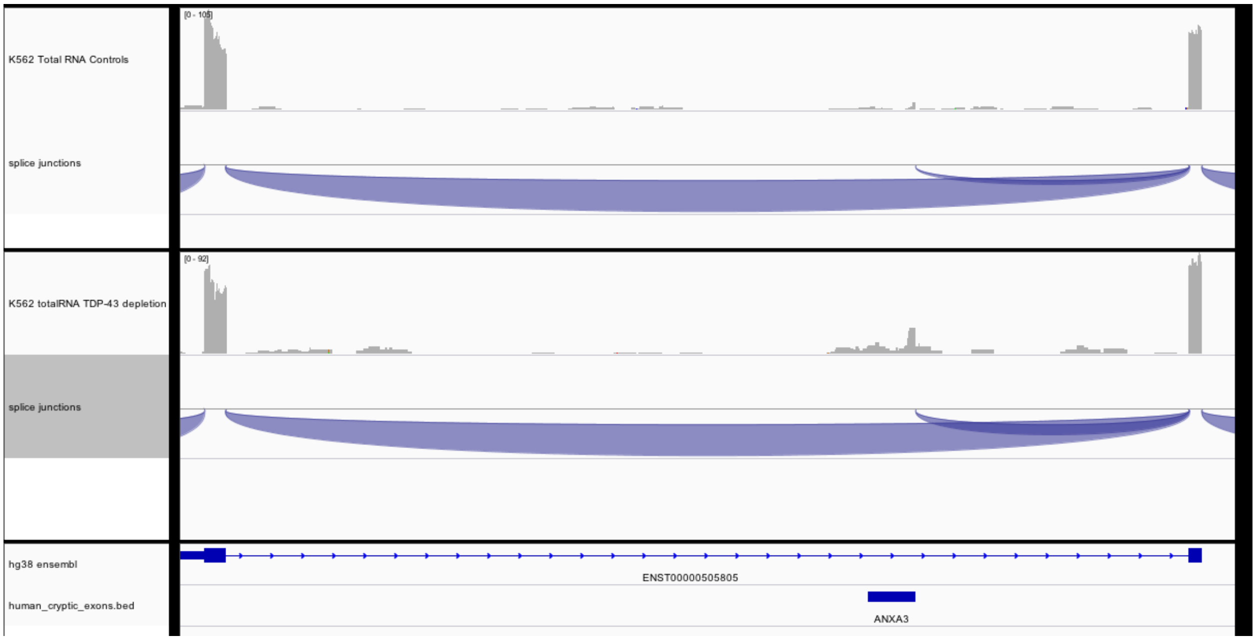

Supplement: Supplementary file 3 — Each cryptic exon discovered in either of the two human K562 datasets. As above, with the hg38 human genome build. (PDF 5917 kb) [file 12920_2017_274_MOESM3_ESM.pdf]
