# Supplementary material for: Quantitative analysis of cryptic splicing associated with TDP-43 depletion
Source: BMC Med Genomics. 2017 May 26;10:38. doi: 10.1186/s12920-017-0274-1 (PMC5446763; doi:10.1186/s12920-017-0274-1)
Supplement: Supplementary file 5 — Inclusion of cryptic exons in normal mouse (ST5) and human (ST6) tissues. RNA-seq data from multiple mouse tissues from [52] and multiple human tissues from the GTEx project [53] was downloaded from the Sequence Read Archive (accession PRJNA177791) and dbGap (phs000424.v6.p1) respectively. All samples were aligned with STAR using two-pass mapping. The resulting junction coordinates were grouped into overlapping clusters for each tissue using Leafcutter [31]. A custom R script then queried the resulting junction tables for evidence of junctions belonging to the previously discovered mouse and human cryptic exons, as well as the canonical junctions in which the cryptic exons splice to and from. The counts of upstream and downstream junctions in each sample were divided by the counts of the canonical junction to produce an inclusion ratio. For each cryptic exon that was detected, the mean inclusion ratio for each tissue is presented with the standard deviation. For the 52 mouse cryptic exons, only 4 showed any evidence of being included in normal tissues. Reep3 E001i1 and Adipor2 E010i2 are included in a few tissues at very low levels. Elmod1 E003i2 is seen in brain tissue at an inclusion rate above 10%, whereas Thoc7 E007i1 was included in every tissue bar heart. However, the inclusion rates were highly variable. Among the 95 human cryptic exons, only three were detected in 49 different human tissues from the GTEx project. MPDZ E015i1, UHRF2 E017i2 and DEAF1 E005i2 were all detected in at least 1 tissue. Only UHRF2 E017i2 was regularly seen above 10% inclusion, albeit with very high variance between samples. (PDF 66 kb) [file 12920_2017_274_MOESM5_ESM.pdf]

Table S5:
Mouse cryptic exon inclusion rates in other mouse tissues from *Merkin et al, 2012*

Tissue (samples)	***Reep3*** E001i1		***Adipor2*** E010i2		***Elmod1*** E003i2		***Thoc7*** E007i1	
	5'	3'	5'	3'	5'	3'	5'	3'
brain (3)	0	0	0.074±0.047	0	0.17±0.26	0.11±0.09	0.59±0.29	0.44±0.17
colon (3)	0	0.048±0.043	0.028±0.01	0	0	0	0.14±0.063	0.094±0.013
heart (2)	0	0	0	0	0	0	0	0
kidney (3)	0	0	0	0	0	0	0.25±0.091	0.2±0.074
liver (2)	0	0.06±0.049	0.017±0.0067	0.007±0.0028	0	0	0.21±0.018	0.14±0.024
lung (3)	0	0	0	0	0	0	0.16±0.18	0.13±0.051
muscle (2)	0	0	0	0	0	0	0.32±0.27	0.17±0.0023
spleen (3)	0	0	0	0	0	0	0.1±0.077	0
testes (3)	0	0	0.086±0.083	0	0	0	1.2±0.48	0.53±0.21

Table S6:
Human cryptic exon inclusion rates in other human tissues from the GTEx project

Tissue (samples)	***MPDZ*** E015i1		***UHRF2*** E017i2		***DEAF1*** E006i2	
	5'	3'	5'	3'	5'	3'
adipose subcutaneous (239)	0	0.023±0.074	0.15±0.12	0.12±0.095	0.011±0.018	0.0069±0.013
adipose visceral (165)	0	0.02±0.08	0.12±0.13	0.12±0.15	0.013±0.024	0.0068±0.013
adrenal gland (97)	0	0.0076±0.021	0.087±0.098	0.057±0.074	0.021±0.026	0.011±0.013
artery aorta (158)	0	0.016±0.087	0.085±0.09	0.079±0.09	0.012±0.019	0.0062±0.011
artery coronary (89)	0	0.0061±0.016	0.11±0.1	0.11±0.12	0.0079±0.012	0.003±0.0072
artery tibial (221)	0	0.015±0.077	0.11±0.1	0.085±0.091	0.0062±0.015	0.0033±0.0088
brain amygdala (61)	0	0.011±0.071	0.054±0.15	0	0.0024±0.0038	0.0018±0.0029
brain anterior cingulate cortex (72)	0	0.0072±0.037	0	0.033±0.054	0.0011±0.0017	0.0016±0.0017
brain caudate (94)	0	0.01±0.049	0.03±0.059	0.01±0.036	0.0019±0.0029	0.002±0.0028
brain cerebellar hemisphere (84)	0	0.036±0.17	0.014±0.028	0.083±0.087	0.013±0.011	0.0081±0.0067
brain cerebellum (108)	0	0.052±0.28	0.021±0.05	0.093±0.089	0.015±0.027	0.008±0.012
brain cortex (98)	0	0.018±0.11	0.018±0.042	0.045±0.09	0±0.003	0±0.0038
brain frontal cortex (89)	0	0.014±0.071	0	0.02±0.036	0	0.0014±0.0021
brain hippocampus (76)	0	0.017±0.099	0.012±0.03	0.012±0.04	0.0016±0.0029	0.0018±0.0029
brain hypothalamus (76)	0	0.013±0.061	0.0053±0.017	0.03±0.056	0.0073±0.0087	0.0047±0.0057
brain nucleus accumbens (89)	0	0.0063±0.035	0.0061±0.02	0.023±0.048	0.002±0.0038	0.0026±0.0035
brain putamen (76)	0	0.017±0.086	0	0.019±0.038	0.0026±0.0059	0.0021±0.0038
brain spinal cord (58)	0	0.009±0.022	0.018±0.035	0.059±0.089	0.0049±0.011	0.0054±0.008
brain substantia nigra (52)	0	0.026±0.11	0	0.037±0.065	0.011±0.016	0.0042±0.007
breast mammary tissue (136)	0	0.018±0.054	0.16±0.15	0.13±0.11	0.018±0.039	0.012±0.019
cells ebv-transformed lymphocytes (57)	0	0	0.18±0.11	0.19±0.11	0.022±0.027	0.012±0.015
cells transformed fibroblasts (157)	0	0.024±0.081	0.061±0.058	0.055±0.055	0.022±0.028	0.016±0.021
colon sigmoid (108)	0	0.017±0.085	0.092±0.092	0.084±0.085	0.01±0.019	0.0029±0.0073
colon transverse (132)	0	0.0081±0.018	0.13±0.16	0.12±0.13	0.015±0.027	0.0075±0.019
esophagus gastroesophageal junction (119)	0	0.021±0.12	0.1±0.12	0.084±0.097	0.0061±0.012	0.0026±0.007
esophagus mucosa (185)	0	0.014±0.045	0.13±0.12	0.1±0.11	0.039±0.05	0.013±0.02
esophagus muscularis (154)	0	0.018±0.069	0.074±0.079	0.062±0.07	0.0074±0.016	0.0057±0.011
heart atrial appendage (149)	0	0.011±0.028	0.053±0.081	0.049±0.077	0.0086±0.022	0.0044±0.011
heart left ventricle (149)	0	0.013±0.034	0.05±0.072	0.043±0.068	0.0087±0.019	0.0054±0.015
kidney cortex (22)	0	0	0.047±0.073	0.077±0.14	0.018±0.024	0.0094±0.018
liver (82)	0	0.024±0.11	0.052±0.069	0.068±0.12	0.04±0.066	0.011±0.025
lung (233)	0	0.034±0.18	0.15±0.15	0.13±0.12	0.021±0.038	0.0063±0.016
minor salivary gland (46)	0	0	0.17±0.15	0.14±0.13	0.017±0.027	0.011±0.014
muscle skeletal (305)	0	0.031±0.052	0.036±0.069	0.035±0.059	0.0037±0.011	0.0046±0.014
nerve tibial (219)	0	0.02±0.074	0.1±0.092	0.089±0.086	0.0093±0.016	0.0056±0.011
ovary (68)	0	0.01±0.015	0.062±0.063	0.063±0.067	0.0073±0.011	0.003±0.007
pancreas (107)	0	0.012±0.033	0.13±0.14	0.11±0.13	0.013±0.026	0.0062±0.013
pituitary (90)	0	0.032±0.17	0.076±0.11	0.054±0.07	0.021±0.021	0.0092±0.012
prostate (68)	0	0.018±0.072	0.11±0.14	0.11±0.13	0.031±0.053	0.01±0.017
skin not sun exposed (195)	0	0.018±0.037	0.16±0.17	0.14±0.13	0.015±0.02	0.0064±0.011
skin sun exposed (255)	0	0.025±0.073	0.16±0.15	0.12±0.11	0.015±0.02	0.0066±0.011
small intestine terminal ileum (56)	0	0.009±0.025	0.31±0.25	0.24±0.22	0.033±0.04	0.0087±0.016
spleen (73)	0	0.0097±0.03	0.16±0.17	0.16±0.18	0.039±0.038	0.011±0.021
stomach (114)	0	0.015±0.069	0.13±0.11	0.1±0.11	0.013±0.018	0.0085±0.017
testis (112)	0	0.03±0.042	0.11±0.11	0.084±0.091	0.024±0.037	0.011±0.015
thyroid (222)	0	0.018±0.096	0.14±0.14	0.11±0.12	0.016±0.021	0.0081±0.011
uterus (56)	0	0.0077±0.012	0.073±0.091	0.09±0.096	0.014±0.027	0.0072±0.015
vagina (62)	0	0.0078±0.017	0.084±0.075	0.086±0.077	0.013±0.025	0.0062±0.01
whole blood (279)	0	0	0.14±0.13	0.14±0.15	0.093±0.16	0.016±0.038
