# Supplementary material for: Quantitative analysis of cryptic splicing associated with TDP-43 depletion
Source: BMC Med Genomics. 2017 May 26;10:38. doi: 10.1186/s12920-017-0274-1 (PMC5446763; doi:10.1186/s12920-017-0274-1)
Supplement: Supplementary file 7 — All repeat elements enriched in mouse (ST3) and human (ST4) cryptic exons. For each class of repeat element that was enriched in a set of cryptic exons, the exact overlapping repeat element was compiled into a table. The strand column refers to the orientation of the gene, not the repeat element. For the simple repeats, all annotations are made in the positive direction. Therefore (AC)n containing repeats in the antisense direction are in fact (GT)n repeats for genes on the negative strand. Figure S5. The RNA sequence of the B2 SINE in the antisense orientation. The DNA sequence of the B2 SINE was downloaded from lncrnadb [71] and converted to the reverse complement to get its antisense orientation. Ts were changed to Us to reflect the RNA sequence. Stretches of UG and GU are highlighted in red. (PDF 123 kb) [file 12920_2017_274_MOESM7_ESM.pdf]

Table S3: repeat families enriched in mouse cryptic exons

| chr | start | end | *geneID* | exonID | strand | repeat family | repeat type |
|---|---|---|---|---|---|---|---|
| chr1 | 21229021 | 21229273 | *Tmem14a* | E005i1 | + | antisense B2 SINE | B3A |
| | 24022406 | 24022770 | *Fam135a* | E008i1 | - | antisense B2 SINE | B2 Mm1t |
| | 24022406 | 24022770 | *Fam135a* | E008i1 | - | antisense B2 SINE | B3 |
| | 24022406 | 24022770 | *Fam135a* | E008i1 | - | antisense B2 SINE | B3 |
| | 193368766 | 193369053 | *Camk1g* | E017i2 | - | simple repeat | (CA)n |
| | 24022406 | 24022770 | *Fam135a* | E008i1 | - | simple repeat | (CA)n |
| chr2 | 58049385 | 58049662 | *Ermn* | E001i1 | - | antisense B2 SINE | B3 |
| | 24691859 | 24692466 | *Cacna1b* | E050i1 | - | low complexity | AT rich |
| | 58049385 | 58049662 | *Ermn* | E001i1 | - | low complexity | AT rich |
| | 165183138 | 165183472 | *Cdh22* | E013i2 | - | simple repeat | (TTCA)n |
| | 24691859 | 24692466 | *Cacna1b* | E050i1 | - | simple repeat | (CA)n |
| | 24691859 | 24692466 | *Cacna1b* | E050i1 | - | simple repeat | (TTTA)n |
| chr3 | 152292456 | 152292758 | *Fam73a* | E015i2 | - | antisense B2 SINE | B3 |
| | 152643769 | 152644016 | *Ak5* | E014i6 | - | simple repeat | (TGGGGG)n |
| chr5 | 52434150 | 52434525 | *Ccdc149* | E009i3 | - | low complexity | GA-rich |
| | 122556832 | 122557092 | *Ift81* | E003i1 | - | low complexity | T-rich |
| | 122556832 | 122557092 | *Ift81* | E003i1 | - | simple repeat | (CA)n |
| | 52434150 | 52434525 | *Ccdc149* | E009i3 | - | simple repeat | (TG)n |
| chr6 | 119369759 | 119370019 | *Adipor2* | E010i2 | - | antisense B2 SINE | B3 |
| | 120204163 | 120204566 | *B4galnt3* | E002i1 | - | simple repeat | (CA)n |
| chr7 | 34110974 | 34111200 | *Wtip* | E001i1 | - | antisense Alu SINE | B1 Mus1 |
| | 34110974 | 34111200 | *Wtip* | E001i1 | - | antisense B2 SINE | B2 Mm2 |
| | 81490078 | 81490378 | *Ap3b2* | E031i2 | - | simple repeat | (CAA)n |
| | 81490078 | 81490378 | *Ap3b2* | E031i2 | - | simple repeat | (CA)n |
| chr9 | 109837772 | 109838325 | *Nme6* | E020i1 | + | antisense B4 SINE | RSINE1 |
| | 78207096 | 78207339 | *Gsta4* | E005i2 | + | antisense B2 SINE | B3 |
| | 60637548 | 60637770 | *Lrrc49* | E017i3 | - | simple repeat | (CA)n |
| chr10 | 45639667 | 45639904 | *Hace1* | E013i1 | + | antisense B2 SINE | B3 |
| | 67013987 | 67014265 | *Reep3* | E001i1 | - | antisense ID SINE | ID4 |
| | 71263484 | 71263818 | *Ube2d1* | E010i1 | - | antisense B2 SINE | B2 Mm2 |
| | 71263484 | 71263818 | *Ube2d1* | E010i1 | - | antisense B2 SINE | B3A |
| | 123150861 | 123151225 | *Usp15* | E015i3 | - | simple repeat | (CA)n |
| | 67013987 | 67014265 | *Reep3* | E001i1 | - | simple repeat | (CCAA)n |
| | 71263484 | 71263818 | *Ube2d1* | E010i1 | - | simple repeat | (AGGGGG)n |
| | 71263484 | 71263818 | *Ube2d1* | E010i1 | - | simple repeat | (TAAA)n |
| chr11 | 29421254 | 29421502 | *Ccdc88a* | E004i8 | + | antisense Alu SINE | B1F1 |
| | 29421254 | 29421502 | *Ccdc88a* | E004i8 | + | antisense Alu SINE | B1 Mus1 |
| | 76468424 | 76468678 | *Abr* | E026i1 | - | simple repeat | (CA)n |
| | 76468424 | 76468678 | *Abr* | E026i1 | - | simple repeat | (GGGA)n |
| chr12 | 55864889 | 55865192 | *Brms1l* | E008i1 | + | antisense B4 SINE | RSINE1 |
| | 81509727 | 81510152 | *Synj2bp+Gm20498* | E036i2 | - | antisense B2 SINE | B3A |
| | 81509727 | 81510152 | *Synj2bp+Gm20498* | E036i2 | - | low complexity | AT rich |
| | 81509727 | 81510152 | *Synj2bp+Gm20498* | E036i2 | - | simple repeat | (CA)n |
| chr13 | 103862427 | 103862688 | *Erbb2ip* | E026i1 | - | antisense B2 SINE | B3 |
| chr14 | 13957359 | 13957732 | *Thoc7* | E007i1 | - | antisense B2 SINE | B3A |
| | 13957359 | 13957732 | *Thoc7* | E007i1 | - | antisense B4 SINE | B4A |
| chr15 | 12833121 | 12833490 | *Drosha* | E009i1 | + | antisense B4 SINE | B4 |
| chr16 | 45389870 | 45390285 | *Cd200* | E003i1 | - | simple repeat | (CA)n |
| chr17 | 34739289 | 34739599 | *C4b* | E032i1 | - | antisense B4 SINE | B4A |
| | 34739289 | 34739599 | *C4b* | E032i1 | - | simple repeat | (CA)n |
| chr18 | 6986038 | 6986336 | *Mkx* | E007i7 | - | simple repeat | (CA)n |
| | 80138052 | 80138405 | *Adnp2* | E002i1 | - | simple repeat | (CA)n |

Figure S5:
The nucleotide sequence of B2 SINE in the antisense orientation

AAAGAUUUAUUUAUUUAUUAUAUGUAAGUACACUGUAGC
UGUCUUCAGACACUCCAGAAGAGGGAAUCAGAUCUCGUU
ACGGAUGGUUGUGAGCCACCAUGUGGUUGCUGGGAUUU
GAACUCCUGACCUUCGGAAGAGCAGUCGGGUGCUCUUA
CCCACUGAGCCAUCUCACCAGCC

Table S4: repeat families enriched in human cryptic exons

| chr | start | end | geneID | exonID | strand | repeat type | repeat class |
|---|---|---|---|---|---|---|---|
| chr1 | 19145498 | 19145779 | *UBR4* | E084i1 | - | simple repeat | (AC)n |
| chr2 | 111799429 | 111799800 | *ANAPC1* | E020i1 | - | simple repeat | (AATCCAA)n |
| | 241668774 | 241669086 | *ATG4B* | E047i1 | + | simple repeat | (GT)n |
| | 43942575 | 43942904 | *LRPPRC* | E024i1 | - | simple repeat | (ATTTT)n |
| chr3 | 121682816 | 121683064 | *GOLGB1* | E013i1 | - | simple repeat | (T)n |
| chr4 | 102324149 | 102325099 | *SLC39A8* | E016i2 | - | simple repeat | (ATAC)n |
| chr7 | 783125 | 783410 | *DNAAF5* | E024i1 | + | simple repeat | (CGTGAG)n |
| chr10 | 27098079 | 27098427 | *ANKRD26* | E045i1 | - | simple repeat | (TTTAATT)n |
| | 3099456 | 3099920 | *PFKP* | E010i1 | + | simple repeat | (GT)n |
| chr11 | 58616892 | 58617155 | *ZFP91+ZFP91-CNTF* | E012i1 | + | simple repeat | (TG)n |
| chr12 | 116789933 | 116790188 | *RNFT2* | E016i2 | + | simple repeat | (TGGA)n |
| | 88086609 | 88086890 | *CEP290* | E024i1 | - | simple repeat | (ATCC)n |
| chr14 | 53096016 | 53096319 | *DDHD1* | E023i1 | - | simple repeat | (AGGG)n |
| chr16 | 4367197 | 4367543 | *CORO7-PAM16+CORO7* | E081i1 | - | simple repeat | (GAGGG)n |
| chr19 | 4491914 | 4492253 | *HDGFRP2* | E015i1 | + | simple repeat | (GT)n |
| chr21 | 43792282 | 43792675 | *RRP1* | E008i1 | + | simple repeat | (GTGA)n |
